# Switching Sides: Regiochemistry and Functionalization Dictate the Photoswitching Properties of Imines

**DOI:** 10.1002/anie.202415464

**Published:** 2024-11-02

**Authors:** Jiarong Wu, Lasse Kreimendahl, Jake L. Greenfield

**Affiliations:** ^1^ Institut für Organische Chemie Universität Würzburg 97074 Würzburg Germany; ^2^ Center for Nanosystems Chemistry (CNC) Universität Würzburg 97074 Würzburg Germany

**Keywords:** Photoswitch, Photoisomerization, Imine, Dynamic-Covalent Chemistry, Aryliminopyrazole

## Abstract

Photoswitchable imines demonstrate light‐dependent dynamic covalent chemistry and can function as molecular ratchets. However, the design of aryliminopyrazoles (AIPs) has been limited to *N*‐pyrazole derivatives with *ortho*‐pyrrolidine motifs. The impact of other functionalization patterns on the photoswitching properties remains unknown. Here, we present a systematic structure‐property analysis and study how the photoswitching properties can be tuned through *ortho*‐ and *para*‐functionalization of the phenyl ring in *N*‐pyrazole and *N*‐phenyl AIPs. This study establishes the first set of design rules for these AIP photoswitches and reports the most stable *Z*‐isomer of an AIP to date, enabling its crystallization and resulting in the first reported crystal structure of a metastable *Z*‐aldimine. Finally, we demonstrate that the AIPs are promising candidates for photoswitching in the condensed phase.

## Graphical abstract

**You know what imine**? Aryliminopyrazoles have demonstrated their suitability as photoswitches and in driving dynamic‐covalent reactions out‐of‐equilibrium. However, the influence of their structure on their photoswitching properties remains unknown. Here, we present the first comprehensive investigation into their structure–property relationship, a detailed list of design rules, and their ability to switch in the condensed phase.

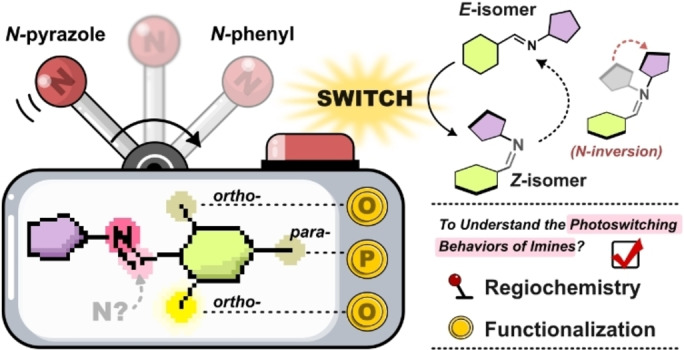

## Introduction

Photochromic molecules are essential components of photon‐driven molecular machines and light‐responsive systems.[^1^, ^2^, ^3^, ^4^, ^5^] Among the approaches to creating such systems,[^6^, ^7^] molecular photoswitches—compounds that switch between two or more states in response to light—are particularly favored.^[1]^ Photoswitches exhibiting *E*/*Z* isomerism are among the most extensively studied and applied classes,[^8^, ^9^, ^10^, ^11^, ^12^, ^13^, ^14^] typically due to the significant geometric change associated with this type of isomerism.^[15]^ Notable members of this class include azobenzenes,[^8^, ^16^] azoheteroarenes,[^17^, ^18^, ^19^, ^20^] arylhydrazones,[^10^, ^21^] and stilbenes,[^12^, ^22^] each displaying a unique combination of photoswitching properties.[^11^, ^23^] Functionalizing the photochromic core can further tune these properties, such as the photostationary state (PSS) distribution, effective switching wavelengths, and thermal half‐life (*t*_1/2_).[^15^, ^19^, ^24^]

In 2024, we discovered a new class of imine‐based photoswitches[^25^, ^26^, ^27^, ^28^] with improved photoswitching properties: the aryliminopyrazoles (AIPs).^[29]^ These aldimine‐based switches can be prepared quantitatively from commercially available precursors and exhibit *E*/*Z* photoisomerism about the C=N bond.^[30]^ Improvements in photoswitching properties, compared to previously reported aldimines,[^28^, ^31^, ^32^] included quantitative *E*‐to‐*Z* photoswitching with visible light, resistance to fatigue, and a *t*_1/2_ extending to 19.2 hours at 20 °C.^[29]^ Utilizing the dynamic‐covalent properties of the imine bond, we found that a transimination equilibrium could be driven to a non‐equilibrium steady state (NESS) under photoirradiation.[^33^, ^34^] This system exhibited characteristics of an autonomously cycling, light‐driven information ratchet,[^2^, ^3^, ^4^] marking the first example of directly coupling a photochemical process to an imine exchange process, reaching a NESS under photoirradiation.^[34]^ This discovery positions AIPs as promising candidates for light‐responsive systems chemistry,[^35^, ^36^, ^37^, ^38^, ^39^] provided that their photoswitching properties exhibit the necessary versatility and tunability to fit the intended applications.^[15]^

To date, functionalization of AIPs has been limited to structures with an *ortho*‐pyrrolidine unit on the phenyl ring,^[29]^ leaving a vast functionalization space unexplored. In the context of azobenzene, pioneering studies by Woolley,[^40^, ^41^, ^42^] Hecht[^8^, ^24^] and others[^43^, ^44^] have shown that functionalizing azo‐based photoswitches at the *ortho‐* and *para*‐positions with electron‐donating (EDGs) and electron‐withdrawing groups (EWGs) yields tunable photochromic properties. This has also been an area of intense interest for the more recently reported azoheteroarenes,[^17^, ^19^, ^45^, ^46^, ^47^, ^48^, ^49^, ^50^] hydrazone photoswitches,[^10^, ^51^] hemithioindigos,[^52^, ^53^, ^54^] and acylhydrazones.^[11]^

Differences in structure–property relationships have already emerged between AIPs and their azo‐counterparts: *ortho*‐amination enhances the *t*_1/2_ for AIPs but decreases it for azo‐based photoswitches.[^43^, ^55^] Additionally, imines display lower symmetry about the photochromic bond (C=N), resulting in positional isomers, specifically regioisomers, providing an additional handle for tuneability.

**Figure 1:**
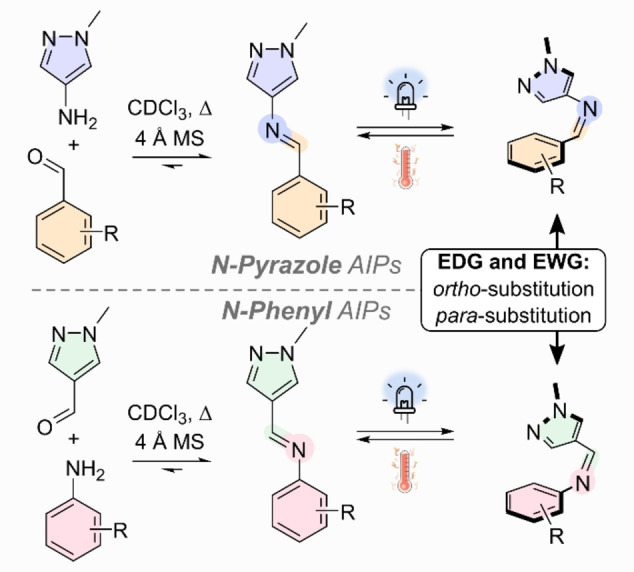
Schematic of the AIPs studied here.

Here, we report a systematic study into the switching properties of AIPs (Figure 1). We show that inverting the directionality of the imine bond with respect to the phenyl and pyrazole ring—i.e., *N*‐pyrazole and *N*‐phenyl analogs—results in significantly different photoswitching properties. By investigating various EWG and EDG groups at the *ortho‐* and *para*‐positions of the phenyl ring, we establish a set of design rules to tune the photophysical properties. Finally, we demonstrate that the AIPs can photoisomerize in the condensed phase. This study provides the first set of design rules for this recently discovered class of photoswitch, allowing the photochromic core to be rationally tailored to achieve customized photoswitching properties.

## Results and Discussion

### Synthesis and X‐ray Crystallography

The imine‐based photoswitches listed in Table 1 were synthesized in one step from their respective amine and aldehyde precursors and isolated as the thermodynamically stable *E*‐isomer (see Supporting Information).^[29]^ The photoswitching properties are summarized in Table 2 and reported more extensively in Section 4 of the Supporting Information. This includes action plots and quantum yields of photoisomerism.

**Table 1:** The *N*‐pyrazole (**1 a**–**o**) and *N*‐phenyl (**2 a**–**o**) AIPs explored.

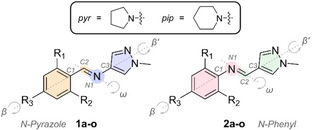			
Compound	R_1_	R_2_	R_3_
**a** ^*a,b*^	H	H	H
**b** ^*a,b*^	H	H	OMe
**c** ^*a,b*^	H	H	NO_2_
**d** ^*b*^	H	H	NMe_2_
**e** ^*a,b*^	*pyr*	H	H
**f** ^*a,b*^	*pyr*	*pyr*	H
**g** ^*a*^	NMe_2_	H	H
**h** ^*a*^	NMe_2_	NMe_2_	H
**i** ^*a*^	*pip*	H	H
**j** ^*a*^	*pip*	*pip*	H
**k** ^*a,b*^	F	H	H
**l** ^*a,b*^	F	F	H
**m** ^*a,b*^	*pyr*	F	H
**n** ^*a,b*^	*pip*	F	H
**o** ^*a*^	*pyr*	F	OMe

^*a*^ Derivative synthesized for *N*‐pyrazole AIP. ^*b*^ Derivative synthesized for *N*‐phenyl AIP.

**Table 2:** Summary of the *N*‐pyrazole (*left*) and *N*‐phenyl (*right*) AIP's photoswitching properties. PSS values were measured using UV/Vis measurements performed at 20 °C in MeCN. The values in parentheses are not the PSS distribution but the maximum measured %*Z* isomer; the PSS is not reached under these conditions due to significant thermal back isomerization. The %*E*‐isomer can be calculated from 100 %‐%*Z*‐isomer. PSSs recorded at other wavelengths are shown in Table S6. Quantum yields for the *E*‐to‐*Z* and *Z*‐to‐*E* photoisomerism are presented in Table S8.

	λ_max_ (nm)	*ϵ* at λ_max_ (M^−1^ cm^−1^)	*t*_1/2_ (20 °C)	%*Z* at PSS			λ_max_ (nm)	*ϵ* at λ_max_ (M^−1^ cm^−1^)	*t*_1/2_ (20 °C)	%*Z* at PSS	
				365 nm	405 nm					365 nm	405 nm
**1 a** ^*a*^	315	14210	12.5 s	(27 %) ^*b*^	–	**2 a**	300	11450	1.1 min	15 %	–
**1 b**	322	19990	15.9 s	(18 %) ^*b*^	–	**2 b**	319	13780	12.2 min	63 %	9 %
**1 c**	358	17390	4.2 s	(29 %) ^*b*^	(7 %) ^*b*^	**2 c**	337	18450	<0.1 s	– ^*b*^	–
–	–	–	–	–	–	**2 d**	353	15570	12.9 min	71 %	44 %
**1 e** ^*a*^	370	8310	22.1 min	66 %	94 %	**2 e**	371	4470	1.4 min	71 %	75 %
**1 f** ^*a*^	362	6340	19.2 h	68 %	95 %	**2 f**	360	1730	20.03 s	(18 %) ^*b*^	(25 %) ^*b*^
**1 g**	344	8080	6.3 min	58 %	60 %	–	–	–	–	–	–
**1 h**	343	8110	4.7 h	74 %	88 %	–	–	–	–	–	–
**1 i**	345	8020	5.4 min	58 %	57 %	–	–	–	–	–	–
**1 j**	335	8230	25.9 h	70 %	86 %	–	–	–	–	–	–
**1 k**	320	14490	44.4 s	46 %	4 %	**2 k**	304	7560	54.5 s	16 %	–
**1 l**	314	13050	6.8 min	72 %	7 %	**2 l**	300	9450	52.3 s	17 %	–
**1 m**	358	5860	5.5 h	67 %	92 %	**2 m**	361	3810	1.4 min	53 %	53 %
**1 n**	310	13090	1.2 h	84 %	80 %	**2 n**	325	3340	21.5 s	(30 %) ^*b*^	(7 %) ^*b*^
**1 o**	351	10050	4.9 h	78 %	78 %	–	–	–	–	–	–

^*a*^ Previously reported in reference [29]. ^*b*^ Significant thermal reversion at room temperature prevented the attainment of a PSS.

**Figure 2:**
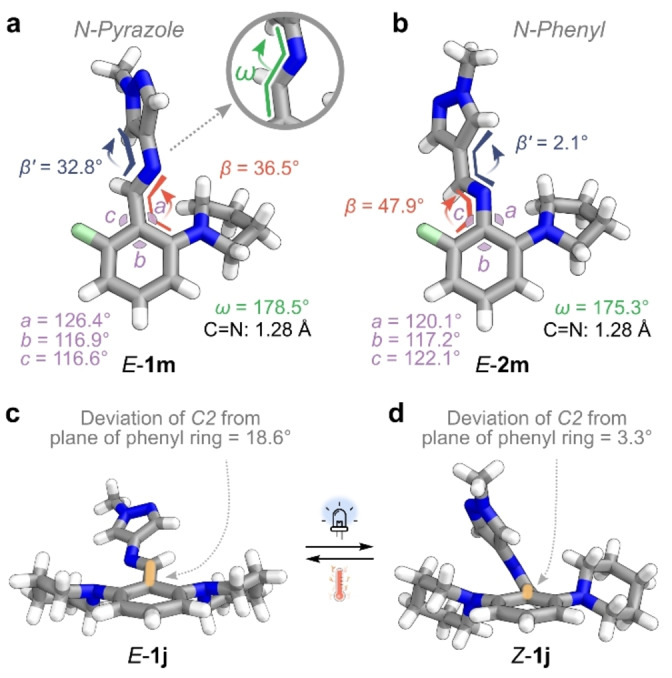
Selected X‐ray crystal structures of AIP photoswitches. **a**, and **b**, The *E*‐isomers of an *N*‐pyrazole and *N*‐phenyl AIP with the same functionalization of the phenyl ring. **c**, The X‐ray crystal structure of *E*‐**1 j**. **d**, The X‐ray crystal structure of the *Z*‐**1 j**.^[56]^

Single crystals of the *E*‐isomers of **1 j**, **1 m**, **1 o**, and **2 m** were obtained by slow solvent evaporation (Figure 2, Table S2). X‐ray crystal structures revealed that the *E*‐isomers adopt a non‐planar conformation (Figure 2),[^28^, ^29^] consistent with DFT geometry‐optimized structures (Section 6 of Supporting Information). Comparing the two regioisomers (**1 m** and **2 m**), the *N*‐phenyl derivative **2 m** shows greater planarity between the pyrazole ring and the imine bond (β’). This is attributed to a reduced steric clash between protons on the pyrazole ring and the imine proton. Additionally, a greater steric clash between the pyrrolidine and imine bond of the *N*‐pyrazole derivative causes the angle marked *“a*” in Figure 2a to deviate further from the ideal value of 120°. This suggests that different behaviors may be expected upon *ortho*‐functionalization of these AIPs.

The single‐crystal X‐ray structure of the *N*‐pyrazole derivative **1 j** was obtained for both the *E‐* and *Z*‐isomer (Figure 2c,d).^[56]^ Crystallization of the *Z*‐isomer was achieved due to the high conversion of the *E*‐ to *Z*‐isomer under irradiation and its enhanced thermal stability (see below). This feat is significant: it confirms that *E*/*Z* isomerism is indeed taking place under visible light irradiation and marks the first crystal structure of a light‐generated metastable *Z*‐aldimine.^[29]^ Notably, the *C1−C2* bond is forced out‐of‐plane of the phenyl ring for *E*‐**1 j** while remaining in‐plane for the *Z*‐isomer.

### Impact of Regiochemistry: *N*‐Pyrazole vs. *N*‐Phenyl AIPs

**Figure 3:**
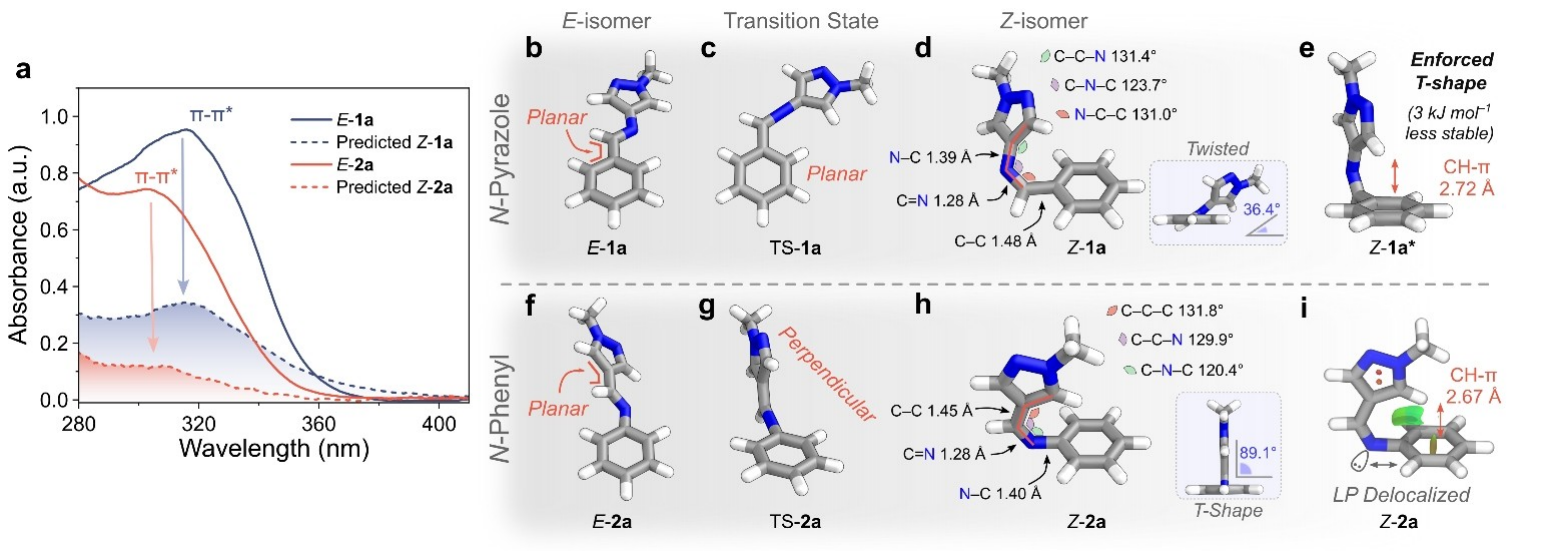
**a**, UV/Vis absorption spectra (l=1 cm, 293 K, MeCN) of *E*‐**1 a** and *E*‐**2 a** and the predicted spectra of their *Z*‐isomers. **b**, DFT‐optimized structure of *E*‐**1 a**. **c**, Optimized TS geometry of **1 a**. **d**, DFT‐optimized structure of *Z*‐**1 a**. **e**, Geometry optimized structure of *Z*‐**1 a** when forced into a T‐shape conformation. **f**, DFT‐optimized structure of *E*‐**2 a**. **g**, Optimized TS geometry of **2 a**. **h**, Geometry optimized structure of *Z*‐. **i**, Structure of *Z*‐**2 a** with NCI surfaces shown. Structures were optimized using a ωB97X−D4 functional with a def2‐TZVPP basis set and a CPCM solvation model for acetonitrile.

Investigating the impact of regiochemistry on the photoswitching properties, AIPs **1 a** and **2 a** were prepared as the simplest model systems: both contain a phenyl and a pyrazole ring but differ in their connectivity to the imine bond. The UV/Vis absorption spectrum of *E*‐**2 a** exhibits a 15 nm hypsochromic shift of the π–π* band compared to the *N*‐pyrazole analog, *E*‐**1 a** (Figure 3a, Table 2). This shift is attributed to *E*‐**1 a** exhibiting greater conjugation from a more planar structure (Figure 3b, f): Theoretical investigations using DFT (ωB97X‐D4 functional with a def2‐TZVPP basis set and a CPCM solvation model for acetonitrile) show that *E*‐**2 a** (β=1°; β’=45°) adopts more of a twisted conformation compared to *E*‐**1 a** (β=2°; β’=5°). This conformational difference is attributed to greater steric clash at the imine bond: the C−H of the imine is directed towards the larger 6‐membered phenyl ring in the *N*‐phenyl AIP, while the same motif is orientated towards the less sterically demanding 5‐membered pyrazole ring in the *N*‐pyrazole AIP (Figure 3b, f).

For both regioisomers, the *Z*‐isomer's UV/Vis absorption spectrum showed a less intense π–π* transition but no significant change in the *λ*_max_ (Figure 3a). Moreover, the thermal stability of *Z*‐**1 a** was low, preventing the attainment of a PSS.^[29]^ Instead, the maximum achievable composition of the *Z*‐isomer under various wavelengths of light irradiation was calculated: a higher proportion of *Z*‐isomer was generated for **1 a** compared to **2 a** under 340 nm and 365 nm irradiation due to the bathochromic shift in its π–π* band. In summary, these simple *N*‐pyrazole and *N*‐phenyl AIPs exhibit similar electronic absorption properties for the *E‐* and *Z*‐isomers; however, sizable differences in the thermal stability of the metastable state were observed.

The *N*‐phenyl derivative, **2 a**, exhibited a five‐fold longer *t*_1/2_ than its *N*‐pyrazole counterpart. Photoswitch *Z*‐**1 a** adopts a twisted conformation stabilized by weak dispersive interactions between the pyrazole and phenyl rings,^[29]^ while *Z*‐**2 a** shows a T‐shape conformation. In the latter, the CH of the pyrazole ring is oriented towards the π‐system of the *N*‐phenyl ring. Non‐covalent interaction (NCI) surfaces show stabilizing CH‐π interactions (Figure 3i), thus lowering the Δ*G*_*Z–E*_ by 13.5 kJ mol^−1^ compared to **1 a** (Section 6 of Supporting Information). This difference in preferred *Z*‐isomer conformation for the two regioisomers is attributed to the overall electronics of the system and the spatial dimensions (Figure 3d, h, Figure S62, S76).

The twisted geometry of *Z*‐**1 a** enables conjugation from the electron‐rich pyrazole unit across the imine and onto the phenyl ring, resulting in net stabilization. In contrast, the T‐shape conformation of *Z*‐**2 a** blocks this conjugation; instead, the imine nitrogen's lone pair of electrons can delocalize into the π‐system of the phenyl ring (Figure 3i). This rationale is supported by DFT investigations: the HOMO/LUMO molecular orbitals of the twisted *Z*‐conformation are delocalized over the entire molecule (Figure S62), while the HOMO of T‐shaped **2 a** is localized to the phenyl ring, and the LUMO to the pyrazole ring (Figure S76).

Bond lengths and angles about the photochromic motif also differ significantly. Those of *Z*‐**2 a** result in a shorter distance between the pyrazole's C−H and the centroid of the phenyl ring compared to a modified *Z*‐**1 a** where a T‐shape conformation has been artificially enforced (2.67 Å for **2 a** vs. 2.72 Å for **1 a**, Figure 3e, i). This reduced distance and greater electron density of the π‐system of the *N*‐phenyl derivative strengthen the CH‐π interaction.[^17^, ^29^] For *N*‐pyrazole to adopt a T‐shaped geometry, the pyrazole ring must planarize with the imine, destabilizing the structure by 3 kJ mol^−1^ compared to its preferred twisted structure (Figure 3d, e). We infer that the combination of electronic properties and spatial dimensions accounts for the differences in the geometry of the *Z*‐isomers.

**Figure 4:**
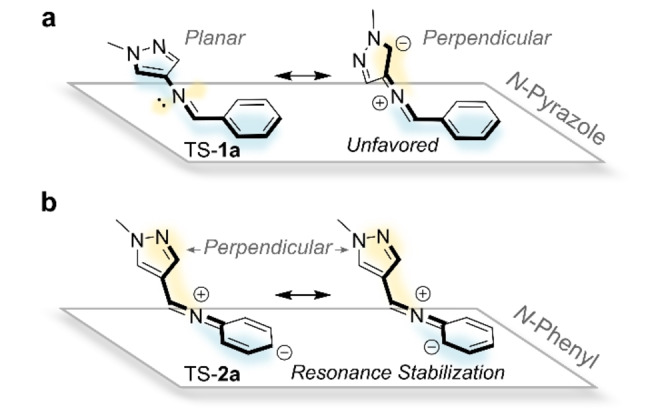
Schematic showing available TS conformations. **a**, *N*‐pyrazole AIPs adopt a planar conformation to hinder the delocalization of the lone pair into the electron‐rich pyrazole ring. **b**, *N*‐phenyl AIPs adopt a perpendicular TS conformation that facilitates resonance stabilization.

For the transition state (TS) structures, both regioisomers exhibit conformations consistent with an inversion pathway, indicated by linearization of the C−N=C(H) motif.[^28^, ^29^, ^30^] However, differences in the orientation of the phenyl and pyrazole rings with respect to the imine are observed. TS‐**1 a** adopts a planar conformation (Figure 3c), while TS‐**2 a** shows a perpendicular arrangement, resulting in a protracted T‐shape (Figure 3g). Previous studies by Haag, Saalfrank, and co‐workers found that the electronics of the imine determines whether a planar or perpendicular TS structure is preferred.^[28]^ Notably, linearization of the C−N=C(H) bond involves rehybridization of the imine nitrogen from *sp* ^2^ to *sp*.[^57^, ^58^] In the perpendicular arrangement, the lone pair on the imine nitrogen can conjugate with the adjacent ring; in the planar state, conjugation with the adjacent ring is not possible (Figure 4a).^[58]^ For the *N*‐phenyl AIP, the lone pair is readily delocalized into the phenyl ring, where resonance leads to net stabilization of the TS (Figure 4b). For the *N*‐pyrazole, delocalization of the lone pair into the electron‐rich pyrazole ring results in destabilization (Figure 4a), which can be avoided in a planar TS. Therefore, the stability of the perpendicular TS will depend more on changes to the electronic properties of the phenyl rings than that of a planar TS.

### Towards a Longer t_1/2_: Stabilize the *Z*‐isomer or Destabilize the TS?

The preferred structures identified for the TSs and *Z*‐isomers of the model *N*‐pyrazole and *N*‐phenyl AIPs (**1 a** and **2 a**) appear general. Collating a library of AIPs based on common functionalization patterns used for azo‐based photoswitches (Table 1), their properties were explored computationally and experimentally (Table 2, S3–S6, S8, and S9). The theoretically predicted *t*_1/2_ values showed excellent agreement with the experimentally determined values, as evidenced by a linear correlation (Figure S61). A linear trend was also observed in the Exner‐plot^[59]^ (Figure S47),^[60]^ indicating that the AIPs investigated here follow a similar inversion mechanism for the thermal *Z*‐to*‐E* isomerization at 20 °C.^[60]^

**Figure 5:**
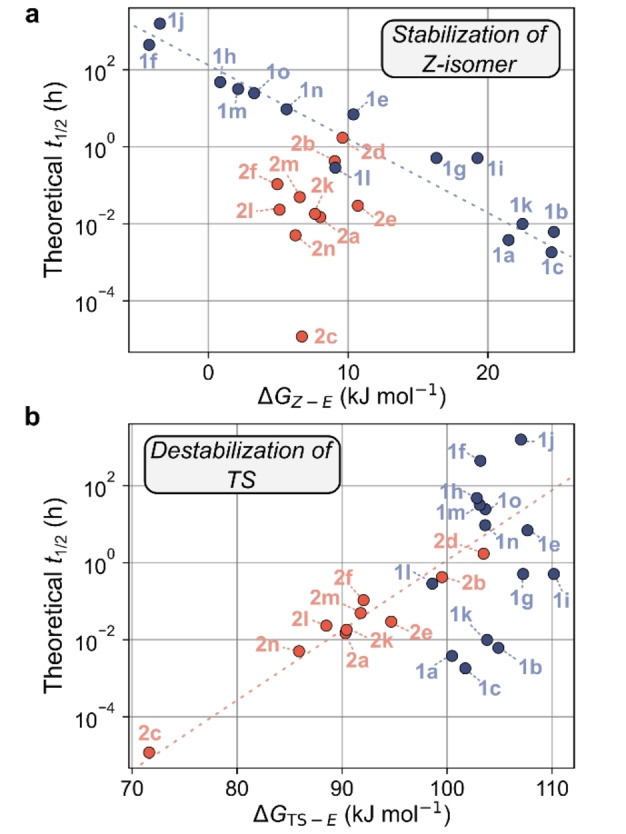
**a**, Plot of the logarithm of the theoretically determined *t*_1/2_ at 20 °C against the difference in the calculated free energy of the *Z*‐ and *E*‐isomers. **b**, Plot of the logarithm of the theoretically determined *t*_1/2_ at 20 °C against the difference in the calculated free energy of the TS and *E*‐isomer. Data points for the *N*‐pyrazoles are colored blue, and those for the *N*‐phenyls are red. Dotted lines are provided to guide the eye.

Plots of theoretical *t*_1/2_ versus Δ*G*_*Z–E*_ and Δ*G*_TS*–E*_ were constructed to gauge the energies of the *Z*‐isomers and TS structures relative to the energy of the *E*‐isomer. For *N*‐pyrazole AIPs, a linear correlation was observed between the theoretical *t*_1/2_ and Δ*G*_*Z–E*_, where a smaller Δ*G*_*Z–E*_ correlates with a longer *t*_1/2_ (Figure 5a). However, *N*‐phenyl analogs showed no discernible trend in this regard. In contrast, the plot of theoretical *t*_1/2_ versus Δ*G*_TS*–E*_ revealed a linear correlation for *N*‐phenyl AIPs, where a larger Δ*G*_TS*–E*_ correlates with a longer *t*_1/2_, while *N*‐pyrazole analogs lacked a trend (Figure 5b).

Taken together, the results indicate that changes in *t*_1/2_ for the *N*‐pyrazoles are dominated by the stability of the *Z*‐isomers rather than the TS energy. In contrast, for the *N*‐phenyl AIPs, changes in *t*_1/2_ are primarily influenced by the TS energy. The electronics of the functionalized phenyl ring more strongly affect the TS stability of the *N*‐phenyl AIPs. This behavior is consistent with the *N*‐phenyl AIPs exhibiting a perpendicular TS geometry and is supported by geometry‐optimized TS structures (Section 6 of Supporting Information). The TS stability of the *N*‐pyrazole derivatives is less impacted by the functionalization of the phenyl ring due to their preferred planar geometry. Instead, the *t*_1/2_ of the *N*‐pyrazoles is primarily determined by the energy of the *Z*‐isomer.

### The Influence of Para‐Substitution on Photoswitching Properties

Functionalizing the *para*‐position of the phenyl modulates the system's electronic properties but is too remote to interact with the imine or pyrazole ring sterically. For the *N*‐pyrazole AIP substituted with a *para*‐methoxy group, **1 b**, there was no significant change in photoswitching properties; the energy of the electronic absorption and *t*_1/2_ remained relatively unchanged. In contrast, *N*‐phenyl analog **2 b** exhibited a 19 nm bathochromic shift in the *E*‐isomer's absorption spectrum relative to **2 a**. Moreover, the *t*_1/2_ of **2 b** increased 12‐fold compared to **2 a**, achieving 12.2 mins at 20 °C. This extended *t*_1/2_ is attributed to a destabilization of the TS: the Δ*G* ^≠^ is ca. 8 kJ mol^−1^ larger for **2 b** compared to **2 a**. This TS destabilization is understood by considering the energy of the resonance structures of the perpendicular TS conformation (Figure 4b).^[28]^

**Figure 6:**
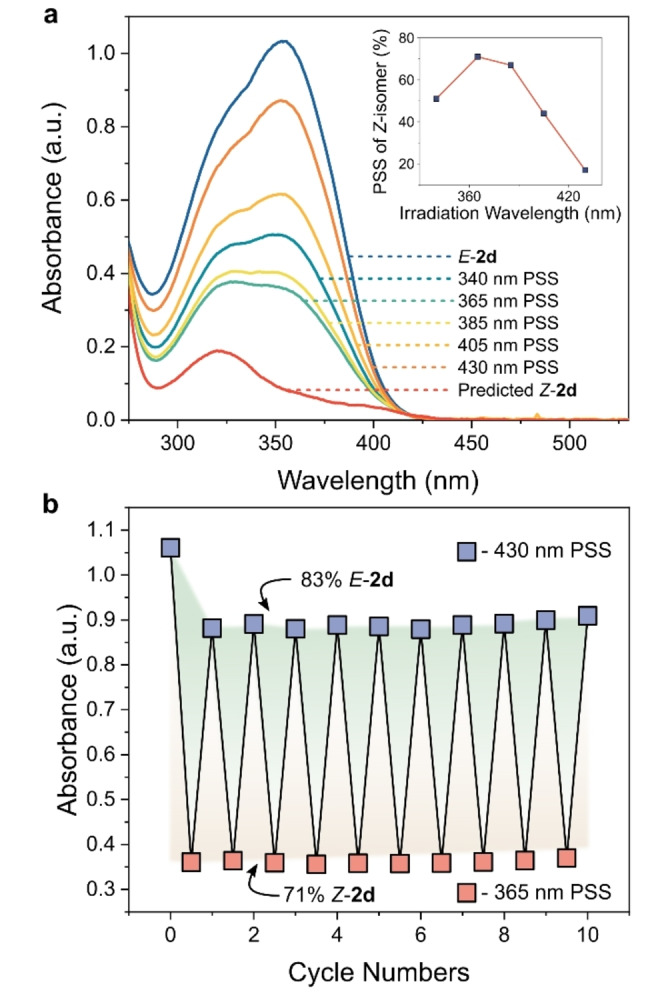
**a**, UV/Vis absorption spectra of **2 d** as the *E*‐isomer, at the PSS under different irradiation conditions, and the predicted *Z*‐isomer. *(Inset)* Plot of the percentage of *Z*‐isomer at the PSS as a function of irradiation wavelength. **b**, Plot showing the fatigue resistance towards bidirectional photoswitching between a *Z*‐rich PSS (71 % *Z*‐isomer, 365 nm irradiation) and an *E*‐rich PSS (83 % *E*‐isomer, 430 nm irradiation).

Increasing the strength of the EDG by using *para–*NMe_2_, yielding **2 d**, further destabilizes the resonance structures of the *N*‐phenyl's TS, affording a *t*_1/2_ of 12.9 mins at 20 °C. This is the longest *t*_1/2_ of the *N*‐phenyl AIPs reported so far. This derivative also exhibits a 53 nm bathochromic shift compared to its unfunctionalized analog **2 a**, attributed to a low‐energy π_n_–π* transition originating from the lone pair on the *para*‐NMe_2_.^[61]^ This enabled **2 d** to achieve *E*‐to*‐Z* photoisomerism with visible light (Figure 6a). In addition, **2 d**, severing as an exemplar *N*‐phenyl AIP, displayed no signs of fatigue after 10 photoswitching cycles (Figure 6b).

Alternatively, the *N*‐phenyl's TS could be stabilized by introducing a *para* −NO_2_ group, yielding **2 c**, with a *t*_1/2_ of less than 0.1 s at 20 °C. A relatively minor 3‐fold decrease in *t*_1/2_ was observed for the *para*‐NO_2_ *N*‐pyrazole analog **1 c**, attributed to the lesser impact of this functionalization on the TS stability.

In summary, *para*‐functionalization has a more significant impact on the *t*_1/2_ of the *N*‐phenyl derivatives by modulating the energy of the TS. This arises from delocalizing the imine nitrogen's lone pair into the phenyl ring, which is only possible when the TS adopts a perpendicular conformation. Interestingly, unlike their azo counterparts,[^46^, ^62^] *para*‐functionalization with EDGs results in a longer *t*_1/2_ due to the absence of lone pair repulsions for the imines′ *Z*‐isomer. This behavior highlights another example of how the structure–property relationships between these two classes of photoswitch differ.

### Moving Closer to the Imine Bond: The Impact of Ortho‐Amination

*Ortho*‐substituting the *N*‐phenyl AIP with pyrrolidine, **2 e**, enacted changes to the photophysical properties similar to the *N*‐pyrazoles. Notably, there was a significant bathochromic shift in the UV/Vis absorption spectrum: 71 nm for the *E*‐isomer relative to **2 a**. This shift, comparable to that seen in the *N*‐pyrazole derivatives, indicates a similar impact of the EDG group on the photoswitch's electronic properties, specifically a low‐energy π_n_–π* transition arising from the *ortho*‐amine substituents,^[61]^ and displaying the useful property of negative photochromism.[^29^, ^61^] The optimized structure of *Z*‐**2 e** reveals a departure from the T‐shape geometry to a twisted conformation (74°), while the TS remains a protracted T‐shape (Figure S80).

While the di‐*ortho*‐pyrrolidine *N*‐pyrazole AIP, *Z*‐**1 f**, displayed a *t*_1/2_ of 19.2 hours at 20 °C,^[29]^ the *t*_1/2_ of *Z*‐**2 f** significantly diminished to 20 s. Analyzing the TS of **2 f** reveals a distorted planar arrangement (Figure S81b). This is the only *N*‐phenyl AIP in this study that shows a deviation from the preferred perpendicular TS and is attributed to increased electron density from the pyrrolidine substituents.^[28]^ The perpendicular TS is electronically destabilized to such an extent that the system adopts an intermediate conformation, supporting previous observations by Haag, Saalfrank, and co‐workers.^[28]^ In doing so, delocalization of the *sp*‐hybridized nitrogen into the electron‐rich phenyl ring is reduced.

For the *Z*‐isomer, less steric congestion between the imine's CH and the di‐*ortho*‐pyrrolidine is observed for the *N*‐phenyl AIPs, supported by the observations made for the crystal structures of the two regioisomers **1 m** and **2 m** (Figure 2a, b). Thus, the steric clash imparted by the *ortho*‐pyrrolidine units on *Z*‐**2 f** was insufficient to force the imine out‐of‐plane, as was observed for *Z*‐**1 f**. *Z*‐**2 f**, therefore, exhibits a greater distance between the C−H of the pyrazole and the centroid of the phenyl ring (2.67 Å for *Z*‐**2 f** vs 2.34 Å of *Z*‐**1 f**), resulting in a weaker CH‐π interaction. This change in TS conformation and weaker CH‐π interactions accounts for the differences in the *t*_1/2_.

### Does the Choice of Ortho‐Amine Substituent Matter?

**Figure 7:**
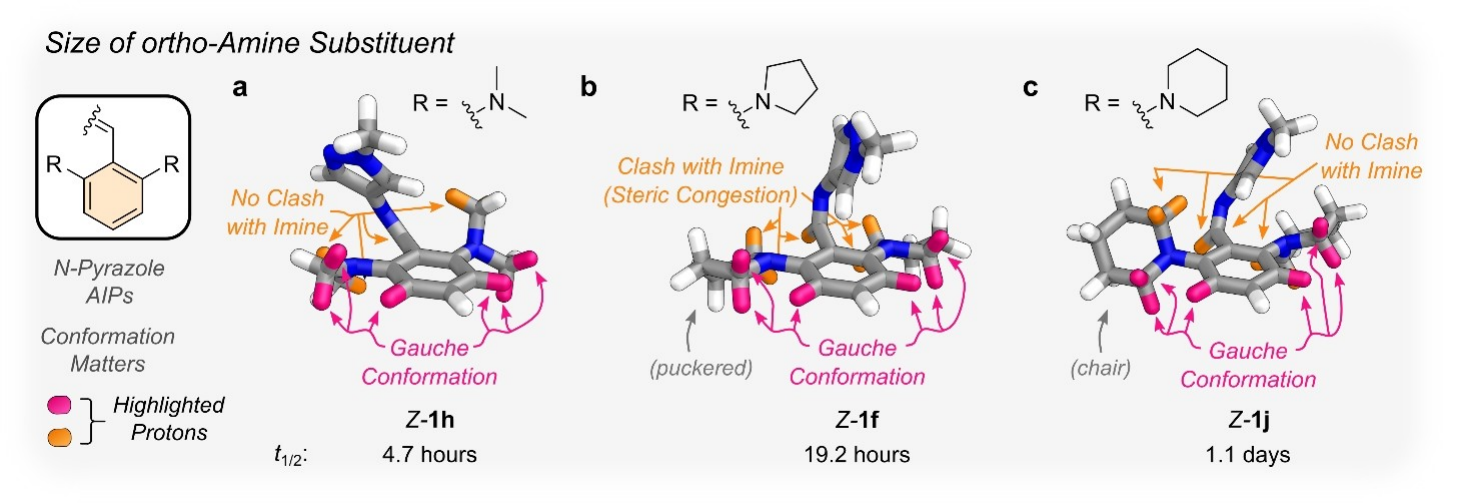
Overview of the influence of the size of the *ortho*‐amine substituents (**a**–**c**) on the DFT‐optimized structures and the *t*_1/2_. Important interactions between protons are highlighted in pink and orange.

The stability of the *Z*‐isomers for the *ortho*‐aminated *N*‐pyrazole derivatives prompted us to study the effect of other amine substituents (Figure 7). We prepared *N*‐pyrazoles with −NMe_2_ at one or both *ortho*‐positions, **1 g**, and **1 h**, respectively. The UV/Vis absorption spectra of both mono‐ and di‐substituted *E*‐isomers were hypsochromically shifted compared to their *ortho*‐pyrrolidine counterparts while retaining negative photochromism.[^29^, ^61^] The *t*_1/2_ was also significantly shorter for the −NMe_2_ derivatives, attributed to weaker dispersive interactions for the *Z*‐isomers, evidenced by a smaller NCI surface (Figure S67 and S68), and a greater Δ*G*_*Z–E*_ (Table S9). This reduced dispersion interaction in *Z*‐**1 g** is due to the pyrazole ring being orientated away from the *ortho*‐NMe_2_. More importantly, in *Z*‐**1 h**, the steric hindrance imparted by the flanking −NMe_2_ motifs is insufficient to enforce a T‐shape geometry (Figure 7a), resulting in a twisted conformation with weaker dispersive interactions and, thus, a shorter *t*_1/2_ than **1 f**.

We prepared *N*‐pyrazoles *ortho*‐functionalized with piperidine to investigate a larger ring size and greater steric hindrance around the imine. However, the conformation of the ring also emerged as an important parameter, specifically the chair conformation of piperidine vs. the puckered conformation of pyrrolidine (Figure 7b, c). Incorporating a single piperidine at the *ortho*‐position of the phenyl ring, **1 i**, showed a shorter *t*_1/2_ compared to its *ortho*‐pyrrolidine counterpart, **1 e**. On the other hand, installing piperidines at both *ortho*‐positions of the phenyl ring, **1 j**, afforded a 30 % longer *t*_1/2_ than the pyrrolidine analog **1 f**. This renders *Z*‐**1 j** the most thermally stable AIP reported to date, displaying a *t*_1/2_ of 25.9 hours at 20 °C. This is attributed to a combination of TS destabilization and *Z*‐isomer stabilization. In the TS, the greater hindrance between the piperidine ring and the imine bond distorts the TS away from planarity, increasing its energy.

Interestingly, the geometry of *Z*‐**1 j** is no longer T‐shaped as observed for *Z*‐**1 f** but is now twisted (Figure 7c and 2d). This difference is due to the conformation of the pyrrolidine and piperidine rings. The chair conformation of the piperidine ring provides more conformational freedom to the imine. Thus, the CH of the imine is not forced out‐of‐plane as observed for the puckered pyrrolidine derivative. The result is the pyrazole ring twisting towards the piperidine to maximize dispersive interactions and stabilize the *Z*‐isomer. In the case of the pyrrolidine derivative *Z*‐**1 f**, the destabilization of the *Z*‐isomer caused by the CH of the imine being forced out‐of‐plane is partially offset by the CH‐π interaction.

### Ortho‐Fluorination: Its Impact, or Lack Thereof

**Figure 8:**
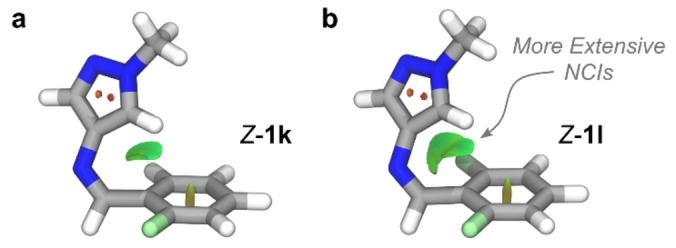
**a**, Optimized structure of *Z*‐**1 k** with NCIs displayed. **b**, Optimized structure of *Z*‐**1 l** showing a larger NCI surface.

Surprisingly, the *ortho*‐fluorination strategy, pioneered by Hecht and co‐workers for azo‐based materials to achieve *t*_1/2_ values ranging from years to decades,[^8^, ^43^, ^63^] did not afford similar enhancements for the AIPs. Only moderate changes were observed in the UV/Vis absorption spectra for the mono‐ and di‐*ortho*‐fluorinated *N*‐pyrazole AIPs (Table S3). Regarding the thermal stability of the *Z*‐isomers, the *t*_1/2_ of mono‐fluorinated AIP **1 k** was 3.5 times longer than **1 a**, while *ortho*‐fluorination of both sites in **1 l** resulted in an over 30‐fold increase in *t*_1/2_ (6.8 mins at 20 °C). Unlike *Z*‐**1 a**, geometry‐optimized *Z*‐isomers show that the CH of the pyrazole ring orients towards the phenyl ring for both **1 k** and **1 l** (Figure 8). This orientation promotes more extensive dispersive interactions (Table S9).

Compared to the *N*‐pyrazole derivatives, *ortho*‐fluorination of the *N*‐phenyl AIPs showed no significant differences in the electronic absorption properties or the thermal stability of the *Z*‐isomer. The T‐shape conformation of the *Z*‐isomers remained intact.

The difference in the impact of *ortho*‐fluorination on the photoswitching properties of azo‐based switches compared to the AIPs highlights their distinct structure–property relationships. This deviation is attributed to the absence of lone pair repulsions in the photochromic bond.^[29]^ In azo‐based switches, the σ‐electron withdrawing fluorine atoms reduce electron repulsion in the HOMO of the *Z*‐isomer.^[8]^ However, the absence of adjacent lone pairs in the imine bond prevents such repulsions; thus, no significant stabilization of the meta‐stable state is observed. An alternative explanation for the increased *t*_1/2_ of *ortho*‐fluorinated azo‐based switches involves electrostatic stabilization in the *Z*‐isomer between the electronegative fluorine atoms and the electropositive hydrogen atoms on the pyrazole ring.^[45]^ Given that such an interaction is also present in the case of the imines, we propose that this is not a significant contributor relative to the lone pair repulsion explanation in azo‐based photoswitches.

### Unexpected Properties from Combining Functionalization Strategies

Combining different functionalization patterns led to new photoswitching behaviors. Notably, merging *ortho*‐amination with *ortho*‐fluorination of the *N*‐pyrazoles, resulting in **1 m**, significantly extended the *t*_1/2_ compared to the mono‐aminated or mono‐fluorinated derivatives. Specifically, the *t*_1/2_ of **1 m** was substantially increased to 5.5 h at 20 °C. This increase is attributed to greater stabilization of the *Z*‐isomer, indicated by a lower Δ*G*_Z–*E*_ value compared to **1 e** and **1 k** (Table S9). Similar results were observed when replacing pyrrolidine with piperidine, as in **1 n**.

While incorporating a *para*‐methoxy group did not substantially improve the photoswitching properties of the *N*‐pyrazole AIPs, it provides a convenient means to integrate these photoswitches into various other scaffolds.[^63^, ^64^] With this in mind, we designed **1 o**, incorporating a *para*‐methoxy unit while demonstrating useful photoswitching properties: high *E*‐to‐*Z* PSS with visible light and a *t*_1/2_ of 4.9 hours at 20 °C. Thus, **1 o** exemplifies a straightforward approach to designing AIPs with handles for further functionalization while maintaining photoswitching performance. For *N*‐phenyl AIPs, combinations of functionalization approaches investigated here did not yield any notable improvements in photoswitching properties.

### Design Rules for Tuning Photoswitching Properties

The first set of design rules for tuning the photoswitching properties of the AIPs can now be drawn (Table 2 and S9):

1. The UV/Vis absorption spectrum of the *E*‐isomers for both the *N*‐pyrazole and *N*‐phenyl AIPs can be bathochromically shifted into the visible light spectral region by amination of the phenyl ring, resulting in a low‐energy π_n_–π* transition. The largest redshift was observed for *ortho*‐pyrrolidine derivatives.
2. Negative photochromism can be achieved by amination of the phenyl ring for both regioisomers.[^29^, ^61^]
3. The *t*_1/2_ of the *N*‐pyrazoles is best tuned by modulating the dispersive interactions between the pyrazole ring and *ortho*‐groups in the *Z*‐isomer.
4. The *t*_1/2_ of the *N*‐phenyl derivatives is best modulated by varying the electronics of the phenyl ring, which significantly influences the stability of the TS.
5. Absence of lone pair repulsions in the *Z*‐isomer of the imine bond, compared to azo‐based switches, allows the inclusion of EDGs at the *ortho‐* and *para*‐positions without compromising the *t*_1/2_. Conversely, *ortho*‐fluorination does not significantly enhance the *t*_1/2_ as is observed in azo‐based photoswitches.
6. Choice of *ortho*‐amine substituent matters. For the *N*‐pyrazoles, the optimal ring size for achieving the longest *t*_1/2_ of *ortho*‐amination at a single site is the 5‐membered pyrrolidine ring. When both *ortho*‐positions are aminated, larger piperidine rings are preferred for a longer *t*_1/2_.
7. Combinations of substitution strategies can result in photoswitching properties beyond the sum of their isolated modifications.

**Figure 9:**
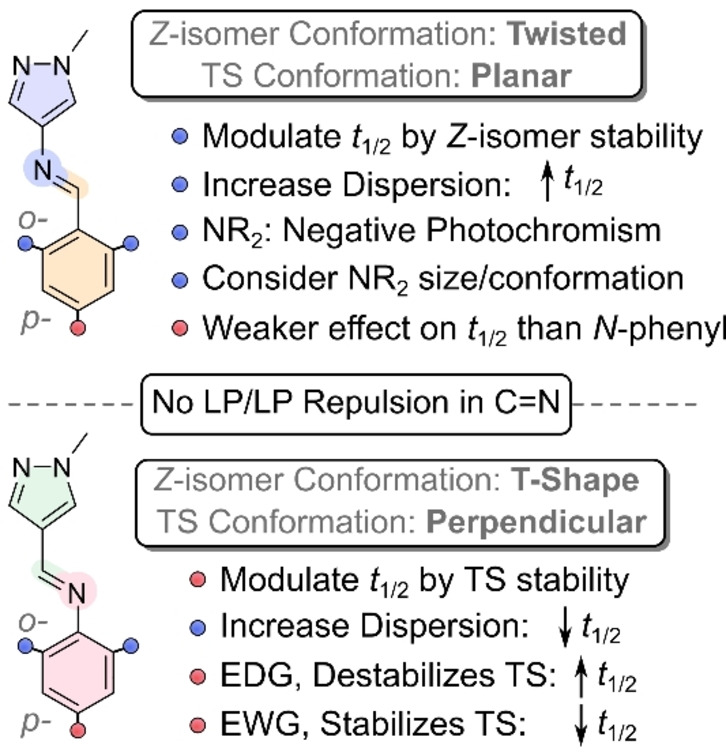
Overview of the key design rules.

When deciding between an *N*‐pyrazole and an *N*‐phenyl AIP, the choice depends on the specific application, with *t*_1/2_ being a critical determining factor. The optimal *t*_1/2_ varies depending on the targeted application: an ultrafast *t*_1/2_ is necessary for information transfer, while hours to days are preferable for supramolecular chemistry and energy storage, and a *t*_1/2_ exceeding years is needed for data storage.^[15]^ The wider variation in photoswitching properties of the *N*‐pyrazole AIPs generally makes them an attractive choice. However, if *ortho*‐substitution is impractical due to steric requirements and a relatively short *t*_1/2_ suffices, *N*‐phenyls are a more promising strategy. Combinations of substitution patterns can be used to fine‐tune the photoswitching properties. Additionally, the *N*‐pyrazoles and *N*‐phenyl AIPs have shown that they can support a *para*‐ether linkage without detriment to their inherent photoswitching properties, facilitating their incorporation into larger scaffolds.[^46^, ^62^, ^63^, ^64^] A diagrammatic summary of these design rules is shown in Figure 9.

Finally, action plots indicate that the optimum wavelength to induce *E*‐to‐*Z* isomerism is redshifted compared to the maxima of the *E*‐isomers absorption.[^65^, ^66^, ^67^] The causes of this redshift are currently being discussed in the literature.^[66]^

### Photoswitching in the Condensed Phase

**Figure 10:**
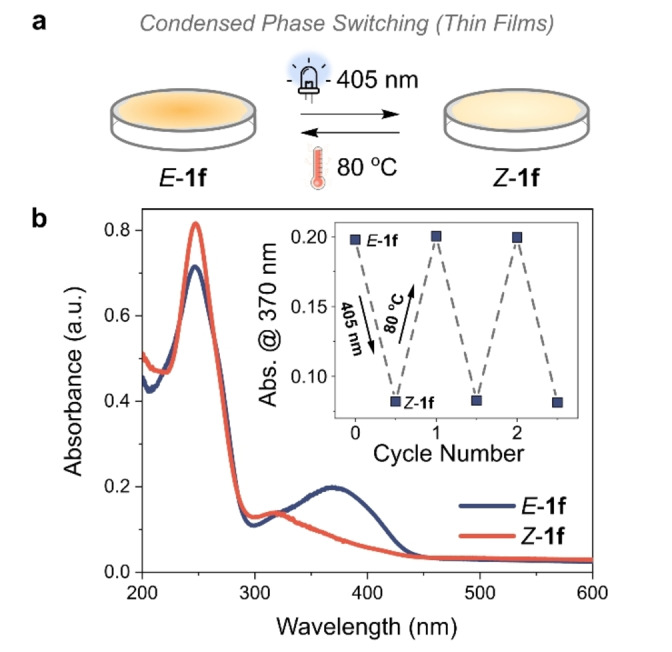
**a**, Schematic representation of the condensed phase measurements, where a thin film of **1 f** is deposited onto a quartz substrate by spin‐coating. **b**, UV/Vis spectra of the thin film in the *E*‐ and *Z*‐ state, measured in transmission mode with an integrating sphere; (*inset*) Plot of absorbance at 370 nm after sequential photoirradiation and heating.

Photoswitching of the AIPs is also possible in the condensed phase (Figure 10a). Translating photoswitching properties from the solution state to the condensed phase is required for many applications,[^68^, ^69^] such as smart, responsive coatings and molecular solar‐thermal (MOST) materials.[^15^, ^18^, ^62^, ^70^] However, photoswitching in the condensed phase is often challenging due to limited light penetration depths and restricted molecular motions.^[18]^ Barner‐Kowollik and co‐workers recently demonstrated that α‐bisimines could efficiently photoisomerize in the solid state by incorporating them into a polymeric architecture to provide conformational flexibility.^[71]^ They could achieve up to 70 % photoisomerism compared to that measured in solution.^[71]^ As an alternative approach to theirs, we postulated that the deviation in planarity observed in the crystal structures of the *E*‐AIPs would result in a less tightly packed structure, facilitating isomerism under photoirradiation.

Thin films of the two AIPs with the longest *t*_1/2_ (*E*‐**1 f** and *E*‐**1 j**) were deposited on quartz substrates via spin coating. These films exhibited photoisomerism in the condensed phase, reaching a total composition of 90 % and 70 % of *Z*‐isomer at the 405 nm PSS for **1 f** and **1 j**, respectively (95 % for **1 f** and 83 % for **1 j** of that achieved in solution, Figure 10b). The *E*‐isomer could be recovered by heating the substrate at 80 °C for 30 mins or *Z*‐to‐*E* photoswitching using 340 nm. This cycle could be repeated (Figure 10b (*inset*), Figure S57, S58). Interestingly, the *t*_1/2_ was shorter in these films (*t*_1/2_ of 5.8 hours for **1 f** and 4.2 hours for **1 j** at 20 °C, Figure S59, S60) compared to the solution state (*t*_1/2_ of 19.2 hours for **1 f** and 25.9 hours for **1 j** at 20 °C in MeCN). This is attributed to the molecular packing; single crystals of *Z*‐**1 j** were stable for over one week, significantly longer than that measured in the solution‐processed thin film. These initial results in condensed phase switching are promising for a further comprehensive study.

## Conclusions

We systematically studied how the functionalization of the photochromic core influences the photoswitching properties of the AIPs, focusing on both *N*‐pyrazole and, for the first time, *N*‐phenyl derivatives. We assessed the effects of *ortho*‐ and *para*‐ functionalization on these compounds′ conformation and photoswitching properties. *N*‐pyrazoles tend to adopt a twisted conformation unless forced into a T‐shape geometry by steric clash, whereas *N*‐phenyl AIPs inherently adopt a T‐shape structure. Differences were also observed in their TSs: *N*‐pyrazole AIPs adopted a planar TS, while the *N*‐phenyl AIPs assumed a perpendicular TS, whose stability is more dependent on the electronics of the phenyl ring.

*Ortho‐*functionalization of the *N*‐pyrazoles enables modulation of the *t*_1/2_ through dispersive interactions, while similar modifications to the *N*‐phenyl derivatives decrease the thermal stability of the *Z*‐isomer. Interestingly, the two regioisomers of the AIPs exhibit different mechanisms underlying their *t*_1/2_ values: the *N*‐pyrazoles are tuned by the varied stability of the *Z*‐isomer. In contrast, the *N*‐phenyls are tuned primarily based on the energy of the TS.

The insights of this study lay the foundation for further engineering AIPs with customized photoswitching properties. Specifically, we are interested in further exploring the light‐controlled dynamic‐covalent chemistry of these systems and determining how properties such as the geometry and stability of the metastable state impact the behavior of a dynamic‐covalent chemical system under light irradiation.

## Supporting Information

Synthetic details and characterization data (PDF)

X‐ray Crystal Structures CCDC Number 2376976‐2376980.

## Conflict of Interests

The authors declare no conflict of interest.

1

## Supporting information

As a service to our authors and readers, this journal provides supporting information supplied by the authors. Such materials are peer reviewed and may be re‐organized for online delivery, but are not copy‐edited or typeset. Technical support issues arising from supporting information (other than missing files) should be addressed to the authors.

Supporting Information

## Data Availability

The data that support the findings of this study are available from the corresponding author upon reasonable request.
