# Supplementary material for: Switching Sides: Regiochemistry and Functionalization Dictate the Photoswitching Properties of Imines
Source: Angew Chem Int Ed Engl. 2024 Nov 2;64(3):e202415464. doi: 10.1002/anie.202415464 (PMC11735892; doi:10.1002/anie.202415464)
Supplement: Supplementary file 1 — Supporting Information [file ANIE-64-e202415464-s001.pdf]

Angewandte Chemie
Eine Zeitschrift der Gesellschaft Deutscher Chemiker

Supporting Information

Switching Sides: Regiochemistry and Functionalization Dictate the Photoswitching Properties of Imines

*J. Wu, L. Kreimendahl, J. L. Greenfield**

Supporting Information:

Switching Sides: Regiochemistry and Functionalization Dictate the Photoswitching Properties of Imines

Jiarong Wu,[a,b] Lasse Kreimendahl[a] and Jake L. Greenfield[a,b]*

[a] *Institut für Organische Chemie, Universität Würzburg, 97074 Würzburg, Germany*

[b] *Center for Nanosystems Chemistry (CNC), Universität Würzburg, 97074 Würzburg, Germany*

* Corresponding Author (Jake.Greenfield@uni-wuerzburg.de)

Table of Contents

1 Materials and Methods 3

2 X-ray Crystallography 6

3 Synthesis and Characterisation 7

4 Photoswitching Properties 39

4.1 Extinction Coefficient 40

4.2 Thermal Dynamic Properties 45

4.3 Photo-Stationary States (PSS) 68

4.4 Quantum Yields 75

4.5 Action Plots 77

5 Thin-film Studies 82

6 Computational Studies 86

7 References 114

1 Materials and Methods

All reagents and solvents were purchased from commercial suppliers and used without further purification unless specified. Solvents were dried with a commercial solvent purification system PS-M6-6/7-En from Inert Technologies. All synthesised photoswitches were stored under N_2, kept dark, and dried under high vacuum for at least 24 h prior to use.

UV/Vis Absorption. Spectra were recorded on a Jasco V-770 spectrophotometer, equipped with a Peltier cooling system (PAC-743R), or a diode array setup as detailed below in Section 1.2.1. Standard 10 mm pathlength quartz glass cuvettes (Fluorescence Cuvettes) from Hellma were used and a stirring bead was added. Measurements were run at 293.15 K, unless otherwise stated. Solutions for the UV-Vis measurements were made using spectroscopy grade dry degassed solvents. A background measurement containing only the solvent used was recorded before measuring samples. This background was subtracted from the sample data using Origin Software.

Absorption Spectroscopy of Thin Films. UV-Vis-NIR absorption spectra were recorded using a PerkinElmer Lambda 950 spectrophotometer equipped with a 150 mm integration sphere. The initial light beam was depolarized using the spectrometer's internal common beam depolarizer and optionally polarized using an integrated mechanically-controlled polarizer. The blank substrate was used for background measurement.

Spin-coating. The process was performed using 10 mg mL−1 solutions of imines in DCM (anhydrous grade, Sigma Aldrich) under ambient conditions. A solution volume of 200 µL was applied using a static dispense method at 1000 rpm for 30 s.

High-Resolution Mass Spectrometry (HR-MS). High-resolution mass spectra (ESI) were recorded on a Bruker Daltonics microTOF focus instrument with a resolution of 18.000 FWHM. Samples were prepared in MeCN and the instrument was run in positive mode.

NMR Spectroscopy. NMR spectra were recorded at 298 K using a Bruker Avance HD III 400 MHz spectrometer automatically tuned and matched to the correct operating frequencies. The ^{13}C NMR spectra are broad-band proton decoupled. TopSpin 4.2 and MestReNova 14.3.2 were used to apply phase and baseline corrections. ^{1}H and ^{13}C NMR spectra were referenced to the residual solvent peak, and the ^{19}F NMR spectra of organic molecules were referenced to hexafluorobenzene at –164.9 ppm. Signals are reported in terms of chemical shift (ppm) and coupling constants (Hz). Abbreviations for multiplicity are as follows: s, singlet; d, doublet; t, triplet; m, multiplet; br, broad; hept, heptet.

Quantum Yield of Photoswitching. The photon flux from the 340, 365, 385, 405, 430 nm LEDs were determined and reported in the previous work from our group.[1] The $\mathbf{\Phi}_{ZE}$ and $\mathbf{\Phi}_{EZ}$

photoisomerization for the aryl imine photoswitches were determined using previously reported literature procedure and the calculation was performed using their provided software.[2] Collimated light was used when determining the photon flux, $\mathbf{\Phi}_{ZE}$, and $\mathbf{\Phi}_{EZ}$.

Diode Array Setup. The diode array setup schematically shown in Figure S1 was employed for Photostationary State (PSS), thermal half-life and quantum yield measurements. The setup consists of an Ocean Insight DH-2000-FHS-DUV-TTL light source (190-2500 nm), connected to a Quantum Northwest QPOD sample holder by 25 nm long premium fibre optics (Ocean Insight QP400-025-SR-BX). The temperature of the QPOD sample holder was controlled with a Quantum Northwest TC 125 temperature controller, which also controlled the stirring. An Ocean Insight Flame-S-XR1-ES diode array spectrometer was used to measure the absorbance spectrum of the sample. Pin-hole slits were employed, as shown in the schematic, along with an optical filter at the light source to reduce the probe light intensity (Thermo Oriel 50550). LEDs of various wavelengths (see Table S1 below) are employed to induce photoisomerization. These LEDs were fitted with adjustable collimation adapters supplied by ThorLabs (either an SM1U with an LA4052-UV Fused Silica Plano Convex Lens with an anti-reflective coating 245-400 nm or an SM1U25-A with an anti-reflective coating 350-700 nm, depending on the wavelength of the LED used). The LEDs were operated at a constant current mode controlled by a ThorLabs DC2200 LED driver.

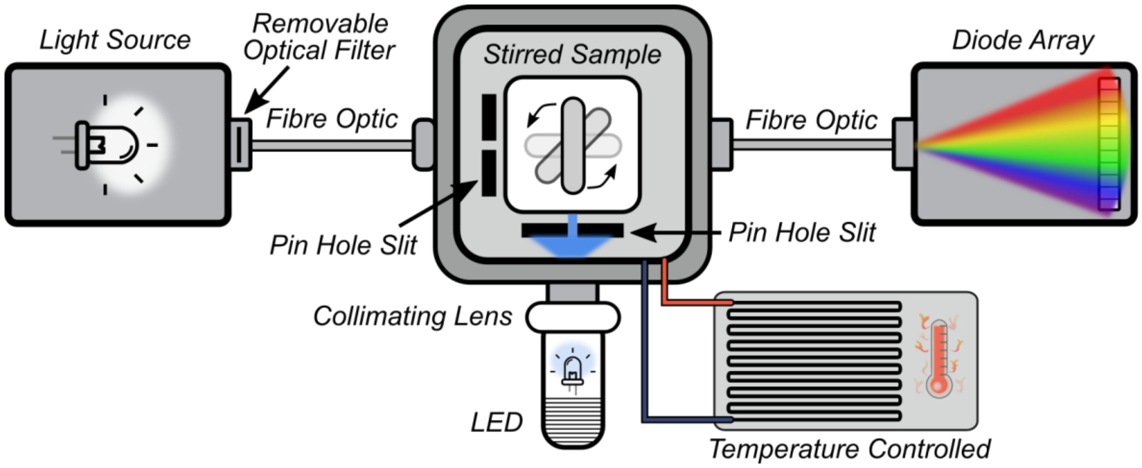

Figure S1. Schematic representation of the diode array setup up to measure the UV-vis absorption spectra and to perform photoswitching studies.

Table S1. Table displaying the models of ThorLabs mounted LEDs used in this study, the nominal emission wavelength (the wavelength in which emission appears brightest to the human eye), and the bandwidth (full width at half maximum, FWHM).

LED model number (ThorLabs)	Wavelength (nm)	Bandwidth/FWHM (nm)
M340L5	340	10
M365L3	365	9
M385L3	385	11
M405L4	405	13
M430L5	430	17
M470L5	470	28
M505L4	505	37
M590L4	590	15

2 X-ray Crystallography

The collection of crystallographic data of the aryl imine photoswitches was obtained by using a Bruker D8 Quest diffractometer with a Photon II CMOS detector and Cu Kα radiation (λ = 1.54178 Å). A solution of the structure was performed with direct methods, refinement with the SHELX software package and expanding using Fourier techniques.[3] Nonhydrogen atoms were refined anisotropically while hydrogen atoms were positioned onto idealized positions and included in calculations of structure factors. CCDC 2376976−2376980 contain the supplementary crystallographic data for this paper. These data can be obtained free of charge from The Cambridge Crystallographic Data Centre via www.ccdc.cam.ac.uk/data_request/cif.

Table S2. X-ray structure characteristics for AIP *E*-**1j**, *Z*-**1j**, *E*-**1m**, and *E*-**2m**.

		E-**1j**	*Z*-**1j**	*E*-**1m**	*E*-**2m**	*E*-**1o**
	Empirical Formula	$C_{21}H_{29}N_5$	$C_{21}H_{29}N_5$	$C_{15}H_{17}FN_4$	$C_{15}H_{17}FN_4$	$C_{16}H_{19}FN_4O$
	$M_{empirical}$ (g mol^{-1})	351.49	351.49	272.32	272.32	302.35
	Wavelength (Å)	1.54178	1.54178	1.54178	1.54178	1.54178
	T (K)	100(2)	100(2)	100(2)	100(2)	100(2)
Crystal Description	Color	Colourless	Colourless	Colourless	Colourless	Colourless
	Habit	Needle	Needle	Plate	Needle	Plate
	Crystal System	Monoclinic	Orthorhombic	Monoclinic	Monoclinic	Orthorhombic
	Space Group	P 2(1)/n	C 222(1)	P 2(1)/c	P 2(1)/c	Pbca
Unit Cell Dimensions	a (Å)	15.634(2)	10.9312(16)	7.8630(8)	5.7554(9)	9.150(5)
	b (Å)	6.7638(11)	19.560(4)	13.4349(16)	16.414(2)	8.793(3)
	c (Å)	19.231(3)	17.729(2)	13.1263(12)	14.480(2)	37.315(15)
	α (°)	90	90	90	90	90
	β (°)	104.809(10)	90	104.051(4)	94.436(7)	90
	γ (°)	90	90	90	90	90
	Volume (Å^3)	1966.1(5)	3790.7(10)	1345.2(2)	1363.8(3)	3002(2)
	Z	4	8	4	4	8
	$\rho_{calc.}$ (g cm^{-3})	1.187	1.232	1.345	1.326	1.338
	F(000)	760	1520	576	576	1280
	Range of θ (°)	3.263 72.314	4.521 72.094	4.785 72.240	4.078 72.318	2.368 72.010
	Goodness of Fit	1.034	1.053	1.046	1.050	1.041
	CCDC	2376977	2376978	2376979	2376976	2376980

3 Synthesis and Characterisation

The precursors 2-(pyrrolidin-1-yl)benzaldehyde, 2,6-di(pyrrolidin-1-yl)benzaldehyde, and imines **1a**, **1e**, and **1f** are synthesized based on previously published work from our group.[1]

(*E*)-1-(4-methoxyphenyl)-*N*-(1-methyl-1*H*-pyrazol-4-yl)methanimine, 1b

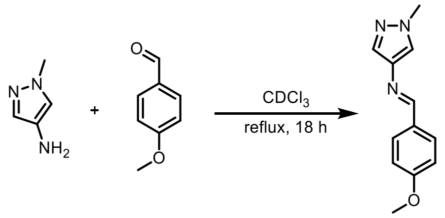

In oven-dried glassware charged with 3 Å molecular sieves, 4-amino-1-methylpyrazole (107.0 mg, 1.1 mmol, 1.5 eq) and 2-fluoro-4-methoxybenzaldehyde (100.0 mg, 0.7 mmol, 1.0 eq) were dissolved in $CDCl_3$ (2.0 mL) under N_2. The mixture was heated to reflux for 18 hours under N_2, and reaction completion was determined by TLC. The solvent was then removed in vacuo. The solid was purified via flash-chromatography using EtOAc with 1% Triethylamine to afford product **1b** as white solid (132.0 mg, 88% yield). ^1H NMR (400 MHz, 298 K, CD_3CN) δ 8.56 (s, 1H), 7.79 – 7.75 (m, 2H), 7.59 (dd, *J* = 15.4, 0.8 Hz, 2H), 7.03 – 6.98 (m, 2H), 3.84 (s, 3H), 3.83 (s, 3H); ^{13}C NMR (101 MHz, 298 K, CD_3CN) δ 162.78, 157.63, 137.13, 131.92, 130.77, 130.46, 124.79, 115.12, 56.10, 39.61; HR ESI-MS (MeCN) for $[C_{12}H_{13}N_3O+H]^+$: m/z calcd: 216.1131; found: 216.1130; −0.5 ppm error.

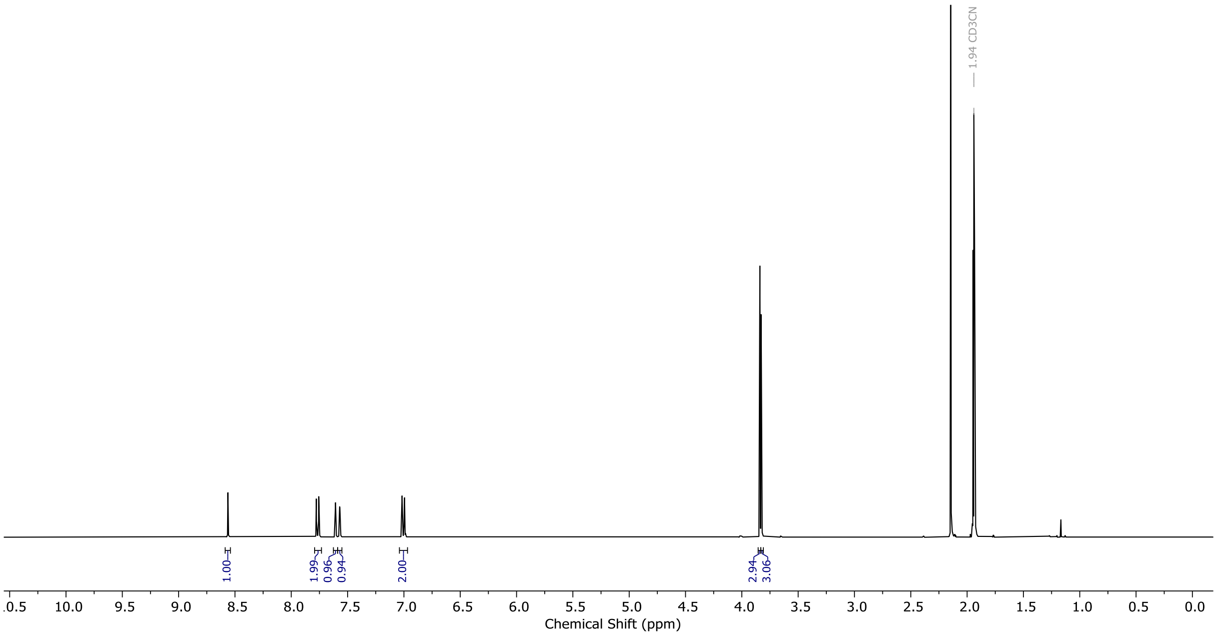

Figure S2. ^1H NMR (400 MHz, 298 K, CD_3CN) of **1b**.

(*E*)-*N*-(1-methyl-1*H*-pyrazol-4-yl)-1-(4-nitrophenyl)methanimine, 1c

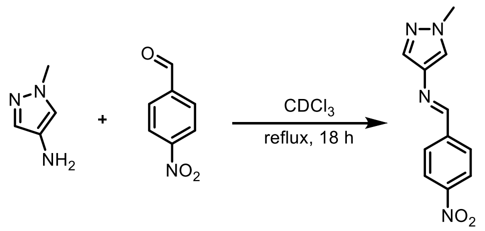

In oven-dried glassware charged with 3 Å molecular sieves, 4-amino-1-methylpyrazole (65.3 mg, 0.7 mmol, 1.5 eq) and 4-nitrobenzaldehyde (75.6 mg, 0.5 mmol, 1.0 eq) were dissolved in $CDCl_3$ (2.0 mL) under N_2. The mixture was heated to reflux for 18 hours under N_2, and reaction completion was determined by TLC. The solvent was then removed in vacuo. The solid was purified via flash-chromatography using EtOAc with 1% Triethylamine to afford product **1c** as yellow solid (106.1 mg, 92% yield). ^{1}H NMR (400 MHz, 298 K, CD_3CN) δ 8.75 (s, 1H), 8.29 (m, 2H), 8.02 (m, 2H), 7.75 (d, *J* = 0.7 Hz, 1H), 7.69 (d, *J* = 0.8 Hz, 1H), 3.86 (s, 3H); ^{13}C NMR (101 MHz, 298 K, CD_3CN) δ 156.50, 143.21, 132.28, 130.54, 129.38, 126.39, 124.89, 39.71; HR ESI-MS (MeCN) for $[C_{11}H_{10}N_4O_2+Na]^+$: m/z calcd: 253.0696; found: 253.0708; 4.7 ppm error.

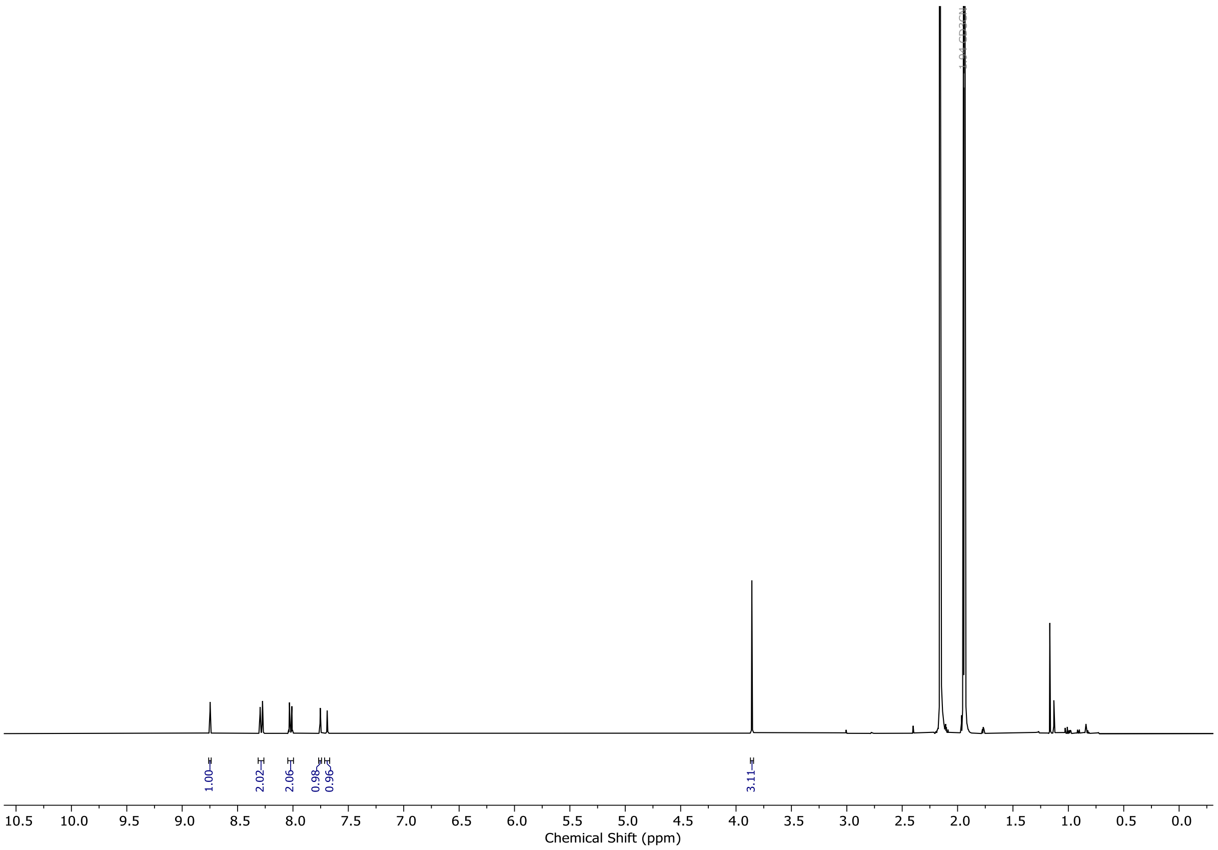

Figure S3. ^{1}H NMR (400 MHz, 298 K, CD_3CN) of **1c**.

(*E*)-*N*,*N*-dimethyl-2-(((1-methyl-1*H*-pyrazol-4-yl)imino)methyl)aniline, 1g

Synthesis of 2-(dimethylamino)benzaldehyde, S1

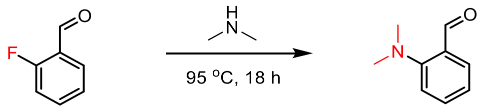

To a flask was added 2-fluorobenzaldehyde (0.56 g, 4.5 mmol, 1 eq) and dimethylamine (40% in water, 12.0 mL, 90.0 mmol, 20 eq). The mixture was then left to stir at 95 °C for 18 h under an N_2 atmosphere. The reaction progress was monitored by TLC. After the complete consumption of 2-fluorobenzaldehyde, 10 mL water was added, and the mixture was extracted with dichloromethane. The resulting yellow solution was dried over $MgSO_4$ and purified by column chromatography on silica gel using an eluent of 9:1 cyclohexane:ethyl acetate. The product **S1** was dried under high vacuum affording a yellow oil (0.42 g, 63% yield). ^{1}H NMR (400 MHz, 298 K, $CDCl_3$) δ 10.22 (d, *J* = 0.6 Hz, 1H), 7.75 (dd, *J* = 7.7, 1.8 Hz, 1H), 7.45 (ddd, *J* = 8.4, 7.2, 1.8 Hz, 1H), 7.07 – 6.94 (m, 2H), 2.91 (s, 6H); ^{13}C NMR (101 MHz, 298 K, $CDCl_3$) δ 191.25, 155.85, 134.67, 131.01, 127.10, 120.68, 117.69, 45.60; HR ESI-MS (MeCN) for $[C_9H_{11}NO+H]^+$: m/z calcd: 150.0913; found: 150.0914; 0.7 ppm error.

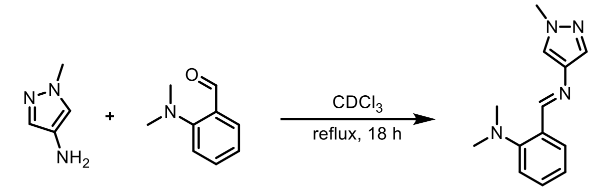

In oven-dried glassware charged with 3 Å molecular sieves, 4-amino-1-methylpyrazole (97.6 mg, 1 mmol, 1.5 eq) and **S1** (100.0 mg, 0.7 mmol, 1.0 eq) were dissolved in $CDCl_3$ (2.0 mL) under N_2. The mixture was heated to reflux for 18 hours under N_2, and reaction completion was determined by TLC. The solvent was then removed in vacuo. The oil was purified via flash-chromatography using EtOAc with 1% Triethylamine to afford product **1g** as yellow oil (119.6 mg, 78% yield). ^{1}H NMR (400 MHz, 298 K, CD_3CN) δ 8.89 (s, 1H), 7.96 – 7.89 (m, 1H), 7.66 (d, *J* = 0.8 Hz, 1H), 7.61 (d, *J* = 0.8 Hz, 1H), 7.43 – 7.34 (m, 1H), 7.15 (dd, *J* = 8.2, 1.2 Hz, 1H), 7.10 – 7.01 (m, 1H), 3.84 (s, 3H), 2.77 (s, 6H); ^{13}C NMR (101 MHz, 298 K, CD_3CN) δ 156.26, 155.35, 137.56, 132.09, 132.02, 130.08, 128.08, 124.91, 123.10, 119.33, 45.77, 39.59; HR ESI-MS (MeCN) for $[C_{13}H_{16}N_4+H]^+$: m/z calcd: 229.1448; found: 229.1452; 1.7 ppm error.

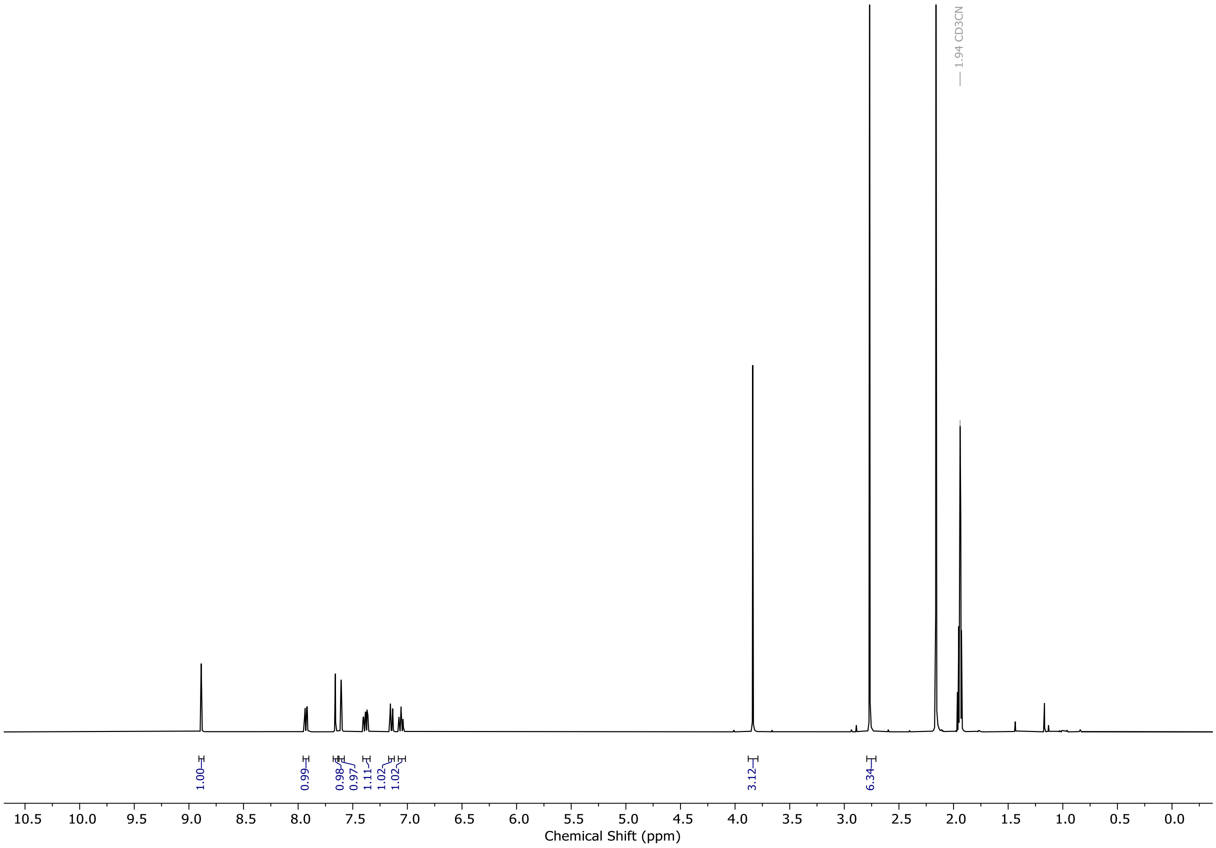

Figure S4. ^{1}H NMR (400 MHz, 298 K, CD_3CN) of **1g**.

(*E*)-*N*[1],*N*[1],*N*[3],*N*[3]-tetramethyl-2-(((1-methyl-1*H*-pyrazol-4-yl)imino)methyl)benzene-1,3-diamine, 1h

Synthesis of 2,6-bis(dimethylamino)benzaldehyde, S2

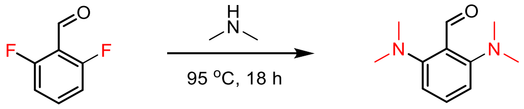

To a flask was added 2,6-difluorobenzaldehyde (0.64 g, 4.5 mmol, 1 eq) and dimethylamine (40% in water, 12.0 mL, 90.0 mmol, 20 eq). The mixture was then left to stir at 95 °C for 18 h under an N_2 atmosphere. The reaction progress was monitored by TLC. After the complete consumption of 2,6-difluorobenzaldehyde, 10 mL water was added, and the mixture was extracted with dichloromethane. The resulting yellow solution was dried over $MgSO_4$ and purified by column chromatography on silica gel using an eluent of 9:1 cyclohexane:ethyl acetate. The product **S2** was dried under high vacuum affording a yellow oil (0.63 g, 73% yield). ^{1}H NMR (400 MHz, 298 K, CD_3CN) δ 9.73 (s, 1H), 7.26 (t, *J* = 8.2 Hz, 1H), 6.42 (d, *J* = 8.2 Hz, 2H), 2.92 (s, 12H); ^{13}C NMR (101 MHz, 298 K, $CDCl_3$) δ 186.88, 157.72, 135.03, 106.66, 44.95, 27.01; HR ESI-MS (MeCN) for $[C_{11}H_{16}N_2O+H]^+$: m/z calcd: 193.1335; found: 193.1333; −1.0 ppm error.

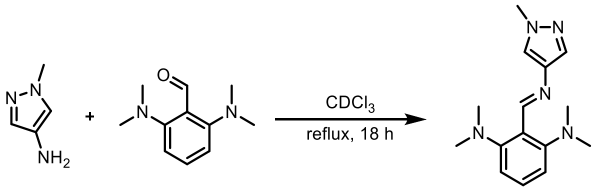

In oven-dried glassware charged with 3 Å molecular sieves, 4-amino-1-methylpyrazole (75.8 mg, 0.8 mmol, 1.5 eq) and **S2** (100.0 mg, 0.5 mmol, 1.0 eq) were dissolved in $CDCl_3$ (2.0 mL) under N_2. The mixture was heated to reflux for 18 hours under N_2, and reaction completion was determined by TLC. The solvent was then removed in vacuo. The oil was purified via flash-chromatography using EtOAc with 1% Triethylamine to afford product **1h** as yellow oil (130.2 mg, 92% yield). ^{1}H NMR (400 MHz, 298 K, CD_3CN) δ 8.89 (s, 1H), 7.96 – 7.89 (m, 1H), 7.66 (d, *J* = 0.8 Hz, 1H), 7.61 (d, *J* = 0.8 Hz, 1H), 7.43 – 7.34 (m, 1H), 7.15 (dd, *J* = 8.2, 1.2 Hz, 1H), 7.10 – 7.01 (m, 1H), 3.84 (s, 3H), 2.77 (s, 6H); ^{13}C NMR (101 MHz, 298 K, CD_3CN) δ 157.81, 156.11, 138.96, 131.71, 131.49, 123.95, 121.12, 111.85, 107.55, 45.56, 45.03, 39.54; HR ESI-MS (MeCN) for $[C_{15}H_{21}N_5+H]^+$: m/z calcd: 272.1870; found: 272.1864; −2.2 ppm error.

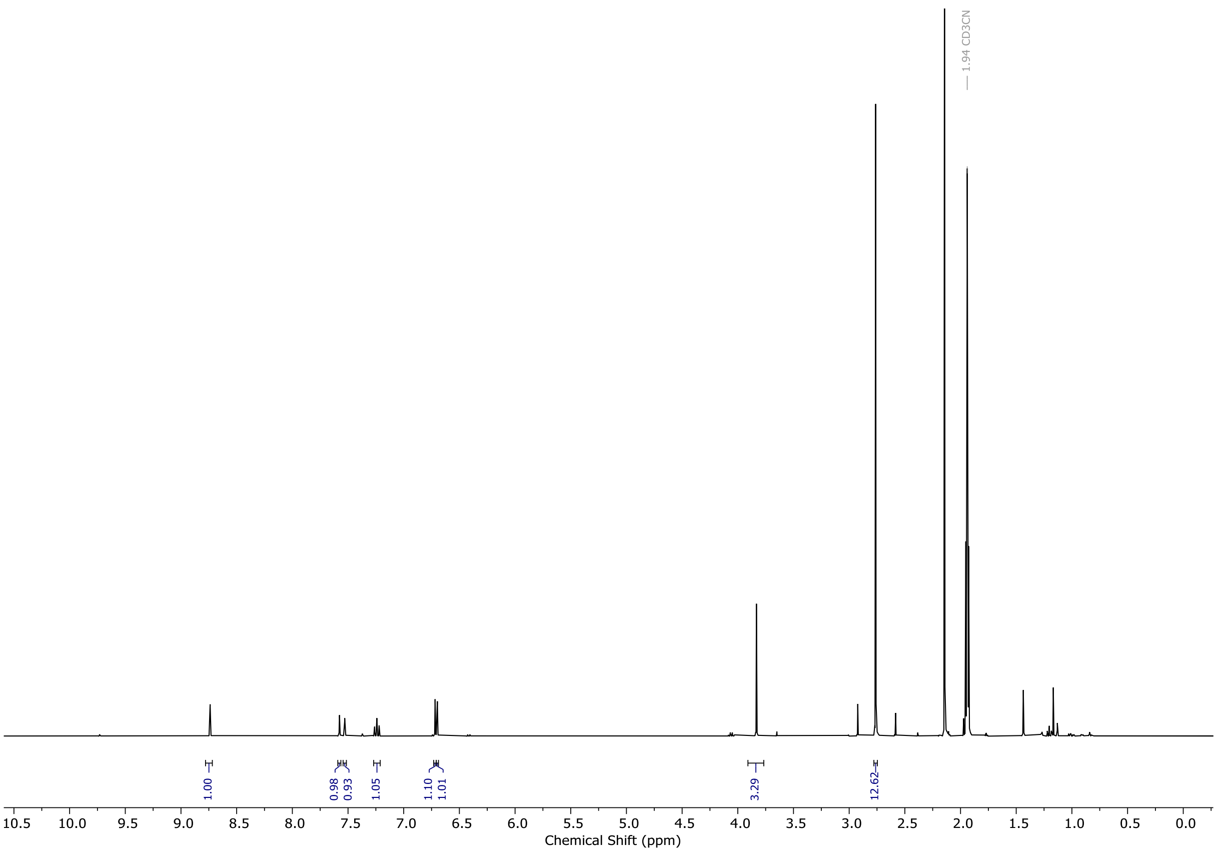

Figure S5. ^1H NMR (400 MHz, 298 K, CD_3CN) of **1h**.

(*E*)-*N*-(1-methyl-1*H*-pyrazol-4-yl)-1-(2-(piperidin-1-yl)phenyl)methanimine, 1i

Synthesis of 2-(piperidin-1-yl)benzaldehyde, S3

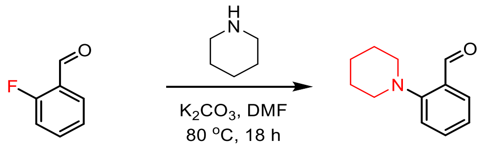

To an oven dried flask was added 2-fluorobenzaldehyde (1.00 g, 8.1 mmol, 1 eq), K_2CO_3 (2.23 g, 16.1 mmol, 2 eq) and 10 mL of anhydrous DMF. To this suspension was added piperidine (1.37 g, 16.1 mmol, 2 eq) and the mixture was then left to stir at 80 °C for 18 h under an N_2 atmosphere. The mixture was then filtered under gravity and the filtrate was concentrated in *vacuo*. The resulting oil was purified by column chromatography on silica gel using an eluent of 9:1 cyclohexane:ethyl acetate. The product **S3** was dried under high vacuum affording a yellow oil (1.26 g, 82% yield). ^{1}H NMR (400 MHz, 298 K, $CDCl_3$) δ 10.30 (d, *J* = 0.8 Hz, 1H), 7.79 (ddd, *J* = 7.7, 1.8, 0.5 Hz, 1H), 7.50 (ddd, *J* = 8.2, 7.2, 1.8 Hz, 1H), 7.13 – 7.02 (m, 2H), 3.09 – 3.01 (m, 4H), 1.82 – 1.71 (m, 4H), 1.66 – 1.55 (m, 2H); ^{13}C NMR (101 MHz, 298 K, $CDCl_3$) δ 191.91, 157.16, 134.96, 129.33, 128.75, 122.10, 119.11, 55.78, 26.34, 24.20; HR ESI-MS (MeCN) for $[C_{12}H_{15}NO+H]^+$: m/z calcd: 190.1226; found: 190.1236; 5.3 ppm error.

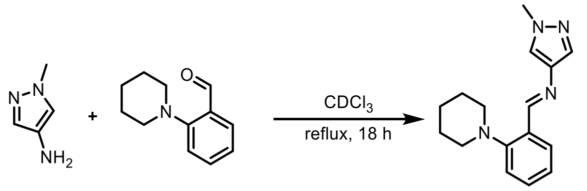

In oven-dried glassware charged with 3 Å molecular sieves, 4-amino-1-methylpyrazole (77.0 mg, 0.8 mmol, 1.5 eq) and **S3** (100.0 mg, 0.5 mmol, 1.0 eq) were dissolved in $CDCl_3$ (2.0 mL) under N_2. The mixture was heated to reflux for 18 hours under N_2, and reaction completion was determined by TLC. The solvent was then removed in vacuo. The oil was purified via flash-chromatography using EtOAc with 1% Triethylamine to afford product **1i** as yellow oil (125.1 mg, 93% yield). ^{1}H NMR (400 MHz, 298 K, CD_3CN) δ 8.91 (s, 1H), 7.94 (dd, *J* = 7.8, 1.7 Hz, 1H), 7.65 (d, *J* = 0.8 Hz, 1H), 7.60 (d, *J* = 0.8 Hz, 1H), 7.39 (ddd, *J* = 8.1, 7.2, 1.7 Hz, 1H), 7.13 (dd, *J* = 8.1, 1.1 Hz, 1H), 7.12 – 7.04 (m, 1H), 3.84 (s, 3H), 2.97 – 2.89 (m, 4H), 1.80 – 1.70 (m, 4H), 1.62 – 1.57 (m, *J* = 6.0 Hz, 2H); ^{13}C NMR (101 MHz, 298 K, CD_3CN) δ 156.02, 155.58, 137.62, 132.21, 131.94, 130.67, 127.76, 124.91, 123.52, 120.01, 55.62, 39.61, 27.06, 24.88; HR ESI-MS (MeCN) for $[C_{16}H_{20}N_4+H]^+$: m/z calcd: 269.1761; found 269.1759; −0.7 ppm error.

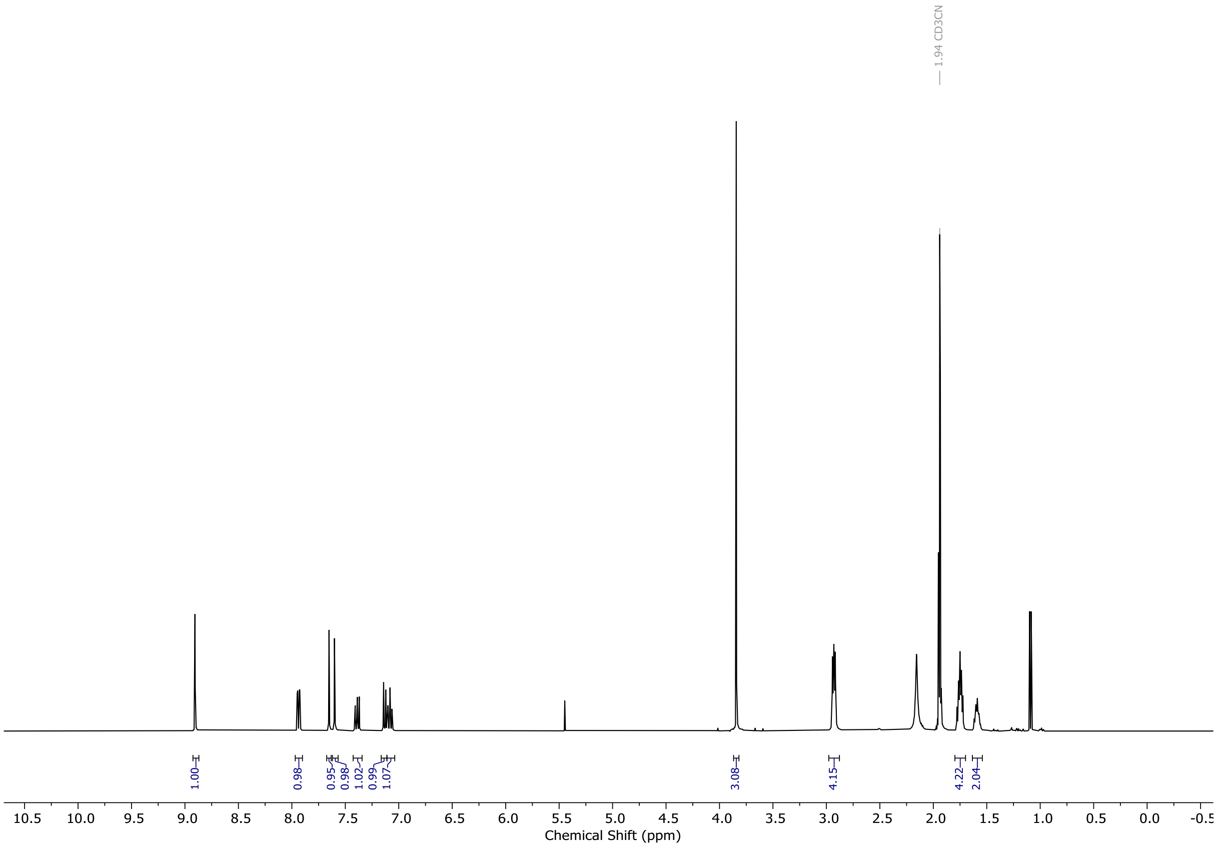

Figure S6. ^1H NMR (400 MHz, 298 K, CD_3CN) of **1i**.

(*E*)-1-(2,6-di(piperidin-1-yl)phenyl)-*N*-(1-methyl-1*H*-pyrazol-4-yl)methanimine, 1j

Synthesis of 2,6-di(piperidin-1-yl)benzaldehyde, S4

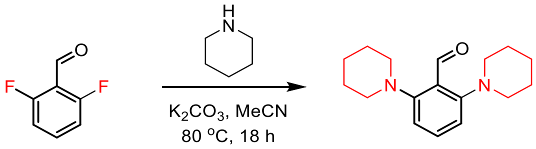

To an oven dried flask was added 2,6-difluorobenzaldehyde (1.00 g, 7.0 mmol, 1 eq), K_2CO_3 (3.89 g, 28.1 mmol, 4 eq) and 10 mL of anhydrous acetonitrile. To this suspension was added piperidine (2.40 g, 28.1 mmol, 4 eq) and the mixture was then left to stir at 80 °C for 18 h under an N_2 atmosphere. The mixture was then filtered under gravity and the filtrate was concentrated in *vacuo*. The resulting oil was purified by column chromatography on silica gel using an eluent of 9:1 cyclohexane:ethyl acetate. The product **S4** was dried under high vacuum affording a yellow solid (1.28 g, 67% yield). ^{1}H NMR (400 MHz, 298 K, $CDCl_3$) δ 9.90 (s, 1H), 7.32 (t, *J* = 8.2 Hz, 1H), 6.53 (d, *J* = 8.2 Hz, 2H), 3.15 – 3.08 (m, 8H), 1.77 (q, *J* = 5.5 Hz, 8H), 1.65 – 1.53 (m, 4H); ^{13}C NMR (101 MHz, 298 K, $CDCl_3$) δ 187.93, 157.60, 135.07, 118.77, 109.59, 55.10, 26.32, 24.44; HR ESI-MS (MeCN) for $[C_{17}H_{24}N_2O+H]^+$: m/z calcd: 273.1961; found: 273.1960; −0.4 ppm error.

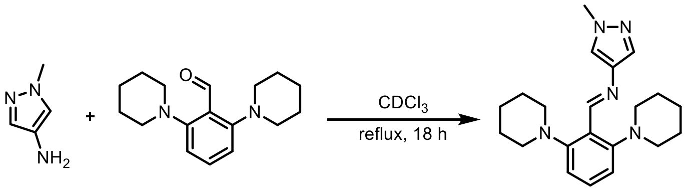

In oven-dried glassware charged with 3 Å molecular sieves, 4-amino-1-methylpyrazole (53.5 mg, 0.55 mmol, 1.5 eq) and **S4** (100.0 mg, 0.4 mmol, 1.0 eq) were dissolved in $CDCl_3$ (2.0 mL) under N_2. The mixture was heated to reflux for 18 hours under N_2, and reaction completion was determined by TLC. The solvent was then removed in vacuo. The solid was purified via flash-chromatography using EtOAc with 1% Triethylamine to afford product **1i** as yellow crystalline solid (108.8 mg, 84% yield). ^{1}H NMR (400 MHz, 298 K, CD_3CN) δ 8.77 (s, 1H), 7.61 (dd, *J* = 17.2, 0.8 Hz, 2H), 7.27 (s, 1H), 6.76 (d, *J* = 8.1 Hz, 2H), 3.84 (s, 3H), 3.00 – 2.93 (m, 8H), 1.69 (dq, *J* = 11.0, 5.2 Hz, 8H), 1.60 – 1.49 (m, 4H); ^{13}C NMR (101 MHz, 298 K, CD_3CN) δ 156.77, 156.26, 131.74, 131.56, 124.29, 113.42, 110.70, 55.58, 54.68, 39.55, 27.06, 26.88, 24.97; HR ESI-MS (MeCN) for $[C_{21}H_{29}N_5+H]^+$: m/z calcd: 352.2496; found 352.2506; 2.8 ppm error.

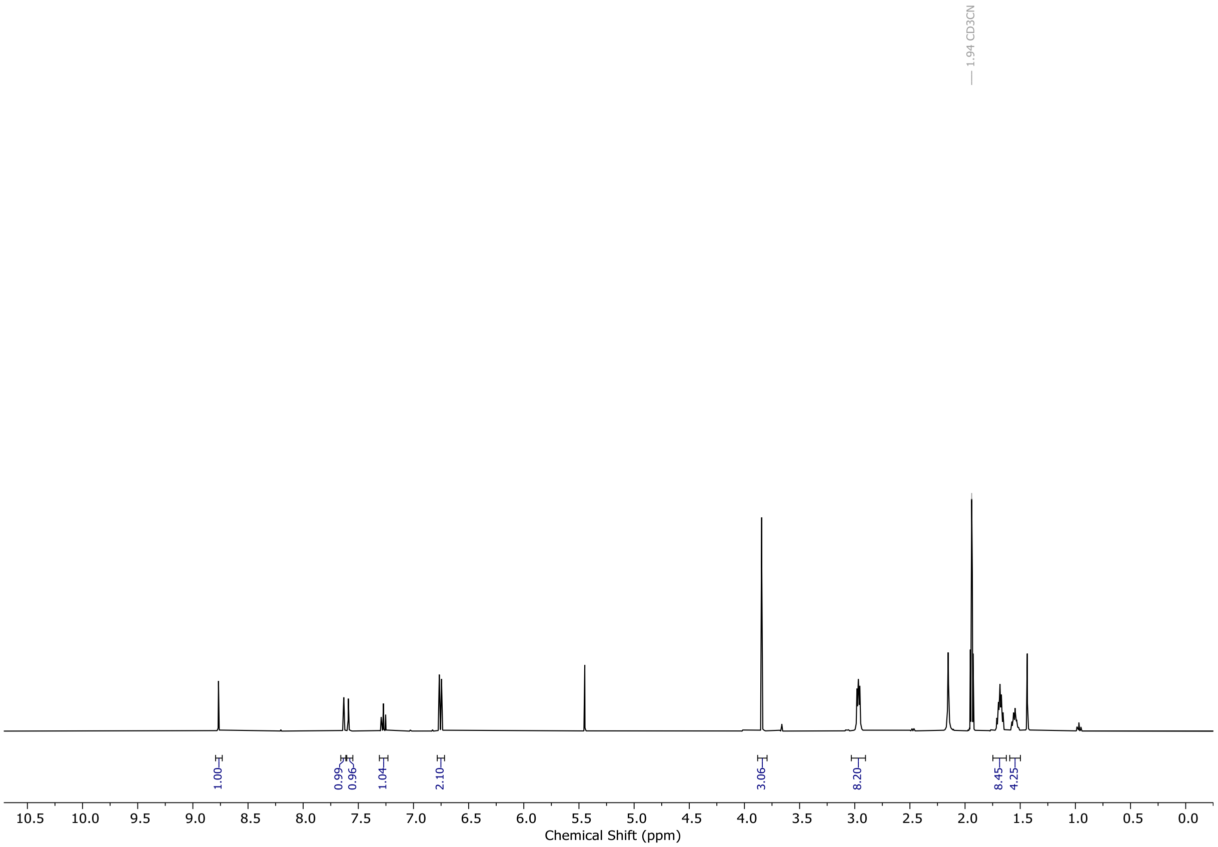

Figure S7. ^{1}H NMR (400 MHz, 298 K, CD_3CN) of **1j**.

(*E*)-1-(2-fluorophenyl)-*N*-(1-methyl-1*H*-pyrazol-4-yl)methanimine, 1k

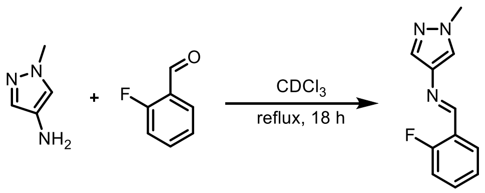

In oven-dried glassware charged with 3 Å molecular sieves, 4-amino-1-methylpyrazole (117.4 mg, 1.2 mmol, 1.5 eq) and 2-Fluorobenzaldehyde (100.0 mg, 0.8 mmol, 1.0 eq) were dissolved in $CDCl_3$ (2.0 mL) under N_2. The mixture was heated to reflux for 18 hours under N_2, and reaction completion was determined by TLC. The solvent was then removed in vacuo. The solid was purified via flash-chromatography using EtOAc with 1% Triethylamine to afford product **1k** as yellow crystalline solid (138.4 mg, 85% yield). ^{1}H NMR (400 MHz, 298 K, CD_3CN) δ 8.87 (s, 1H), 8.05 (td, *J* = 7.6, 1.8 Hz, 1H), 7.70 (d, *J* = 0.8 Hz, 1H), 7.65 (d, *J* = 0.8 Hz, 1H), 7.50 − 7.45 (m, 1H), 7.29 − 7.24 (m, 1H), 7.18 (ddd, *J* = 11.0, 8.3 Hz, 1H), 3.84 (s, 3H); ^{13}C NMR (101 MHz, 298 K, CD_3CN) δ 164.43, 161.93, 150.51, 136.76, 133.35, 132.27, 127.95, 125.60, 125.35, 116.70, 39.67; ^{19}F NMR (376 MHz, 298 K, CD_3CN) δ −125.21. HR ESI-MS (MeCN) for $[C_{11}H_{10}FN_3+H]^+$: m/z calcd: 204.0932; found 204.0933; 0.5 ppm error.

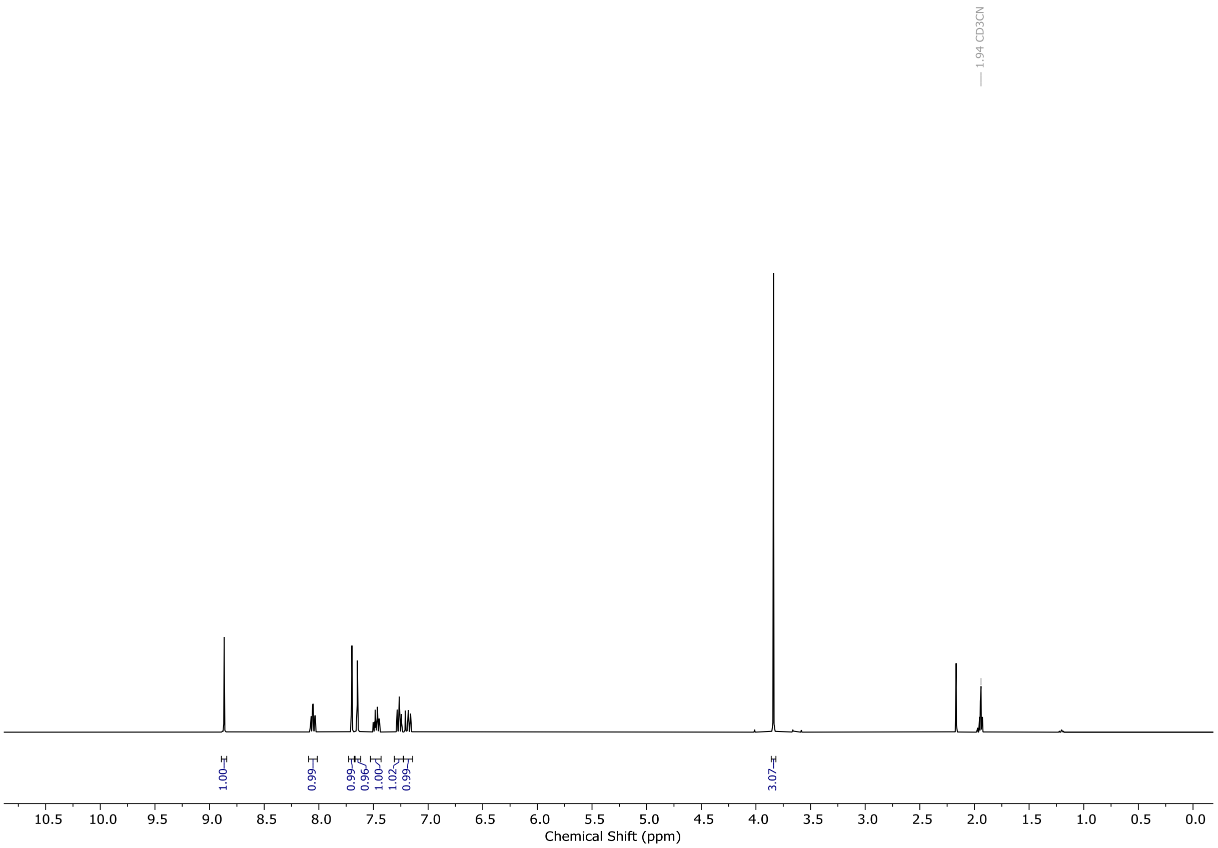

Figure S8. ^{1}H NMR (400 MHz, 298 K, CD_3CN) of **1k**.

(*E*)-1-(2-fluorophenyl)-*N*-(1-methyl-1*H*-pyrazol-4-yl)methanimine, 1l

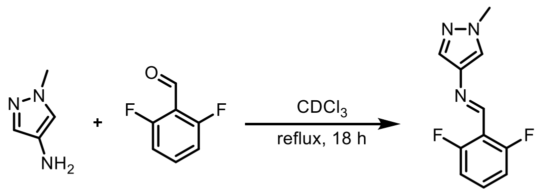

In oven-dried glassware charged with 3 Å molecular sieves, 4-amino-1-methylpyrazole (102.5 mg, 1.1 mmol, 1.5 eq) and 2,6-Difluorobenzaldehyde (100.0 mg, 0.7 mmol, 1.0 eq) were dissolved in $CDCl_3$ (2.0 mL) under N_2. The mixture was heated to reflux for 18 hours under N_2, and reaction completion was determined by TLC. The solvent was then removed in vacuo. The solid was purified via flash-chromatography using EtOAc with 1% Triethylamine to afford product **1l** as yellow solid (146.1 mg, 94% yield). ^{1}H NMR (400 MHz, 298 K, CD_3CN) δ 8.78 (s, 1H), 7.70 (s, 1H), 7.64 (d, *J* = 0.9 Hz, 1H), 7.49 – 7.40 (m, 1H), 7.10 – 6.98 (m, 2H), 3.84 (s, 3H); ^{13}C NMR (101 MHz, 298 K, CD_3CN): δ 163.86, 161.32, 148.38, 137.16, 133.00, 131.98, 125.67, 113.20, 113.00, 77.13, 39.69; ^{19}F NMR (376 MHz, 298 K, CD_3CN) δ -116.84; HR ESI-MS (MeCN) for $[C_{11}H_9F_2N_3+H]^+$: m/z calcd: 222.0837; found 222.0841; 1.6 ppm error.

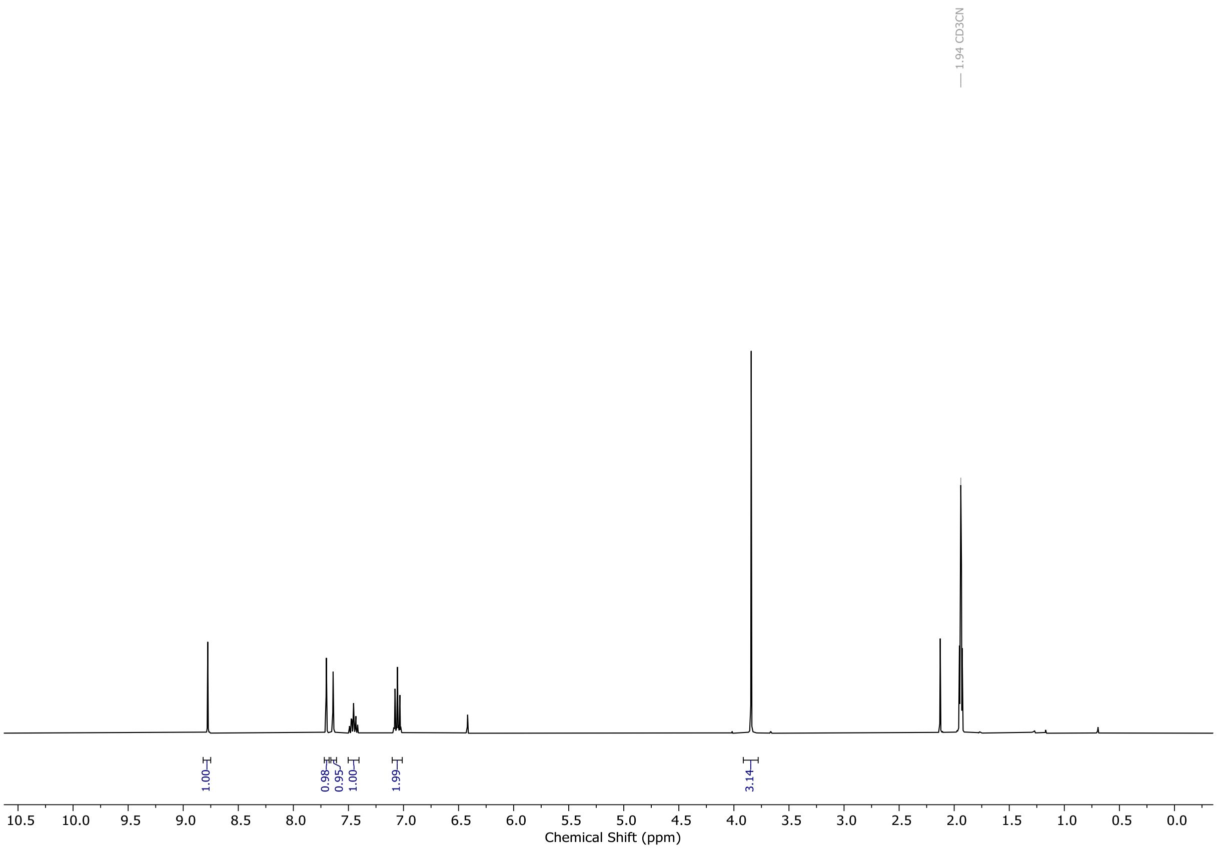

Figure S9. ^{1}H NMR (400 MHz, 298 K, CD_3CN) of **1l**.

(*E*)-1-(2-fluoro-6-(pyrrolidin-1-yl)phenyl)-*N*-(1-methyl-1*H*-pyrazol-4-yl)methanimine, 1m

Synthesis of 2-fluoro-6-(pyrrolidin-1-yl)benzaldehyde, S5

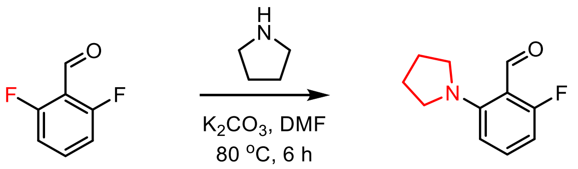

To an oven dried flask was added 2,6-difluorobenzaldehyde (1.10 g, 7.7 mmol, 1 eq), K_2CO_3 (2.14 g, 15.5 mmol, 2 eq) and 10 mL of anhydrous DMF. To this suspension was added pyrrolidine (0.55 g, 7.7 mmol, 1 eq) and the mixture was then left to stir at 80 °C for 6 h under an N_2 atmosphere. The mixture was then filtered under gravity and the filtrate was concentrated in *vacuo*. The resulting oil was purified by column chromatography on silica gel using an eluent of 9:1 cyclohexane:ethyl acetate. The product **S5** was dried under high vacuum affording a waxy yellow solid (1.06 g, 71% yield). ^{1}H NMR (400 MHz, 298 K, $CDCl_3$) δ 10.33 (d, *J* = 0.7 Hz, 1H), 7.31 – 7.25 (m, 1H), 6.60 (dq, *J* = 8.7, 0.9 Hz, 1H), 6.41 (ddd, *J* = 11.4, 8.0, 0.9 Hz, 1H), 3.25 – 3.19 (m, 4H), 1.99 – 1.95 (m, 4H); ^{13}C NMR (101 MHz, 298 K, Chloroform-*d*) δ 186.21, 168.01, 150.06, 134.42, 111.75, 110.36, 102.18, 52.64, 26.01; ^{19}F NMR (376 MHz, 298 K, $CDCl_3$) δ −116.74; HR ESI-MS (MeCN) for $[C_{11}H_{12}OFN+H]^+$: m/z calcd: 194.0976; found: 194.0975; −0.5 ppm error.

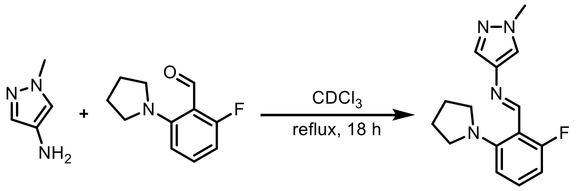

In oven-dried glassware charged with 3 Å molecular sieves, 4-amino-1-methylpyrazole (75.4 mg, 0.8 mmol, 1.5 eq) and **S5** (100.0 mg, 0.5 mmol, 1.0 eq) were dissolved in $CDCl_3$ (2.0 mL) under N_2. The mixture was heated to reflux for 18 hours under N_2, and reaction completion was determined by TLC. The solvent was then removed in vacuo. The solid was purified via flash-chromatography using EtOAc with 1% Triethylamine to afford product **1m** as yellow solid (128.0 mg, 94% yield). ^{1}H NMR (400 MHz, 298 K, CD_3CN) δ 8.78 (s, 1H), 7.62 (d, *J* = 0.7 Hz, 1H), 7.56 (d, *J* = 0.8 Hz, 1H), 7.22 (td, *J* = 8.3, 6.7 Hz, 1H), 6.67 (dt, *J* = 8.5, 1.0 Hz, 1H), 6.51 (ddd, *J* = 10.7, 8.1, 0.9 Hz, 1H), 3.83 (s, 3H), 3.23 – 3.18 (m, 4H), 1.91 – 1.87 (m, 4H); ^{13}C NMR (101 MHz, 298 K, CD_3CN) δ 164.82, 162.37, 153.84, 151.28, 137.49, 131.71, 124.64,

113.00, 111.29, 104.65, 53.24, 51.33, 39.62, 26.38, 26.35; ^{19}F NMR (376 MHz, 298 K, CD_3CN) δ -120.32 ppm; HR ESI-MS (MeCN) for $[C_{15}H_{17}FN_4+H]^+$: m/z calcd: 273.1510; found 273.1518; 2.9 ppm error.

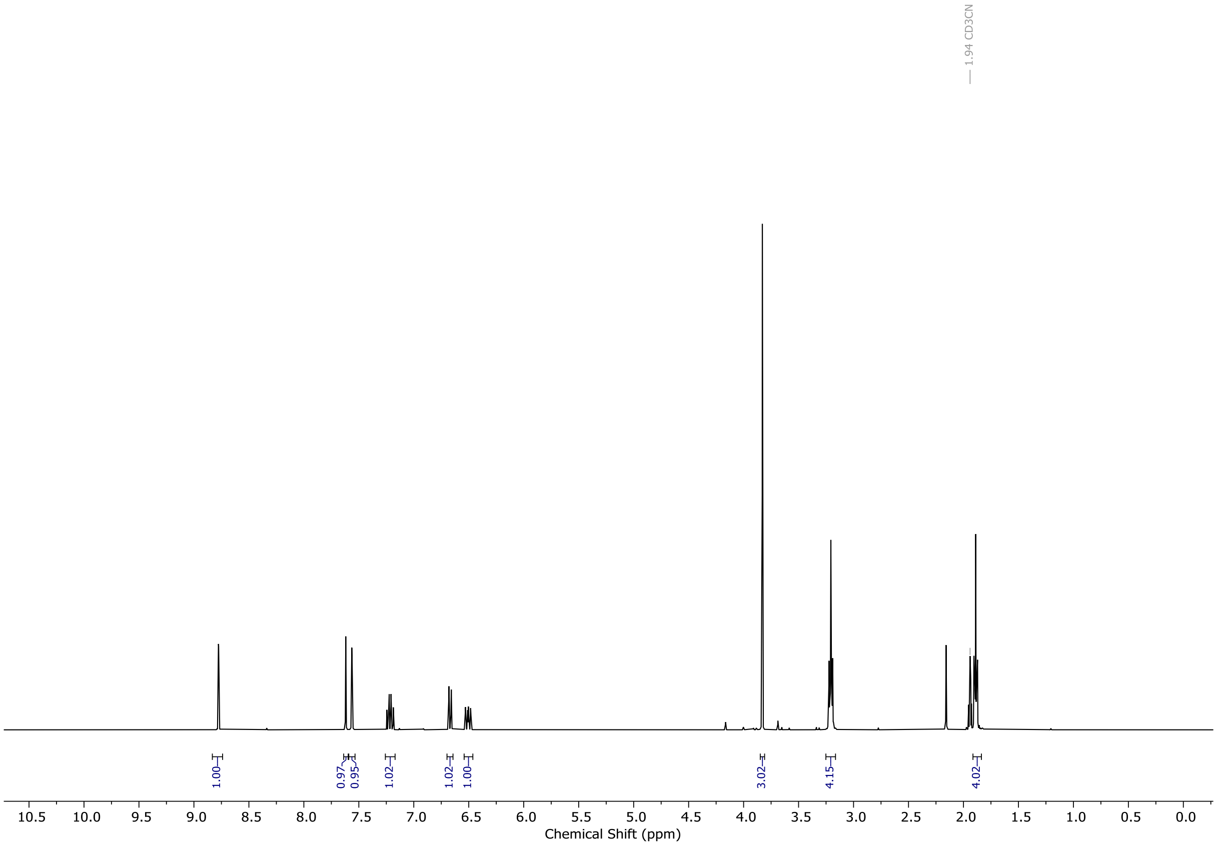

Figure S10. ^1H NMR (400 MHz, 298 K, CD_3CN) of **1m**.

<u>(*E*)-1-(2-fluoro-6-(piperidin-1-yl)phenyl)-*N*-(1-methyl-1*H*-pyrazol-4-yl)methanimine, 1n</u>

<u>Synthesis of 2-fluoro-6-(piperidin-1-yl)benzaldehyde, S6</u>

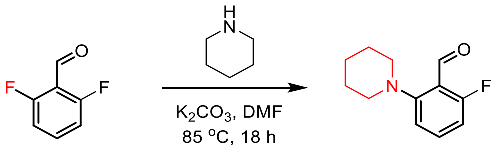

To an oven dried flask was added 2,6-difluorobenzaldehyde (1.11 g, 7.8 mmol, 1 eq), K_2CO_3 (2.15 g, 15.6 mmol, 2 eq) and 10 mL of anhydrous DMF. To this suspension was added piperidine (0.66 g, 7.8 mmol, 1 eq) and the mixture was then left to stir at 85 °C for 18 h under an N_2 atmosphere. The mixture was then filtered under gravity and the filtrate was concentrated in *vacuo*. The resulting oil was purified by column chromatography on silica gel using an eluent of 9:1 cyclohexane:ethyl acetate. The product **S6** was dried under high vacuum affording a pale yellow solid (1.12 g, 69% yield). ^{1}H NMR (400 MHz, 298 K, $CDCl_3$) δ 10.20 (d, *J* = 1.0 Hz, 1H), 7.42 (td, *J* = 8.3, 6.3 Hz, 1H), 6.84 (d, *J* = 8.4 Hz, 1H), 6.72 – 6.66 (m, 1H), 3.07 – 3.03 (m, 4H), 1.79 – 1.72 (m, 4H), 1.64 – 1.57 (m, 2H); ^{13}C NMR (101 MHz, 298 K, Chloroform-*d*) δ 118.16, 164.76, 162.17, 157.24, 135.32, 116.96, 108.91, 55.18, 26.24, 24.04; ^{19}F NMR (376 MHz, 298 K, $CDCl_3$) δ -115.40; HR ESI-MS (MeCN) for $[C_{12}H_{14}OFN+H]^+$: m/z calcd: 208.1132; found: 208.1130; −1.0 ppm error.

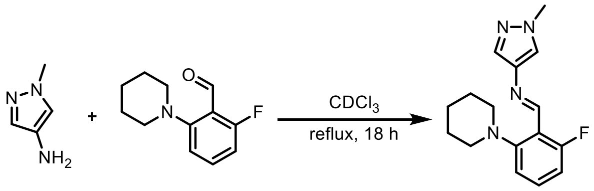

In oven-dried glassware charged with 3 Å molecular sieves, 4-amino-1-methylpyrazole (70.3 mg, 0.7 mmol, 1.5 eq) and **S6** (100.0 mg, 0.5 mmol, 1.0 eq) were dissolved in $CDCl_3$ (2.0 mL) under N_2. The mixture was heated to reflux for 18 hours under N_2, and reaction completion was determined by TLC. The solvent was then removed in vacuo. The solid was purified via flash-chromatography using EtOAc with 1% Triethylamine to afford product **1n** as yellow solid (121.7 mg, 85% yield). ^{1}H NMR (400 MHz, 298 K, CD_3CN) δ 8.68 (s, 1H), 7.66 (d, *J* = 0.8 Hz, 1H), 7.61 (d, *J* = 0.9 Hz, 1H), 7.35 (td, *J* = 8.2, 6.2 Hz, 1H), 6.92 (dt, *J* = 8.2, 0.9 Hz, 1H), 6.81 (ddt, *J* = 10.7, 8.2, 0.8 Hz, 1H), 3.84 (s, 3H), 2.97 – 2.92 (m, 4H), 1.71 (p, *J* = 5.6 Hz, 4H), 1.61 – 1.54 (m, 2H); ^{13}C NMR (101 MHz, 298 K, CD_3CN) δ 163.26, 160.74, 157.00, 153.48, 137.93, 132.40, 131.67, 124.98, 115.58, 15.55, 110.86, 110.64, 55.27, 39.66, 27.01, 24.84; ^{19}F NMR

(376 MHz, 298 K, CD_3CN) δ −115.25; HR ESI-MS (MeCN) for $[C_{16}H_{19}N_4F+H]^+$: m/z calcd: 287.1667; found: 287.1662; −1.7 ppm error.

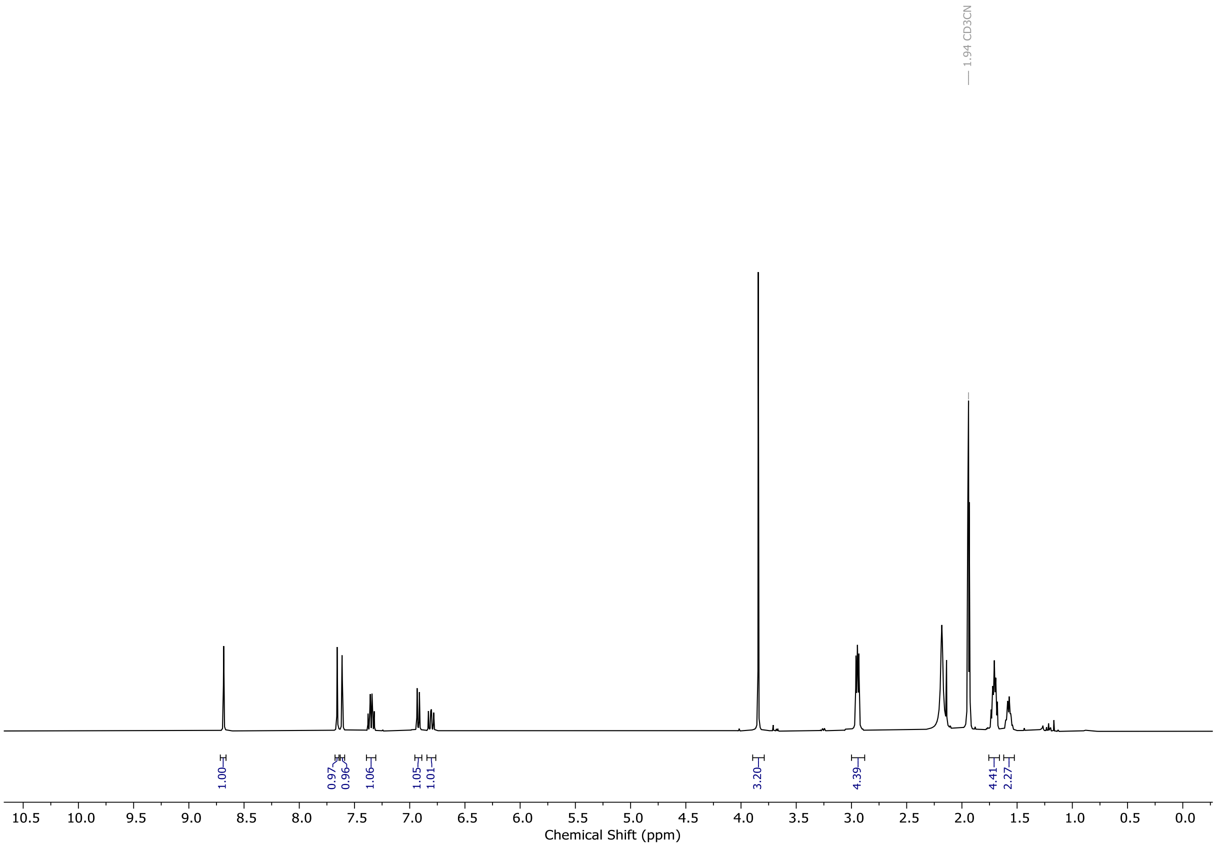

Figure S11. ^1H NMR (400 MHz, 298 K, CD_3CN) of **1n**.

(*E*)-1-(2-fluoro-4-methoxy-6-(pyrrolidin-1-yl)phenyl)-*N*-(1-methyl-1*H*-pyrazol-4-yl)methanimine, 1o

Synthesis of 2-fluoro-4-methoxy-6-(pyrrolidin-1-yl)benzaldehyde, S7

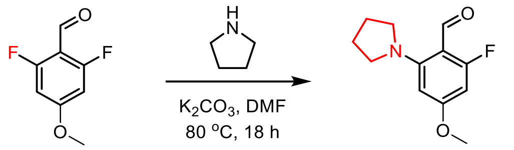

To an oven dried flask was added 2,6-difluoro-4-methoxybenzaldehyde (1.00 g, 5.8 mmol, 1 eq), K_2CO_3 (1.60 g, 11.6 mmol, 2 eq) and 10 mL of anhydrous DMF. To this suspension was added pyrrolidine (0.66 g, 7.8 mmol, 1 eq) and the mixture was then left to stir at 80 °C for 18 h under an N_2 atmosphere. The mixture was then filtered under gravity and the filtrate was concentrated in *vacuo*. The resulting oil was purified by column chromatography on silica gel using an eluent of 9:1 cyclohexane:ethyl acetate. The product **S7** was dried under high vacuum affording a pale yellow solid (0.92 g, 71% yield). ^{1}H NMR (400 MHz, 298 K, $CDCl_3$) δ 10.14 (s, 1H), 6.02 – 5.96 (m, 2H), 3.82 (s, 3H), 3.28 – 3.22 (m, 4H), 2.00 – 1.93 (m, 4H); ^{13}C NMR (101 MHz, 298 K, $CDCl_3$) δ 184.35, 168.56, 164.83, 151.40, 107.00, 94.44, 91.08, 55.64, 52.60, 25.99; ^{19}F NMR (376 MHz, 298 K, $CDCl_3$) δ −117.60; HR ESI-MS (MeCN) for $[C_{12}H_{14}O_2FN+H]^+$: m/z calcd: 224.1081; found: 224.1090; 4.0 ppm error.

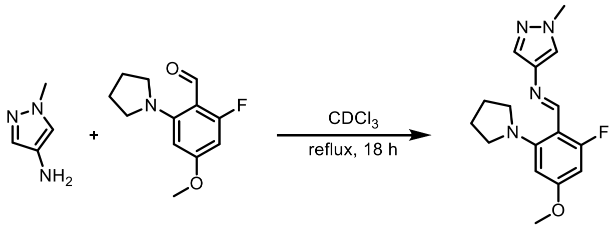

In oven-dried glassware charged with 3 Å molecular sieves, 4-amino-1-methylpyrazole (65.3 mg, 0.7 mmol, 1.5 eq) and **S7** (100.0 mg, 0.5 mmol, 1.0 eq) were dissolved in $CDCl_3$ (2.0 mL) under N_2. The mixture was heated to reflux for 18 hours under N_2, and reaction completion was determined by TLC. The solvent was then removed in vacuo. The solid was purified via flash-chromatography using EtOAc with 1% Triethylamine to afford product **1o** as yellow solid (131.8 mg, 87% yield). ^{1}H NMR (400 MHz, 298 K, CD_3CN) δ 8.69 (s, 1H), 7.57 (s, 1H), 7.52 (s, 1H), 6.18 (d, *J* = 11.3 Hz, 2H), 3.82 (s, 3H), 3.79 (s, 3H), 3.24 – 3.20 (m, 4H), 1.91 – 1.87 (m, 4H); ^{13}C NMR (101 MHz, 298 K, CD_3CN) δ 165.97, 162.85, 153.45, 152.13, 137.90, 131.73, 124.23, 106.72, 96.73, 92.15, 77.12, 56.15, 53.29, 39.59; ^{19}F NMR (376 MHz, 298 K, CD_3CN)

δ −117.90; HR ESI-MS (MeCN) for $[C_{16}H_{19}FN_4O+H]^+$: m/z calcd: 303.1617; found 303.1617; 0.5 ppm error.

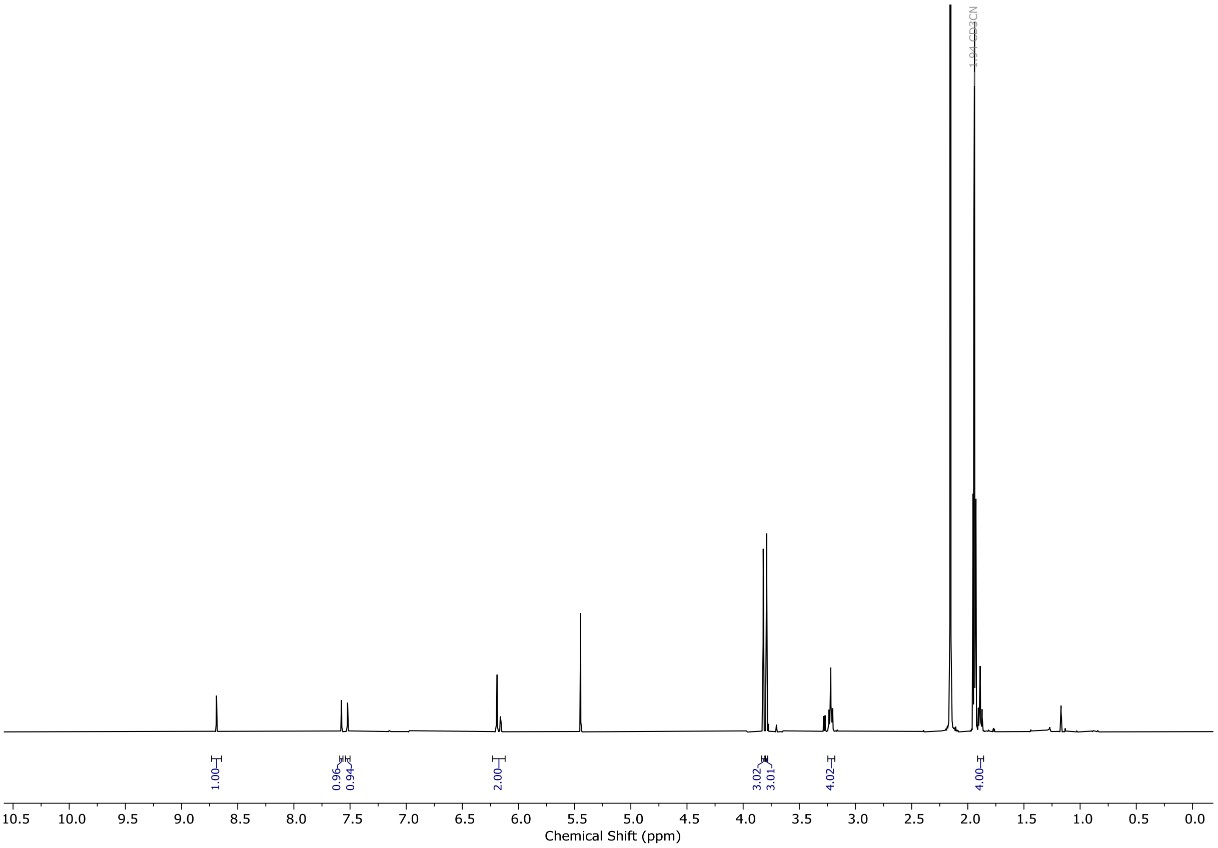

Figure S12. ^1H NMR (400 MHz, 298 K, CD_3CN) of **1o**.

(*E*)-1-(1-methyl-1*H*-pyrazol-4-yl)-*N*-phenylmethanimine, 2a

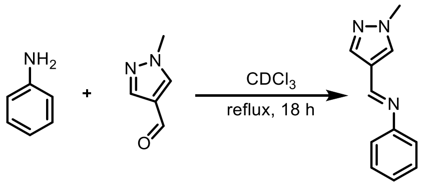

In oven-dried glassware charged with 3 Å molecular sieves, aniline (88.0 mg, 0.9 mmol, 1.3 eq) and 1-methyl-1*H*-pyrazole-4-carbaldehyde (80.0 mg, 0.7 mmol, 1.0 eq) were dissolved in $CDCl_3$ (2.0 mL) under N_2. The mixture was heated to reflux for 18 hours under N_2, and reaction completion was determined by TLC. The solvent was then removed in vacuo. The solid was purified via flash-chromatography using EtOAc with 1% Triethylamine to afford product **2a** as white crystalline solid (121.1 mg, 93% yield). ^{1}H NMR (400 MHz, 298 K, CD_3CN) δ 8.40 (s, 1H), 7.94 (s, 1H), 7.86 – 7.85 (m, 1H), 7.40 – 7.35 (m, 2H), 7.22 – 7.17 (m, 1H), 7.15 – 7.12 (m, 2H), 3.90 (s, 3H); ^{13}C NMR (101 MHz, 298 K, CD_3CN) δ 153.42, 153.24, 139.34, 132.48, 129.75, 125.89, 122.18, 121.22, 39.18; HR ESI-MS (MeCN) for $[C_{11}H_{11}N_3+H]^+$: m/z calcd: 186.1026; found 186.1025; −0.5 ppm error.

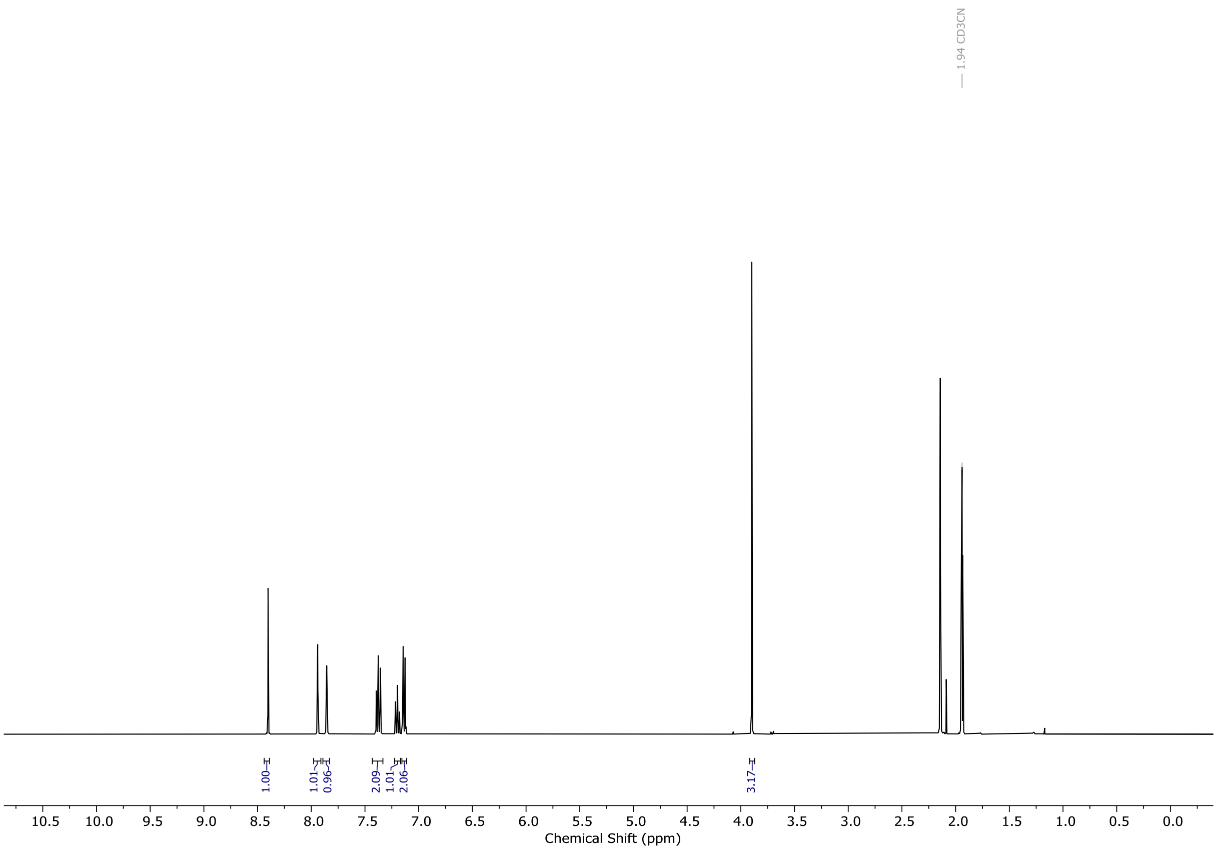

Figure S13. ^{1}H NMR (400 MHz, 298 K, CD_3CN) of **2a**.

***E*)-*N*-(2-fluoro-6-(pyrrolidin-1-yl)phenyl)-1-(1-methyl-1*H*-pyrazol-4-yl)methanimine, 2b**

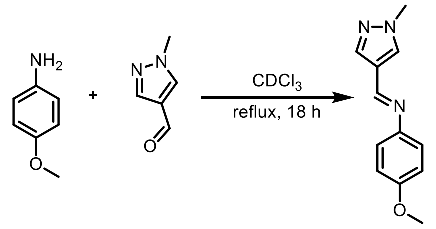

In oven-dried glassware charged with 3 Å molecular sieves, 4-methoxyaniline (116.3 mg, 0.9 mmol, 1.3 eq) and 1-methyl-1*H*-pyrazole-4-carbaldehyde (80.0 mg, 0.7 mmol, 1.0 eq) were dissolved in $CDCl_3$ (2.0 mL) under N_2. The mixture was heated to reflux for 18 hours under N_2, and reaction completion was determined by TLC. The solvent was then removed in vacuo. The solid was purified via flash-chromatography using EtOAc with 1% Triethylamine to afford product **2b** as white solid (138.2 mg, 92% yield). ^1H NMR (400 MHz, 298 K, CD_3CN) δ 8.41 (s, 1H), 7.91 (s, 1H), 7.83 (d, J = 0.7 Hz, 1H), 7.16 – 7.12 (m, 2H), 6.95 – 6.90 (m, 2H), 3.89 (s, 3H), 3.79 (s, 3H); ^{13}C NMR (101 MHz, 298 K, CD_3CN) δ 158.83, 151.92, 146.44, 139.51, 132.49, 122.76, 115.29, 56.05, 39.52; HR ESI-MS (MeCN) for $[C_{12}H_{13}ON_3+H]+$: m/z calcd: 216.1131; found: 216.1130; −0.5 ppm error.

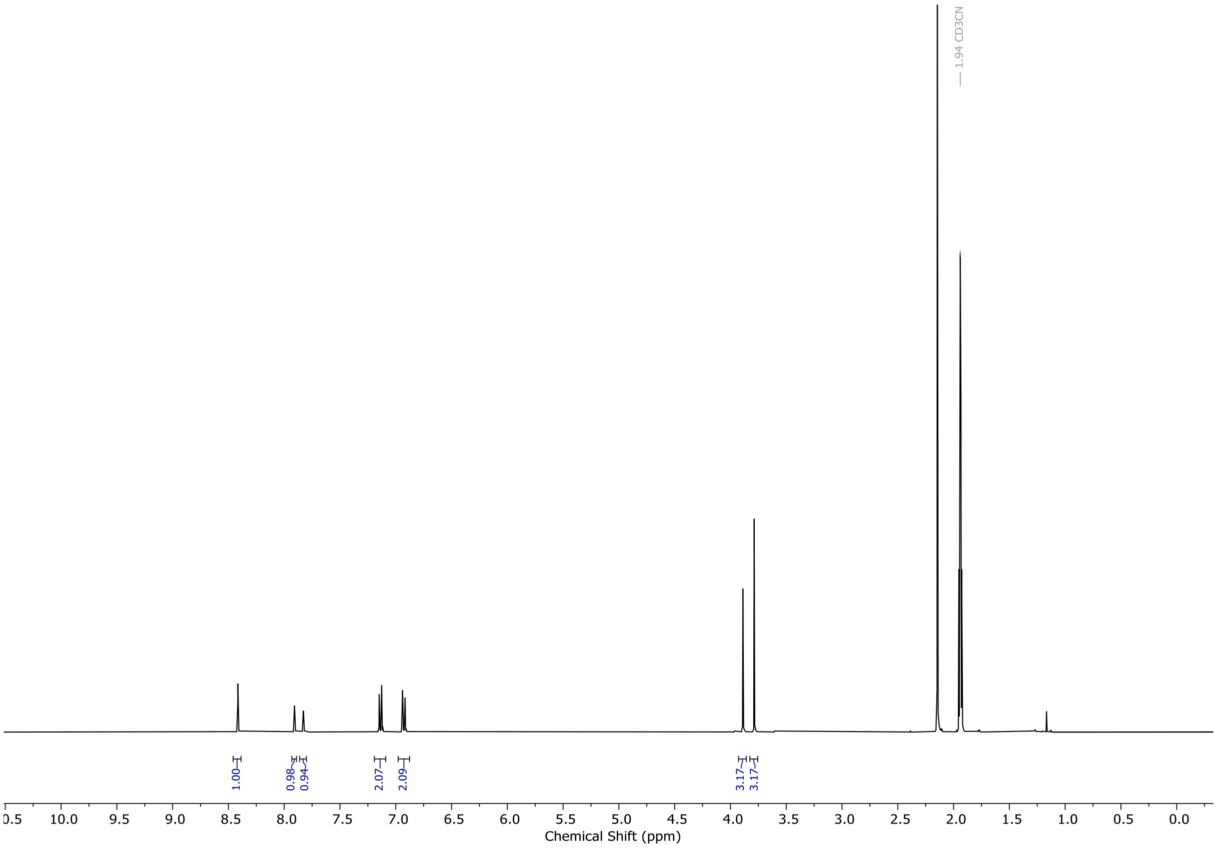

Figure S14. ^1H NMR (400 MHz, 298 K, CD_3CN) of **2b**.

<u>(*E*)-1-(1-methyl-1*H*-pyrazol-4-yl)-*N*-(4-nitrophenyl)methanimine, 2c</u>

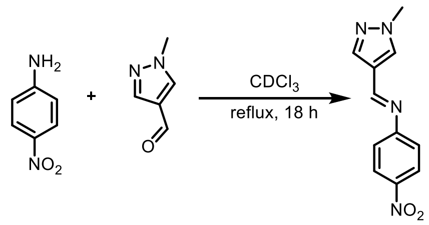

In oven-dried glassware charged with 3 Å molecular sieves, 4-nitroaniline (130.4 mg, 0.9 mmol, 1.3 eq) and 1-methyl-1*H*-pyrazole-4-carbaldehyde (80.0 mg, 0.7 mmol, 1.0 eq) were dissolved in $CDCl_3$ (2.0 mL) under N_2. The mixture was heated to reflux for 18 hours under N_2, and reaction completion was determined by TLC. The solvent was then removed in vacuo. The solid was purified via flash-chromatography using EtOAc with 1% Triethylamine to afford product **2c** as yellow solid (86.8 mg, 52% yield). ^{1}H NMR (400 MHz, 298 K, CD_3CN) δ 8.42 (s, 1H), 8.27 – 8.19 (m, 2H), 8.01 (s, 1H), 7.91 (s, 1H), 7.29 – 7.21 (m, 2H), 3.91 (s, 3H); ^{13}C NMR (101 MHz, 298 K, CD_3CN) δ 159.50, 156.51, 140.19, 133.73, 127.10, 125.88, 122.33, 113.64, 39.63; HR ESI-MS (MeCN) for $[C_{11}H_{10}N_4O_2+Na]^+$: m/z calcd: 253.0696; found: 253.0699; 1.2 ppm error.

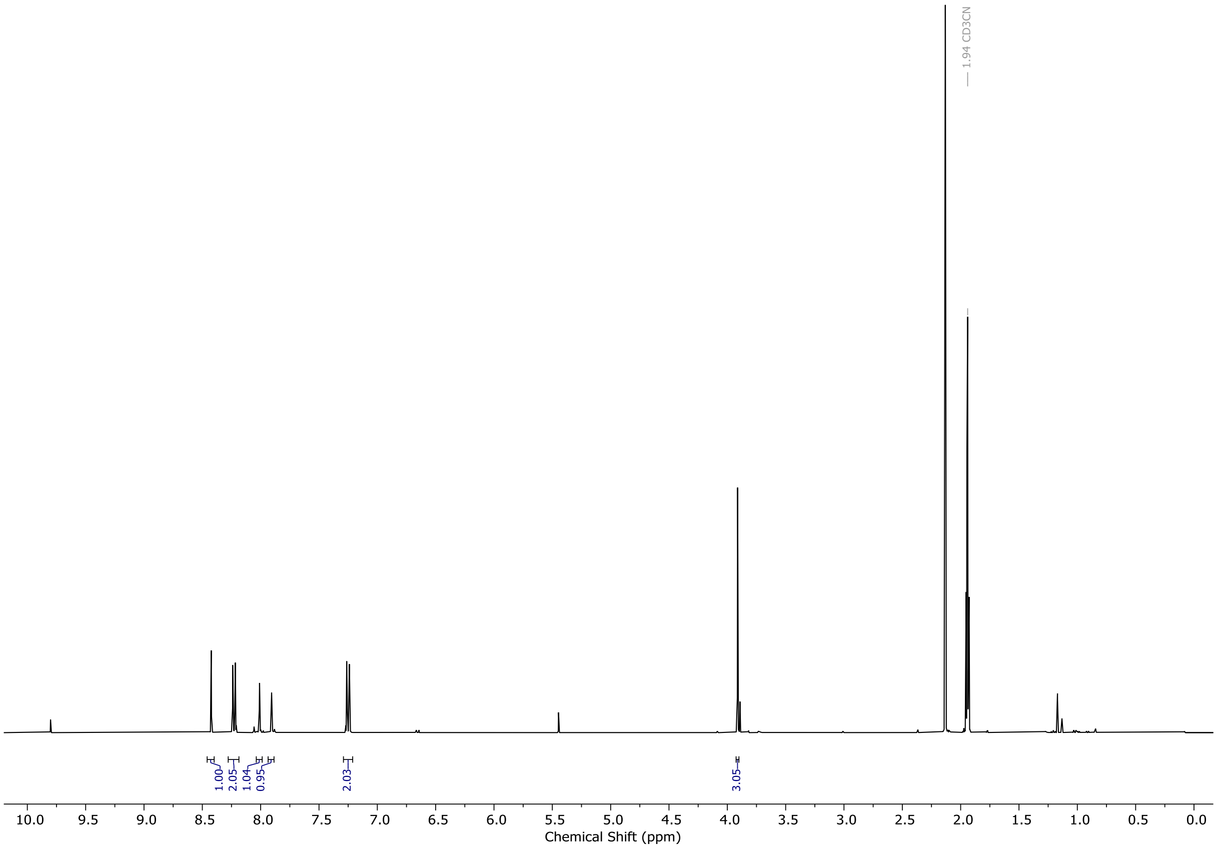

Figure S15. ^{1}H NMR (400 MHz, 298 K, CD_3CN) of **2c**. The small amount of aldehyde presents due to constant hydrolysation of this unstable imine.

(*E*)-*N*,*N*-dimethyl-4-(((1-methyl-1*H*-pyrazol-4-yl)methylene)amino)aniline, 2d

Synthesis of *N*,*N*-dimethyl-4-nitroaniline, S8

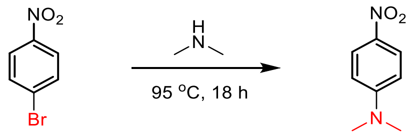

To a flask was added 1-bromo-4-nitrobenzene (1.01 g, 5 mmol, 1 eq) and dimethylamine (40% in water, 13.3 mL, 100 mmol, 20 eq). The mixture was then left to stir at 95 °C for 18 h under an N_2 atmosphere. The reaction progress was monitored by TLC. After the complete consumption of 1-bromo-4-nitrobenzene, 10 mL water was added, and the mixture was extracted with dichloromethane. The resulting yellow solution was dried over $MgSO_4$ and purified by column chromatography on silica gel using an eluent of 9:1 cyclohexane:ethyl acetate. The product **S8** was dried under high vacuum affording a yellow crystalline solid (0.53 g, 64% yield). ^{1}H NMR (400 MHz, 298K, $CDCl_3$) δ 8.17 – 8.09 (m, 2H), 6.65 – 6.57 (m, 2H), 3.12 (s, 6H); ^{13}C NMR (101 MHz, 298K, $CDCl_3$) δ 132.78, 126.28, 125.16, 110.35, 40.42; HR ESI-MS (MeCN) for $[C_8H_{10}N_2O_2+Na]^+$: m/z calcd: 189.0634; found: 189.0631; −1.6 ppm error.

Synthesis of *N*^1^,*N*^1^-dimethylbenzene-1,4-diamine, S9

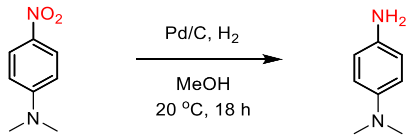

Precursor **S8** (0.50 g, 3 mmol, 1 eq) was dissolved in MeOH (20 mL) and degassed with N_2. To this solution was added 10% Pd/C (0.03 g, 5 wt%) and the solution was degassed for a further 5 min, followed by displacing the N_2 with H_2. The reaction was left to stir vigorously overnight under a H_2 atmosphere. The mixture was filtered through Celite and concentrated in *vacuo*, yielding a colourless oil that was used without further purification, **S9** (0.40 g, 98% yield). ^{1}H NMR (400 MHz, 298K, $CDCl_3$) δ 6.78 – 6.56 (m, 4H), 3.32 (s, 2H), 2.82 (s, 6H); ^{13}C NMR (101 MHz, 298K, $CDCl_3$) δ 116.72, 115.72, 42.27; HR ESI-MS (MeCN) for $[C_8H_{12}N_2+H]^+$: m/z calcd: 137.1073; found: 137.1078; 3.6 ppm error.

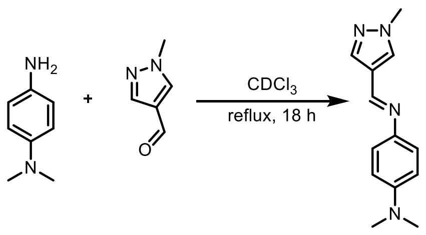

In oven-dried glassware charged with 3 Å molecular sieves, **S9** (128.6 mg, 0.9 mmol, 1.3 eq) and 1-methyl-1*H*-pyrazole-4-carbaldehyde (80.0 mg, 0.7 mmol, 1.0 eq) were dissolved in $CDCl_3$ (2.0 mL) under N_2. The mixture was heated to reflux for 18 hours under N_2, and reaction completion was determined by TLC. The solvent was then removed in vacuo. The solid was purified via flash-chromatography using EtOAc with 1% Triethylamine to afford product **2d** as white crystalline solid (137.6 mg, 83% yield). ^{1}H NMR (400 MHz, 298 K, CD_3CN) δ 8.43 (s, 1H), 7.87 (s, 1H), 7.80 (d, *J* = 0.7 Hz, 1H), 7.16 − 7.08 (m, 2H), 6.80 − 6.72 (m, 2H), 3.88 (s, 3H), 2.92 (s, 6H); ^{13}C NMR (101 MHz, 298 K, CD_3CN) δ 150.31, 149.51, 142.31, 139.25, 132.05, 122.98, 122.56, 113.92, 40.92, 39.44; HR ESI-MS (MeCN) for $[C_{13}H_{16}N_4+H]^+$: m/z calcd: 229.1448; found: 229.1449; 0.4 ppm error.

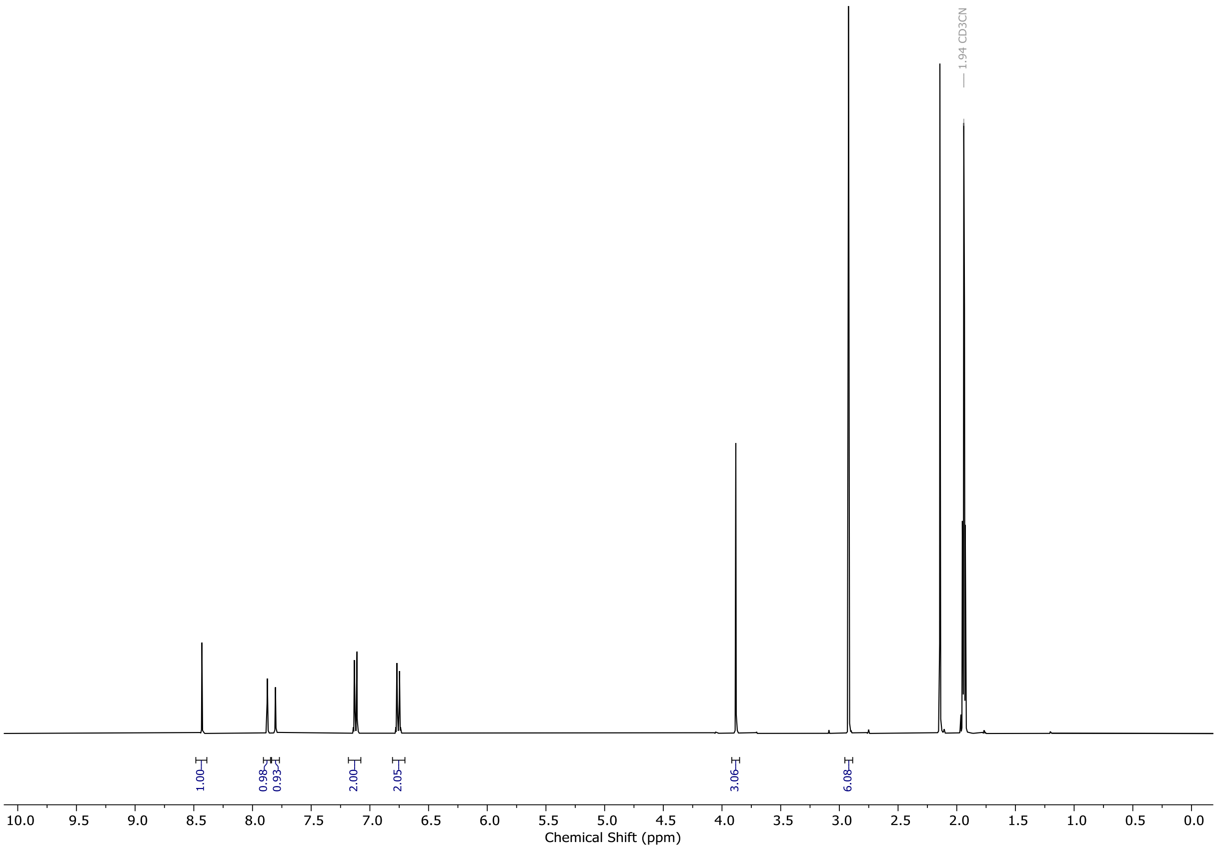

Figure S16. ^{1}H NMR (400 MHz, 298 K, CD_3CN) of **2d**.

2.1 (*E*)-1-(1-methyl-1*H*-pyrazol-4-yl)-*N*-(2-(pyrrolidin-1-yl)phenyl)methanimine, 2e

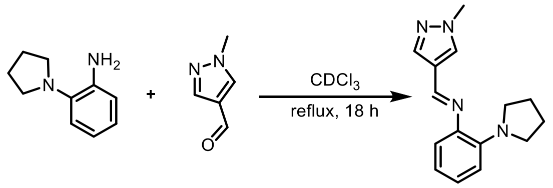

In oven-dried glassware charged with 3 Å molecular sieves, 2-(pyrrolidin-1-yl)aniline (153.2 mg, 0.9 mmol, 1.3 eq) and 1-methyl-1*H*-pyrazole-4-carbaldehyde (80.0 mg, 0.7 mmol, 1.0 eq) were dissolved in $CDCl_3$ (2.0 mL) under N_2. The mixture was heated to reflux for 18 hours under N_2, and reaction completion was determined by TLC. The solvent was then removed in vacuo. The solid was purified via flash-chromatography using EtOAc with 1% Triethylamine to afford product **2e** as yellow solid (151.2 mg, 82% yield). ^1H NMR (400 MHz, 298 K, CD_3CN) δ 8.27 (s, 1H), 7.91 (s, 1H), 7.84 (s, 1H), 7.05 – 7.01 (m, 1H), 6.81 (dd, *J* = 7.6, 1.7 Hz, 1H), 6.74 – 6.68 (m, 2H), 3.89 (s, 3H), 3.40 – 3.35 (m, 4H), 1.89 – 1.85 (m, 4H); ^{13}C NMR (101 MHz, 298 K, CD_3CN) δ 150.47, 144.57, 142.68, 139.53, 132.36, 126.91, 123.22, 119.92, 119.05, 118.26, 115.34, 51.72, 39.51, 25.93; HR ESI-MS (MeCN) for $[C_{15}H_{18}N_4+H]^+$: m/z calcd: 255.1604; found 255.1597; −2.7 ppm error.

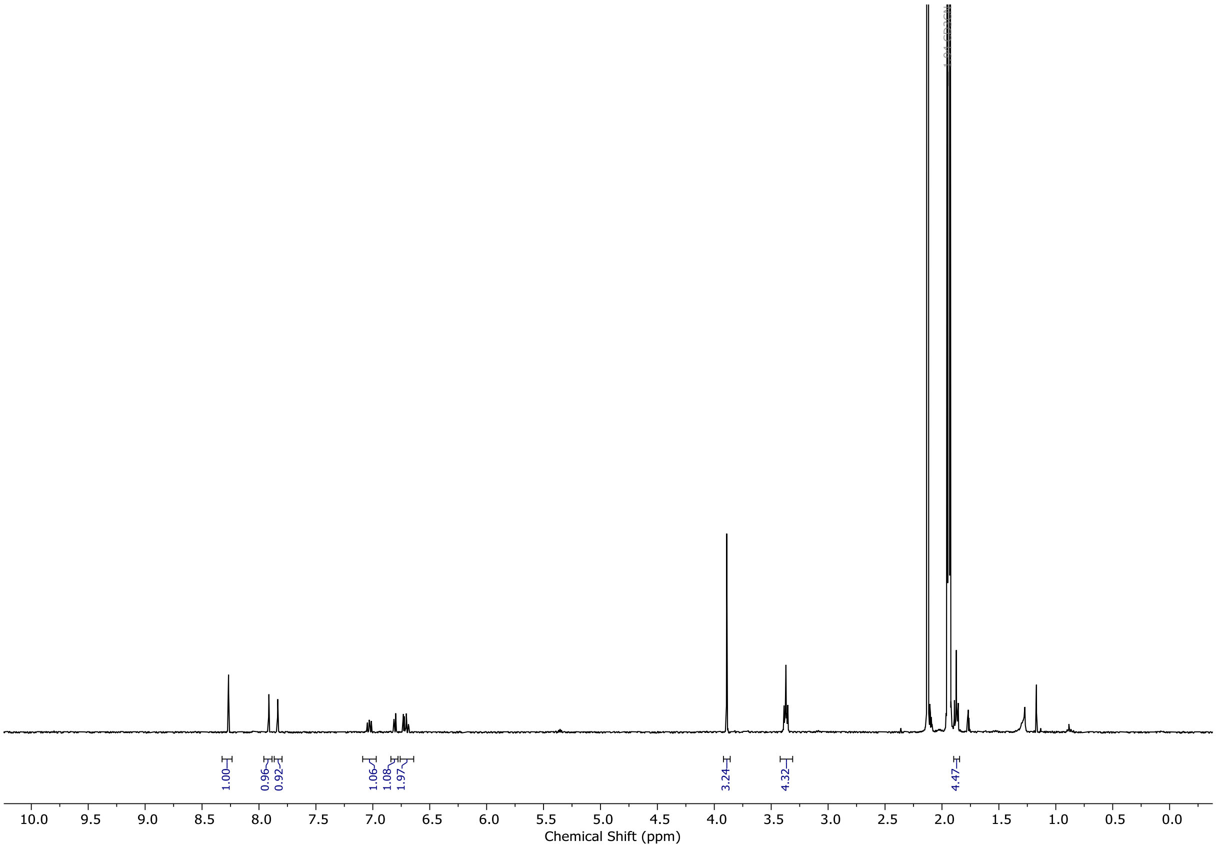

Figure S17. ^1H NMR (400 MHz, 298 K, CD_3CN) of **2e**.

(*E*)-*N*-(2,6-di(pyrrolidin-1-yl)phenyl)-1-(1-methyl-1*H*-pyrazol-4-yl)methanimine, 2f

Synthesis of 1,1'-(2-nitro-1,3-phenylene)dipyrrolidine, S10

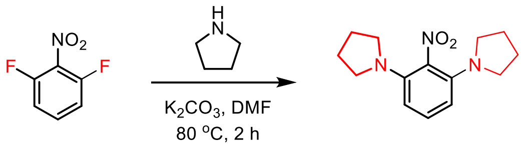

To an oven dried flask was added 1,3-difluoro-2-nitrobenzene (0.64 g, 4 mmol, 1 eq), K_2CO_3 (2.21 g, 16 mmol, 4 eq) and 4 mL of anhydrous DMF. To this suspension was added pyrrolidine (1.14 g, 16 mmol, 4 eq) and the mixture was then left to stir at 80 °C for 2 h under an N_2 atmosphere. The mixture was then filtered under gravity and the filtrate was concentrated in *vacuo*. The resulting oil was purified by column chromatography on silica gel using an eluent of 9:1 cyclohexane:ethyl acetate. The product **S10** was dried under high vacuum affording a red crystalline solid (0.88 g, 84% yield). ^1H NMR (400 MHz, 298 K, $CDCl_3$) δ 7.09 (t, J = 8.3 Hz, 1H), 6.35 (d, J = 8.3 Hz, 2H), 3.31 – 3.16 (m, 8H), 1.97 – 1.84 (m, 8H). ^{13}C NMR (101 MHz, 298 K, $CDCl_3$) δ 143.66, 130.38, 106.40, 49.73, 25.68, 25.57; HR ESI-MS (MeCN) for $[C_{14}H_{19}N_3O_2+H]^+$: m/z calcd: 262.1550; found: 262.1543; −2.67 ppm error.

Synthesis of 2,6-di(pyrrolidin-1-yl)aniline, S11

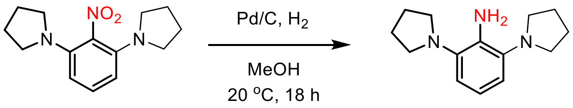

Precursor **S10** (0.78 g, 3 mmol, 1 eq) was dissolved in MeOH (20 mL) and degassed with N_2. To this solution was added 10% Pd/C (0.04 g, 5 wt%) and the solution was degassed for a further 5 min, followed by displacing the N_2 with H_2. The reaction was left to stir vigorously overnight under a H_2 atmosphere. The mixture was filtered through Celite and concentrated in *vacuo*, yielding a colourless oil that was used without further purification, **S11** (0.69 g, 99% yield). ^1H NMR (400 MHz, 298K, $CDCl_3$) δ 6.77 – 6.66 (m, 3H), 4.04 (s, 2H), 3.10 (m, 8H), 1.54 (m, 8H); ^{13}C NMR (101 MHz, 298 K, $CDCl_3$) δ 138.11, 135.97, 117.68, 112.63, 50.92, 24.24; HR ESI-MS (MeCN) for $[C_{14}H_{21}N_3+H]^+$: m/z calcd: 232.1808; found: 232.1811; 1.3 ppm error.

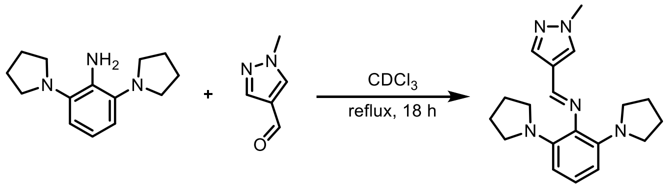

In oven-dried glassware charged with 3 Å molecular sieves, **S11** (218.5 mg, 0.9 mmol, 1.3 eq) and 1-methyl-1*H*-pyrazole-4-carbaldehyde (80.0 mg, 0.7 mmol, 1.0 eq) were dissolved in $CDCl_3$ (2.0 mL) under N_2. The mixture was heated to reflux for 18 hours under N_2, and reaction completion was determined by TLC. The solvent was then removed in vacuo. The solid was purified via flash-chromatography using EtOAc with 1% Triethylamine to afford product **2f** as yellow solid (186.8 mg, 80% yield). ^1H NMR (400 MHz, 298 K, CD_3CN) δ 7.93 (s, 1H), 7.91 – 7.81 (m, 2H), 6.84 (t, J = 8.1 Hz, 1H), 6.42 (d, J = 8.1 Hz, 2H), 3.89 (s, 3H), 3.14 – 3.01 (m, 8H), 1.82 – 1.69 (m, 8H); ^{13}C NMR (101 MHz, 298 K, CD_3CN) δ 155.89, 143.13, 139.30, 136.14, 132.26, 124.81, 123.01, 108.43, 50.89, 39.49, 25.38; HR ESI-MS (MeCN) for $[C_{19}H_{25}N_5+H]^+$: m/z calcd: 324.2183; found 324.2186; 0.9 ppm error.

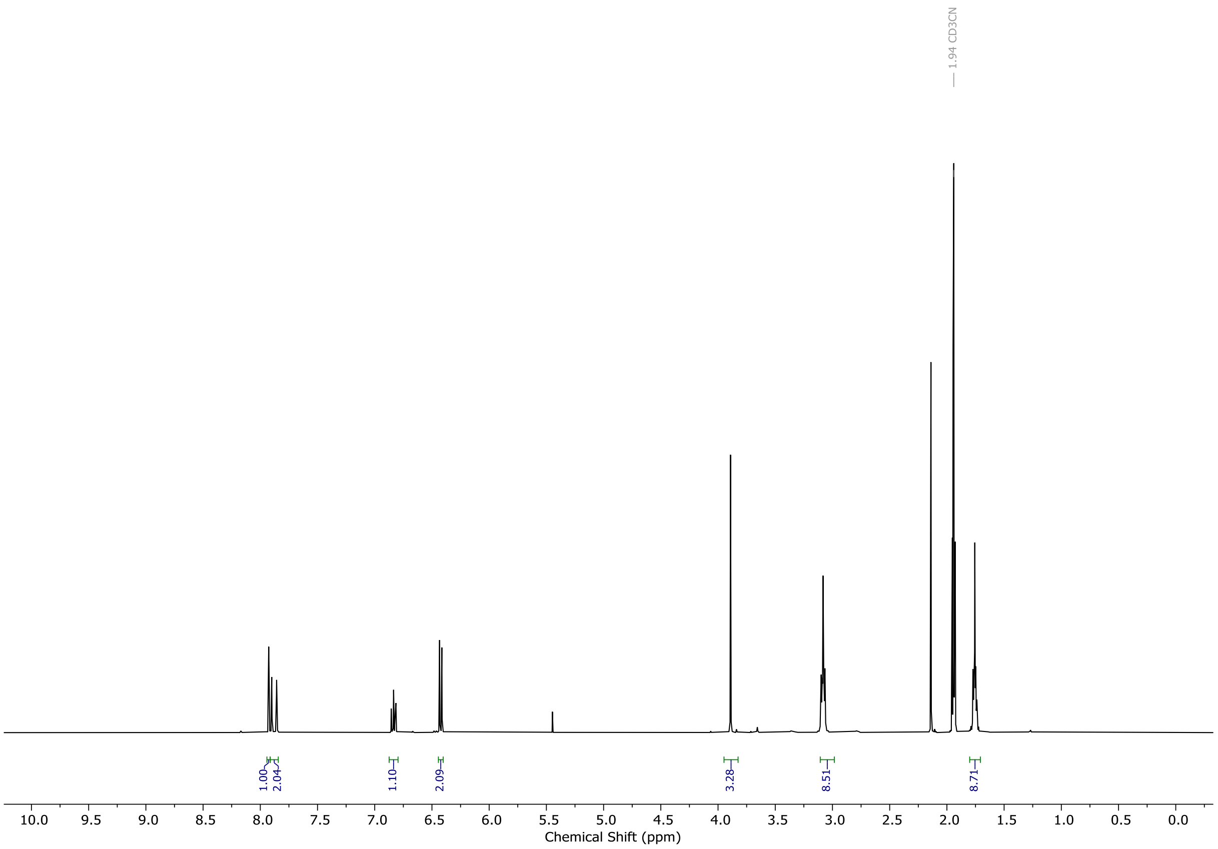

Figure S18. ^1H NMR (400 MHz, 298 K, CD_3CN) of **2f**.

(*E*)-*N*-(2-fluorophenyl)-1-(1-methyl-1*H*-pyrazol-4-yl)methanimine, 2k

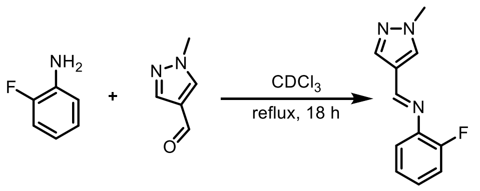

In oven-dried glassware charged with 3 Å molecular sieves, 2-fluoroaniline (145.3 mg, 1.3 mmol, 1.8 eq) and 1-methyl-1*H*-pyrazole-4-carbaldehyde (80.0 mg, 0.7 mmol, 1.0 eq) were dissolved in $CDCl_3$ (2.0 mL) under N_2. The mixture was heated to reflux for 18 hours under N_2, and reaction completion was determined by TLC. The solvent was then removed in vacuo. The solid was purified via flash-chromatography using EtOAc with 1% Triethylamine to afford product **2k** as colorless liquid (128.9 mg, 87% yield). ^{1}H NMR (400 MHz, 298 K, CD_3CN) δ 8.43 (d, *J* = 0.7 Hz, 1H), 7.97 (d, *J* = 0.7 Hz, 1H), 7.88 (d, *J* = 0.7 Hz, 1H), 7.23 – 7.08 (m, 4H), 3.90 (s, 3H); ^{13}C NMR (101 MHz, 298 K, CD_3CN) δ 157.50, 156.22, 155.06, 114.55, 139.89, 133.19, 127.25, 125.79, 122.35, 116.83, 39.60; ^{19}F NMR (376 MHz, 298 K, CD_3CN) δ −129.00; HR ESI-MS (MeCN) for $[C_{11}H_{10}N_3F+H]^+$: m/z calcd: 204.0932; found 204.0929; −1.0 ppm error.

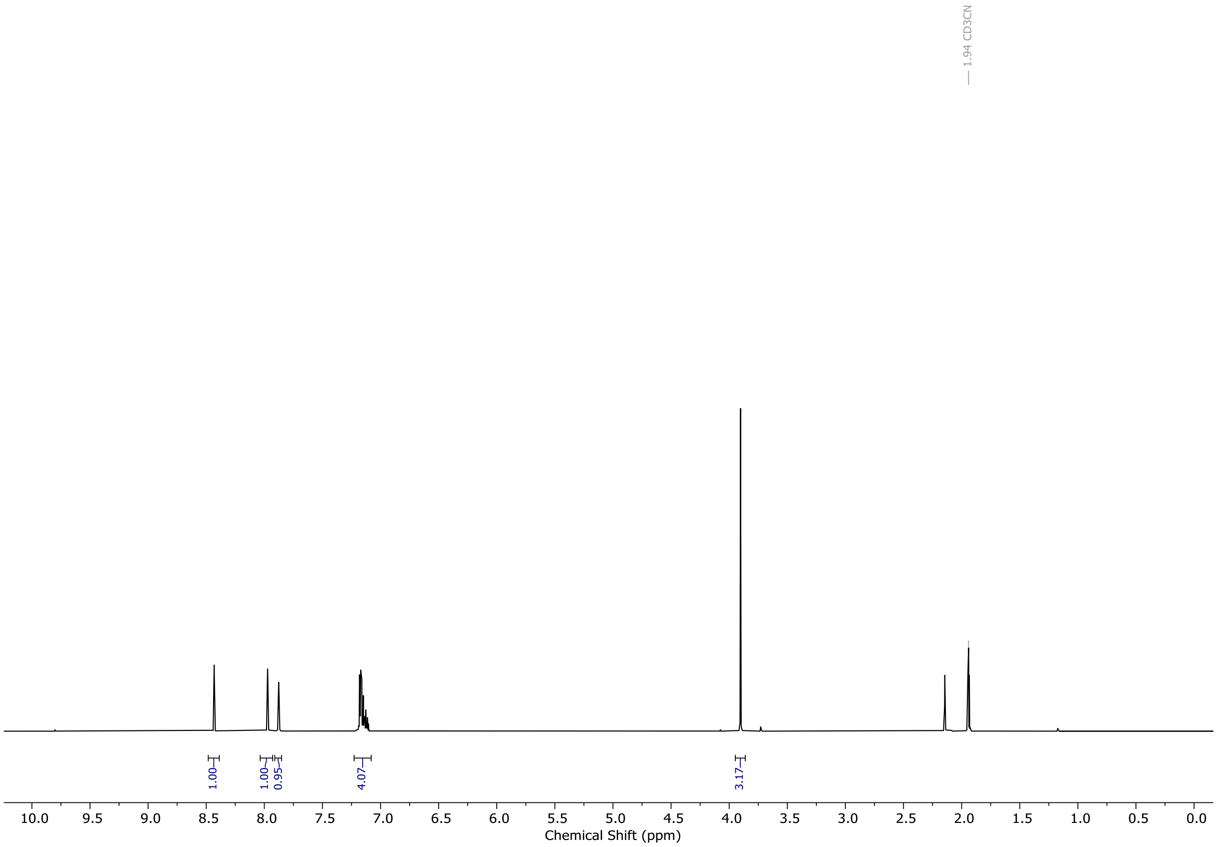

Figure S19. ^{1}H NMR (400 MHz, 298 K, CD_3CN) of **2k**.

(*E*)-*N*-(2,6-difluorophenyl)-1-(1-methyl-1*H*-pyrazol-4-yl)methanimine, 2l

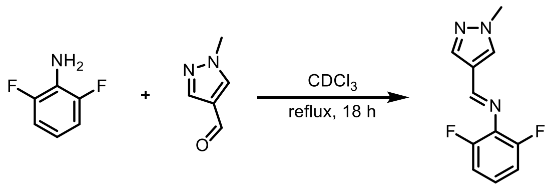

In oven-dried glassware charged with 3 Å molecular sieves, 2,6-difluoroaniline (121.9 mg, 0.9 mmol, 1.3 eq) 1-methyl-1*H*-pyrazole-4-carbaldehyde (80.0 mg, 0.7 mmol, 1.0 eq) were dissolved in $CDCl_3$ (2.0 mL) under N_2. The mixture was heated to reflux for 18 hours under N_2, and reaction completion was determined by TLC. The solvent was then removed in vacuo. The solid was purified via flash-chromatography using EtOAc with 1% Triethylamine to afford product **2l** as white crystalline solid (143.8 mg, 89% yield). ^1H NMR (400 MHz, 298 K, CD_3CN) δ 8.48 (s, 1H), 8.00 (d, *J* = 2.3 Hz, 1H), 7.90 (d, *J* = 2.3 Hz, 1H), 7.11 (ddd, *J* = 8.4, 6.1, 4.5, 1.0 Hz, 1H), 7.03 (ddt, *J* = 8.8, 7.5, 1.5 Hz, 2H), 3.91 (t, *J* = 1.5 Hz, 3H); ^{13}C NMR (101 MHz, 298 K, CD_3CN) δ 160.81, 157.17, 154.73, 139.93, 133.49, 125.75, 118.26, 117.85, 112.89, 112.64, 39.64; ^{19}F NMR (376 MHz, 298 K, CD_3CN) δ −127.66; HR ESI-MS (MeCN) for $[C_{11}H_9N_3F_2+H]^+$: m/z calcd: 222.0837; found 222.0838; 0.5 ppm error.

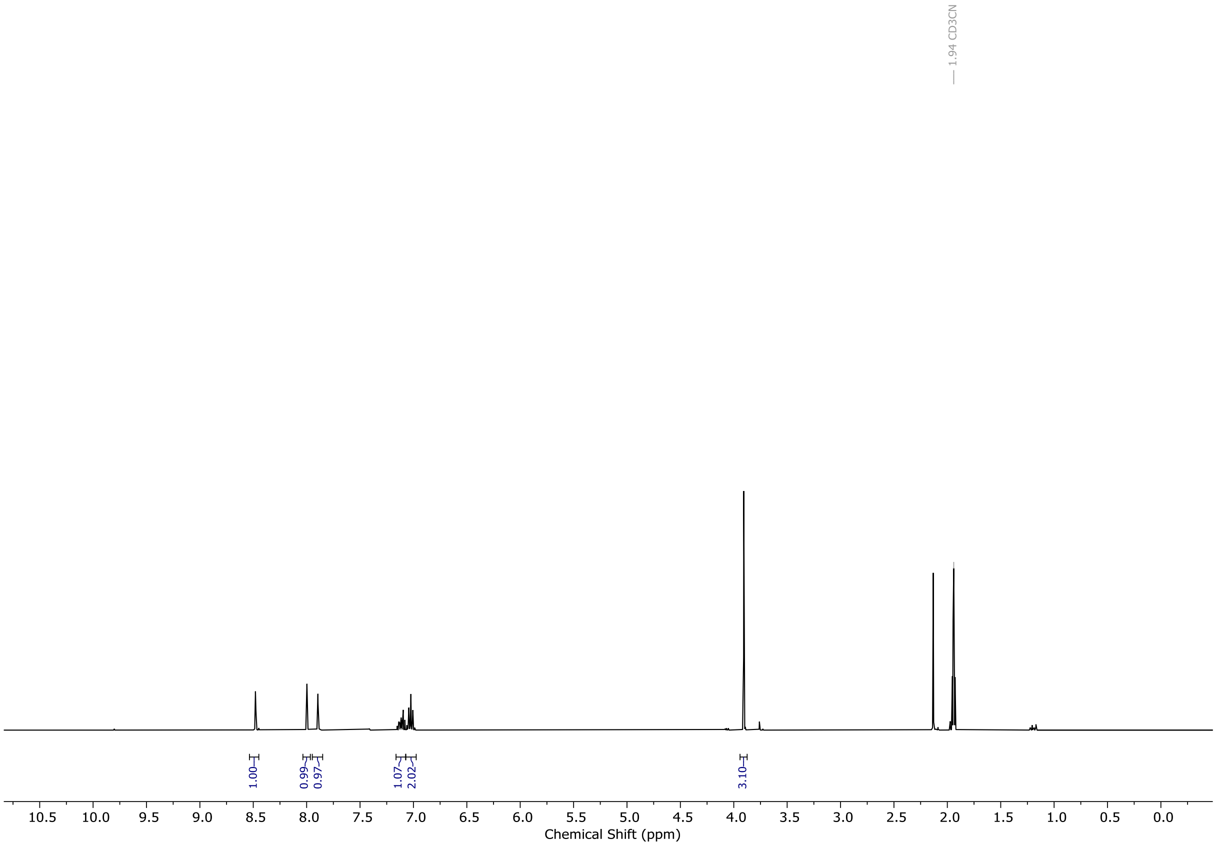

Figure S20. ^1H NMR (400 MHz, 298 K, CD_3CN) of **2l**.

(*E*)-*N*-(2-fluoro-6-(pyrrolidin-1-yl)phenyl)-1-(1-methyl-1*H*-pyrazol-4-yl)methanimine, 2m

Synthesis of 1-(3-fluoro-2-nitrophenyl)pyrrolidine, S12

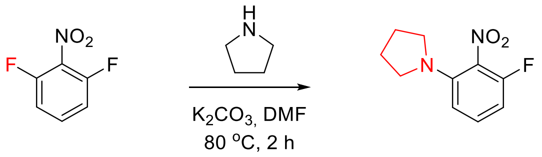

To an oven dried flask was added 1,3-difluoro-2-nitrobenzene (3.00 g, 18.9 mmol, 1 eq), K_2CO_3 (5.20 g, 37.7 mmol, 2 eq) and 15 mL of anhydrous DMF. To this suspension was added pyrrolidine (1.34 g, 18.9 mmol, 1 eq) and the mixture was then left to stir at 80 °C for 2 h under an N_2 atmosphere. The mixture was then filtered under gravity and the filtrate was concentrated in *vacuo*. The resulting oil was purified by column chromatography on silica gel using an eluent of 9:1 cyclohexane:ethyl acetate. The product **S12** was dried under high vacuum affording a yellow oil (3.69 g, 93% yield). ^1H NMR (400 MHz, 298 K, $CDCl_3$) δ 7.20 (ddd, *J* = 8.8, 8.2, 6.3 Hz, 1H), 6.55 (dt, *J* = 8.9, 1.3 Hz, 1H), 6.45 (ddd, *J* = 9.5, 8.2, 1.1 Hz, 1H), 3.30 – 3.22 (m, 4H), 1.99 – 1.94 (m, 4H); ^{13}C NMR (101 MHz, 298 K, $CDCl_3$): δ 157.28, 154.75, 142.32, 131.40, 110.99, 102.55, 49.22, 25.73 ppm; ^{19}F NMR (376 MHz, 298 K, Chloroform-*d*): −126.57 ppm; HR ESI-MS (MeCN) for $[C_{10}H_{11}O_2FN_2+H]^+$: m/z calcd: 211.0877; found: 211.0874; 1.4 ppm error.

Synthesis of 2-fluoro-6-(pyrrolidin-1-yl)aniline, S13

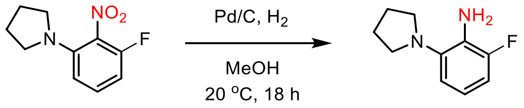

Precursor **S12** (3.50 g, 16.7 mmol, 1 eq) was dissolved in MeOH (100 mL) and degassed with N_2. To this solution was added 10% Pd/C (0.18 g, 5 wt%) and the solution was degassed for a further 5 min, followed by displacing the N_2 with H_2. The reaction was left to stir vigorously overnight under a H_2 atmosphere. The mixture was filtered through Celite and concentrated in *vacuo*, yielding a colourless oil that was used without further purification, **S13** (2.98 g, 99% yield). ^1H NMR (400 MHz, 298 K, $CDCl_3$) δ 6.78 – 6.70 (m, 2H), 6.68 – 6.61 (m, 1H), 3.79 (s, 2H), 3.12 – 3.04 (m, 4H), 1.97 – 1.89 (m, 4H); ^{13}C NMR (101 MHz, 298 K, $CDCl_3$) δ 153.56, 139.90, 129.15, 117.42, 113.68, 109.44, 50.86, 24.29; ^{19}F NMR (376 MHz, 298 K, $CDCl_3$) δ −137.75; HR ESI-MS (MeCN) for $[C_{10}H_{13}FN_2+H]^+$: m/z calcd: 181.1136; found: 181.1135; −0.6 ppm error.

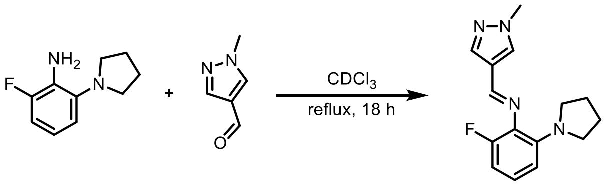

In oven-dried glassware charged with 3 Å molecular sieves, **S13** (170.2 mg, 0.9 mmol, 1.3 eq) 1-methyl-1*H*-pyrazole-4-carbaldehyde (80.0 mg, 0.7 mmol, 1.0 eq) were dissolved in $CDCl_3$ (2.0 mL) under N_2. The mixture was heated to reflux for 18 hours under N_2, and reaction completion was determined by TLC. The solvent was then removed in vacuo. The solid was purified via flash-chromatography using EtOAc with 1% Triethylamine to afford product **2m** as yellow solid (180.8 mg, 91% yield). ^1H NMR (400 MHz, 298 K, CD_3CN) δ 8.31 (d, *J* = 4.1 Hz, 1H), 7.94 (s, 1H), 7.86 (d, *J* = 0.7 Hz, 1H), 6.91 (td, *J* = 8.3, 6.4 Hz, 1H), 6.56 – 6.50 (m, 2H), 3.89 (s, 3H), 3.35 – 3.29 (m, 4H), 1.86 – 1.81 (m, 4H); ^{13}C NMR (101 MHz, 298 K, CD_3CN) δ 157.03, 149.45, 139.52, 132.68, 125.67, 125.56, 118.26, 111.09, 105.72, 105.50, 51.75, 39.56, 25.96; ^{19}F NMR (376 MHz, 298 K, CD_3CN) δ −133.39; HR ESI-MS (MeCN) for $[C_{15}H_{17}FN_4+H]^+$: m/z calcd: 273.1510; found 273.1510; −0.1 ppm error.

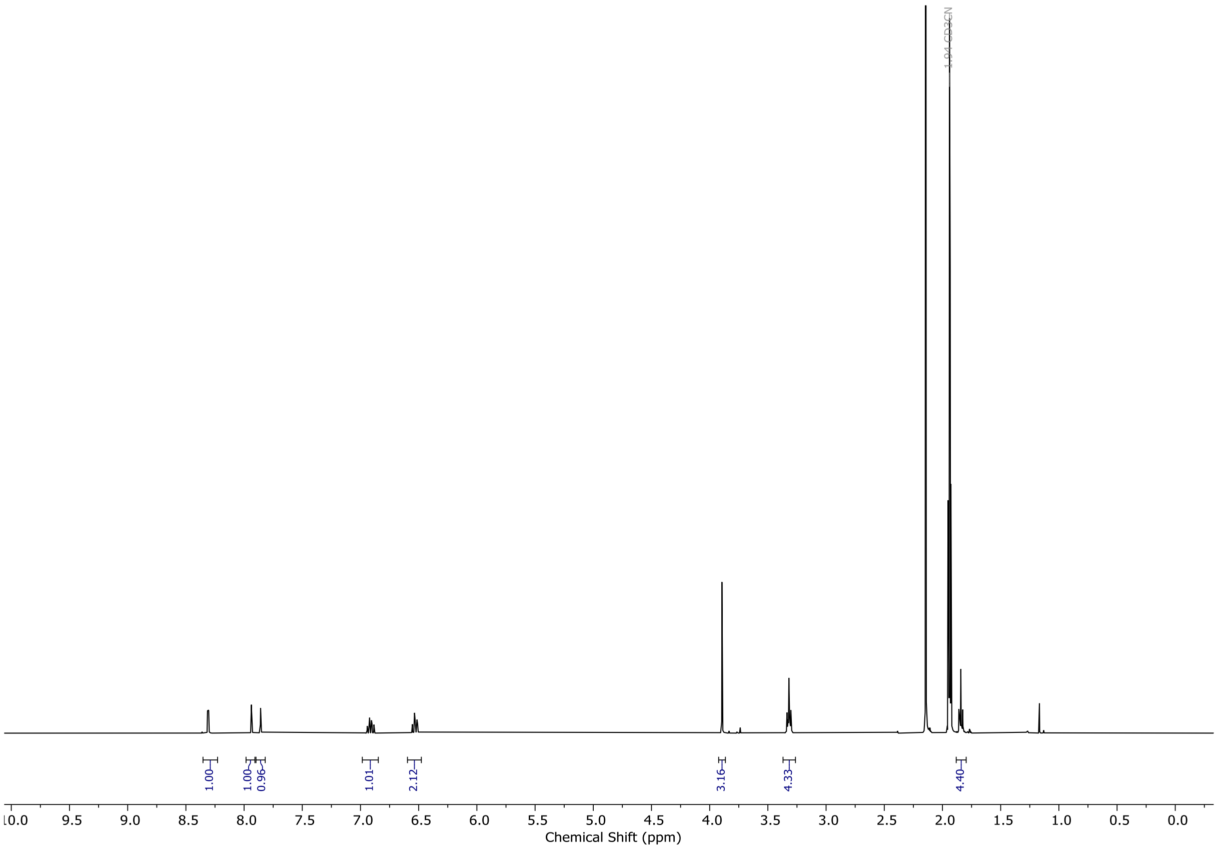

Figure S21. ^1H NMR (400 MHz, 298 K, CD_3CN) of **2m**.

(*E*)-*N*-(2-fluoro-6-(pyrrolidin-1-yl)phenyl)-1-(1-methyl-1*H*-pyrazol-4-yl)methanimine, 2n

Synthesis of 1-(3-fluoro-2-nitrophenyl)piperidine, S14

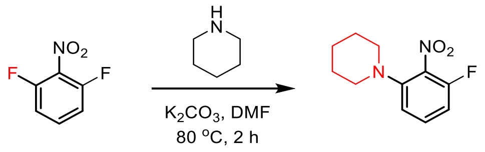

To an oven dried flask was added 1,3-difluoro-2-nitrobenzene (3.00 g, 18.9 mmol, 1 eq), K_2CO_3 (5.20 g, 37.7 mmol, 2 eq) and 15 mL of anhydrous DMF. To this suspension was added piperidine (1.61 g, 18.9 mmol, 1 eq) and the mixture was then left to stir at 80 °C for 2 h under an N_2 atmosphere. The mixture was then filtered under gravity and the filtrate was concentrated in *vacuo*. The resulting oil was purified by column chromatography on silica gel using an eluent of 9:1 cyclohexane:ethyl acetate. The product **S14** was dried under high vacuum affording a yellow oil (3.90 g, 92% yield). ^{1}H NMR (400 MHz, 298 K, $CDCl_3$) δ 7.34 – 7.28 (m, 1H), 6.88 (dt, *J* = 8.5, 1.2 Hz, 1H), 6.79 (ddd, *J* = 9.3, 8.4, 1.1 Hz, 1H), 3.01 – 2.96 (m, 4H), 1.69 – 1.61 (m, 4H), 1.59 – 1.52 (m, 2H); ^{13}C NMR (101 MHz, 298 K, $CDCl_3$) δ 156.14, 147.52, 131.50, 116.56, 109.34, 109.24, 53.21, 26.15, 24.06; ^{19}F NMR (376 MHz, 298 K, $CDCl_3$) δ −126.58; HR ESI-MS (MeCN) for $[C_{11}H_{13}O_2FN_2+H]^+$: m/z calcd: 225.1034; found: 225.1039; 2.2 ppm error.

Synthesis of 2-fluoro-6-(piperidin-1-yl)aniline, S15

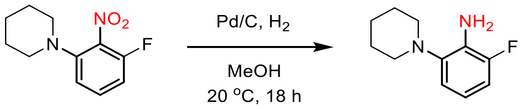

Precursor **S14** (3.50 g, 15.6 mmol, 1 eq) was dissolved in MeOH (100 mL) and degassed with N_2.To this solution was added 10% Pd/C (0.18 g, 5 wt%) and the solution was degassed for a further 5 min, followed by displacing the N_2 with H_2. The reaction was left to stir vigorously overnight under a H_2 atmosphere. The mixture was filtered through Celite and concentrated in *vacuo*, yielding a colorless oil that was used without further purification, **S15** (3.00 g, 99% yield). ^{1}H NMR (400 MHz, 298 K, $CDCl_3$) 6.80 – 6.74 (m, 2H), 6.64 (td, *J* = 8.1, 6.2 Hz, 1H), 3.93 (s, 2H), 2.85 (t, *J* = 5.3 Hz, 4H), 1.72 (m, *J* = 5.6 Hz, 4H), 1.59 (q, *J* = 6.7, 5.9 Hz, 2H); ^{13}C NMR (101 MHz, 298 K, $CDCl_3$) δ 153.17, 142.60, 129.85, 117.10, 115.21, 110.45, 52.69, 27.04, 24.46; ^{19}F NMR (376 MHz, 298 K, $CDCl_3$) δ −137.34; HR ESI-MS (MeCN) for $[C_{11}H_{15}FN_2+H]^+$: m/z calcd: 195.1292; found: 195.1294; 1.0 ppm error.

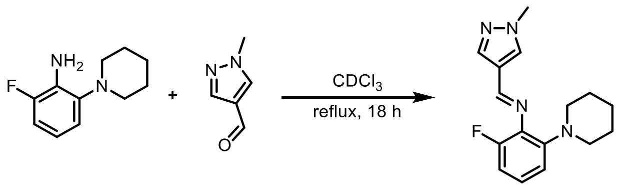

In oven-dried glassware charged with 3 Å molecular sieves, **S15** (183.5 mg, 0.9 mmol, 1.3 eq) 1-methyl-1*H*-pyrazole-4-carbaldehyde (80.0 mg, 0.7 mmol, 1.0 eq) were dissolved in $CDCl_3$ (2.0 mL) under N_2. The mixture was heated to reflux for 18 hours under N_2, and reaction completion was determined by TLC. The solvent was then removed in vacuo. The solid was purified via flash-chromatography using EtOAc with 1% Triethylamine to afford product **2n** as yellow solid (188.9 mg, 91% yield). ^{1}H NMR (400 MHz, 298 K, CD_3CN) δ 8.35 (d, *J* = 2.4 Hz, 1H), 8.05 – 7.82 (m, 2H), 7.06 – 6.94 (m, 1H), 6.83 – 6.71 (m, 2H), 3.90 (s, 3H), 2.98 – 2.85 (m, 4H), 1.59 – 1.46 (m, 6H); ^{13}C NMR (101 MHz, 298 K, CD_3CN) δ 158.23, 155.73, 153.59, 148.71, 139.55, 132.84, 125.40, 122.62, 115.01, 110.90, 52.61, 39.58, 27.73, 24.94; ^{19}F NMR (376 MHz, 298 K, CD_3CN) δ −131.01; HR ESI-MS (MeCN) for $[C_{16}H_{19}FN_4+H]^+$: m/z calcd: 287.1666; found 287.1665; −0.5 ppm error.

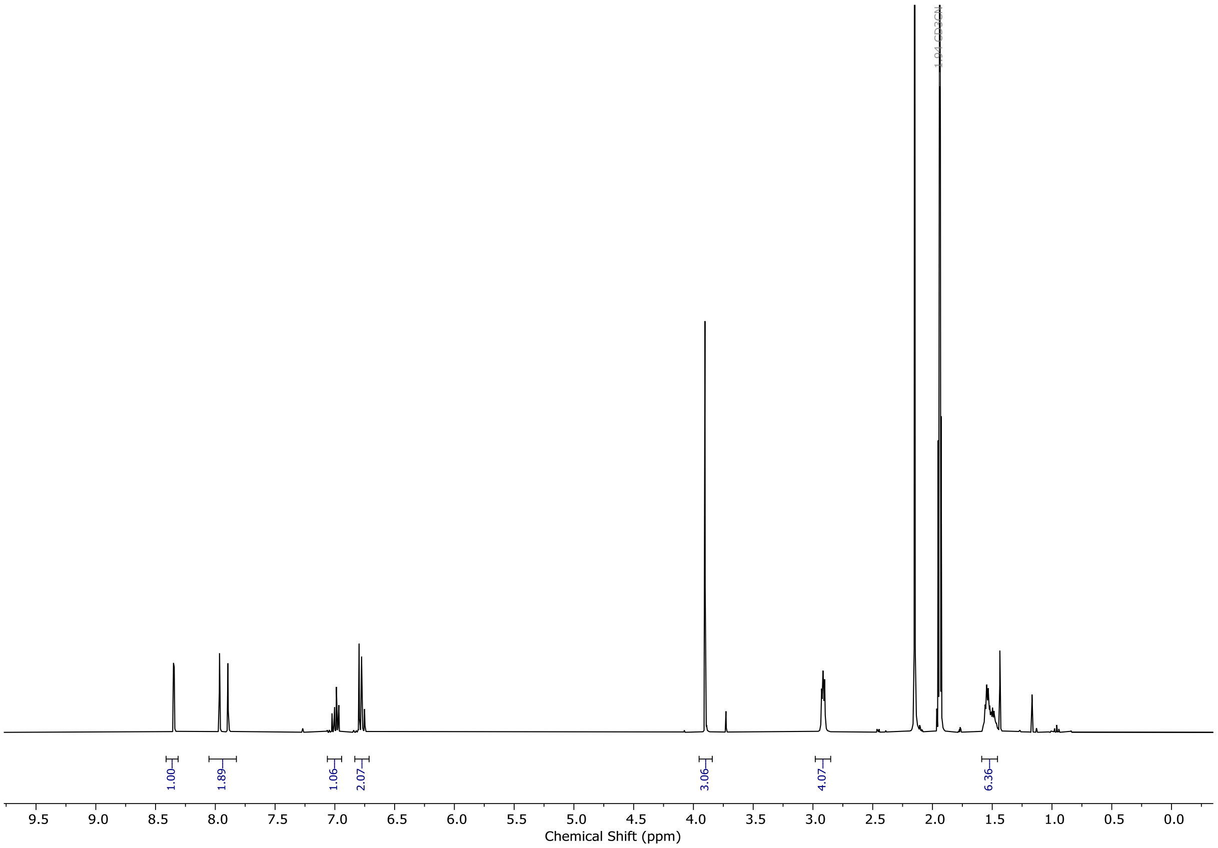

Figure S22. ^{1}H NMR (400 MHz, 298 K, CD_3CN) of **2n**.

3 Photoswitching Properties

This section contains additional information relating to the photoswitching properties of the AIP imines. The data presented in Table S3 was obtained by fitting the UV/vis absorption spectra of the *E*- and *Z*- isomers. The spectra of *E*-isomers used for fitting were measured on the diode array set-up (Figure S1), and spectra of *Z*-isomers were predicted as introduced in Section 4.3 of Supporting Information. Specifically, the peak analyzer tool in OriginPro 2020 was used to fit the UV/vis spectra with the aim of deconvoluting overlapping transitions. The peaks were fitted to Gaussians, and the fit results are presented in Table S3.

Table S3. Summary of the peak wavelengths of the fitted π-π* UV/vis absorption bands and the differences in peak wavelength between the *E*- and *Z*-isomers. The measurements are performed in MeCN at 20°C. The data of imine **1a**, **1e**, and **1f** were taken from our previous work.[1]

	E-Isomer π-π*(nm)	*Z*-Isomer π-π*(nm)	Δπ-π* (*E*-*Z*, nm)
**1a**	320	326	−6
**1b**	324	352	−28
**1c**	361	356	5
**1e**	370	345	26
**1f**	363	310	53
**1g**	352	326	26
**1h**	369	351	18
**1i**	353	325	28
**1j**	338	305	33
**1k**	343	321	22
**1l**	314	319	−5
**1m**	365	347	18
**1n**	336	311	25
**1o**	355	328	27
**2a**	307	288	19
**2b**	323	332	−9
**2c**	337	–	–
**2d**	363	326	37
**2e**	372	337	35
**2f**	367	273	94
**2k**	308	291	17
**2l**	303	297	6
**2m**	366	353	13
**2n**	329	306	23

3.1 Extinction Coefficient

The UV-visible spectra of all imines included in this work were measured using a Jasco V-770 UV-Visible/NIR spectrophotometer at various concentrations. According to Beer-Lambert Law,[4] there is a linear relationship between the absorbance and the concentration of the solution, which can be expressed as:

$$A = \epsilon l c$$

In this equation, "A" represents the absorbance in absorbance units (a.u.), "l" is the optical path length, which is typically the width of the cuvette used for the absorbance measurement (usually 1 cm), and "c" is the molar concentration in moles per liter (M). From the absorption spectra, the molar extinction coefficient (ε) at the specific wavelength (λ_{max}) can be determined by fitting a linear calibration curve of the absorbance versus concentration.

Table S4. The wavelengths of maximum absorbance (λ_{max}) and the extinction coefficients at λ_{max} of each switch. The data of imine **1a**, **1e**, and **1f** were obtained from our previous work.[1]

	**λ_{max}** (nm)	**Extinction Coefficient at λ_{max}** (M^{-1} cm^{-1})
**1a**	315	14210
**1b**	322	19990
**1c**	358	17390
**1e**	370	8310
**1f**	362	6340
**1g**	344	8080
**1h**	343	8110
**1i**	345	8020
**1j**	335	8230
**1k**	320	14490
**1l**	314	13050
**1m**	358	5860
**1n**	310	13090
**1o**	351	10050
**2a**	300	11450
**2b**	319	13780
**2c**	337	18450
**2d**	353	15570
**2e**	371	4470
**2f**	360	1730
**2k**	304	7560
**2l**	300	9450
**2m**	361	3810
**2n**	325	3340

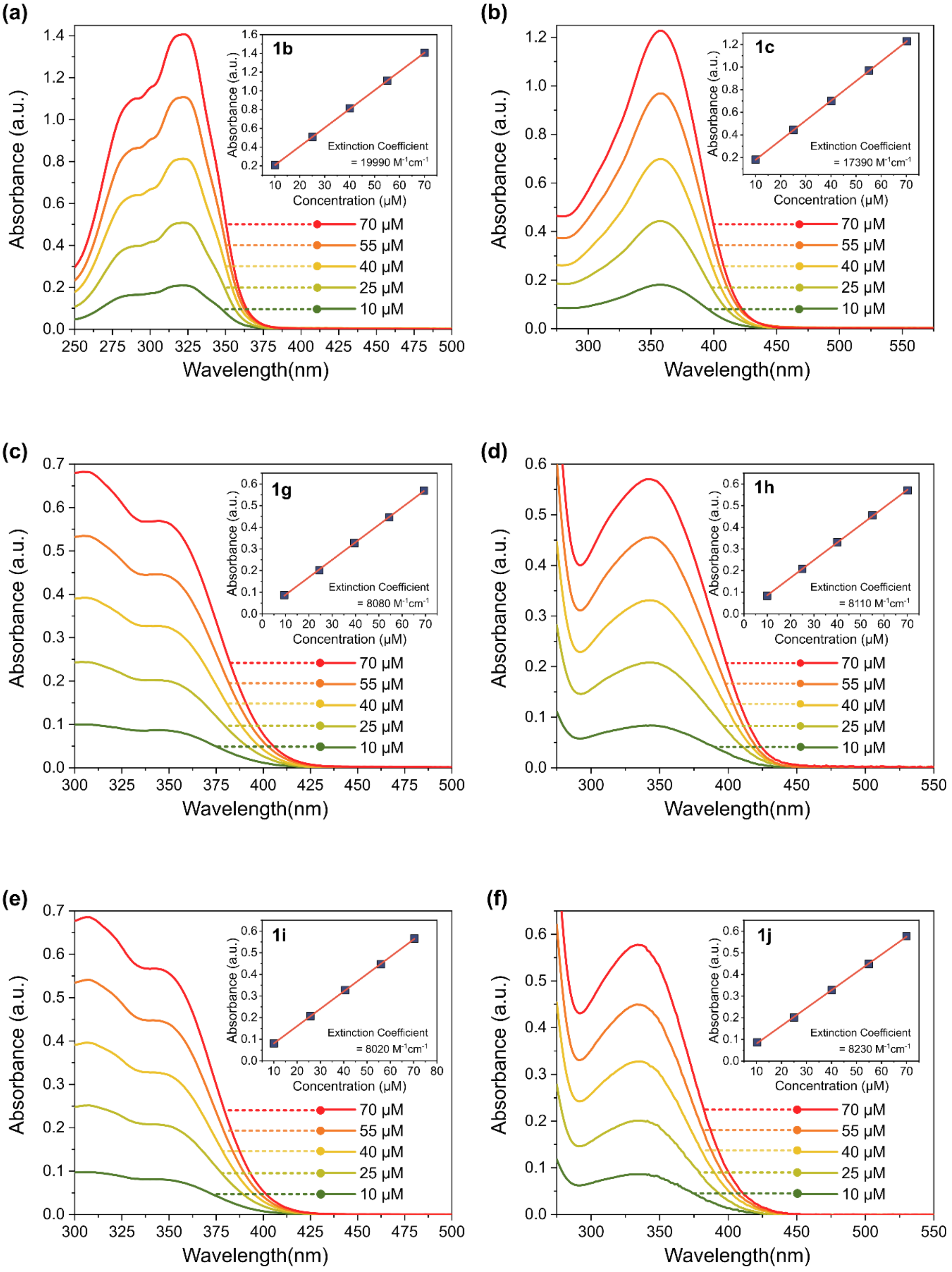

Figure S23. The UV-visible absorption spectra of imines **1b**, **1c**, **1g**, **1h**, **1i**, and **1j** were measured at 20°C in acetonitrile, each at different concentrations. The calibration curves, shown in the top right corner of each figure, were plotted at the wavelength of λ_{max} to determine the slopes, which represent the extinction coefficients.

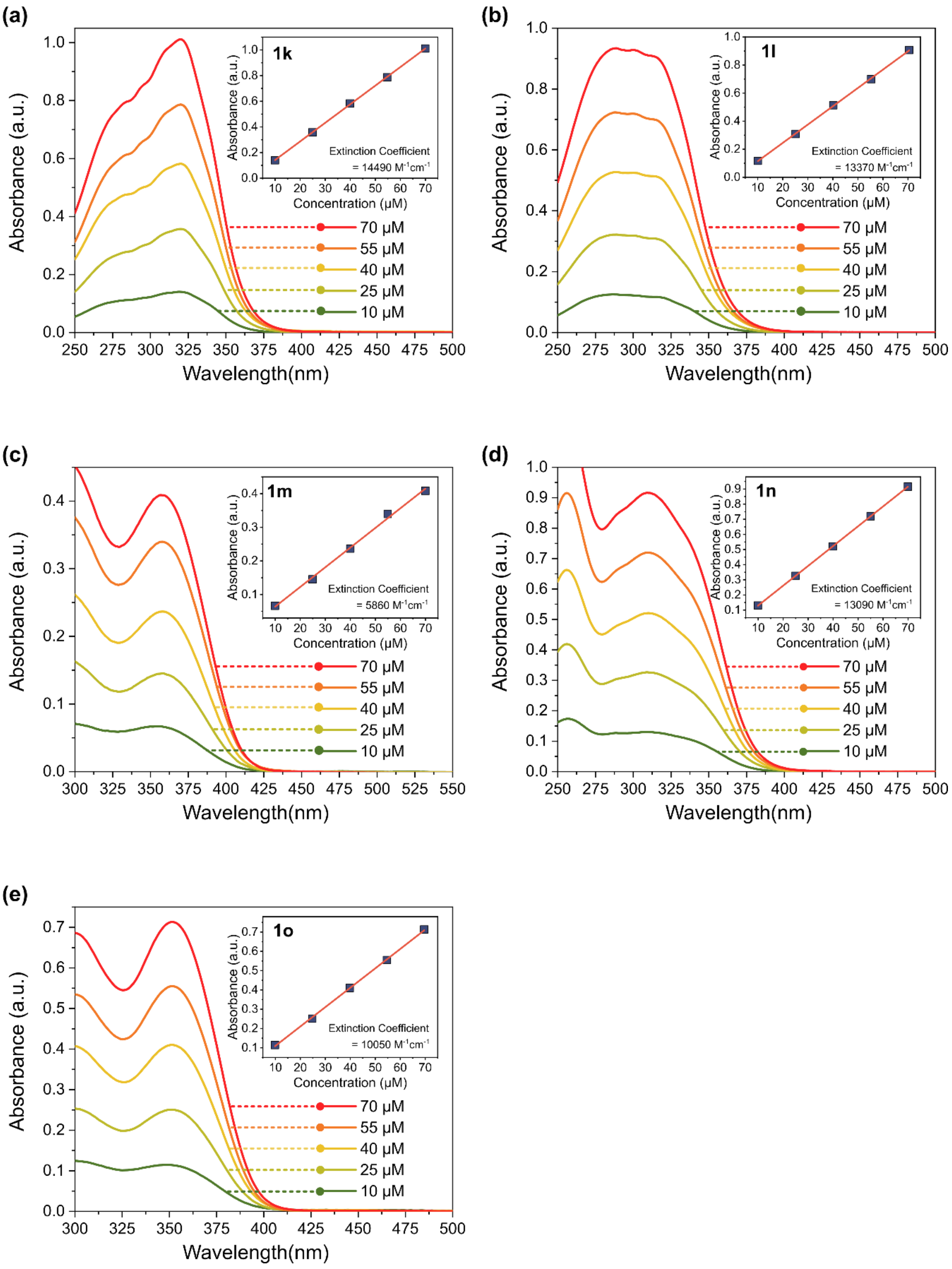

Figure S24. The UV-visible absorption spectra of imines **1k**, **1l**, **1m**, **1n**, and **1o** were measured at 20°C in acetonitrile, each at different concentrations. The calibration curves, shown in the top right corner of each figure, were plotted at the wavelength of λ_{max} to determine the slopes, which represent the extinction coefficients.

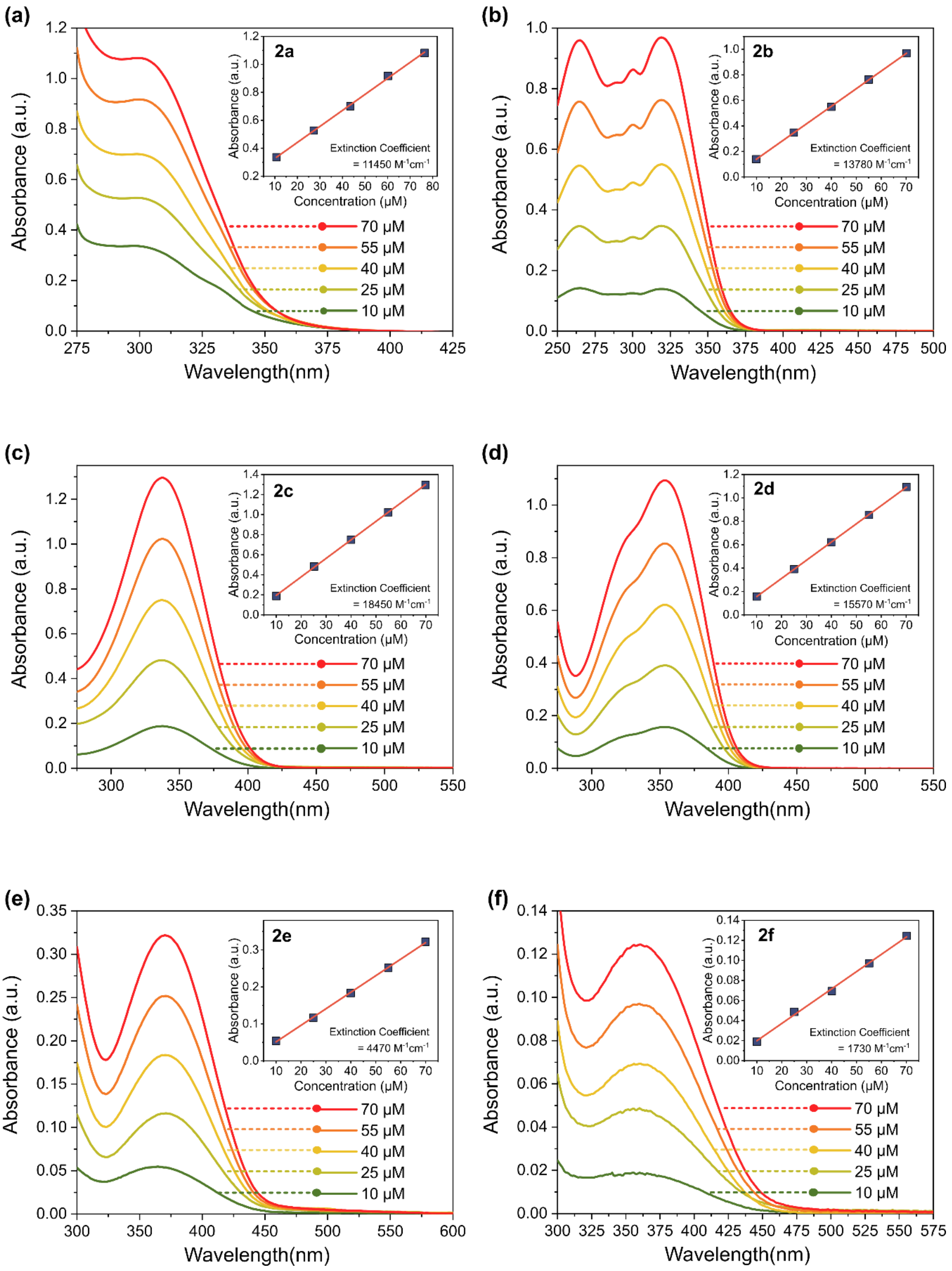

Figure S25. The UV-visible absorption spectra of imines **2a**, **2b**, **2c**, **2d**, **2e**, and **2f** were measured at 20°C in acetonitrile, each at different concentrations. The calibration curves, shown in the top right corner of each figure, were plotted at the wavelength of λ_{max} to determine the slopes, which represent the extinction coefficients.

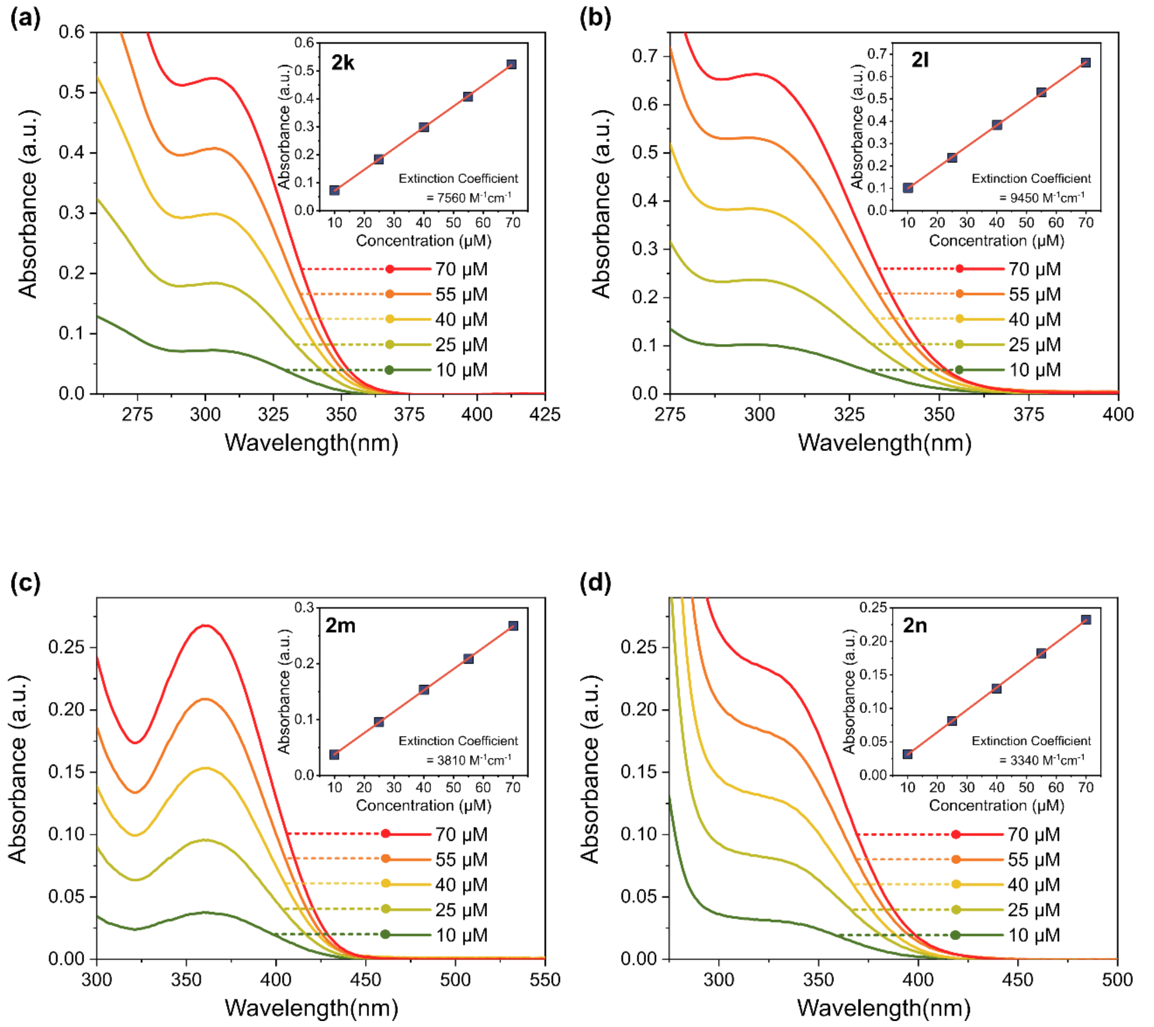

Figure S26. The UV-visible absorption spectra of imines **2k, 2l**, **2m**, and **2n** were measured at 20°C in acetonitrile, each at different concentrations. The calibration curves, shown in the top right corner of each figure, were plotted at the wavelength of λ_{max} to determine the slopes, which represent the extinction coefficients.

3.2 Thermal Dynamic Properties

The kinetics of all imine switches included in this work were investigated using the diode array setup (introduced in Section 1). These measurements were conducted in the solution state using acetonitrile as the solvent, and a range of temperatures was employed for linear Eyring plots. The isomerization from *E*-isomers to *Z*-isomers was induced by irradiation with 365 nm LED light. Assuming first-order reactions, the rate constants (k) for the thermodynamic switching process from *Z*- to *E*-state were determined by fitting the absorbance changes over time to an exponential, and the thermal half-lives ($t_{1/2}$) were calculated based on this rate constants. Notably, all thermal half-lives presented in this work were performed at 20 °C.

Utilizing the linearized form of the Eyring equation[5] presented below:

$$ln\ \frac{k}{T} = \frac{-\Delta H^{\ddagger}}{R} \cdot \frac{1}{T} + ln\ \frac{k_B}{h} + \frac{\Delta S^{\ddagger}}{R}$$

The values for enthalpy of activation ($\Delta H^{\ddagger}$) and entropy of activation ($\Delta S^{\ddagger}$) in Table S5 can be determined from the kinetic data at different temperatures by linear fitting. Gibbs energy of activation (listed in Table S5) can be calculated using the Gibbs function:

$$\Delta G^{\ddagger} = \Delta H^{\ddagger} - T\Delta S^{\ddagger}$$

Table S5. The summary of activation parameters including thermal half-life, enthalpy of activation (ΔH‡), entropy of activation (ΔS‡), and Gibbs energy of activation (ΔG‡) data of imines discussed in this work. The data of imine **1a**, **1e**, and **1f** were obtained from our previous work.[1] In this table, the Gibbs energy of activation corresponds to a temperature of 295K.

	$t_{1/2}$	**ΔH‡** ($kJ•mol^{-1}$)	**ΔS‡** ($J•K^{-1}•mol^{-1}$)	**ΔG‡** ($kJ•mol^{-1}$)
**1a**	12.5 s	82.68	13.41	78.73
**1b**	15.9 s	82.94	12.29	79.32
**1c**	4.2 s	78.78	9.34	76.03
**1e**	22.1 min	86.27	–13.25	90.18
**1f**	19.2 h	105.08	17.56	99.9
**1g**	6.3 min	83.86	–10.78	87.04
**1h**	4.7 h	91.92	–15.03	96.36
**1i**	5.4 min	83.14	–11.92	86.66
**1j**	25.9 h	99.71	–2.92	100.57
**1k**	44.4 s	80.27	–5.48	81.89
**1l**	6.8 min	85.47	–6.07	87.26
**1m**	5.5 h	92.50	–14.50	96.77
**1n**	1.2 h	88.29	–16.24	93.08
**1o**	4.9 h	93.43	–10.59	96.56
**2a**	1.1 min	87.40	16.08	82.65
**2b**	12.2 min	86.10	–8.71	88.67
**2c**	< 0.1 s	-	-	-
**2d**	12.9 min	75.31	–46.45	89.02
**2e**	1.4 min	80.92	–8.50	83.42
**2f**	20.03 s	72.57	–24.40	79.77
**2k**	54.5 s	84.05	5.71	82.37
**2l**	52.3 s	78.81	–12.24	82.42
**2m**	1.4 min	86.54	10.70	83.39
**2n**	21.5 s	79.34	–2.63	80.12

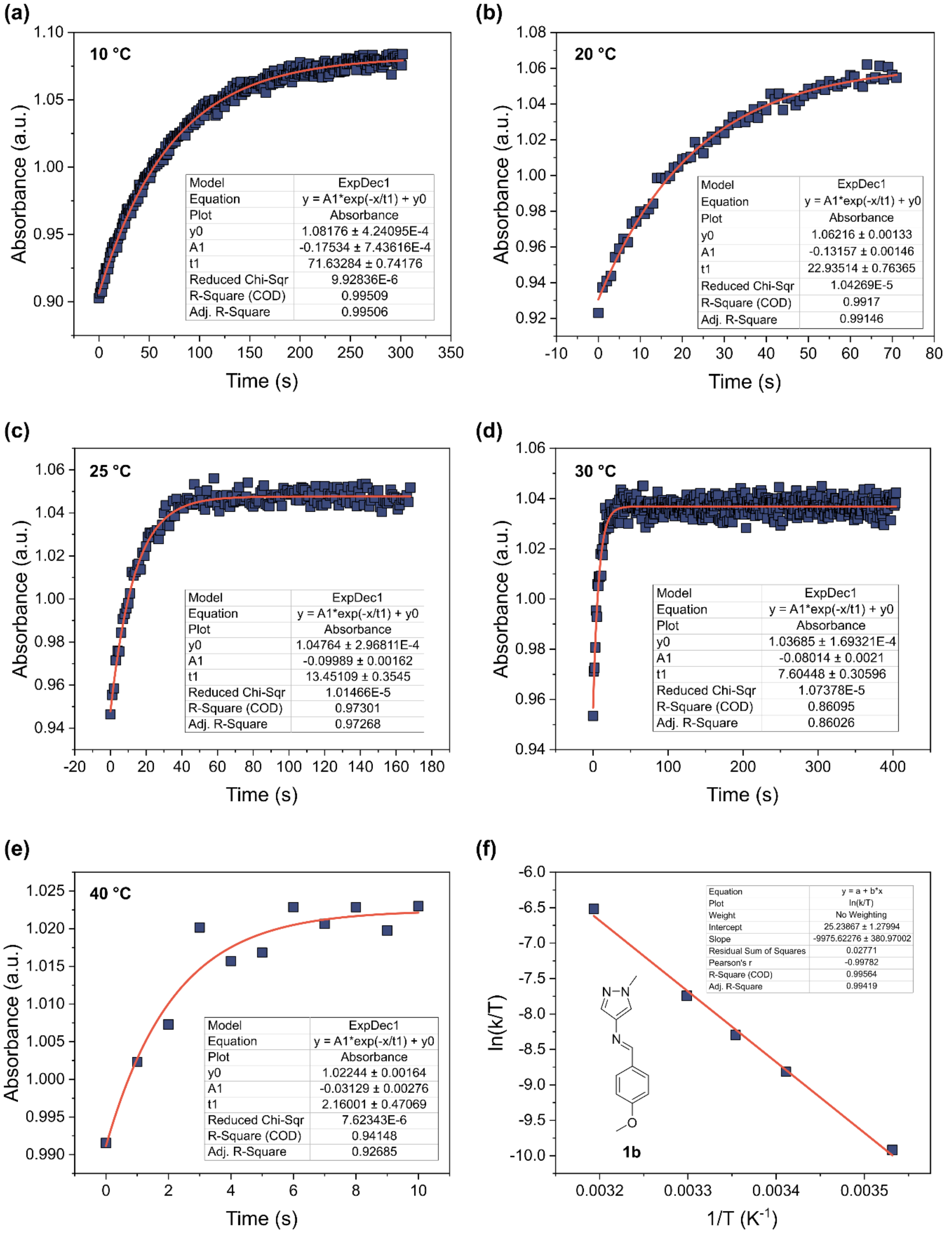

Figure S27. The change in absorbance of **1b** at the λ_{max} of the *E*-isomer over time at **a**) 10 °C, **b**) 20 °C, **c**) 25 °C, **d**) 30 °C, **e**) 40 °C with the increase in absorbance relating to the thermal isomerization of the *Z*-isomer to the *E*-isomer. All samples were irradiated under 365 nm LED for 5 minutes in acetonitrile before the measurements started. The exponential fittings were applied to determine the rate constants and, thus, thermal half-lives. **f**) The Eyring plots were generated using the rate constants calculated at different temperatures, and the fitted parameter was used to estimate the enthalpy of activation ($\Delta H^{\ddagger}$), entropy of activation ($\Delta S^{\ddagger}$), and Gibbs energy of activation ($\Delta G^{\ddagger}$) listed in Table S5.

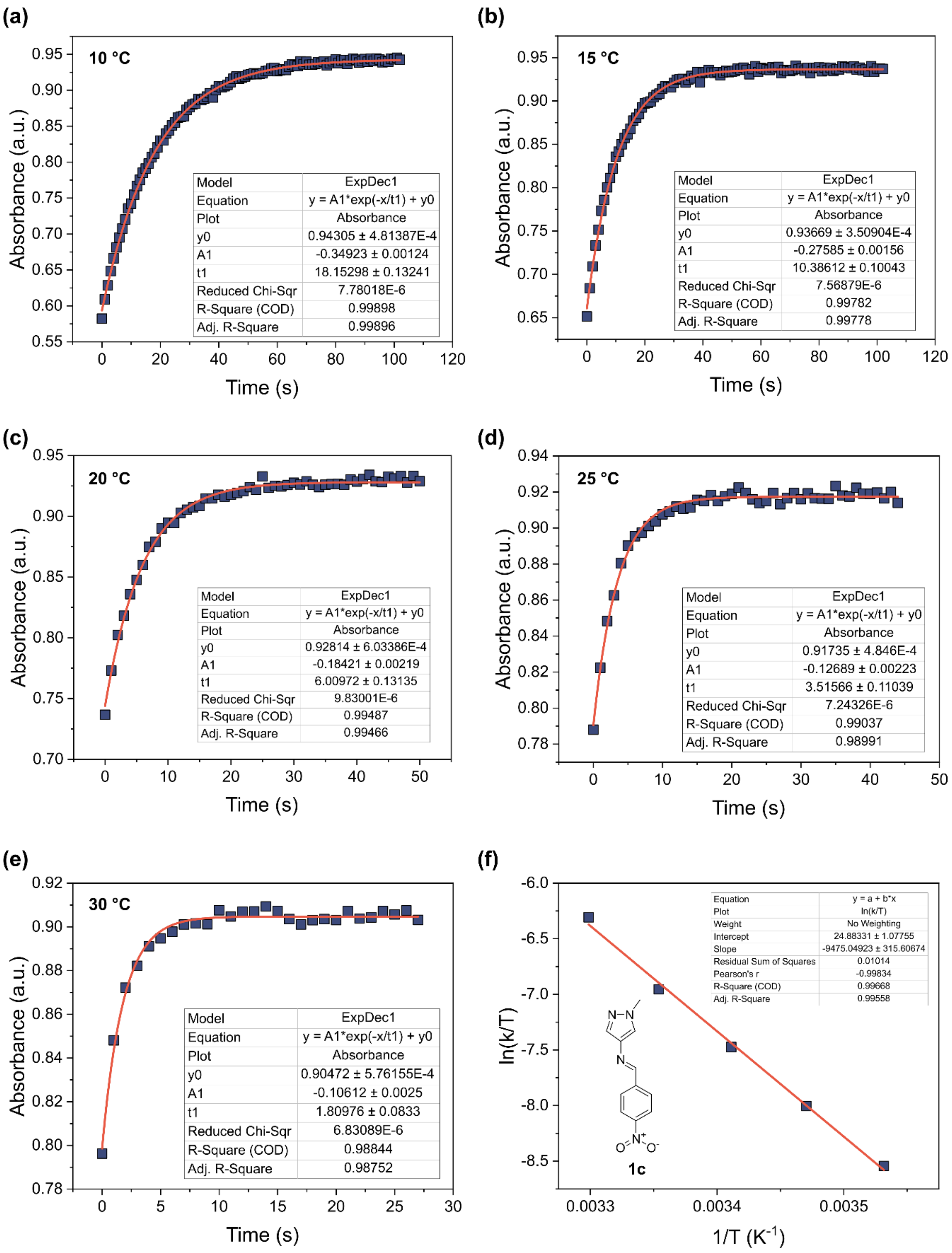

Figure S28. The change in absorbance of **1c** at the λ_{max} of the *E*-isomer over time at **a**) 10 °C, **b**) 15 °C, **c**) 20 °C, **d**) 25 °C, **e**) 30 °C with the increase in absorbance relating to the thermal isomerization of the *Z*-isomer to the *E*-isomer. All samples were irradiated under 365 nm LED for 5 minutes in acetonitrile before the measurements started. The exponential fittings were applied to determine the rate constants and thus thermal half-lives. **f**) The Eyring plots were generated using the rate constants calculated at different temperatures, and the fitted parameter was used to estimate the enthalpy of activation ($\Delta H^‡$), entropy of activation ($\Delta S^‡$), and Gibbs energy of activation ($\Delta G^‡$) listed in Table S5.

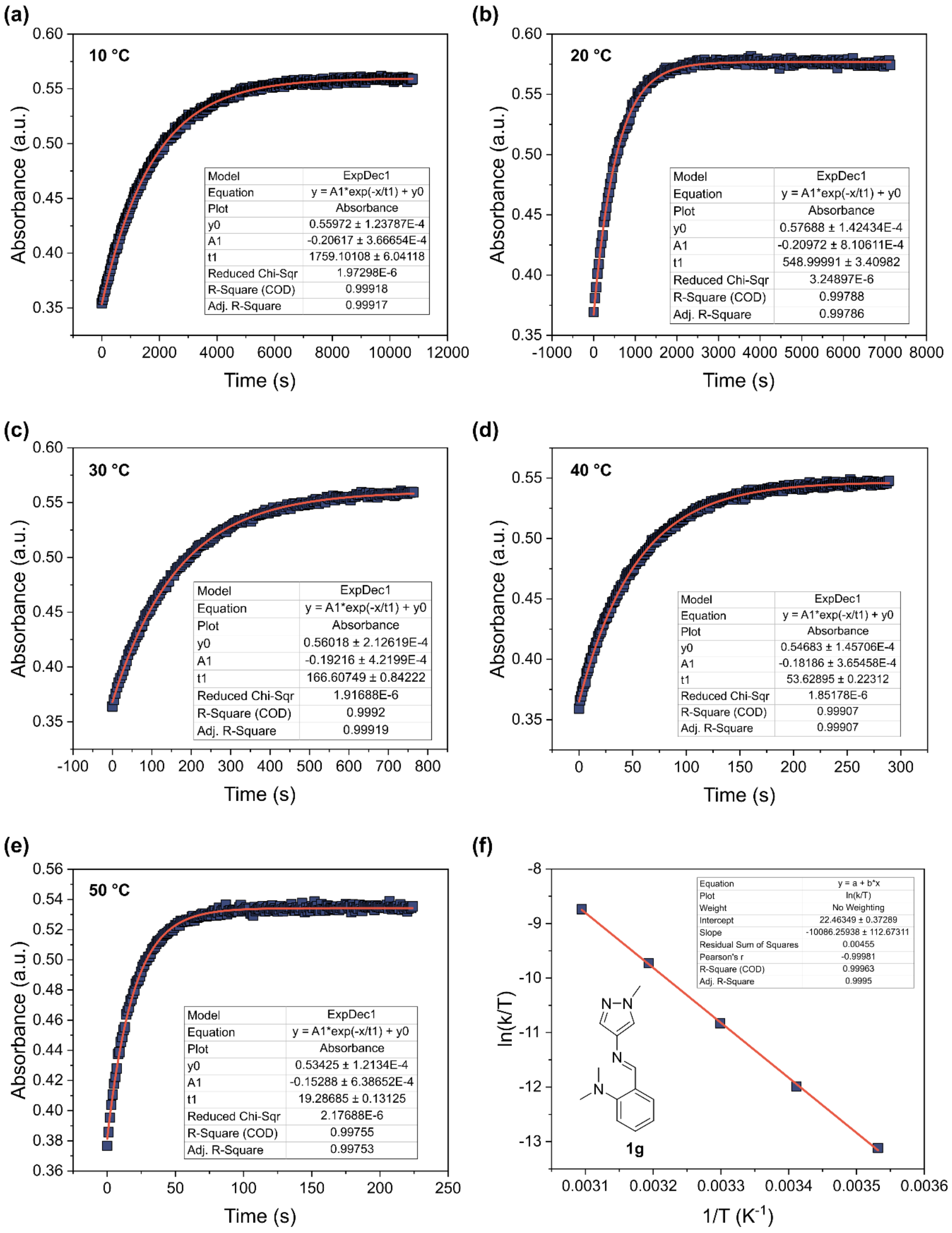

Figure S29. The change in absorbance of **1g** at the λ_{max} of the *E*-isomer over time at **a**) 10 °C, **b**) 20 °C, **c**) 30 °C, **d**) 40 °C, **e**) 50 °C with the increase in absorbance relating to the thermal isomerization of the *Z*-isomer to the *E*-isomer. All samples were irradiated under 365 nm LED for 5 minutes in acetonitrile before the measurements started. The exponential fittings were applied to determine the rate constants and thus thermal half-lives. **f**) The Eyring plots were generated using the rate constants calculated at different temperatures, and the fitted parameter was used to estimate the enthalpy of activation ($\Delta H^‡$), entropy of activation ($\Delta S^‡$), and Gibbs energy of activation ($\Delta G^‡$) listed in Table S5.

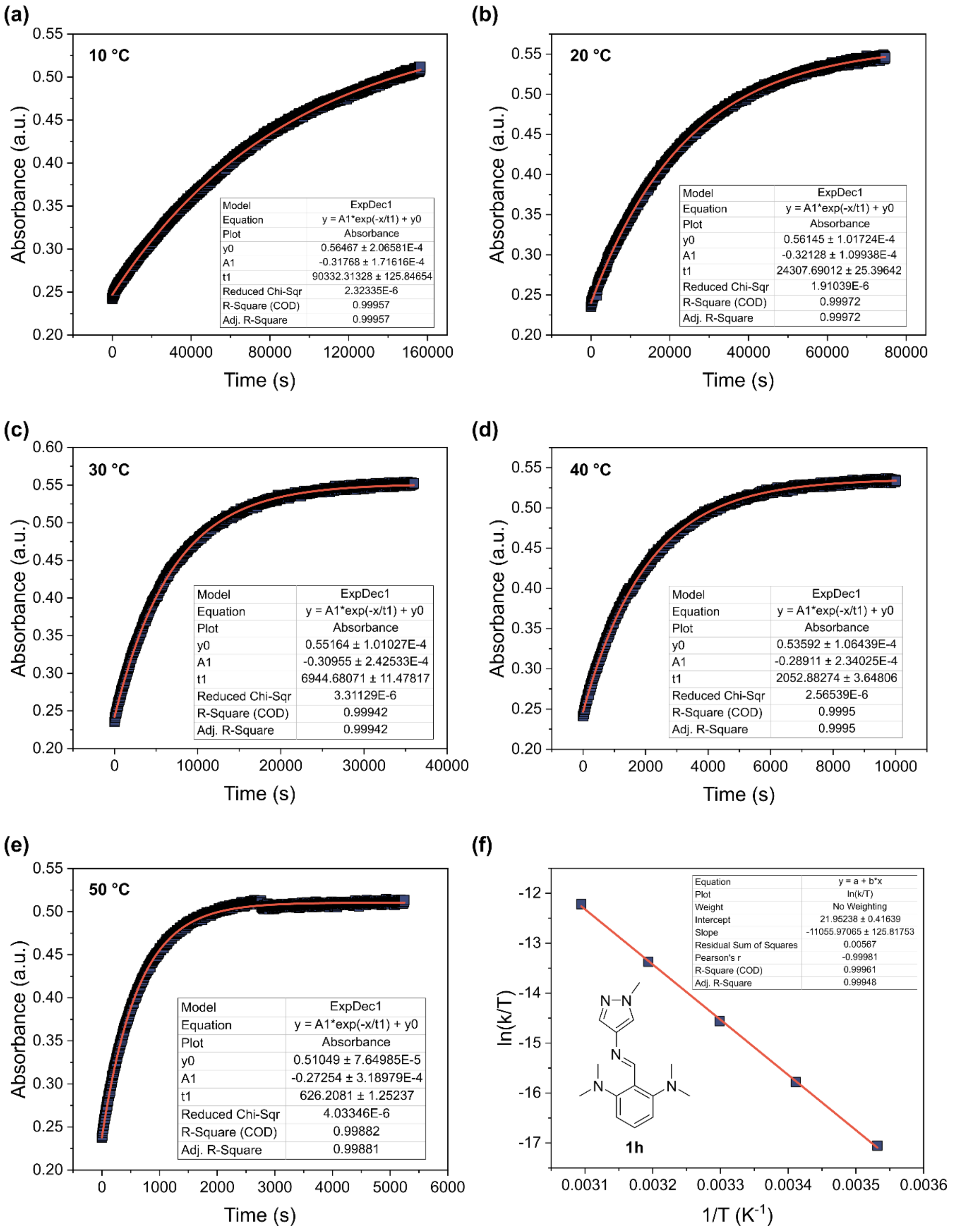

Figure S30. The change in absorbance of **1h** at the λ_{max} of the *E*-isomer over time at **a**) 10 °C, **b**) 20 °C, **c**) 30 °C, **d**) 40 °C, **e**) 50 °C with the increase in absorbance relating to the thermal isomerization of the *Z*-isomer to the *E*-isomer. All samples were irradiated under 365 nm LED for 5 minutes in acetonitrile before the measurements started. The exponential fittings were applied to determine the rate constants and thus thermal half-lives. **f**) The Eyring plots were generated using the rate constants calculated at different temperatures, and the fitted parameter was used to estimate the enthalpy of activation ($\Delta H^{\ddagger}$), entropy of activation ($\Delta S^{\ddagger}$), and Gibbs energy of activation ($\Delta G^{\ddagger}$) listed in Table S5.

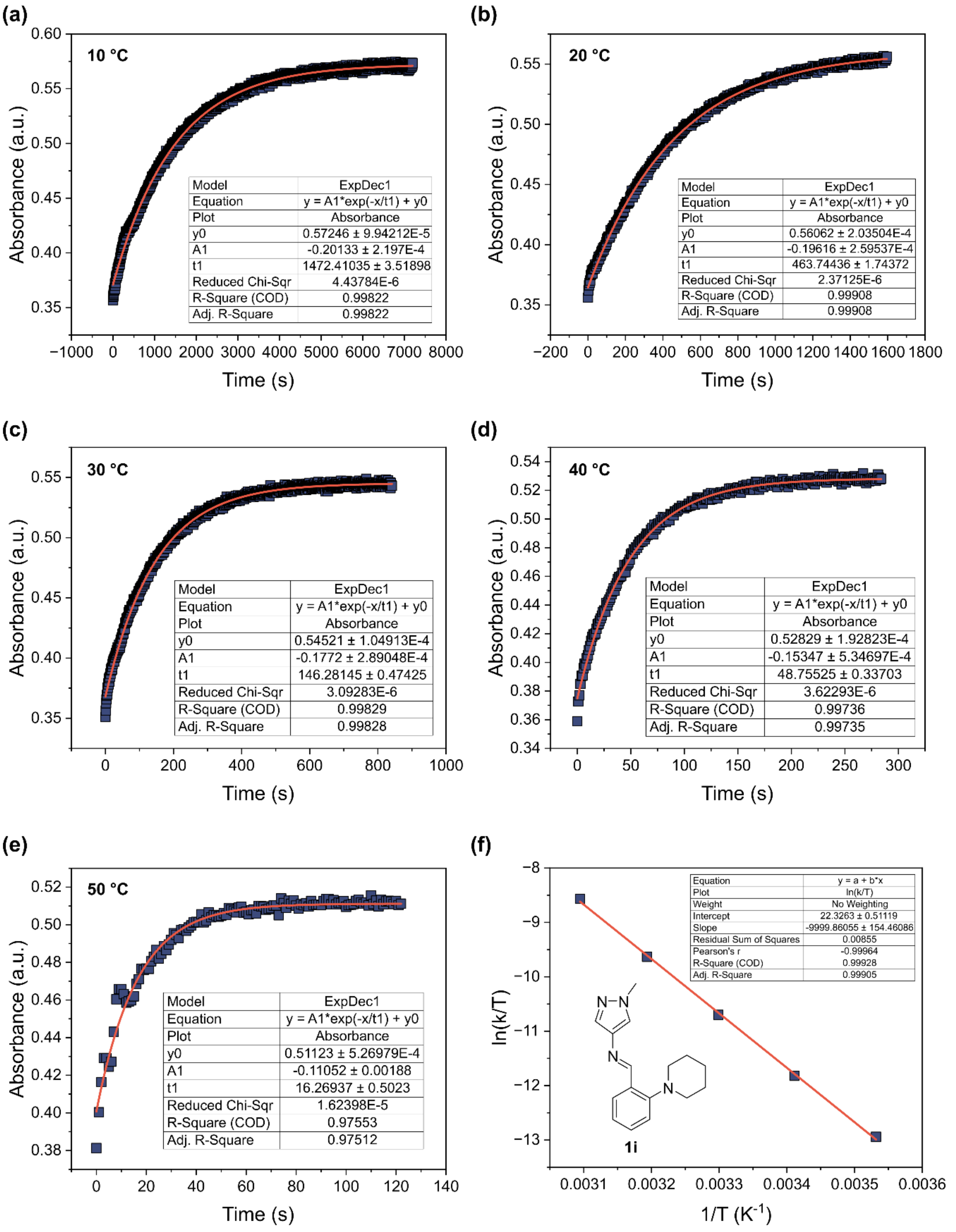

Figure S31. The change in absorbance of **1i** at the λ_{max} of the *E*-isomer over time at **a**) 10 °C, **b**) 20 °C, **c**) 30 °C, **d**) 40 °C, **e**) 50 °C with the increase in absorbance relating to the thermal isomerization of the *Z*-isomer to the *E*-isomer. All samples were irradiated under 365 nm LED for 5 minutes in acetonitrile before the measurements started. The exponential fittings were applied to determine the rate constants and thus thermal half-lives. **f**) The Eyring plots were generated using the rate constants calculated at different temperatures, and the fitted parameter was used to estimate the enthalpy of activation ($\Delta H^{‡}$), entropy of activation ($\Delta S^{‡}$), and Gibbs energy of activation ($\Delta G^{‡}$) listed in Table S5.

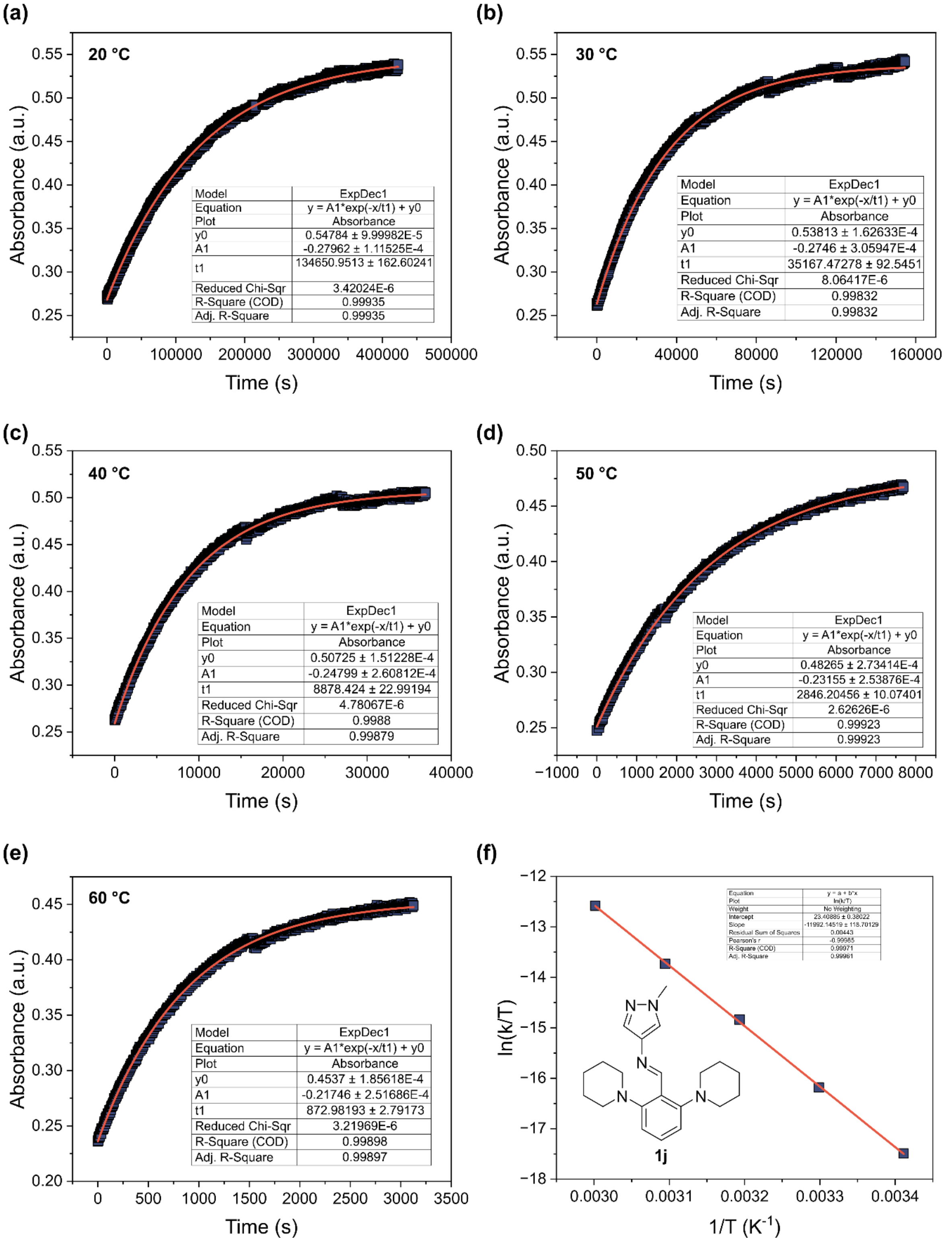

Figure S32. The change in absorbance of **1j** at the λ_{max} of the *E*-isomer over time at **a**) 20 °C, **b**) 30 °C, **c**) 40 °C, **d**) 50 °C, **e**) 60 °C with the increase in absorbance relating to the thermal isomerization of the *Z*-isomer to the *E*-isomer. All samples were irradiated under 365 nm LED for 5 minutes in acetonitrile before the measurements started. The exponential fittings were applied to determine the rate constants and, thus, thermal half-lives. **f**) The Eyring plots were generated using the rate constants calculated at different temperatures, and the fitted parameter was used to enthalpy of activation ($\Delta H^{\ddagger}$), entropy of activation ($\Delta S^{\ddagger}$), and Gibbs energy of activation ($\Delta G^{\ddagger}$) listed in Table S5.

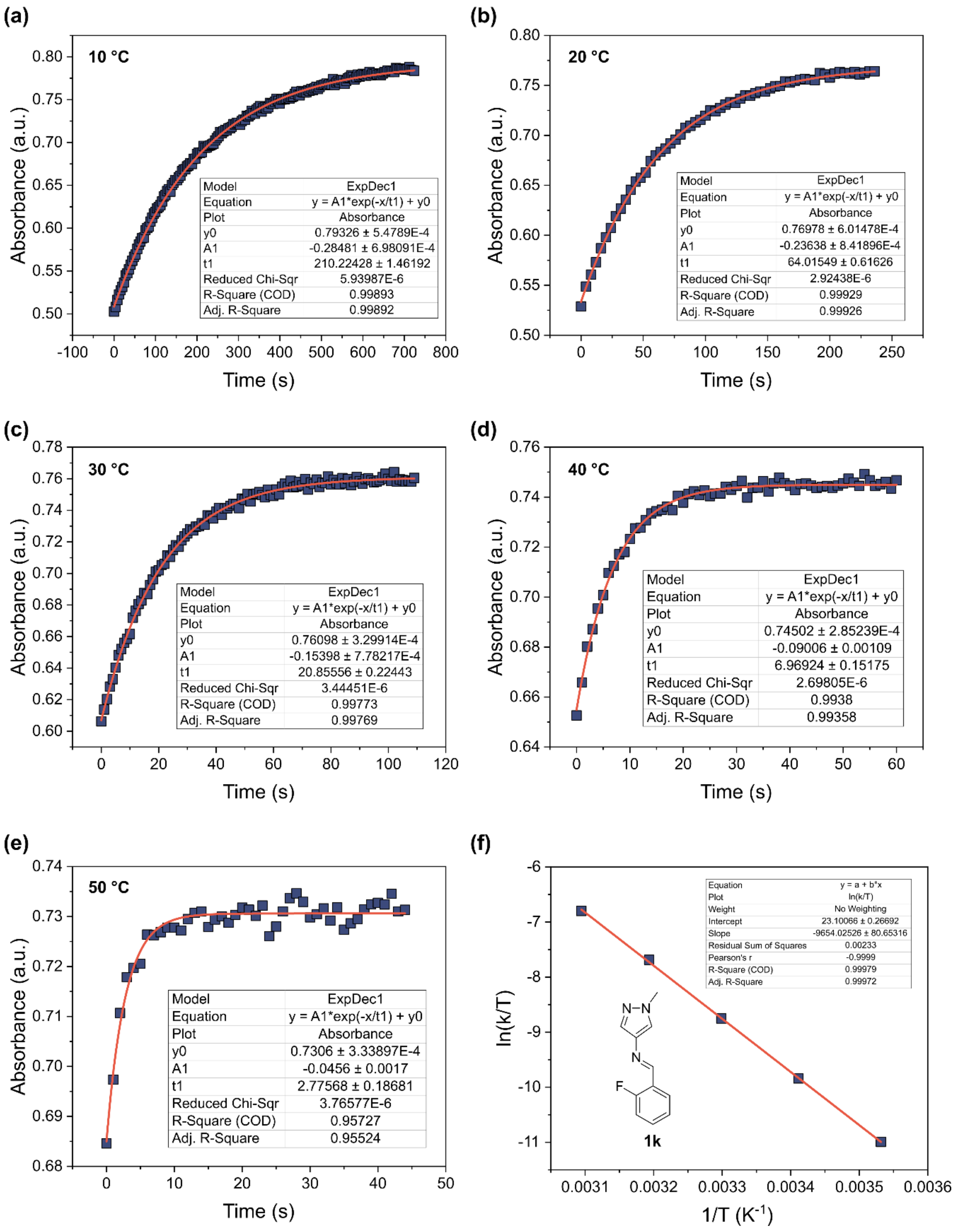

Figure S33. The change in absorbance of **1k** at the λ_{max} of the *E*-isomer over time at **a**) 10 °C, **b**) 20 °C, **c**) 30 °C, **d**) 40 °C, **e**) 50 °C with the increase in absorbance relating to the thermal isomerization of the *Z*-isomer to the *E*-isomer. All samples were irradiated under 365 nm LED for 5 minutes in acetonitrile before the measurements started. The exponential fittings were applied to determine the rate constants and thus thermal half-lives. **f**) The Eyring plots were generated using the rate constants calculated at different temperatures, and the fitted parameter was used to estimate the enthalpy of activation ($\Delta H^{\ddagger}$), entropy of activation ($\Delta S^{\ddagger}$), and Gibbs energy of activation ($\Delta G^{\ddagger}$) listed in Table S5.

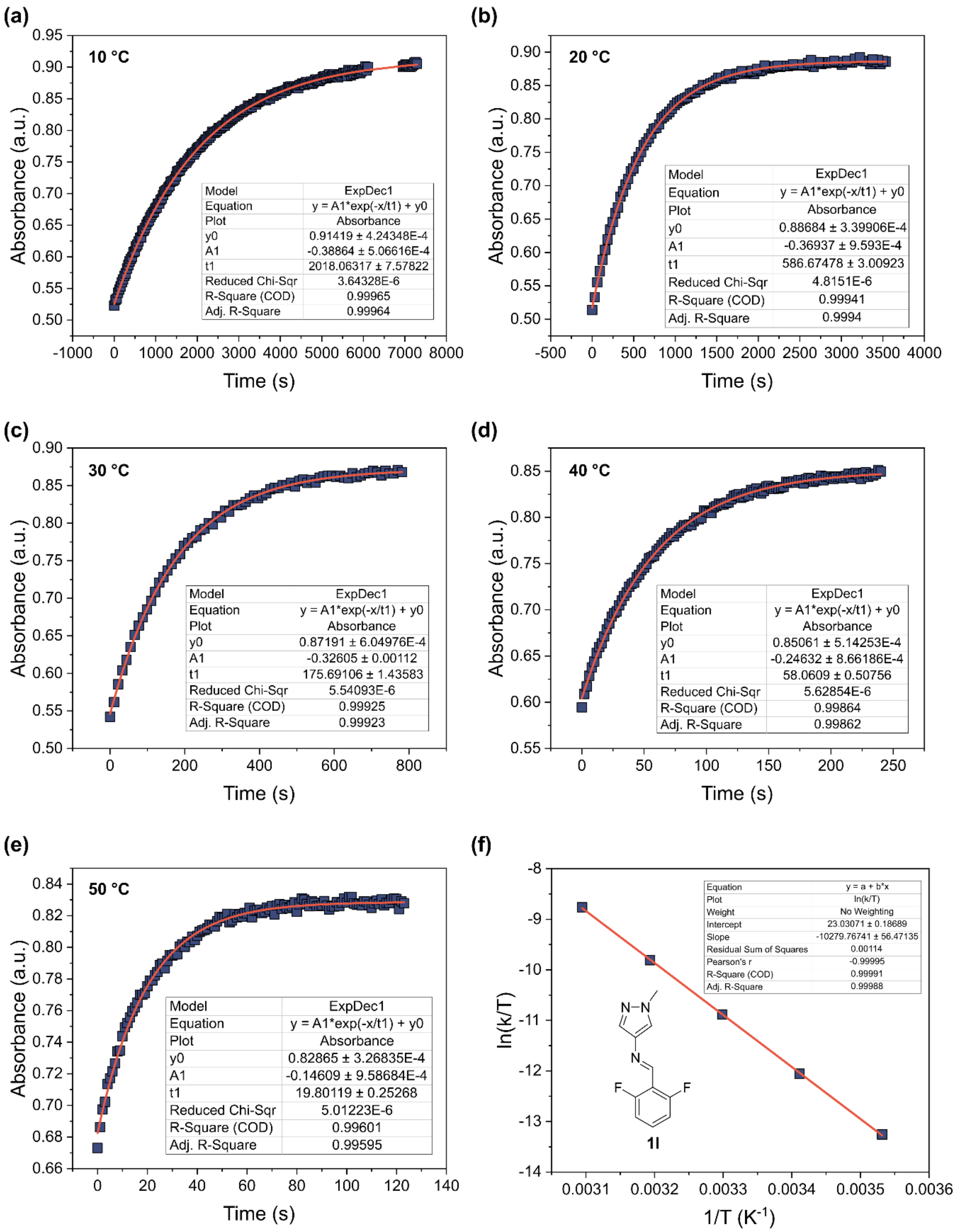

Figure S34. The change in absorbance of **1l** at the λ_{max} of the *E*-isomer over time at **a**) 10 °C, **b**) 20 °C, **c**) 30 °C, **d**) 40 °C, **e**) 50 °C with the increase in absorbance relating to the thermal isomerization of the *Z*-isomer to the *E*-isomer. All samples were irradiated under 365 nm LED for 5 minutes in acetonitrile before the measurements started. The exponential fittings were applied to determine the rate constants and thus thermal half-lives. **f**) The Eyring plots were generated using the rate constants calculated at different temperatures, and the fitted parameter was used to estimate the enthalpy of activation ($\Delta H^‡$), entropy of activation ($\Delta S^‡$), and Gibbs energy of activation ($\Delta G^‡$) listed in Table S5.

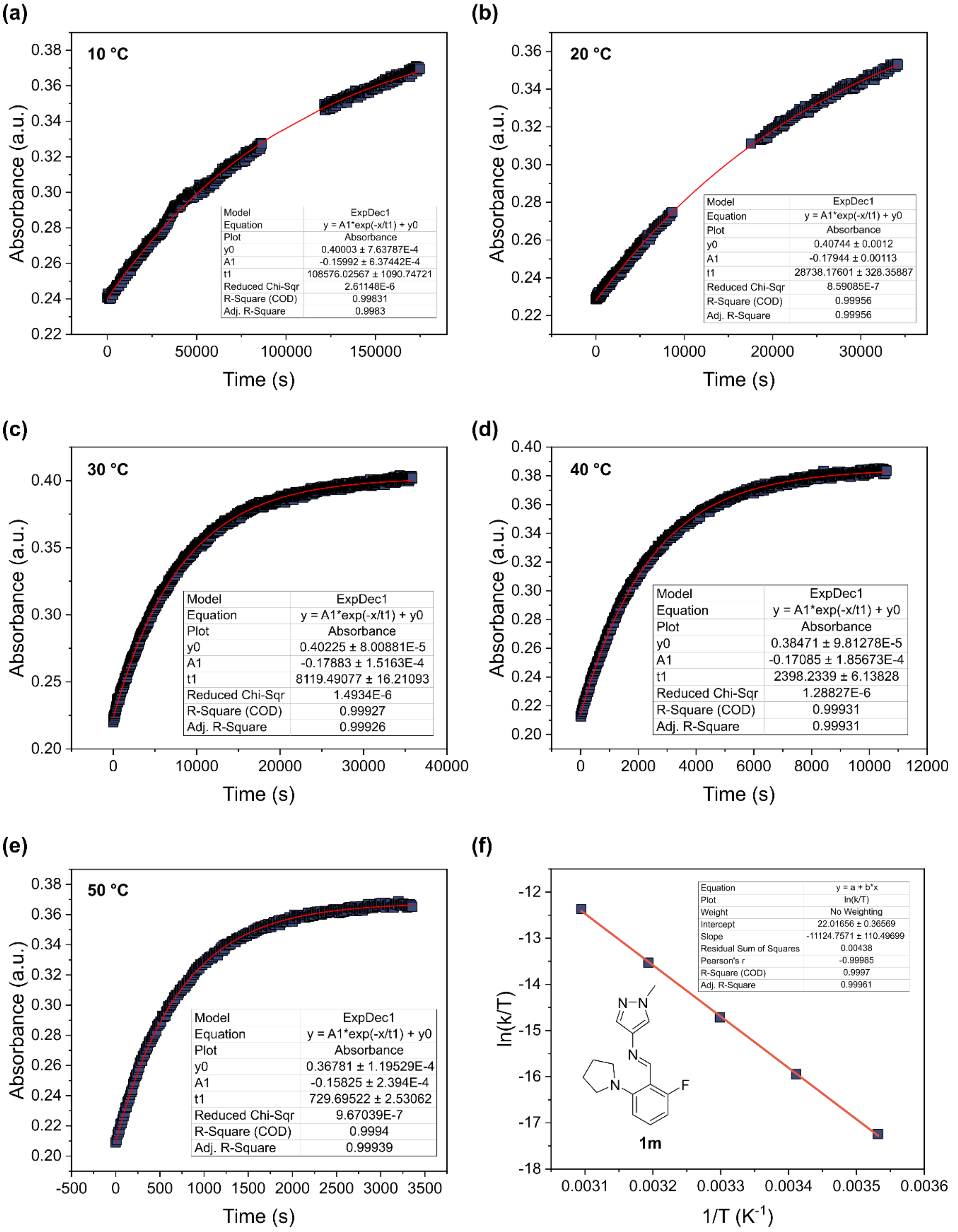

Figure S35. The change in absorbance of **1m** at the λ_{max} of the *E*-isomer over time at **a**) 10 °C, **b**) 20 °C, **c**) 30 °C, **d**) 40 °C, **e**) 50 °C with the increase in absorbance relating to the thermal isomerization of the *Z*-isomer to the *E*-isomer. All samples were irradiated under 365 nm LED for 5 minutes in acetonitrile before the measurements started. The exponential fittings were applied to determine the rate constants and thus thermal half-lives. **f**) The Eyring plots were generated using the rate constants calculated at different temperatures, and the fitted parameter was used to estimate the enthalpy of activation ($\Delta H^{\ddagger}$), entropy of activation ($\Delta S^{\ddagger}$), and Gibbs energy of activation ($\Delta G^{\ddagger}$) listed in Table S5.

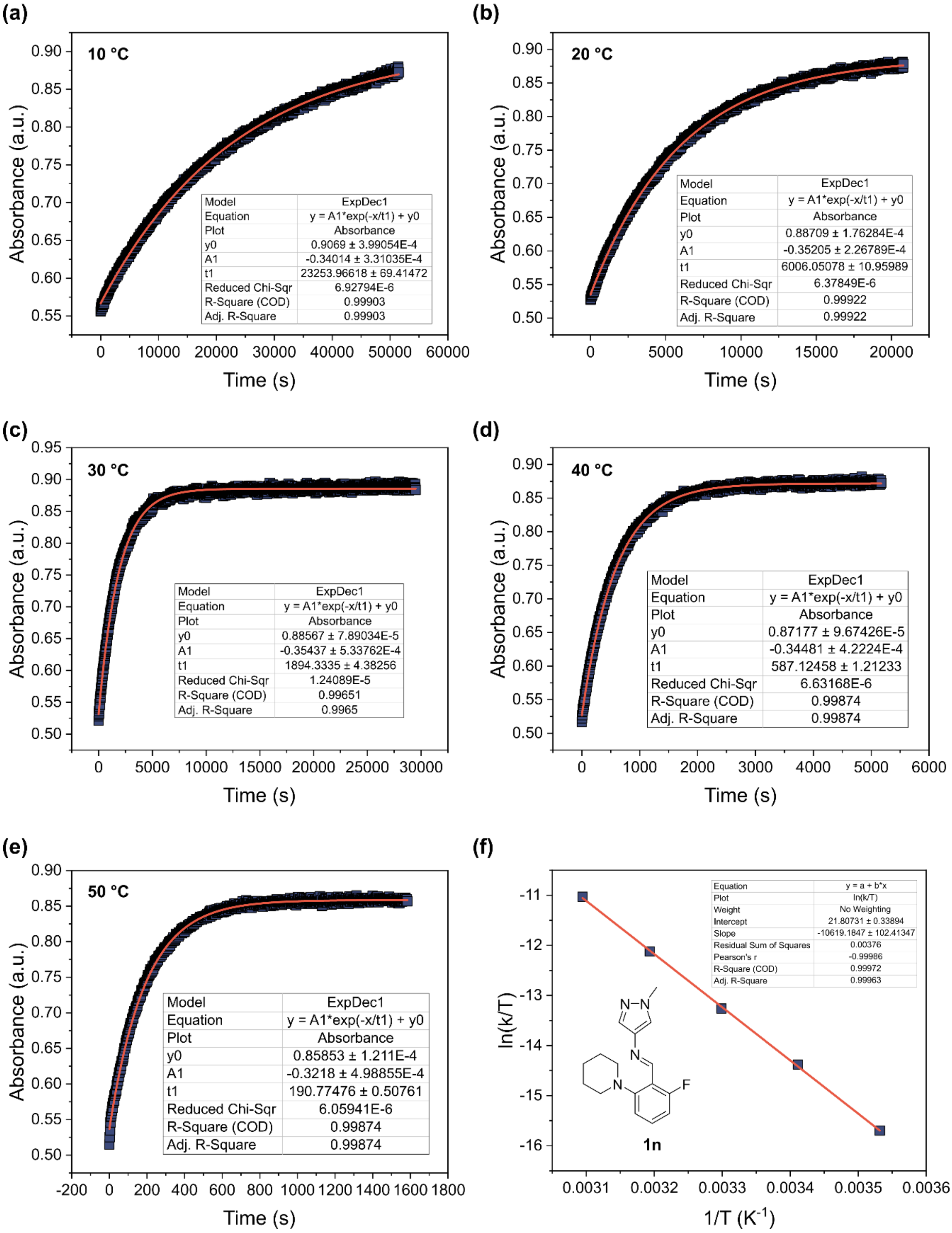

Figure S36. The change in absorbance of **1n** at the λ_{max} of the *E*-isomer over time at **a**) 10 °C, **b**) 20 °C, **c**) 30 °C, **d**) 40 °C, **e**) 50 °C with the increase in absorbance relating to the thermal isomerization of the *Z*-isomer to the *E*-isomer. All samples were irradiated under 365 nm LED for 5 minutes in acetonitrile before the measurements started. The exponential fittings were applied to determine the rate constants and thus thermal half-lives. **f**) The Eyring plots were generated using the rate constants calculated at different temperatures, and the fitted parameter was used to estimate the enthalpy of activation ($\Delta H^{\ddagger}$), entropy of activation ($\Delta S^{\ddagger}$), and Gibbs energy of activation ($\Delta G^{\ddagger}$) listed in Table S5.

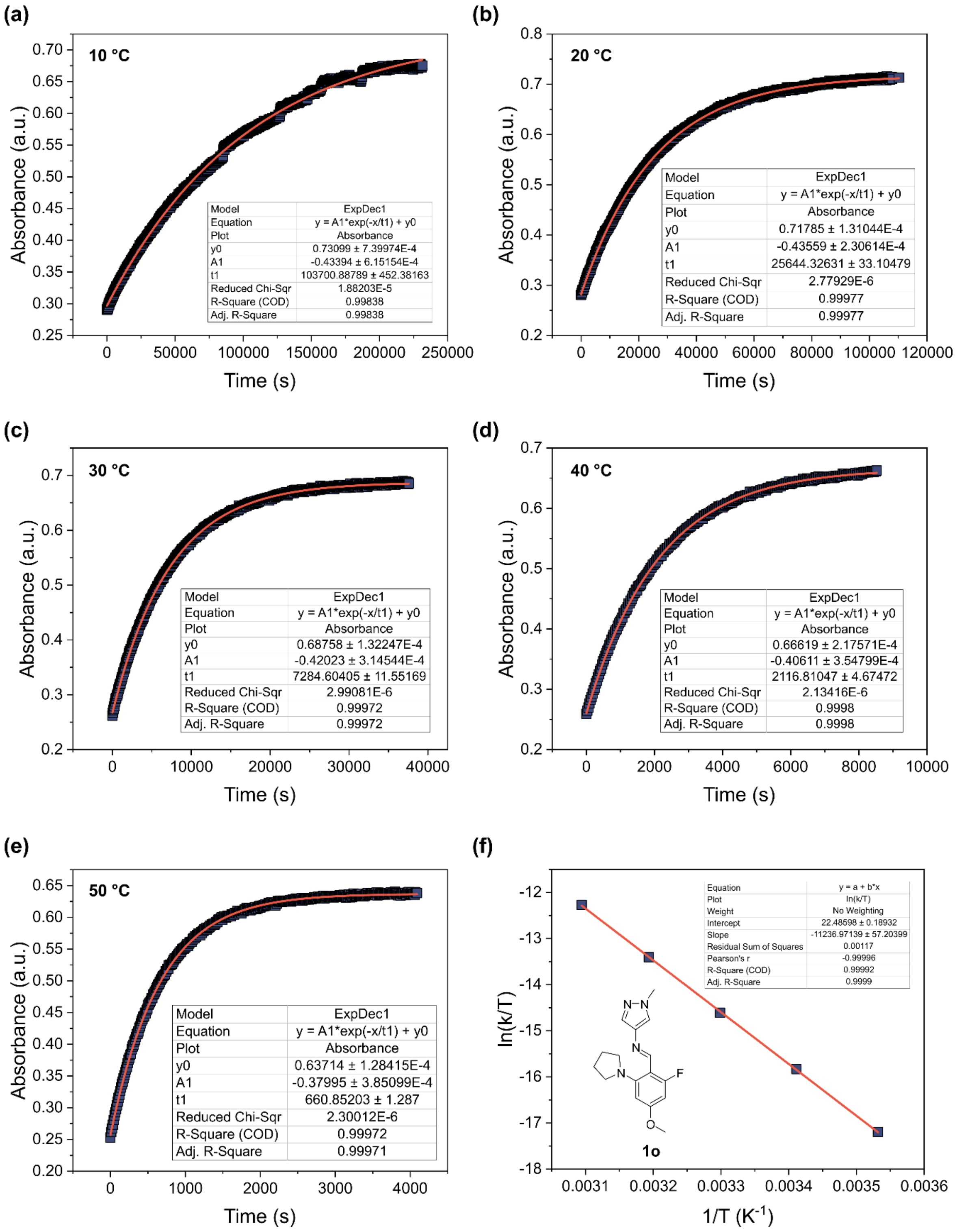

Figure S37. The change in absorbance of **1o** at the λ_{max} of the *E*-isomer over time at **a**) 10 °C, **b**) 20 °C, **c**) 30 °C, **d**) 40 °C, **e**) 50 °C with the increase in absorbance relating to the thermal isomerization of the *Z*-isomer to the *E*-isomer. All samples were irradiated under 365 nm LED for 5 minutes in acetonitrile before the measurements started. The exponential fittings were applied to determine the rate constants and thus thermal half-lives. **f**) The Eyring plots were generated using the rate constants calculated at different temperatures, and the fitted parameter was used to estimate the enthalpy of activation ($\Delta H^{\ddagger}$), entropy of activation ($\Delta S^{\ddagger}$), and Gibbs energy of activation ($\Delta G^{\ddagger}$) listed in Table S5.

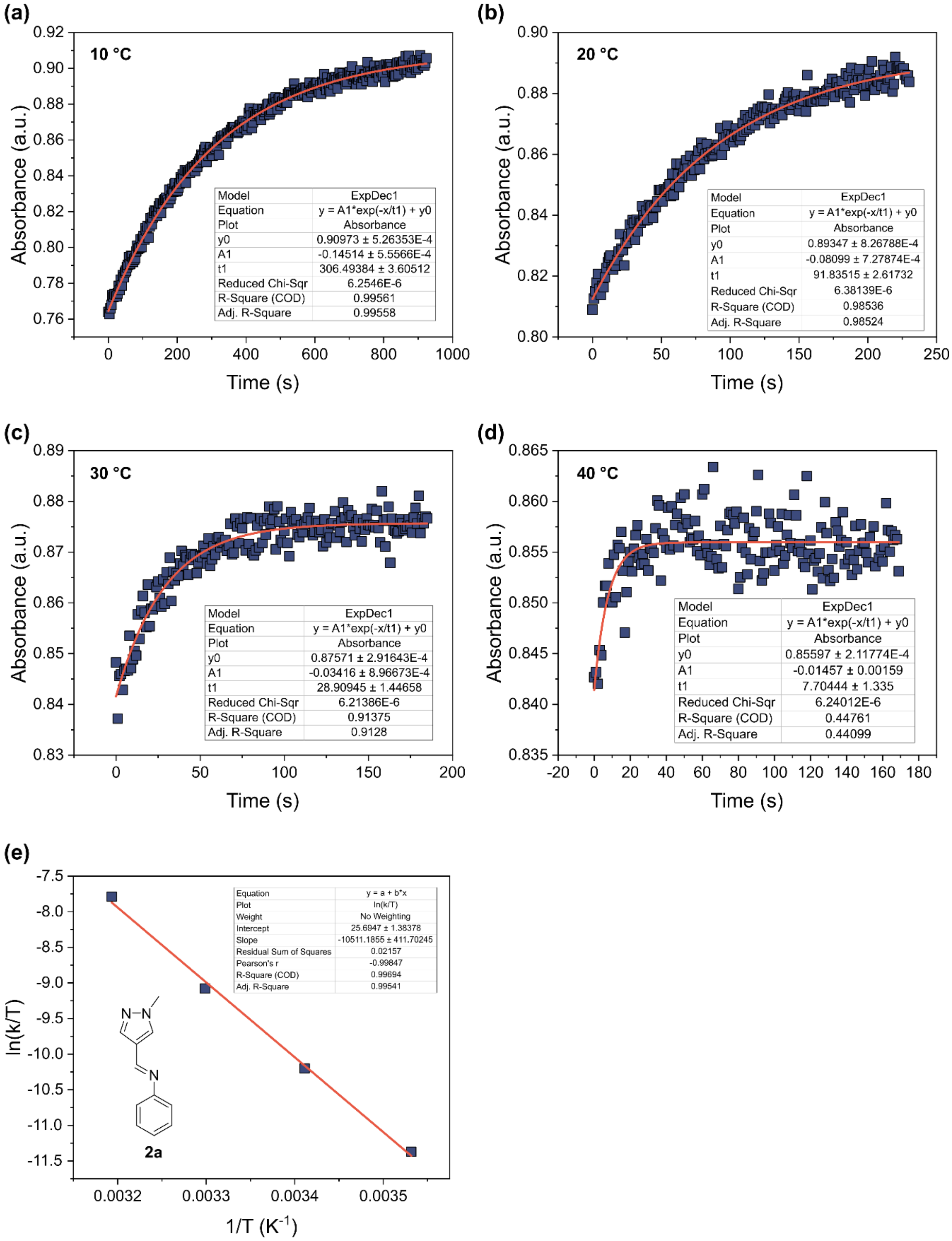

Figure S38. The change in absorbance of **2a** at the λ_{max} of the *E*-isomer over time at **a**) 10 °C, **b**) 20 °C, **c**) 30 °C, and **d**) 40 °C with the increase in absorbance relating to the thermal isomerization of the *Z*-isomer to the *E*-isomer. All samples were irradiated under 365 nm LED for 5 minutes in acetonitrile before the measurements started. The exponential fittings were applied to determine the rate constants and thus thermal half-lives. **e**) The Eyring plots were generated using the rate constants calculated at different temperatures, and the fitted parameter was used to estimate the enthalpy of activation ($\Delta H^{‡}$), entropy of activation ($\Delta S^{‡}$), and Gibbs energy of activation ($\Delta G^{‡}$) listed in Table S5.

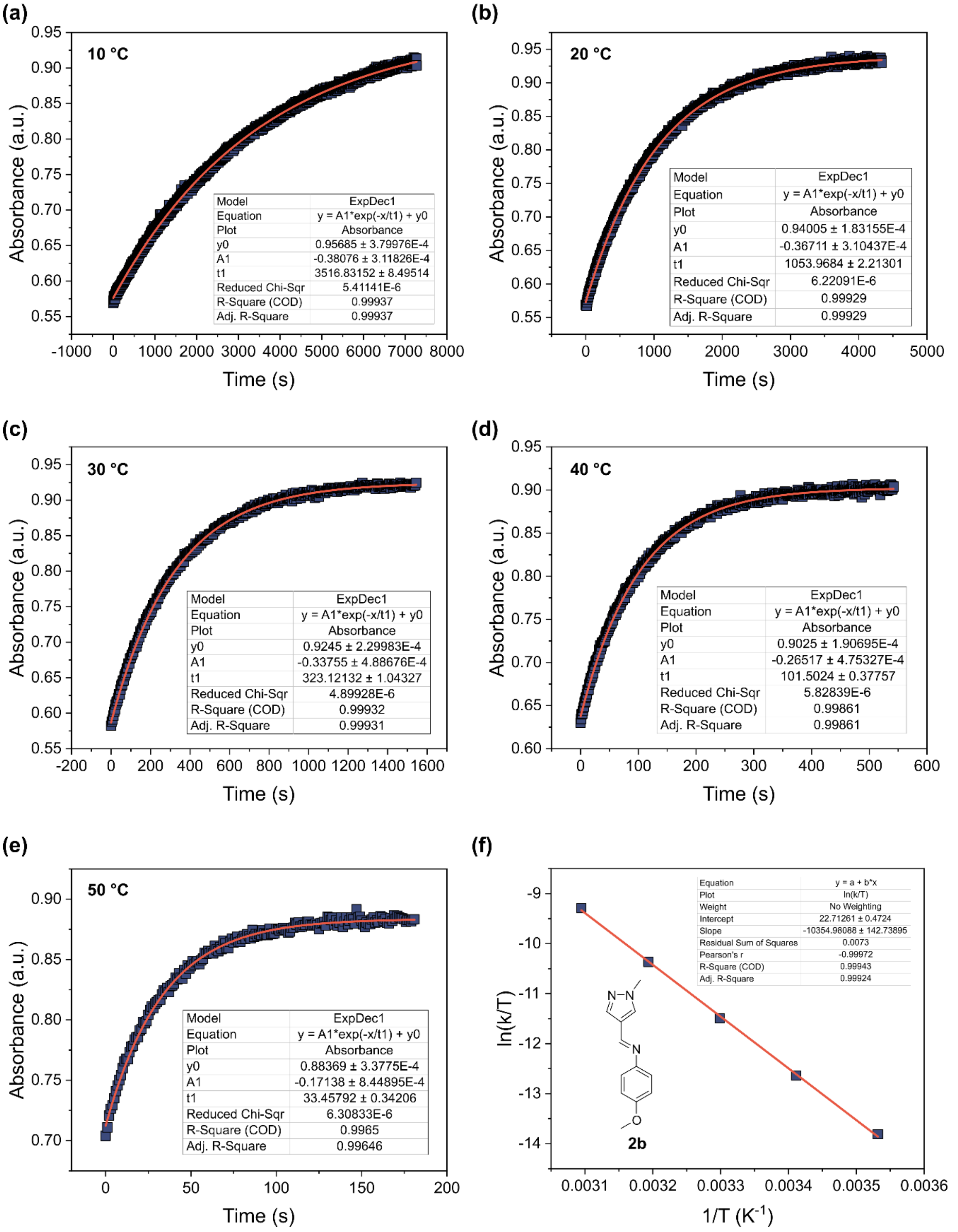

Figure S39. The change in absorbance of **2b** at the λ_{max} of the *E*-isomer over time at **a**) 10 °C, **b**) 20 °C, **c**) 30 °C, **d**) 40 °C, **e**) 50 °C with the increase in absorbance relating to the thermal isomerization of the *Z*-isomer to the *E*-isomer. All samples were irradiated under 365 nm LED for 5 minutes in acetonitrile before the measurements started. The exponential fittings were applied to determine the rate constants and thus thermal half-lives. **f**) The Eyring plots were generated using the rate constants calculated at different temperatures, and the fitted parameter was used to estimate the enthalpy of activation ($\Delta H^{‡}$), entropy of activation ($\Delta S^{‡}$), and Gibbs energy of activation ($\Delta G^{‡}$) listed in Table S5.

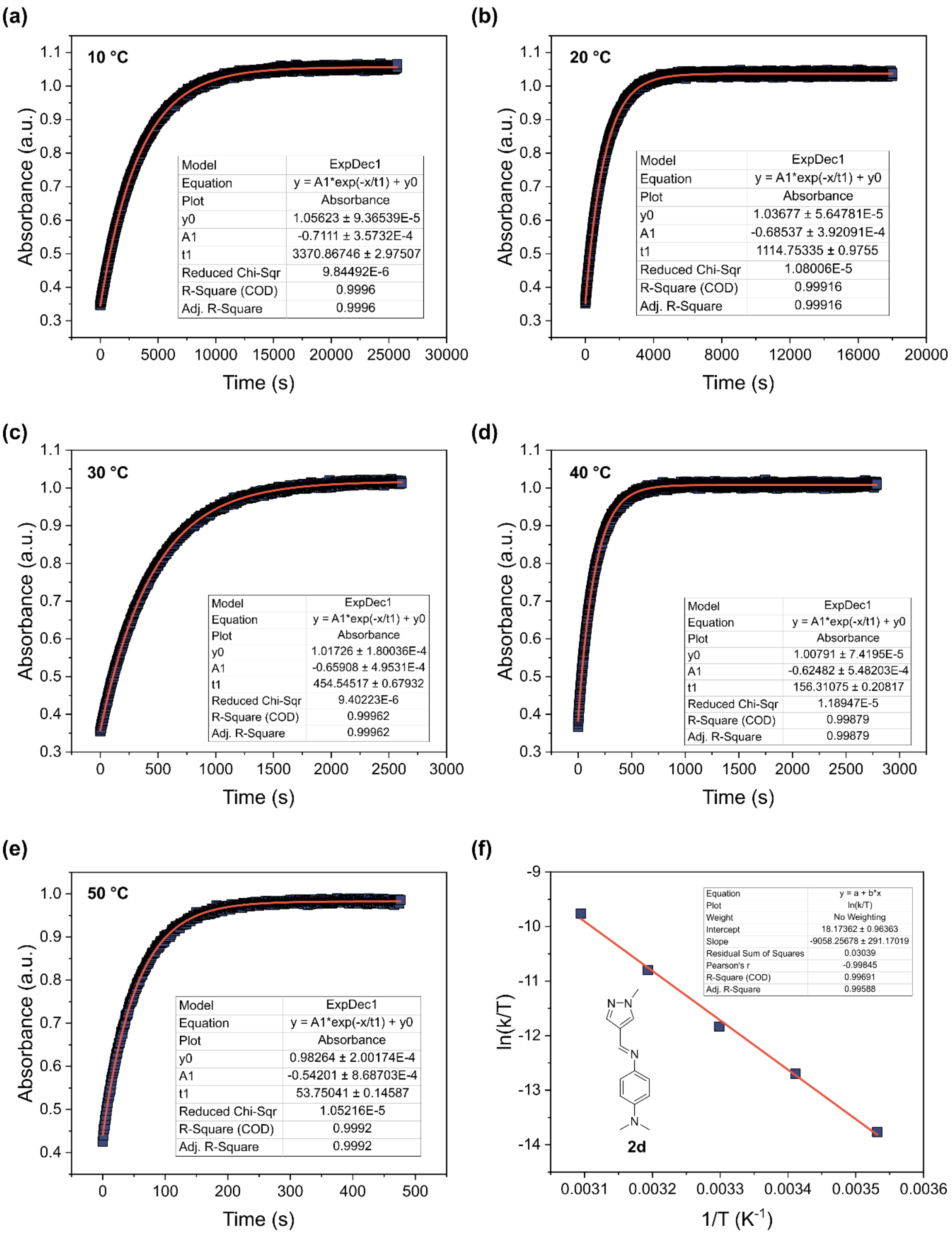

Figure S40. The change in absorbance of **2d** at the λ_{max} of the *E*-isomer over time at **a**) 10 °C, **b**) 20 °C, **c**) 30 °C, **d**) 40 °C, **e**) 50 °C with the increase in absorbance relating to the thermal isomerization of the *Z*-isomer to the *E*-isomer. All samples were irradiated under 365 nm LED for 5 minutes in acetonitrile before the measurements started. The exponential fittings were applied to determine the rate constants and thus thermal half-lives. **f**) The Eyring plots were generated using the rate constants calculated at different temperatures, the fitted parameter was used to estimate the enthalpy of activation ($\Delta H^{\ddagger}$), entropy of activation ($\Delta S^{\ddagger}$), and Gibbs energy of activation ($\Delta G^{\ddagger}$) listed in Table S5.

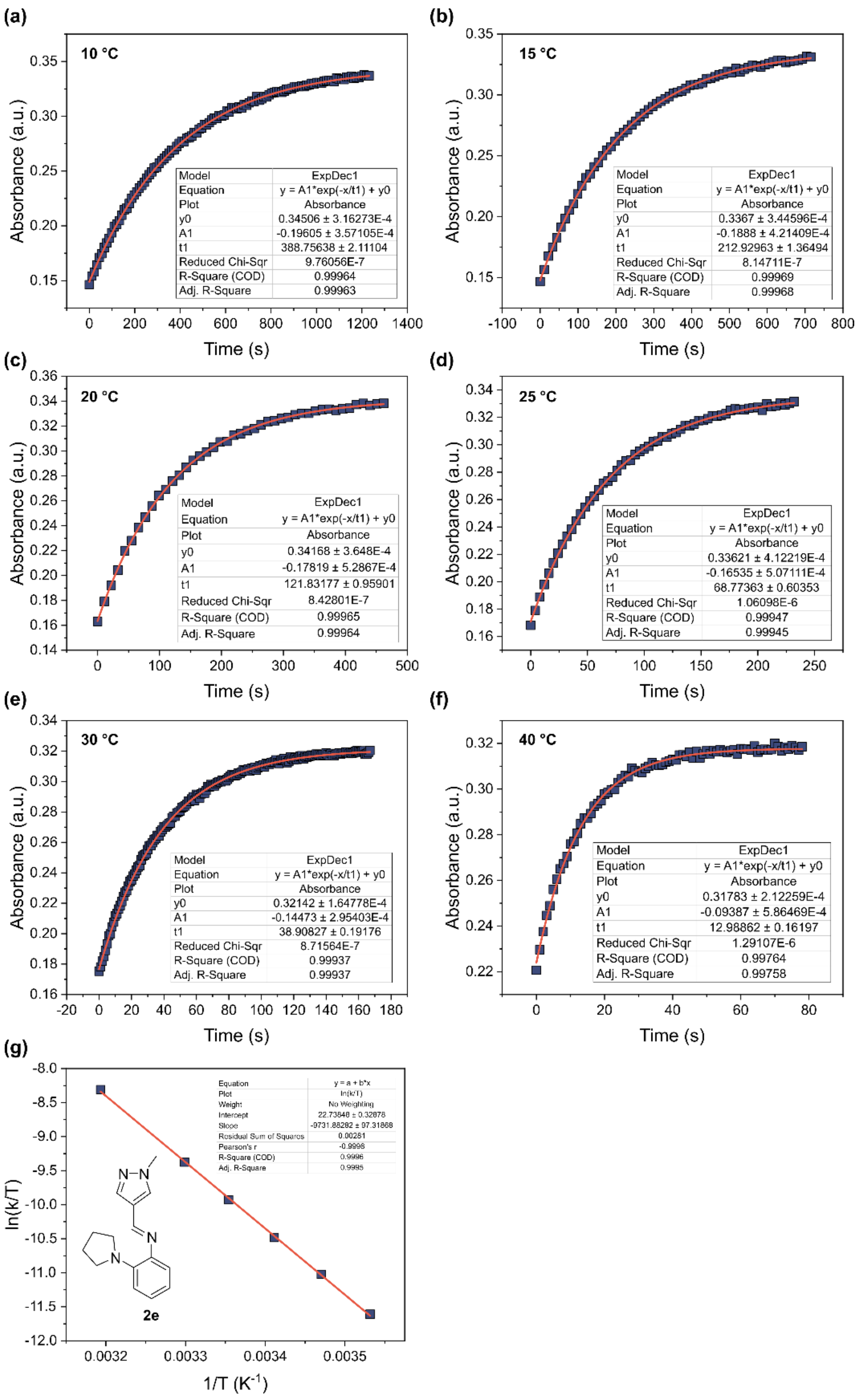

Figure S41. The change in absorbance of **2e** at the λ_{max} of the *E*-isomer over time at **a**) 10 °C, **b**) 15 °C, **c**) 20 °C, **d**) 25 °C, **e**) 30 °C, and **f**) 40 °C with the increase in absorbance relating to the thermal isomerization of the *Z*-isomer to the *E*-isomer. All samples were irradiated under 365 nm LED for 5 minutes in acetonitrile before the measurements started. The exponential fittings were applied to determine the rate constants and thus thermal half-lives. **g**) The Eyring plots were generated using the rate constants calculated at different temperatures, and the fitted parameter was used to estimate the enthalpy of activation ($\Delta H^‡$), entropy of activation ($\Delta S^‡$), and Gibbs energy of activation ($\Delta G^‡$) listed in Table S5.

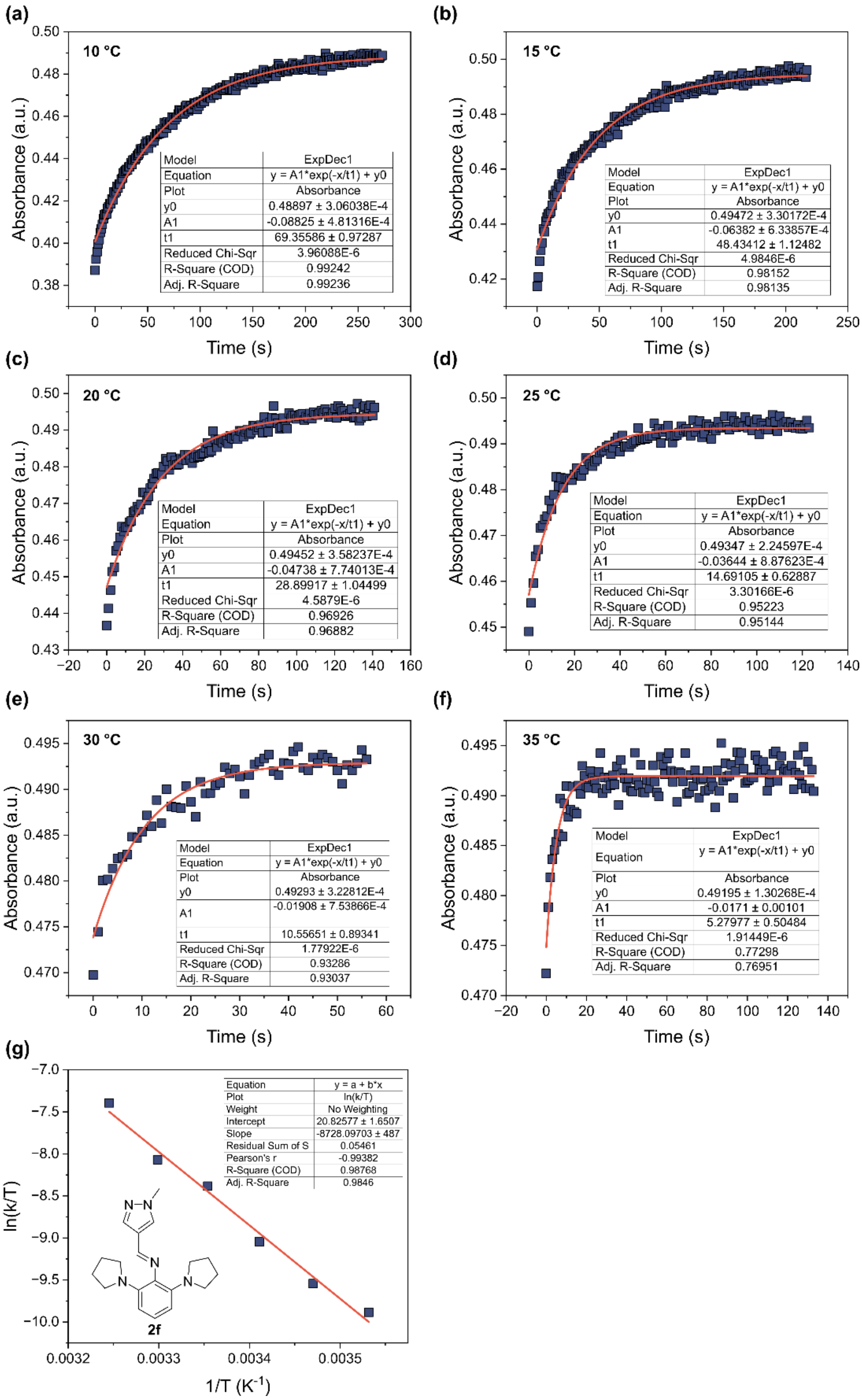

Figure S42. The change in absorbance of **2f** at the λ_{max} of the *E*-isomer over time at **a**) 10 °C, **b**) 15 °C, **c**) 20 °C, **d**) 25 °C, **e**) 30 °C, and **f**) 35 °C with the increase in absorbance relating to the thermal isomerization of the *Z*-isomer to the *E*-isomer. All samples were irradiated under 365 nm LED for 5 minutes in acetonitrile before the measurements started. The exponential fittings were applied to determine the rate constants and thus thermal half-lives. **g**) The Eyring plots were generated using the rate constants calculated at different temperatures, and the fitted parameter was used to estimate the enthalpy of activation ($\Delta H^‡$), entropy of activation ($\Delta S^‡$), and Gibbs energy of activation ($\Delta G^‡$) listed in Table S5.

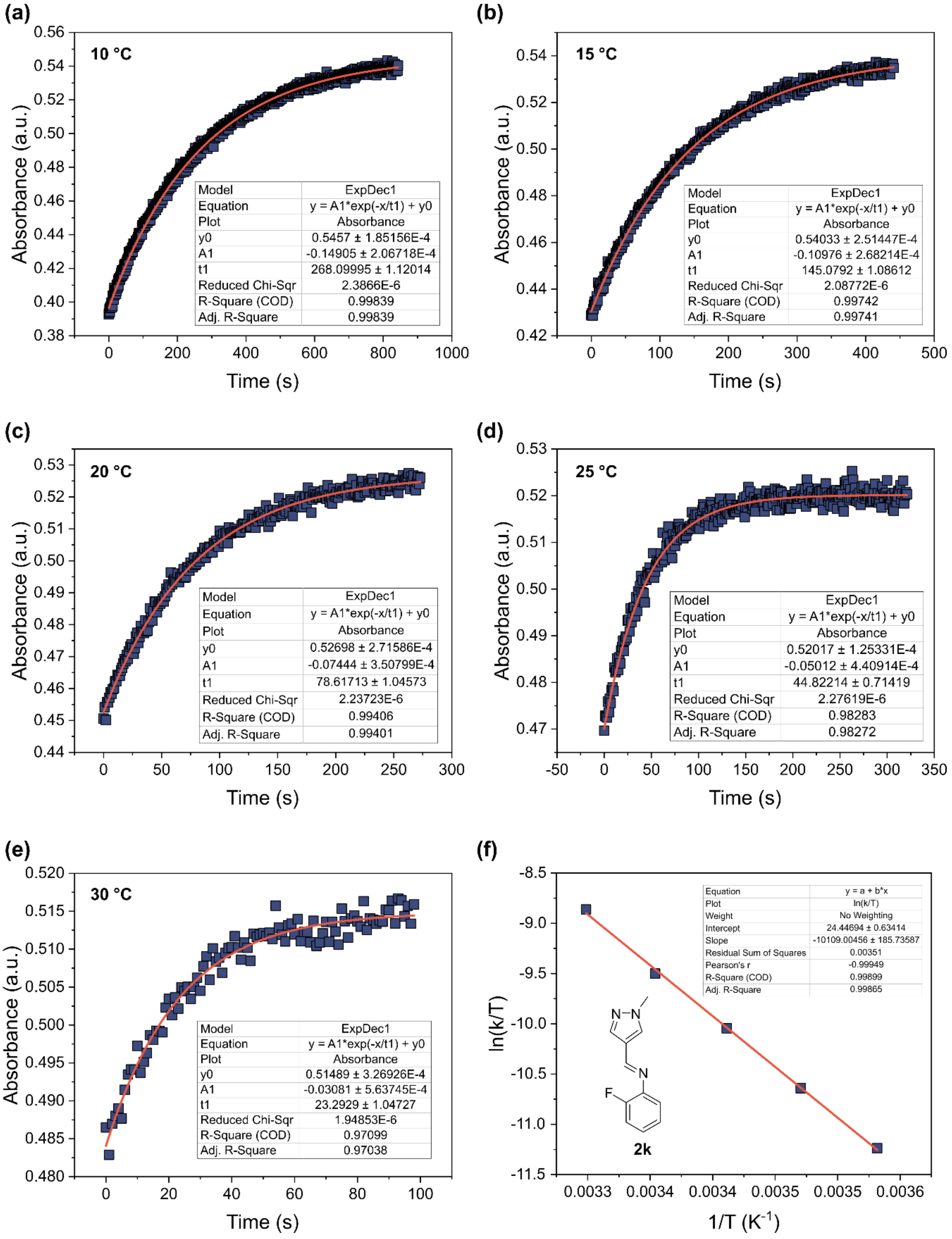

Figure S43. The change in absorbance of **2k** at the λ_{max} of the *E*-isomer over time at **a**) 10 °C, **b**) 15 °C, **c**) 20 °C, **d**) 25 °C, **e**) 30 °C with the increase in absorbance relating to the thermal isomerization of the *Z*-isomer to the *E*-isomer. All samples were irradiated under 365 nm LED for 5 minutes in acetonitrile before the measurements started. The exponential fittings were applied to determine the rate constants and thus thermal half-lives. **f**) The Eyring plots were generated using the rate constants calculated at different temperatures, and the fitted parameter was used to estimate the enthalpy of activation ($\Delta H^{\ddagger}$), entropy of activation ($\Delta S^{\ddagger}$), and Gibbs energy of activation ($\Delta G^{\ddagger}$) listed in Table S5.

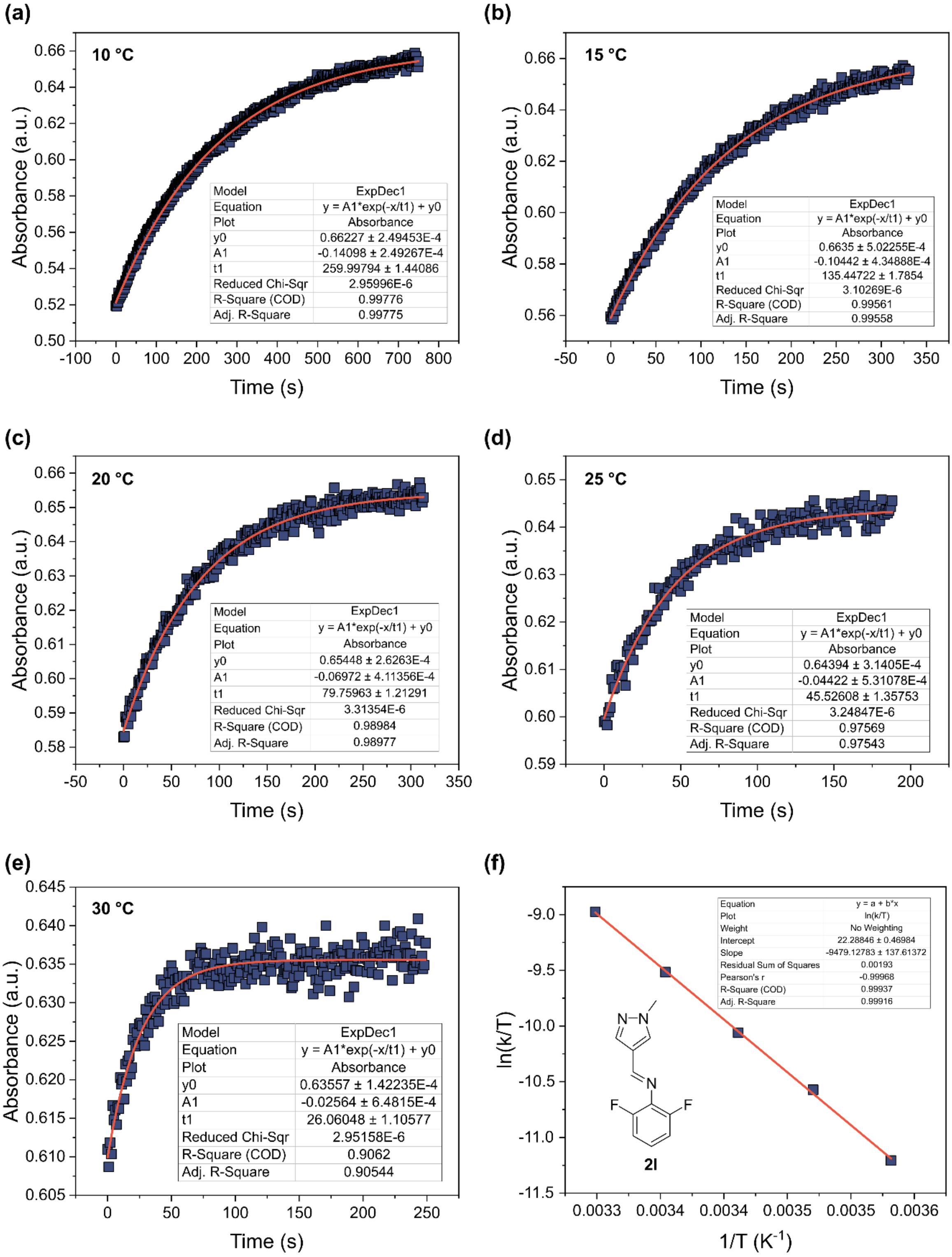

Figure S44. The change in absorbance of **2l** at the λ_{max} of the *E*-isomer over time at **a**) 10 °C, **b**) 15 °C, **c**) 20 °C, **d**) 25 °C, **e**) 30 °C with the increase in absorbance relating to the thermal isomerization of the *Z*-isomer to the *E*-isomer. All samples were irradiated under 365 nm LED for 5 minutes in acetonitrile before the measurements started. The exponential fittings were applied to determine the rate constants and thus thermal half-lives. **f**) The Eyring plots were generated using the rate constants calculated at different temperatures, and the fitted parameter was used to estimate the enthalpy of activation ($\Delta H^{\ddagger}$), entropy of activation ($\Delta S^{\ddagger}$), and Gibbs energy of activation ($\Delta G^{\ddagger}$) listed in Table S5.

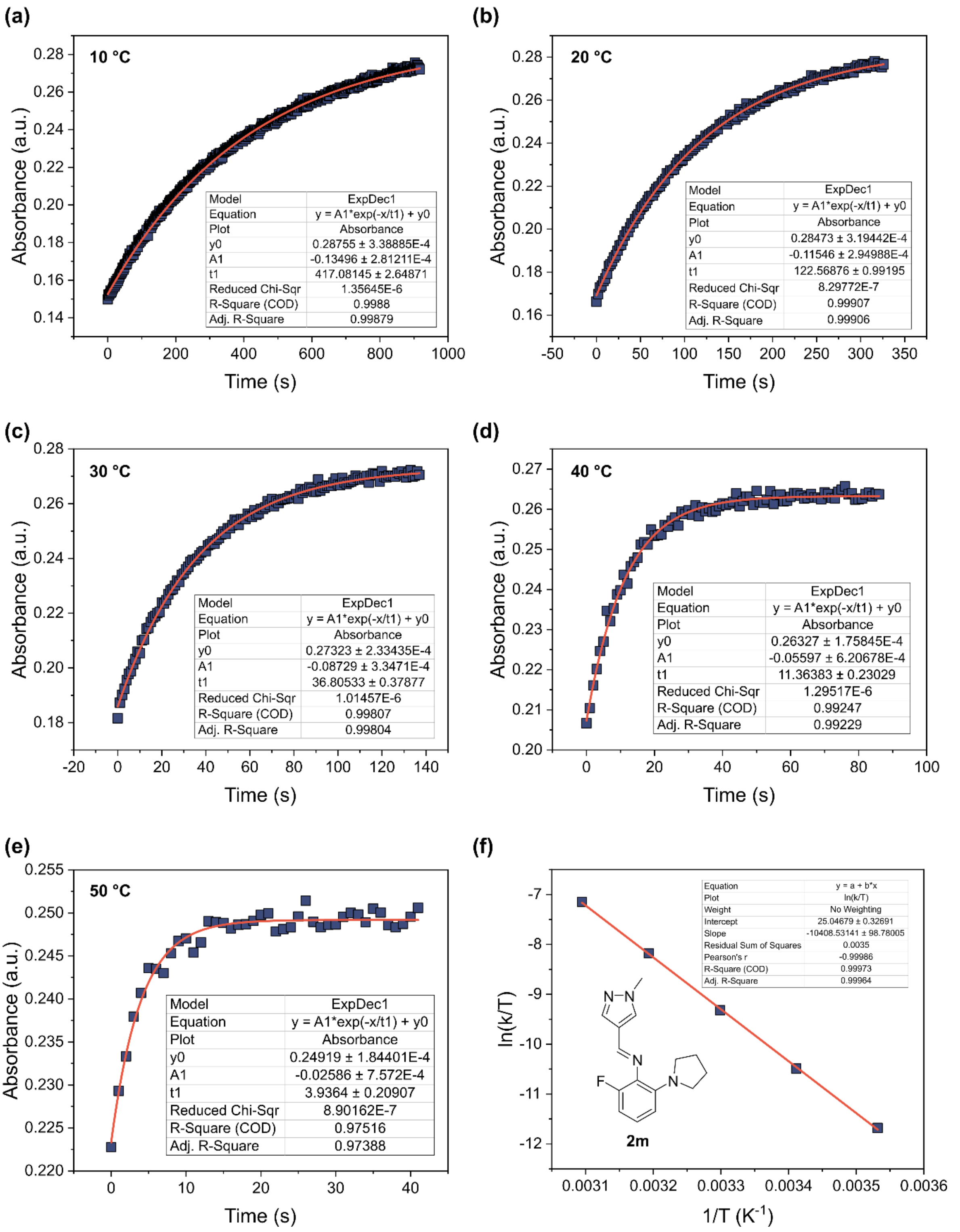

Figure S45. The change in absorbance of **2m** at the λ_{max} of the *E*-isomer over time at **a**) 10 °C, **b**) 20 °C, **c**) 30 °C, **d**) 40 °C, **e**) 50 °C with the increase in absorbance relating to the thermal isomerization of the *Z*-isomer to the *E*-isomer. All samples were irradiated under 365 nm LED for 5 minutes in acetonitrile before the measurements started. The exponential fittings were applied to determine the rate constants and thus thermal half-lives. **f**) The Eyring plots were generated using the rate constants calculated at different temperatures, and the fitted parameter was used to estimate the enthalpy of activation ($\Delta H^{\ddagger}$), entropy of activation ($\Delta S^{\ddagger}$), and Gibbs energy of activation ($\Delta G^{\ddagger}$) listed in Table S5.

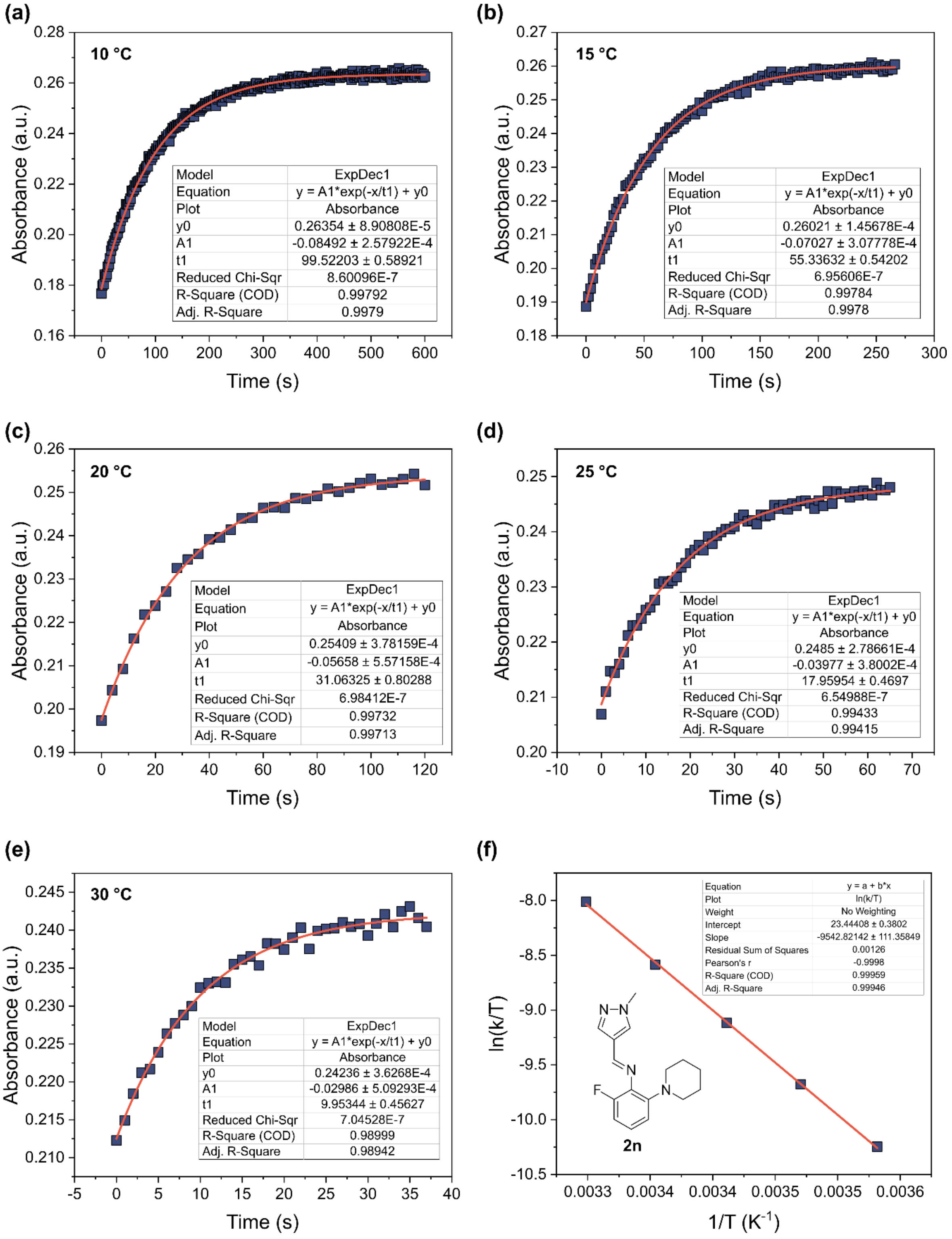

Figure S46. The change in absorbance of **2n** at the λmax of the *E*-isomer over time at **a**) 10 °C, **b**) 15 °C, **c**) 20 °C, **d**) 25 °C, **e**) 30 °C with the increase in absorbance relating to the thermal isomerization of the *Z*-isomer to the *E*-isomer. All samples were irradiated under 365 nm LED for 5 minutes in acetonitrile before the measurements started. The exponential fittings were applied to determine the rate constants and thus thermal half-lives. **f**) The Eyring plots were generated using the rate constants calculated at different temperatures, and the fitted parameter was used to estimate the enthalpy of activation ($\Delta H^‡$), entropy of activation ($\Delta S^‡$), and Gibbs energy of activation ($\Delta G^‡$) listed in Table S5.

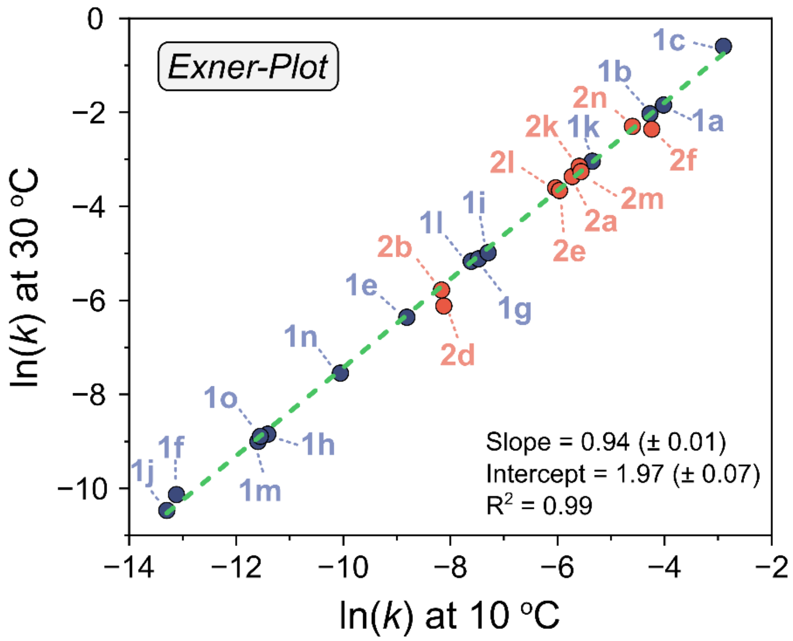

Figure S47. The Exner plot of the AIPs exhibits a linear relationship, indicating that the *E*-to-*Z* thermal isomerization follows the same mechanism in the experimental temperature range.[6]

3.3 Photo-Stationary States (PSS)

Irradiation for PSS measurements was conducted using the diode array setup (Section 1 of Supporting Information) with LED lights ranging from 340 nm to 470 nm, as detailed in Table S1. The PSS at a given irradiation wavelength, λ, can be defined as an equilibrium constant or percentage distribution based on the proportion of isomer present (x_Z and x_E). This distribution of isomers, under photoirradiation, is governed by the extinction coefficient, ε, and quantum yield of photoisomerism, Φ, of each isomer, as shown below.

$$K_{eq}^{\lambda} = \frac{\varepsilon_E^{\lambda} \cdot \Phi_{E\to Z}^{\lambda}}{\varepsilon_Z^{\lambda} \cdot \Phi_{Z\to E}^{\lambda}} \quad , \quad \%Z = \frac{x_Z}{x_Z + x_E} \times 100$$

The proportions of the *Z*-isomers at the PSS of all imines investigated in this study were determined by UV-vis spectroscopy at room temperature, using acetonitrile as the solvent. The calculations of PSS were performed according to Fischer's approach,[7] by analyzing the absorption spectra of the pure *E*-isomer before irradiation and after photo-equilibration at two different wavelengths.

The absorption spectra of *Z*-isomers can be estimated by the relationship between the dark spectra (Pure *E*), the PSS spectra and the PSS quantity, as presented below:

$$Estimated\ Pure\ Z = (Spectra\ of\ pure\ E) + \frac{(PSS\ spectra) - (Pure\ E\ spectra)}{(PSS\ quantity)}$$

Additionally, for imine **1m**, **1n**, and **1o**, ^{1}H NMR measurements were employed to ascertain the isomer ratios, as shown in Figure S52. These results corroborated the values obtained from UV-vis spectroscopy.

Notably, for the switches with short thermal half-lives, it's challenging to achieve the real photostationary states due to competing thermal relaxation during photoisomerization. Thus, here we use the maximum achievable %*Z* isomer to determine the properties of these imines, specifically **1b**, **1c**, **1k**, **2a**, **2e**, **2f**, **2k**, **2l**, **2m**, and **2n**. The experimentally measured quantity of *Z*% is an important descriptor when selecting photoswitches for a particular application.

It is also important to note that our setup, shown schematically in Figure S1, facilitates the rapid acquisition of the UV/vis spectra: 50 ms integration time, average of 10 spectra, resulting in a spectrum being recorded every 0.5 s. The combination of the high intensity of irradiation, rapid spectral acquisition, and temperature control limits the amount of time between irradiation and spectral acquisition, providing a relatively more reliable measurement of the %*Z* isomer at the given irradiation wavelength, intensity, and temperature.

While only the %*Z* isomer is reported in this work, the %*E*-isomer can be readily calculated by the following relation: %*E*-isomer = 100% − %*Z*-isomer.

Table S6. The summary of the maximum achievable %*Z* isomer at 20 °C of all imine switches included in this work. Data of **1a**, **1e**, and **1f** are obtained from our previous work.[1] Note that the %E-isomer can be calculated from 100% − %Z-isomer.

	340 nm	365 nm	385 nm	405 nm	430 nm	470 nm
**1a***	(37%)	(27%)	(7%)	-	-	-
**1b***	(6%)	(18%)	(2%)	-	-	-
**1c***	(1%)	(29%)	(29%)	(7%)	(2%)	-
**1e**	35%	66%	82%	94%	90%	55%
**1f**	38%	68%	79%	95%	99%	94%
**1g**	29%	58%	63%	60%	50%	-
**1h**	56%	74%	86%	88%	91%	92%
**1i**	33%	58%	61%	57%	24%	-
**1j**	50%	70%	81%	86%	86%	-
**1k**	29%	46%	10%	4%	-	-
**1l**	66%	72%	59%	7%	-	-
**1m**	23%	67%	85%	92%	89%	-
**1n**	52%	84%	85%	80%	11%	-
**1o**	45%	78%	89%	78%	43%	-
**2a**	12%	15%	6%	-	-	-
**2b**	78%	63%	22%	9%	-	-
**2c***	-	-	-	-	-	-
**2d**	51%	71%	67%	44%	17%	-
**2e**	10%	71%	71%	75%	59%	26%
**2f***	(1%)	(18%)	(23%)	(25%)	(6%)	-
**2k**	10%	16%	1%	-	-	-
**2l**	9%	17%	1%	-	-	-
**2m**	5%	53%	53%	53%	25%	5%
**2n***	(2%)	(30%)	(16%)	(7%)	-	-

*Significant thermal reversion at room temperature prevented the attainment of a PSS.

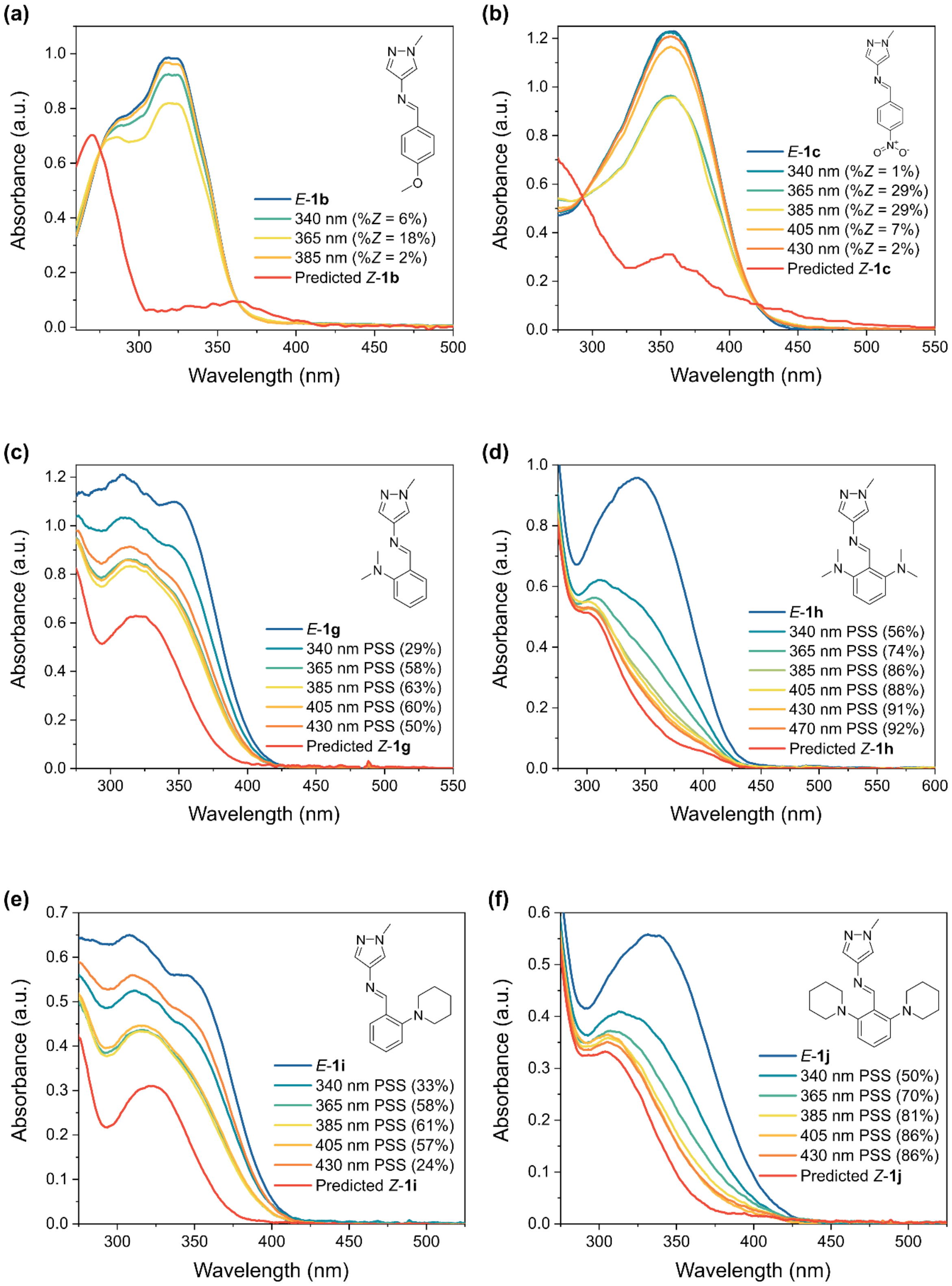

Figure S48. The UV/vis spectra of **1b** (50 μM in MeCN), **1c** (60 μM in MeCN), **1g** (120 μM in MeCN), **1h** (120 μM in MeCN), **1i** (70 μM in MeCN) and **1j** (70 μM in MeCN) under different irradiation conditions, alongside the predicted *Z*-isomer spectra. For **1g**, **1h**, **1i** and **1j**, the proportions of *Z*-isomers under different wavelength irradiations were quantified as PSS values, with the *E*-isomer set at 0% and the predicted *Z*-isomer at 100%.

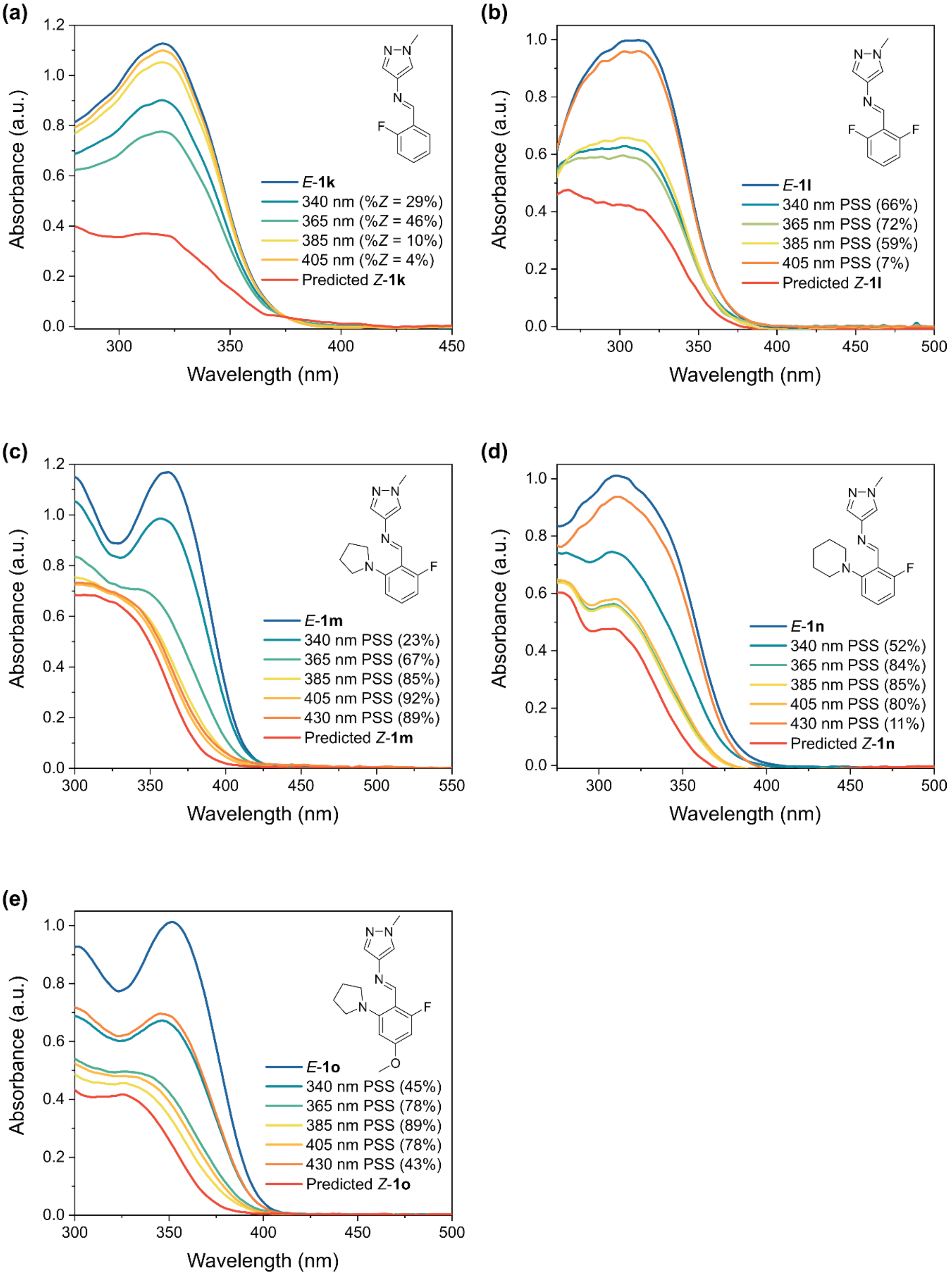

Figure S49. The UV/vis spectra of **1k** (70 μM in MeCN), **1l** (80 μM in MeCN), **1m** (170 μM in MeCN), **1n** (80 μM in MeCN) and **1o** (100 μM in MeCN) under different irradiation conditions, alongside the predicted *Z*-isomer spectra. For **1l**, **1m**, **1n** and **1o**, the proportions of *Z*-isomers under different wavelength irradiations were quantified as PSS values, with the *E*-isomer set at 0% and the predicted *Z*-isomer at 100%.

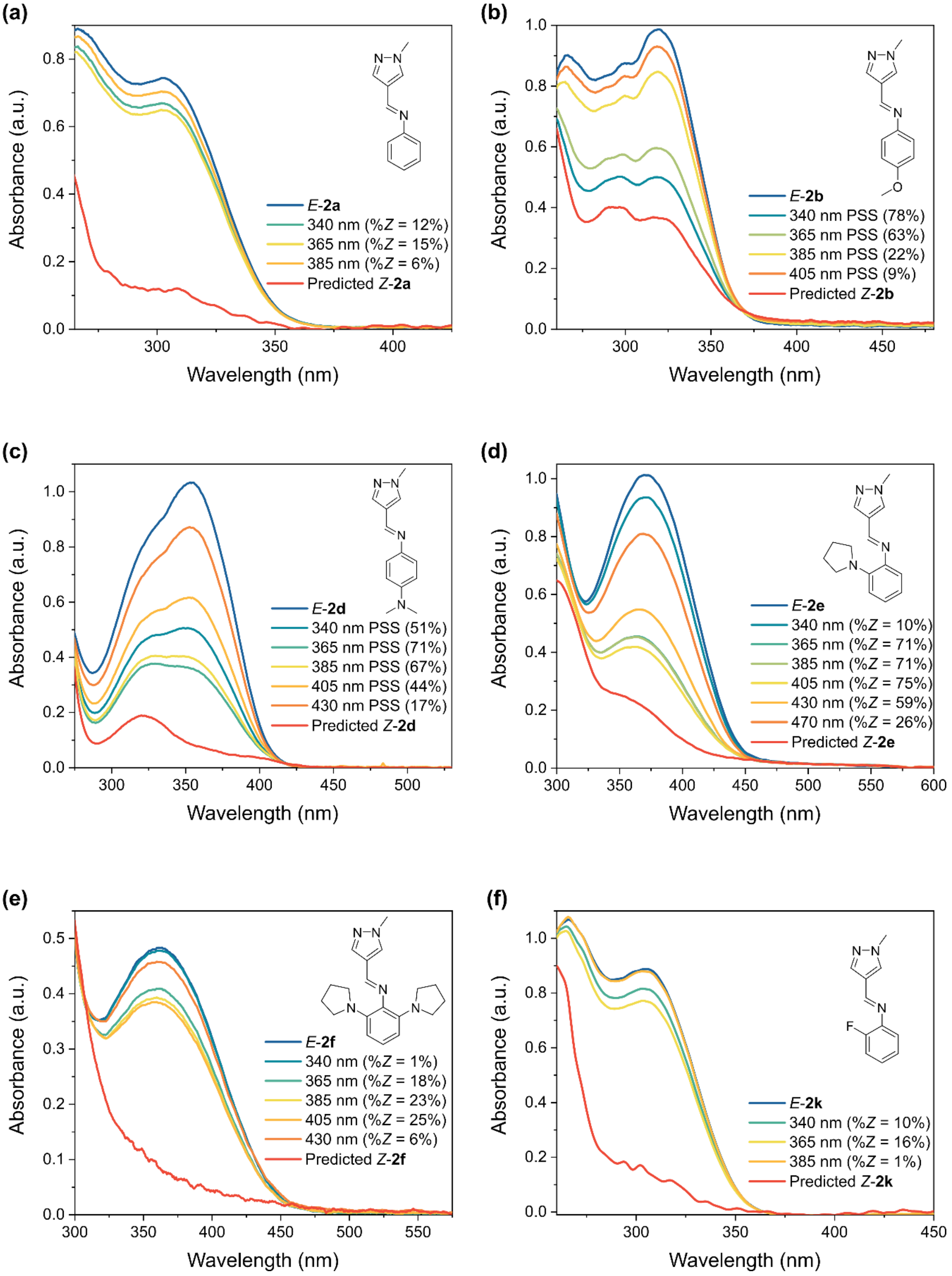

Figure S50. The UV/vis spectra of **2a** (70 μM in MeCN), **2b** (70 μM in MeCN), **2d** (60 μM in MeCN), **2e** (220 μM in MeCN), **2f** (220 μM in MeCN) and **2k** (130 μM in MeCN) under different irradiation conditions, alongside the predicted *Z*-isomer spectra. For **2b** and **2d**, the proportions of *Z*-isomers under different wavelength irradiations were quantified as PSS values, with the *E*-isomer set at 0% and the predicted *Z*-isomer at 100%.

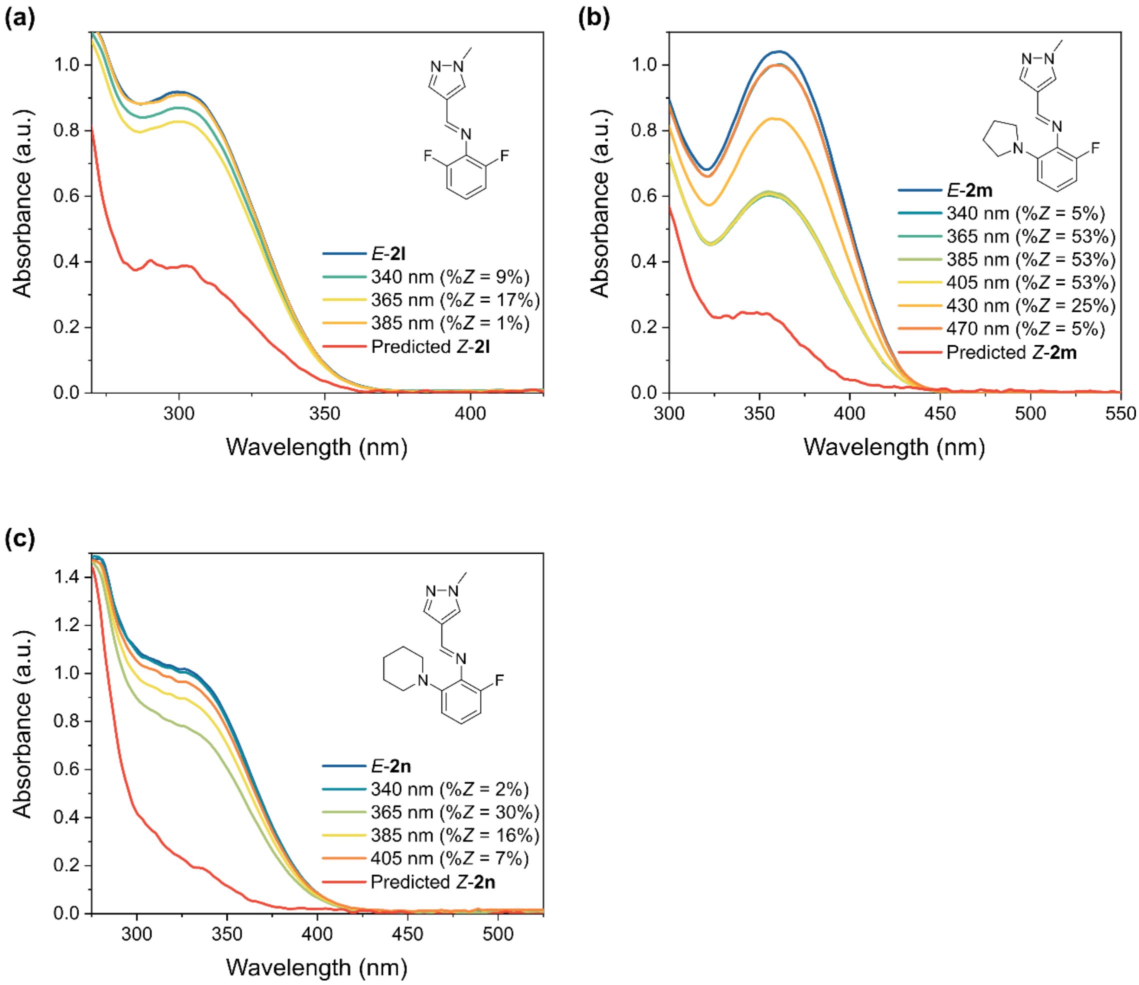

Figure S51. The UV/vis spectra of **2l** (100 μM in MeCN), **2m** (260 μM in MeCN) and **2n** (300 μM in MeCN) under different irradiation conditions, alongside the predicted *Z*-isomer spectra.

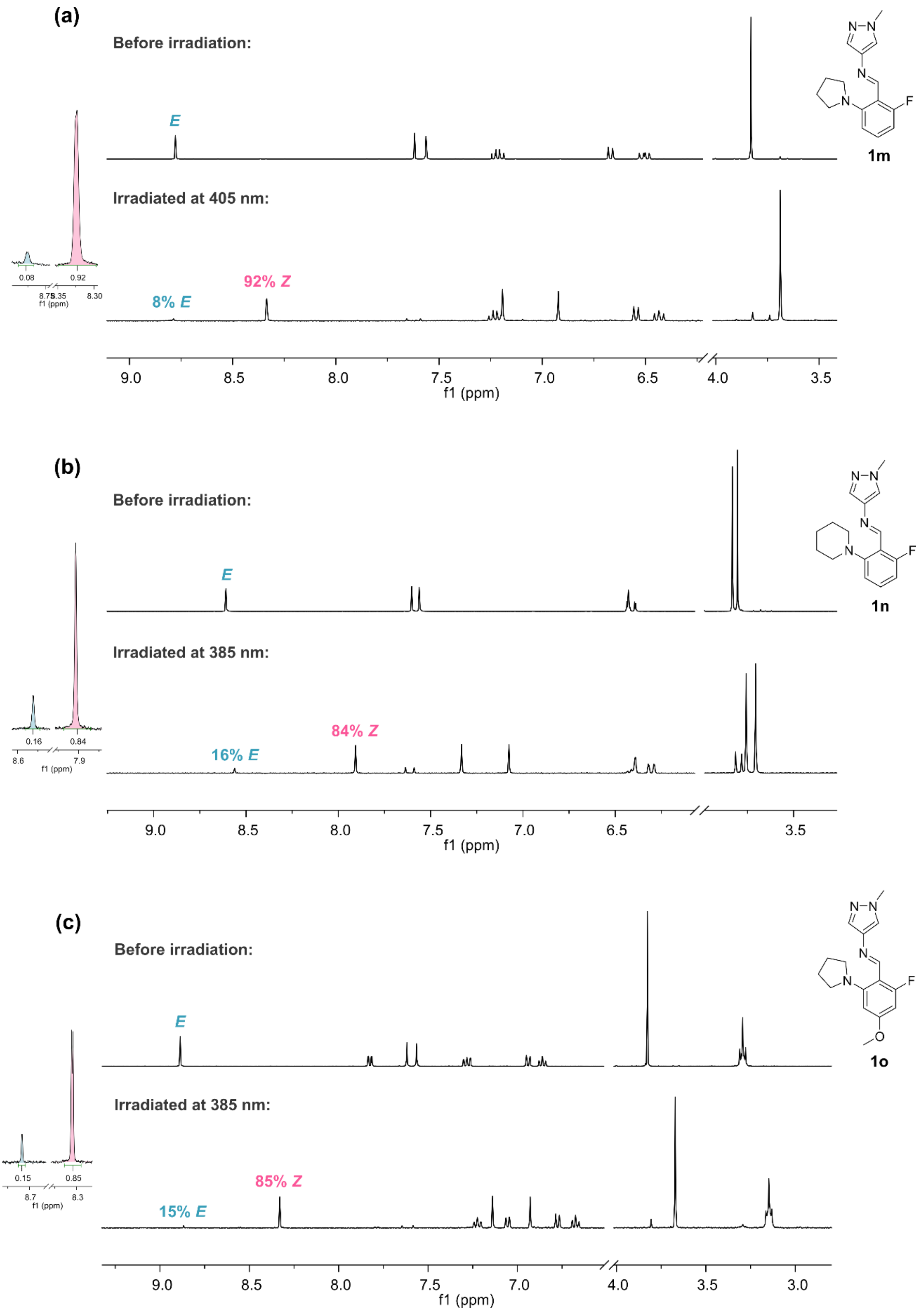

Figure S52. ^{1}H NMR spectra (400 MHz, MeCN-*d3*) of (a) **1m** before irradiation (above) and after irradiation under 405 nm LED (below); (b) **1n** before irradiation (above) and after irradiation under 405 nm LED (below); (c) **1o** before irradiation (above) and after irradiation under 385 nm LED (below). *Z*-isomer proportions were determined by the integration of labelled signals to determine PSS at the specified wavelength. Samples were irradiated in NMR tubes until no further change was observed in the distribution of the *E*/*Z* isomers in the ^{1}H NMR spectra.

3.4 Quantum Yields

The irradiation setup employed in this study was identical to that described in our previous work.[1] The photon fluxes provided by the light source with the wavelengths of 340 nm, 365 nm, 385 nm, 405 nm, and 430 nm are listed in Table S7.

Table S7. The photon fluxes provided by light sources with different wavelengths respectively.[1]

**Wavelength**	**Photon Flux** (Photons/s)
340 nm	4.25×10^{14}
365 nm	6.32×10^{14}
385 nm	7.38×10^{14}
405 nm	6.29×10^{14}
430 nm	7.61×10^{14}

The samples of imines were prepared in acetonitrile at room temperature. The irradiation and measurements were performed in the diode array setup simultaneously, with the same irradiation conditions as the calibration process[2,8] as detailed in our previous work:[1] 340 nm (500 mA, 90%), 365 nm (200 mA, 20%), 385 nm (200 mA, 20%), 405 nm (200 mA, 20%), 430 nm (200 mA, 20%), respectively.

Table S8. The summary of quantum yields under different wavelengths of all imines studied in this work. The quantum yields of **1a**, **1e**, and **1f** are obtained from our previous work.[1] The values listed correspond to the quantum yields of *E*-to-*Z* photoisomerism; the corresponding quantum yields of *Z*-to-*E* photoisomerism are given in parentheses.

	**340 nm**	**365 nm**	**385 nm**	**405 nm**	**430 nm**
**1a**	3 (10)	-	-	-	-
**1b**	1 (7)	-	-	-	-
**1c**	1 (8)	1 (35)	2 (34)	-	-
**1e**	2 (2)	2 (2)	3 (2)	3 (1)	4 (0)
**1f**	1 (1)	1 (1)	1 (1)	1 (0)	1 (0)
**1g**	1 (2)	2 (4)	3 (7)	-	-
**1h**	1 (2)	1 (2)	1 (2)	1 (0)	-
**1i**	3 (8)	7 (30)	3 (0)	-	-
**1j**	2 (5)	4 (12)	7 (7)	3 (3)	-
**1k**	1 (0)	3 (6)	-	-	-
**1l**	1 (0)	2 (3)	1 (0)	-	-
**1m**	1 (2)	2 (3)	2 (2)	1 (1)	-
**1n**	1 (1)	2 (1)	4 (0)	-	-
**1o**	1 (2)	1 (3)	2 (0)	3 (0)	-
**2a**	1 (0)	-	-	-	-
**2b**	1 (0)	5 (0)	-	-	-
**2c**	-	-	-	-	-
**2d**	3 (11)	6 (32)	10 (56)	7 (31)	-
**2e**	1 (1)	2 (1)	2 (2)	2 (1)	3 (0)
**2f**	-	-	-	-	-
**2k**	1 (9)	-	-	-	-
**2l**	1 (3)	-	-	-	-
**2m**	1 (2)	1 (1)	2 (4)	1 (1)	2 (0)
**2n**	1 (1)	1 (0)	3 (0)	1(0)	-

3.5 Action Plots

Action plots were constructed. Briefly, the photon flux of the different irradiation wavelengths was calculated by chemical actinometry. For each wavelength, the yield of *E*-to-*Z* photoisomerism was determined once 8.5×10^{15} photons had been delivered to the sample. Note that this corresponds to less than 30% conversion in all cases. These action plots, shown together with the absorption spectra of the *E*-isomers and predicted spectra of the *Z*-isomers, are shown below.

In general, a small redshift in the action plot was observed compared to the UV/vis absorption spectra. We attribute this to the overlapping absorption spectra. Whereby lower energy light can be used to selectively address more of the *E*-isomer compared to the *Z*-isomer.

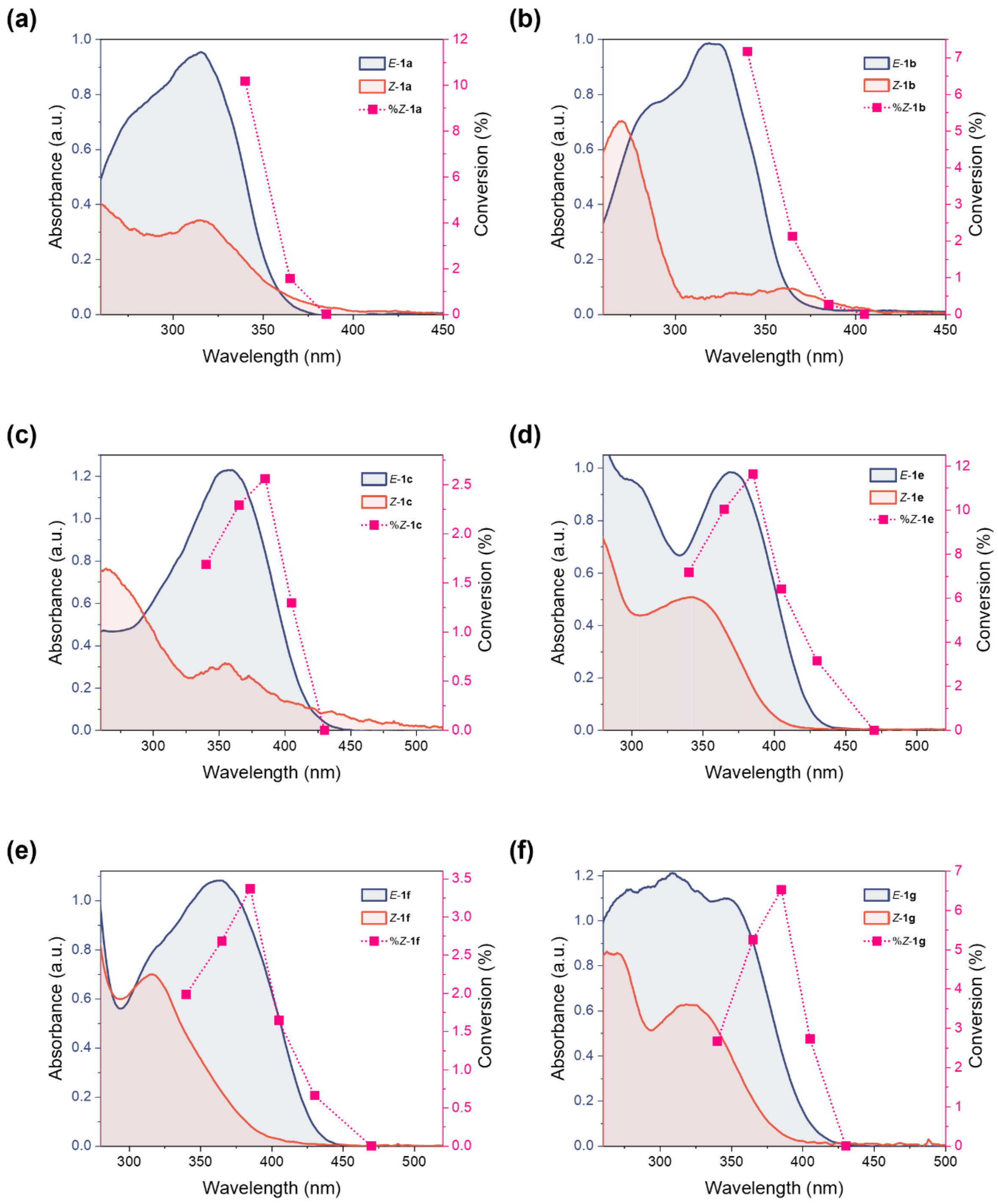

Figure S53. Action plots of (a) **1a**, (b) **1b**, (c) **1c**, (d) **1e**, (e) **1f**, and (f) **1g**. The conversions to *Z*-isomers are calculated for each irradiation wavelength when 8.5×10^{15} photons are delivered into the system according to the photon fluxes listed in Table S7. Spectra of *E*- and *Z*- isomers are presented for comparison.

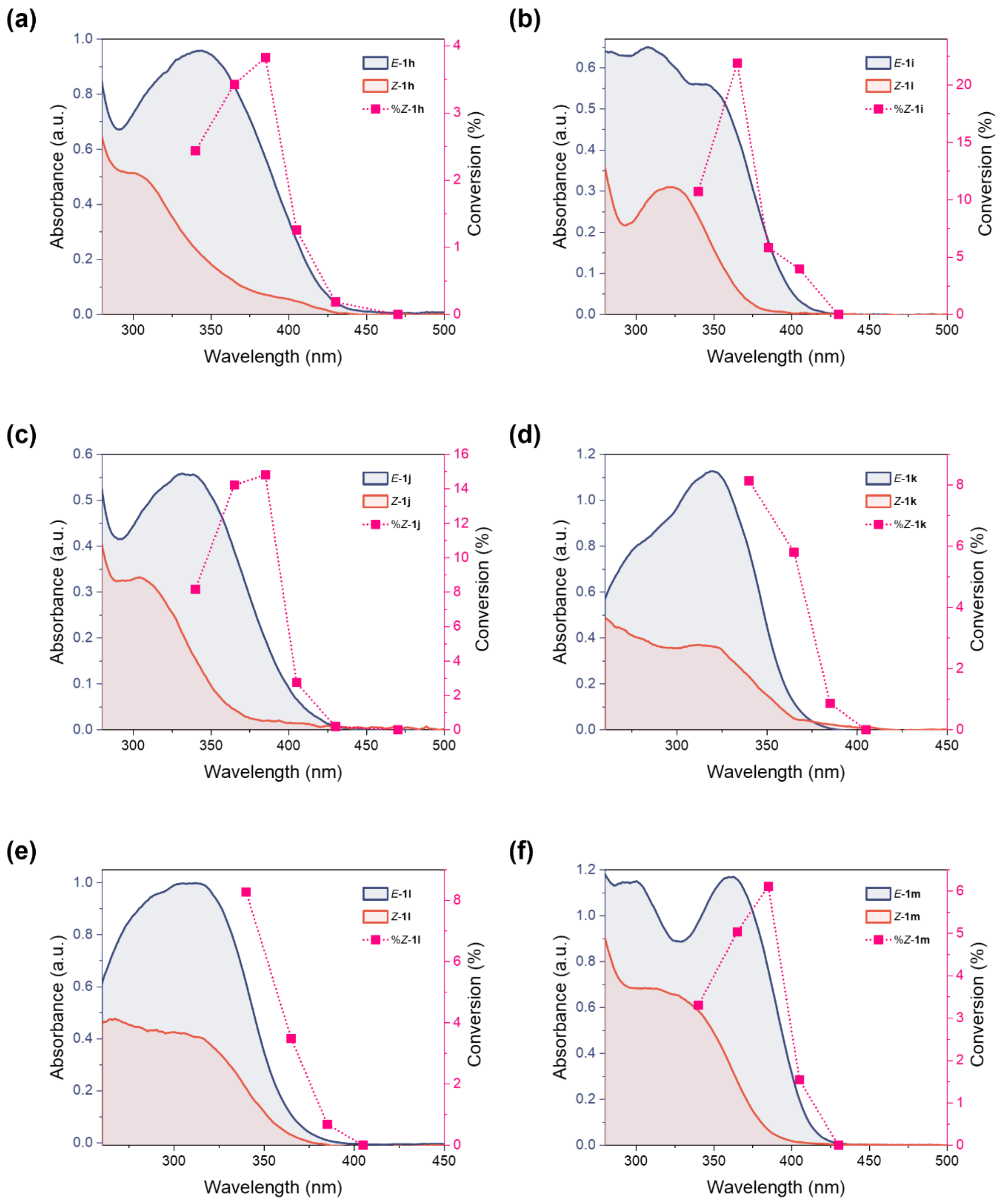

Figure S54. Action plots of (a) **1h**, (b) **1i**, (c) **1j**, (d) **1k**, (e) **1l**, and (f) **1m**. The conversions to *Z*-isomers are calculated for each irradiation wavelength when 8.5×10^{15} photons are delivered into the system according to the photon fluxes listed in Table S7. Spectra of *E*- and *Z*- isomers are presented for comparison.

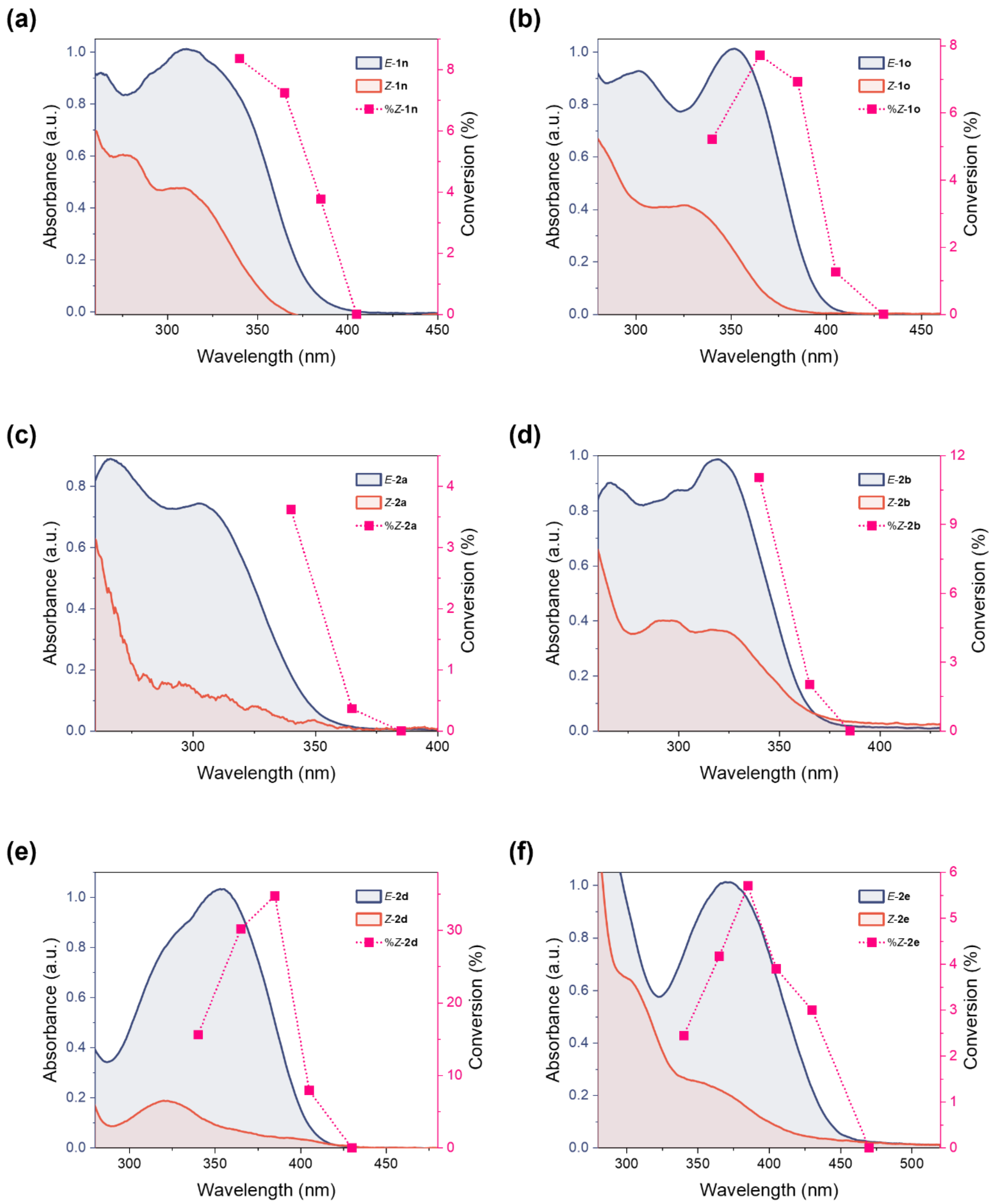

Figure S55. Action plots of (a) **1n**, (b) **1o**, (c) **2a**, (d) **2b**, (e) **2d**, and (f) **2e**. The conversions to *Z*-isomers are calculated for each irradiation wavelength when 8.5×10^{15} photons are delivered into the system according to the photon fluxes listed in Table S7. Spectra of *E*- and *Z*- isomers are presented for comparison.

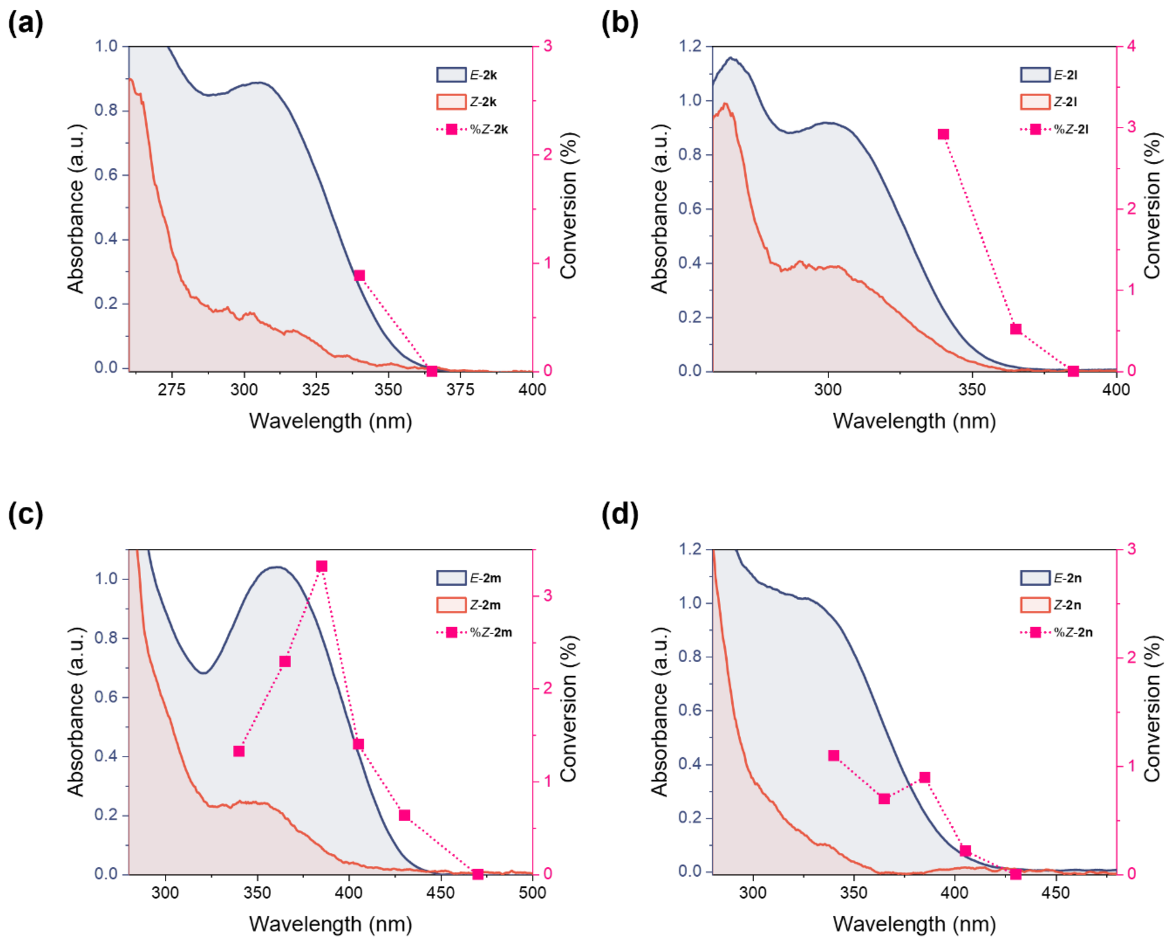

Figure S56. Action plots of (a) **2k**, (b) **2l**, (c) **2m**, and (d) **2n**. The conversions to *Z*-isomers are calculated for each irradiation wavelength when 8.5×10^{15} photons are delivered into the system according to the photon fluxes listed in Table S7. Spectra of *E*- and *Z*- isomers are presented for comparison.

4 Thin-film Studies

Thin films of the photoswitches with the longest $t_{1/2}$ (**1f** and **1j**) were prepared by spin coating. These switches were selected for their thermal stability, ensuring minimal thermally-induced *Z*-to-*E* back isomerism took place over the spectra measurement (approximately 2 minutes, PerkinElmer Lambda 950 spectrophotometer equipped with a 150 mm integration sphere).

Spin coating was performed using 10 mg mL^{-1} solutions of imines in DCM (anhydrous grade, Sigma Aldrich) under ambient conditions. A solution volume of 200 µL was applied using a static dispense method at 1000 rpm for 30 s. The resulting films were then thermally annealed at 80°C for 30 minutes before measurement (under an ambient atmosphere). The resulting films were translucent.

Note that for **1f**, a cycle of 405 nm and 80°C switching, as shown in the inset of Figure 10b in the main manuscript, shows no indication of degradation after two cycles. However, the use of 340 nm light to trigger the *Z*-to-*E* isomerism does show a degree of degradation attributed to the use of the high-energy UV light. These measurements were performed in an air-conditioned laboratory with an inside temperature set to 20°C.

To rule out the possibility that the spectrometer's probe light also induces photoswitching, $t_{1/2}$ measurements were performed at different intervals (every ca. 45 minutes compared to 1 minute used in Figures S59 and S60). No discrepancies in the $t_{1/2}$ from these two-time interval measurements were apparent.

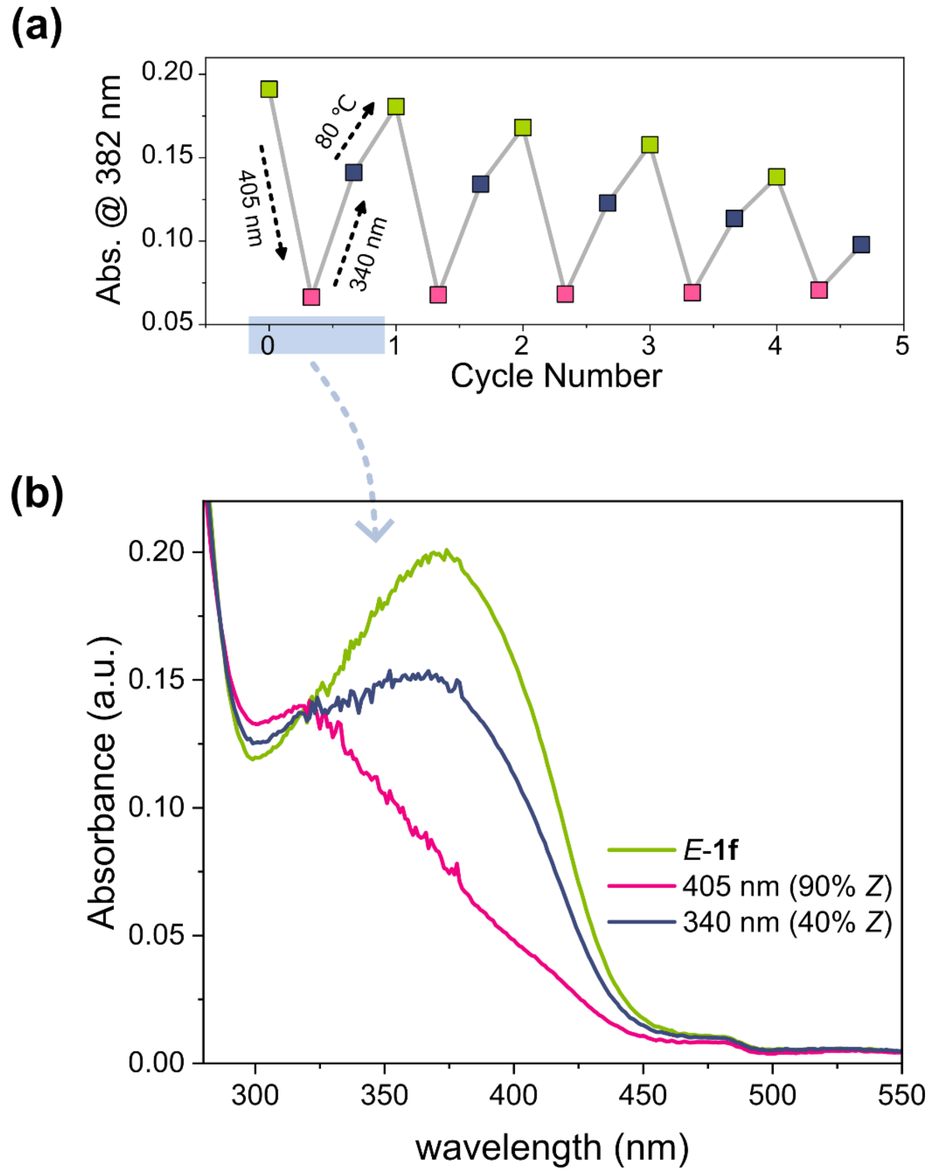

Figure S57. Condensed phase measurements of imine **1f**. The thin film was prepared by spin-coating on a quartz substrate, and the data was collected using PerkinElmer Lambda 950 spectrophotometer equipped with a 150 mm integration sphere. (a) Plots of the absorbance at 382 nm after sequential irradiation at 405 nm, 340 nm, and heating at 80°C, repeated for 5 cycles, (b) the UV/vis spectra of **1f** in condensed phase from the first cycle.

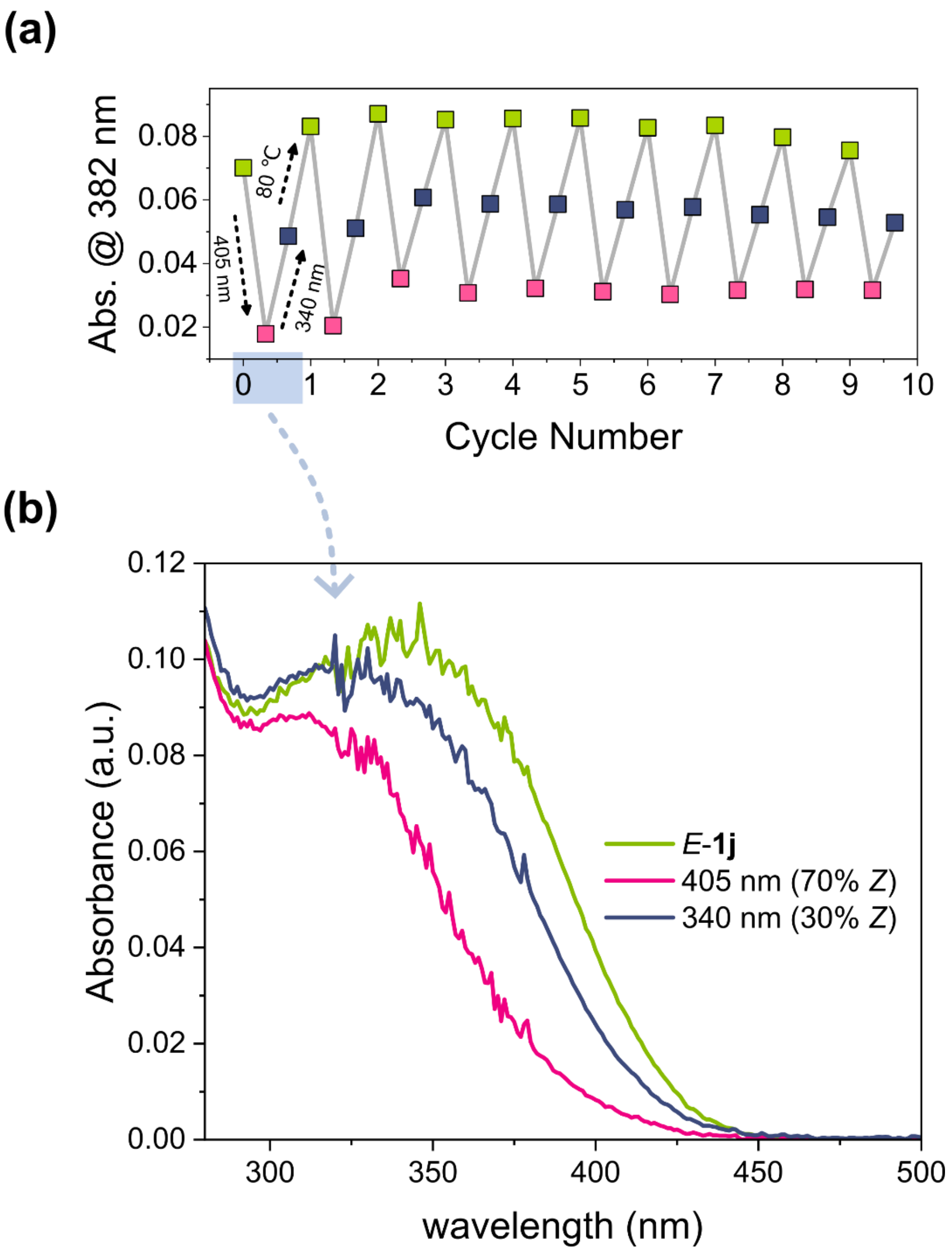

Figure S58. Condensed phase measurements of imine **1j**. The thin film was prepared by spin-coating on a quartz substrate, and the data was collected using PerkinElmer Lambda 950 spectrophotometer equipped with a 150 mm integration sphere. (a) Plots of the absorbance at 382 nm after sequential irradiation at 405 nm, 340 nm, and heating at 80°C, repeated for 10 cycles, (b) the UV/vis spectra of **1j** in condensed phase from the first cycle.

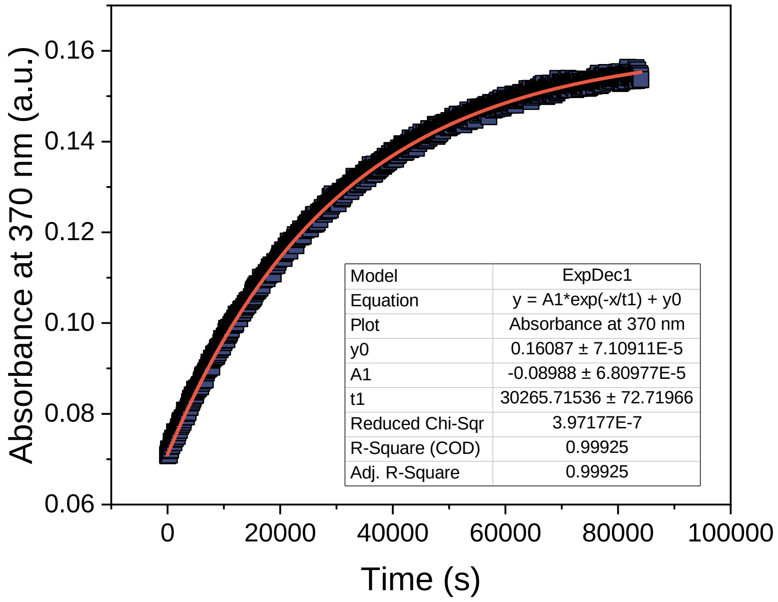

Figure S59. The change in absorbance of **1f** in the condensed phase at 370 nm overtime at 20°C, with the increase in absorbance relating to the thermal isomerization of the *Z*-isomer to the *E*-isomer. The sample was irradiated under 405 nm for 5 minutes before measurements started. The data was collected using PerkinElmer Lambda 950 spectrophotometer equipped with a 150 mm integration sphere. The $t_{1/2}$ of **1f** in the condensed phase is 5.8 hours.

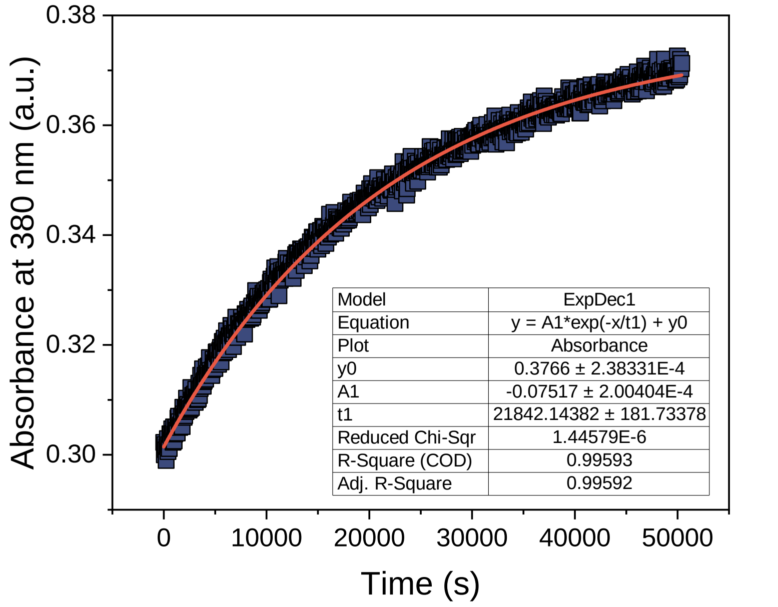

Figure S60. The change in absorbance of **1j** in the condensed phase at 380 nm overtime at 20°C, with the increase in absorbance relating to the thermal isomerization of the *Z*-isomer to the *E*-isomer. The sample was irradiated under 405 nm for 5 minutes before measurements started. The data was collected using PerkinElmer Lambda 950 spectrophotometer equipped with a 150 mm integration sphere. The $t_{1/2}$ of **1j** in the condensed phase is 4.2 hours.

5 Computational Studies

All underlying (TD-)DFT calculations were performed using the ORCA 5.0.4 software.[9–11] Other programs we used as part of our approach are CREST 2.12[12,13] and CENSO 1.2.1[14] for conformational search, NCIPLOT 4.0[15–17] for intramolecular interaction analysis. Images of molecules were created with VMD 1.9.2[18] and Pymol.

Conformer Search and Geometry Optimisation of *E* and *Z* Isomers and Transition State

We investigated the thermally accessible conformers of the imine switches at room temperature using the Conformer-Rotamer Ensemble Sampling Tool (CREST). Based on metadynamics simulations[13] and stepwise optimization at semiempirical GFN2-xTB level,[19–22] it is suitable for finding low-energy conformers quickly and efficiently. The identified conformers were then refined using the Commandline Energetic Sorting (CENSO) algorithm. This process ranked the conformers based on their free energy at DFT-level of theory and optimised their geometries further using the meta-generalized-gradient approximation (mGGA) functional r^2SCAN-3c in combination with a def2-mTZVPP basis set.[23] The geometries of the lowest energy *E* and *Z* conformers were further optimised at ωB97X-D4/def2-TZVPP level of theory with a CPCM solvation model for acetonitrile.[24–30] Ground-state equilibrium geometries of the *E* and *Z* isomers were confirmed by the absence of imaginary vibrational frequencies.

In previous studies,[1] we found that the *E*-*Z* isomerization of imines occurs via inversion of the C-N=C bond. Having identified these structures for all investigated imines, a conformational search of the transition state was applied using CREST. Therefore, the TS mode was fixed (α = 180°) and conformers were searched as described above. The lowest-energy conformers were subsequently optimized at ωB97X-D4/def2-TZVPP level and the resulting transition state with the lowest energy was selected for the following calculations. All transition states were confirmed by the presence of a single imaginary vibrational frequency.

Theoretical Investigation of Thermal Isomerisation Rates and Half-Lives

The rate of thermal isomerisation from the metastable *Z*-isomer to the thermodynamically stable *E*-isomer via the optimised transition state was approximated using Eyring theory.[5] The rate, *k*, of *Z* to *E* isomerisation is given by equation (1),

$$k_{Z\to E}(T) = \frac{k_B T}{h} \cdot e^{\frac{-\Delta G^{\ddagger}}{RT}} \qquad (1)$$

where k_B is the Boltzmann constant, T is the temperature in Kelvin, h is the Planck constant, $\Delta G^{\ddagger}$ is the difference in the Gibbs energy of the *Z*-isomer and the transition state, and R is the gas constant. Once the rate is known, the thermal half-life, $t_{1/2}$, can be calculated from

$$t_{1/2} = \frac{ln\, 2}{k_{Z\to E}} \qquad (2)$$

Noncovalent Interaction (NCI) Analysis

Noncovalent interaction analysis was used to rationalize relationships between the geometries and thermal half-lives of the imine switches. This approach provides a visual representation of weak intramolecular interactions in the low-density regime. NCI analysis was performed using the NCIPLOT 4.0 program starting from the electron density of the optimized *E*, *Z* and TS structures obtained at ωB97X-D4/def2-TZVPP level.

Table S9. Summary of the theoretical data obtained for the AIPs.

	$\Delta G^{‡}$/kJ mol^{-1}	$t_{1/2}$/hh:mm:ss	ΔG_{Z-E}/kJ mol^{-1}	$E^{D4}{}_{Z-E}$ /kJ mol^{-1}	$E^{D4}{}_{TS-E}$ /kJ mol^{-1}
**1a**	78.99	0:00:13	21.48	-7.04	0.80
**1b**	80.17	0:00:22	24.72	-6.90	0.90
**1c**	77.18	0:00:06	24.55	-7.30	0.77
**1e**	97.28	6:56:12	10.38	-18.15	-5.10
**1f**	107.42	444:45:24	-4.24	-12.27	-7.97
**1g**	90.93	0:30:40	16.32	-7.70	1.85
**1h**	101.99	47:55:17	0.85	-16.81	-4.63
**1i**	90.92	0:30:36	19.25	-8.00	2.14
**1j**	110.50	1578:42:58	-3.47	-14.06	-5.55
**1k**	81.33	0:00:35	22.48	-7.59	0.67
**1l**	89.52	0:17:12	9.08	-8.40	0.36
**1m**	100.98	31:42:37	2.13	-14.77	-3.07
**1n**	98.03	9:26:54	5.60	-8.45	2.47
**1o**	100.37	24:38:39	3.28	-16.77	-2.94
**2a**	82.32	0:00:53	8.00	-8.40	2.09
**2b**	90.47	0:25:26	9.04	-8.71	2.25
**2c**	64.93	0:00:00	6.69	-7.85	1.84
**2d**	93.88	1:43:02	9.60	-9.81	2.14
**2e**	83.98	0:01:46	10.70	-9.28	-0.54
**2f**	87.11	0:06:24	4.93	-10.28	-4.95
**2k**	82.80	0:01:05	7.63	-8.52	1.78
**2l**	83.40	0:01:23	5.10	-8.30	1.80
**2m**	85.24	0:02:57	6.54	-8.64	0.45
**2n**	79.68	0:00:18	6.23	-9.65	0.49

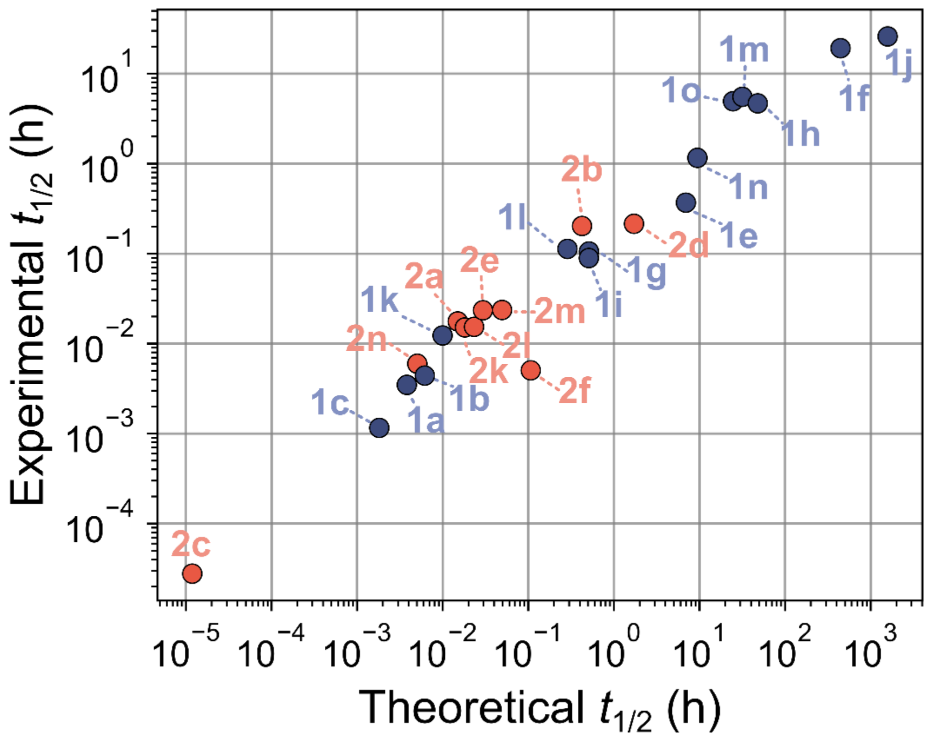

Figure S61. Plot showing the linear trend observed between the experimentally determined and theoretically calculated $t_{1/2}$ values at 20 °C of the *N*-pyrazole and *N*-phenyl AIPs.

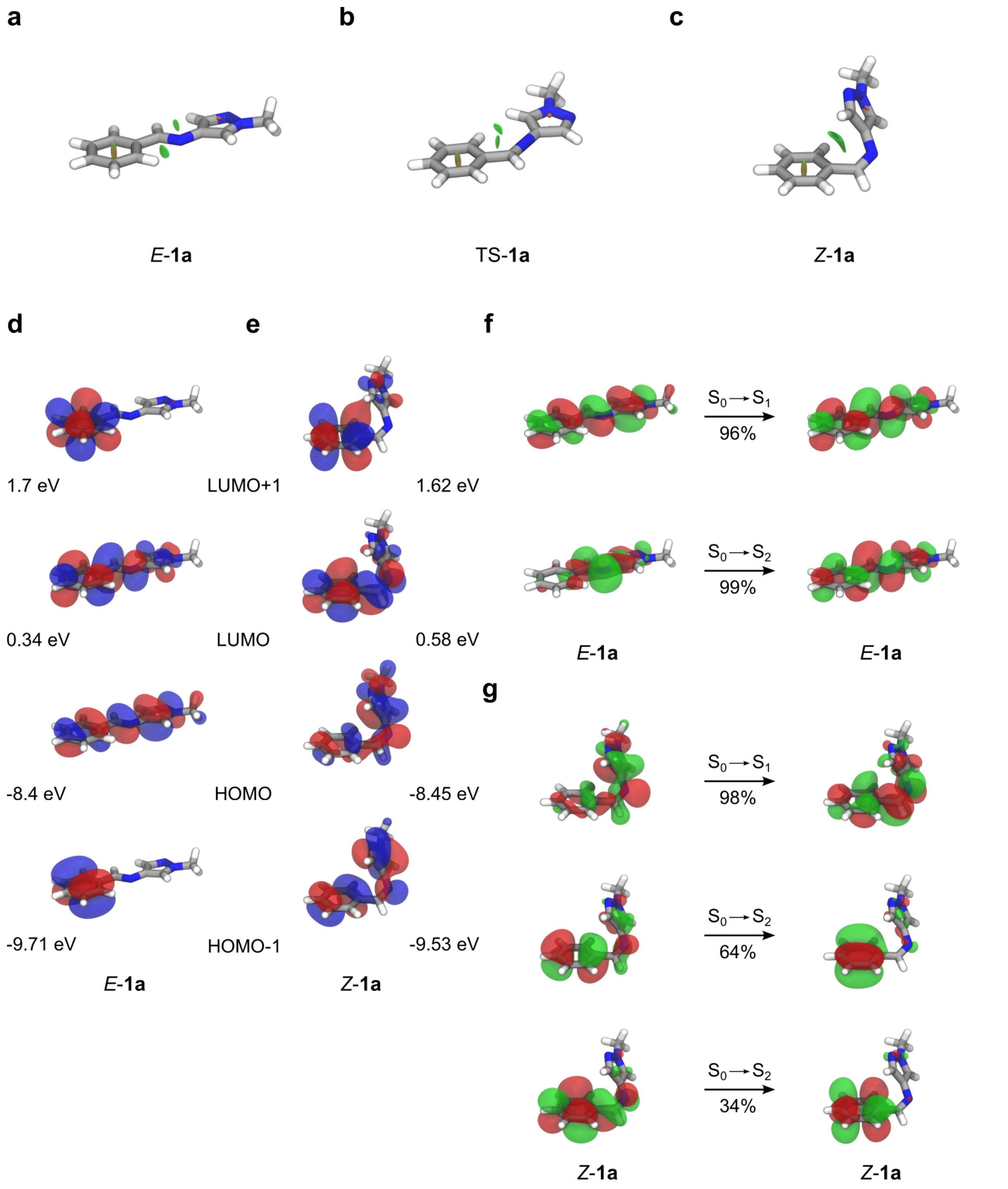

Figure S62. Overview of the theoretically calculated properties of **1a**. The geometry optimized structures, along with calculate non-covalent interaction (NCI) surfaces of (a) *E*-**1a**, (b) TS-**1a**, and (c) *Z*-**1a**. Frontier molecular orbitals (FMOs) of (d) *E*-**1a** and (e) *Z*-**1a** and corresponding energies. NTO pairs ("hole" → "particle") of the $S_0 \rightarrow S_1$ and $S_0 \rightarrow S_1$ transitions of (f) *E*-**1a** and (g) *Z*-**1a** with percentage contributions stated.

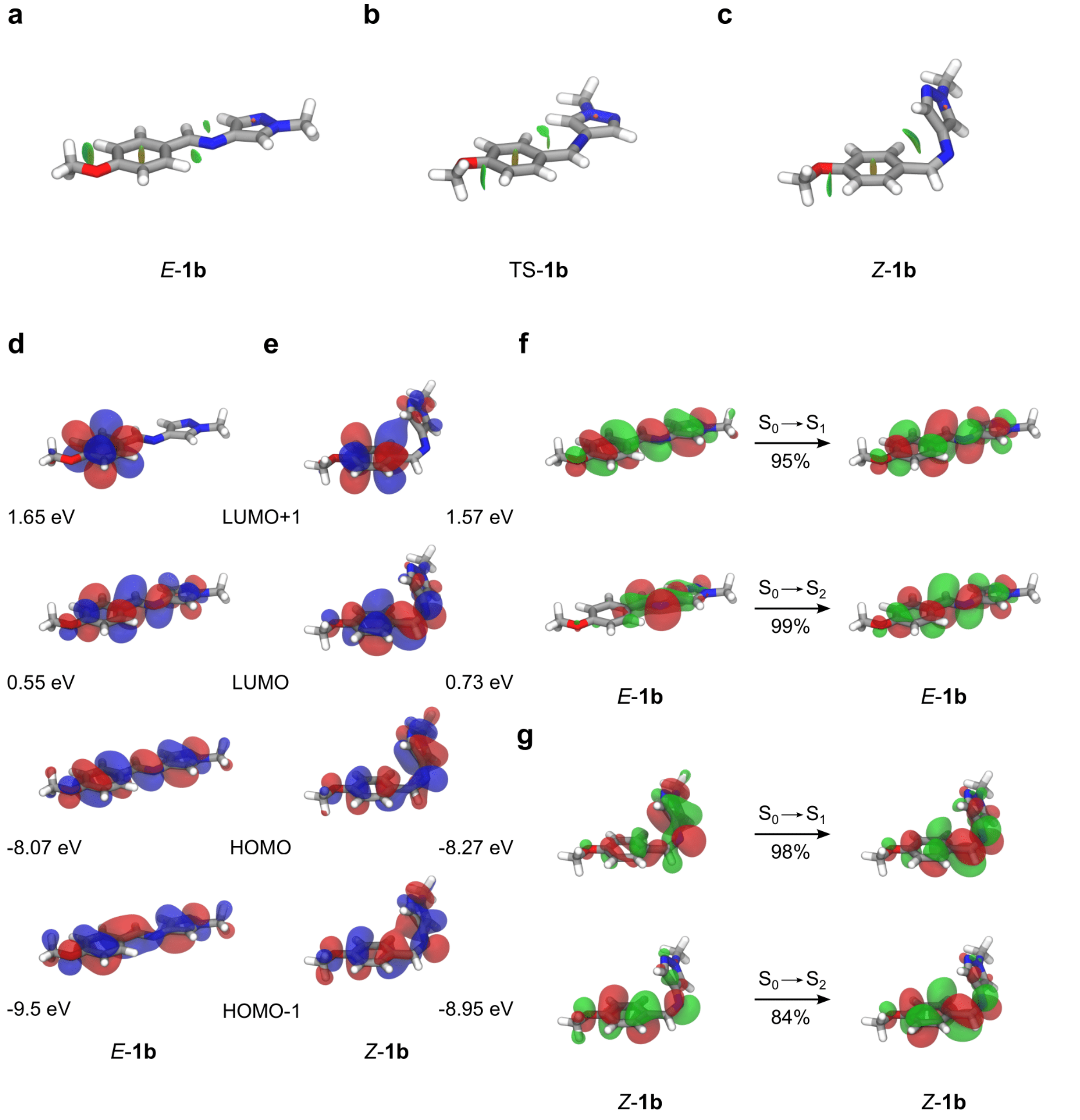

Figure S63. Overview of the theoretically calculated properties of **1b**. The geometry optimized structures, along with calculated non-covalent interaction (NCI) surfaces of (a) *E*-**1b**, (b) TS-**1b**, and (c) *Z*-**1b**. Frontier molecular orbitals (FMOs) of (d) *E*-**1b** and (e) *Z*-**1b** and corresponding energies. NTO pairs (“hole” → “particle”) of the $S_0 \rightarrow S_1$ and $S_0 \rightarrow S_1$ transitions of (f) *E*-**1b** and (g) *Z*-**1b** with percentage contributions stated.

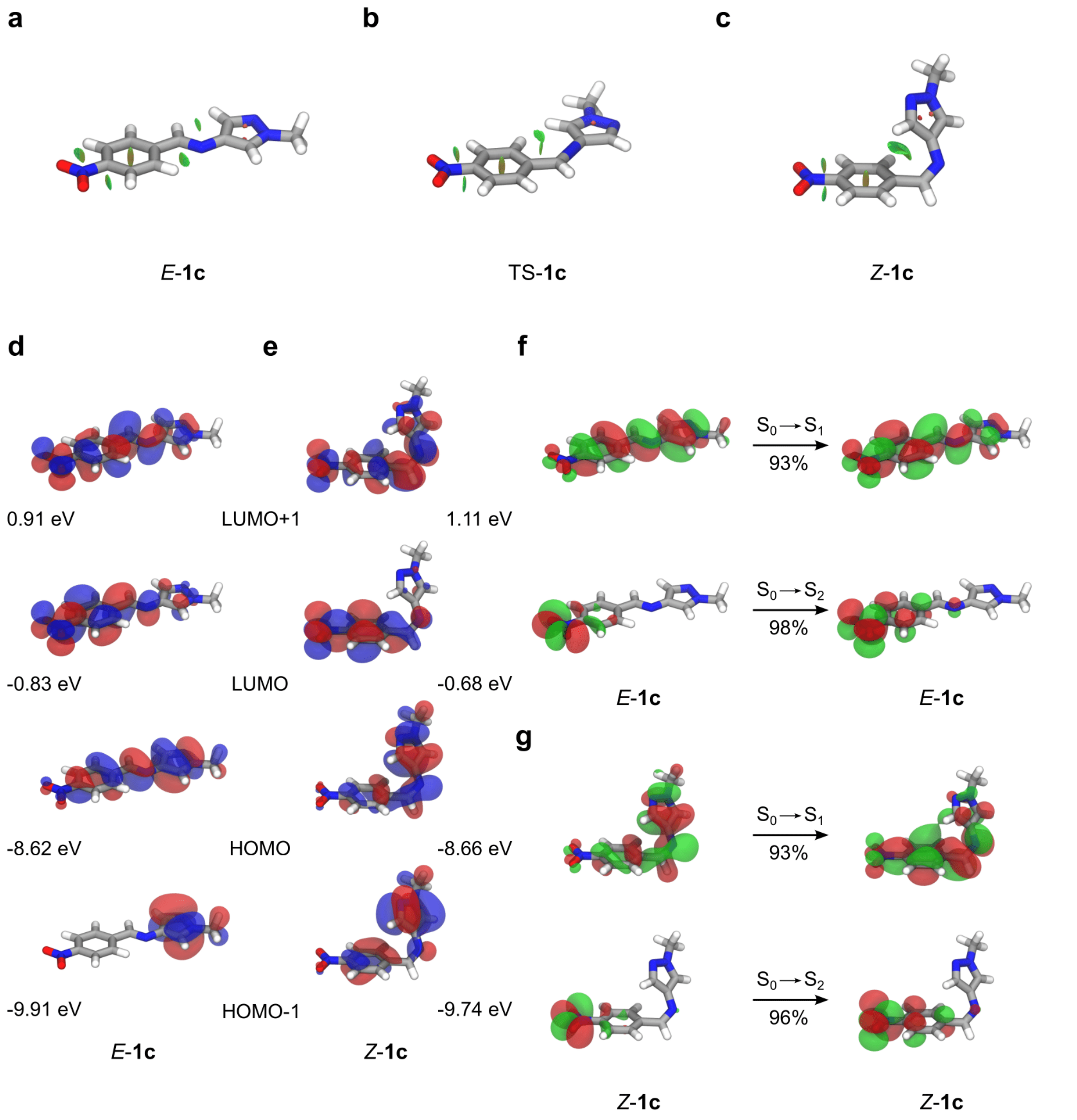

Figure S64. Overview of the theoretically calculated properties of **1c**. The geometry optimized structures, along with calculated non-covalent interaction (NCI) surfaces of (a) *E*-**1c**, (b) TS-**1c**, and (c) *Z*-**1c**. Frontier molecular orbitals (FMOs) of (d) *E*-**1c** and (e) *Z*-**1c** and corresponding energies. NTO pairs ("hole" → "particle") of the $S_0 \rightarrow S_1$ and $S_0 \rightarrow S_1$ transitions of (f) *E*-**1c** and (g) *Z*-**1c** with percentage contributions stated.

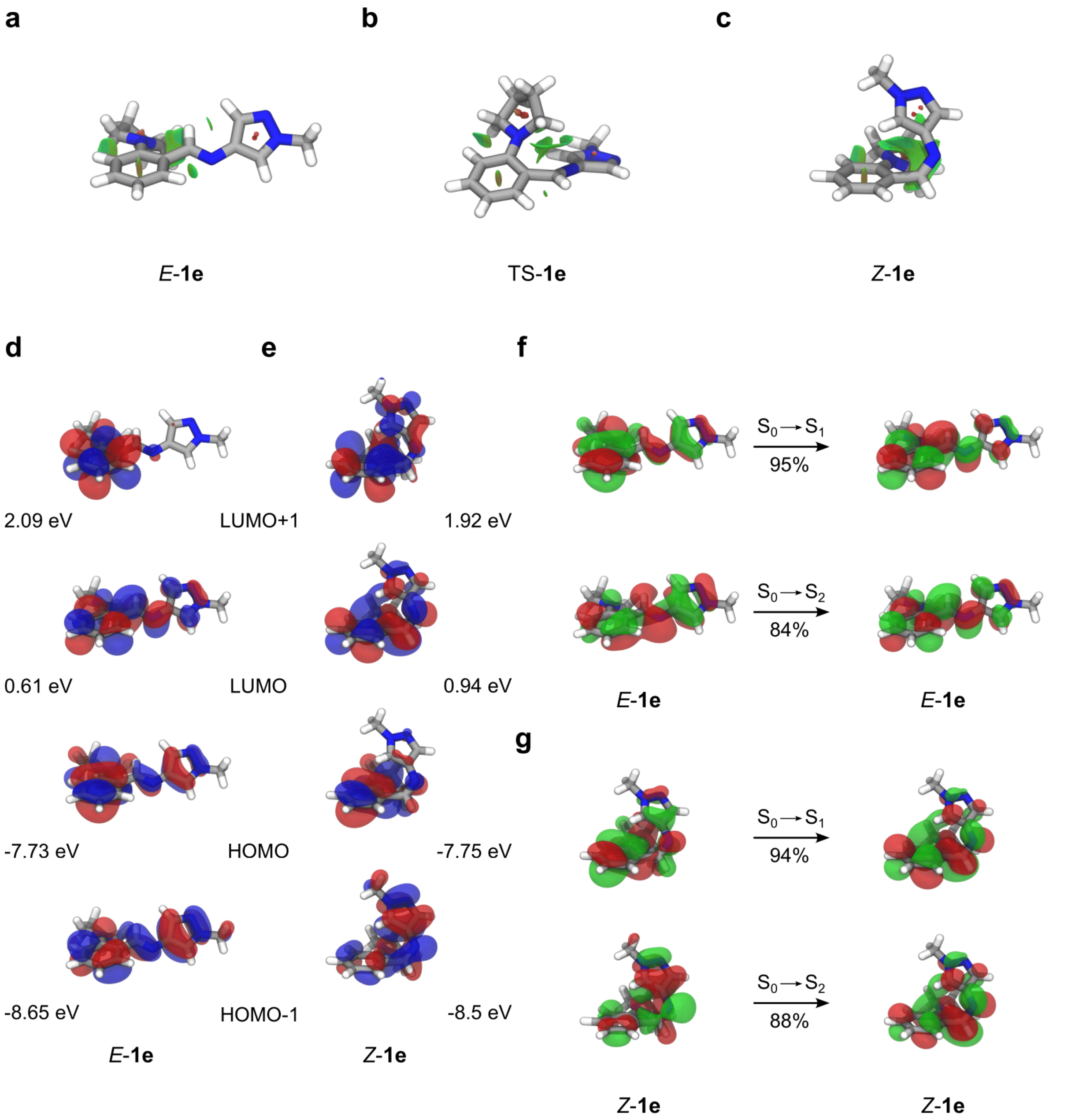

Figure S65. Overview of the theoretically calculated properties of **1e**. The geometry optimized structures, along with calculated non-covalent interaction (NCI) surfaces of (a) *E*-**1e**, (b) TS-**1e**, and (c) *Z*-**1e**. Frontier molecular orbitals (FMOs) of (d) *E*-**1e** and (e) *Z*-**1e** and corresponding energies. NTO pairs ("hole" → "particle") of the S_0→S_1 and S_0→S_1 transitions of (f) *E*-**1e** and (g) *Z*-**1e** with percentage contributions stated.

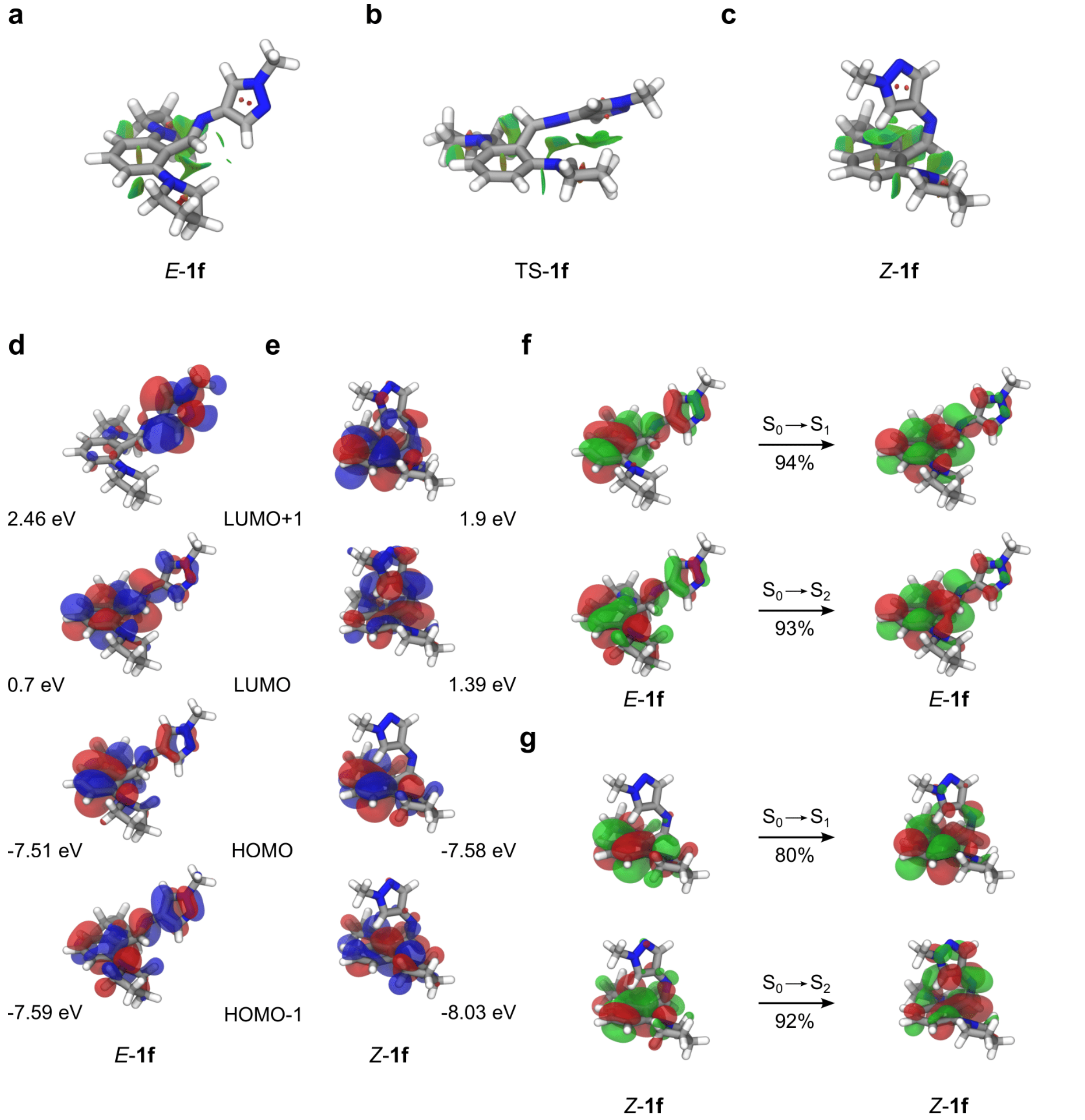

Figure S66. Overview of the theoretically calculated properties of **1f**. The geometry optimized structures, along with calculated non-covalent interaction (NCI) surfaces of (a) *E*-**1f**, (b) TS-**1f**, and (c) *Z*-**1f**. Frontier molecular orbitals (FMOs) of (d) *E*-**1f** and (e) *Z*-**1f** and corresponding energies. NTO pairs ("hole" → "particle") of the $S_0 \rightarrow S_1$ and $S_0 \rightarrow S_1$ transitions of (f) *E*-**1f** and (g) *Z*-**1f** with percentage contributions stated.

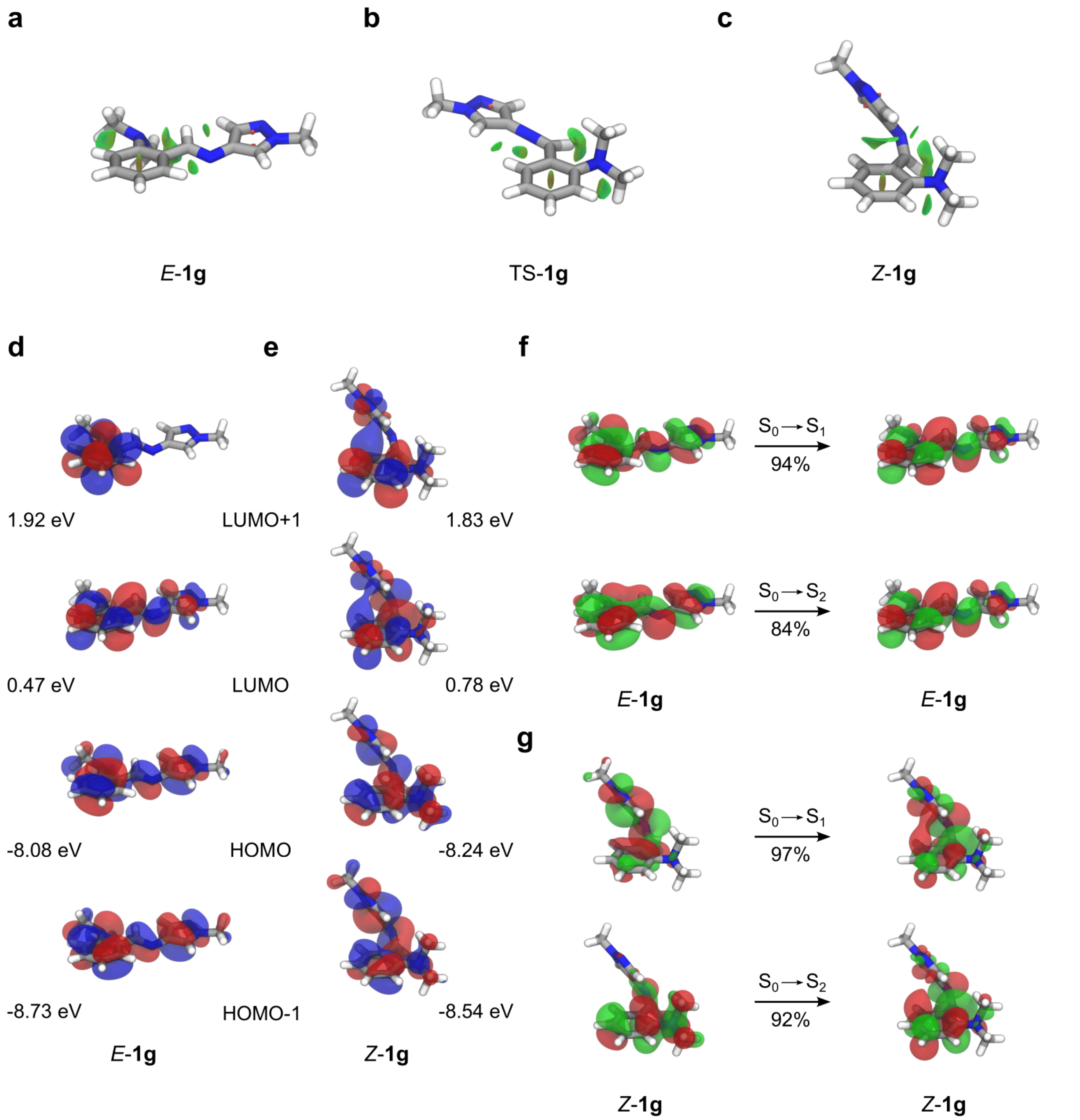

Figure S67. Overview of the theoretically calculated properties of **1g**. The geometry optimized structures, along with calculated non-covalent interaction (NCI) surfaces of (a) *E*-**1g**, (b) TS-**1g**, and (c) *Z*-**1g**. Frontier molecular orbitals (FMOs) of (d) *E*-**1g** and (e) *Z*-**1g** and corresponding energies. NTO pairs ("hole" → "particle") of the S_0→S_1 and S_0→S_1 transitions of (f) *E*-**1g** and (g) *Z*-**1g** with percentage contributions stated.

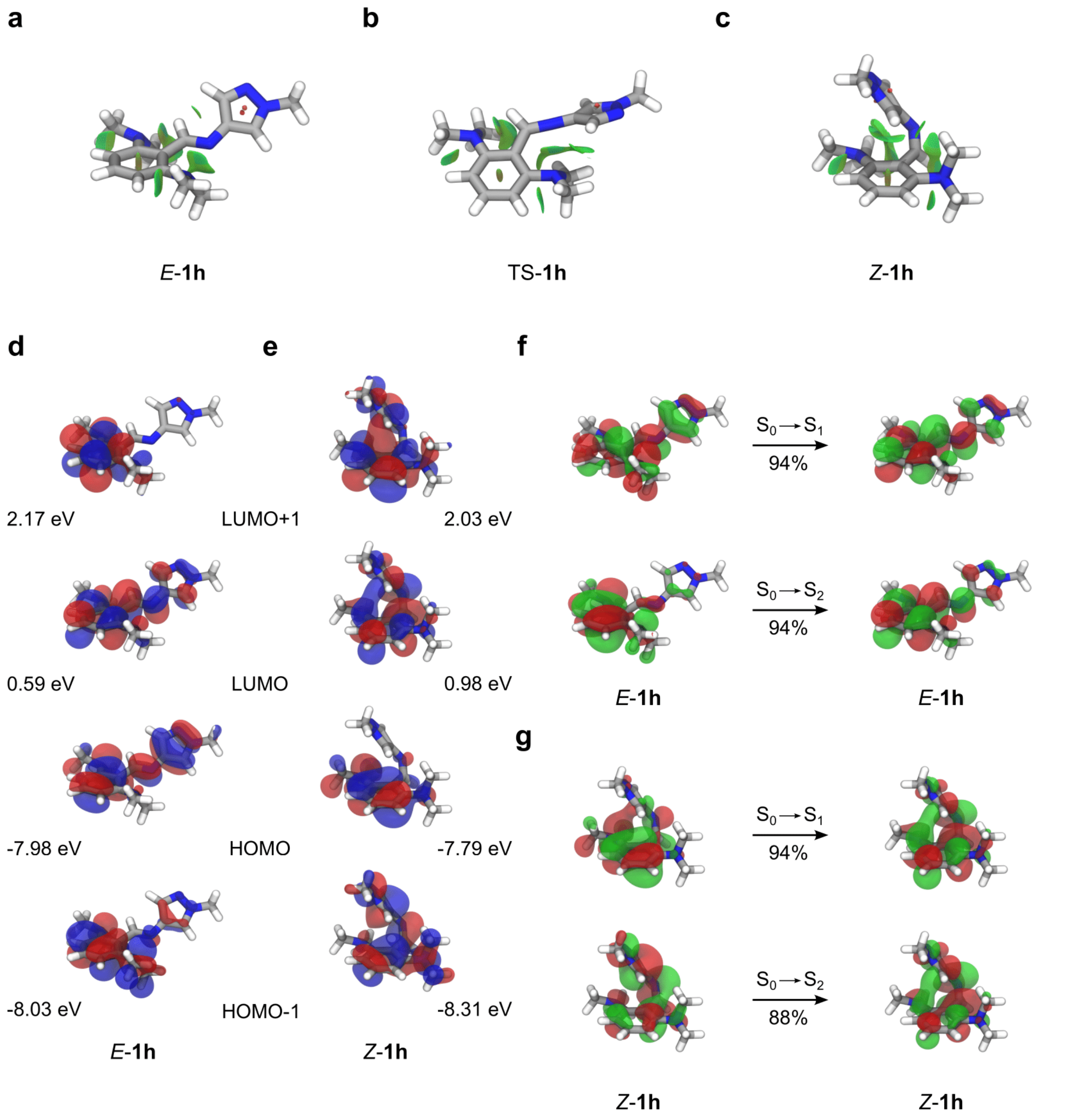

Figure S68. Overview of the theoretically calculated properties of **1h**. The geometry optimized structures, along with calculated non-covalent interaction (NCI) surfaces of (a) *E*-**1h**, (b) TS-**1h**, and (c) *Z*-**1h**. Frontier molecular orbitals (FMOs) of (d) *E*-**1h** and (e) *Z*-**1h** and corresponding energies. NTO pairs ("hole" → "particle") of the $S_0 \rightarrow S_1$ and $S_0 \rightarrow S_1$ transitions of (f) *E*-**1h** and (g) *Z*-**1h** with percentage contributions stated.

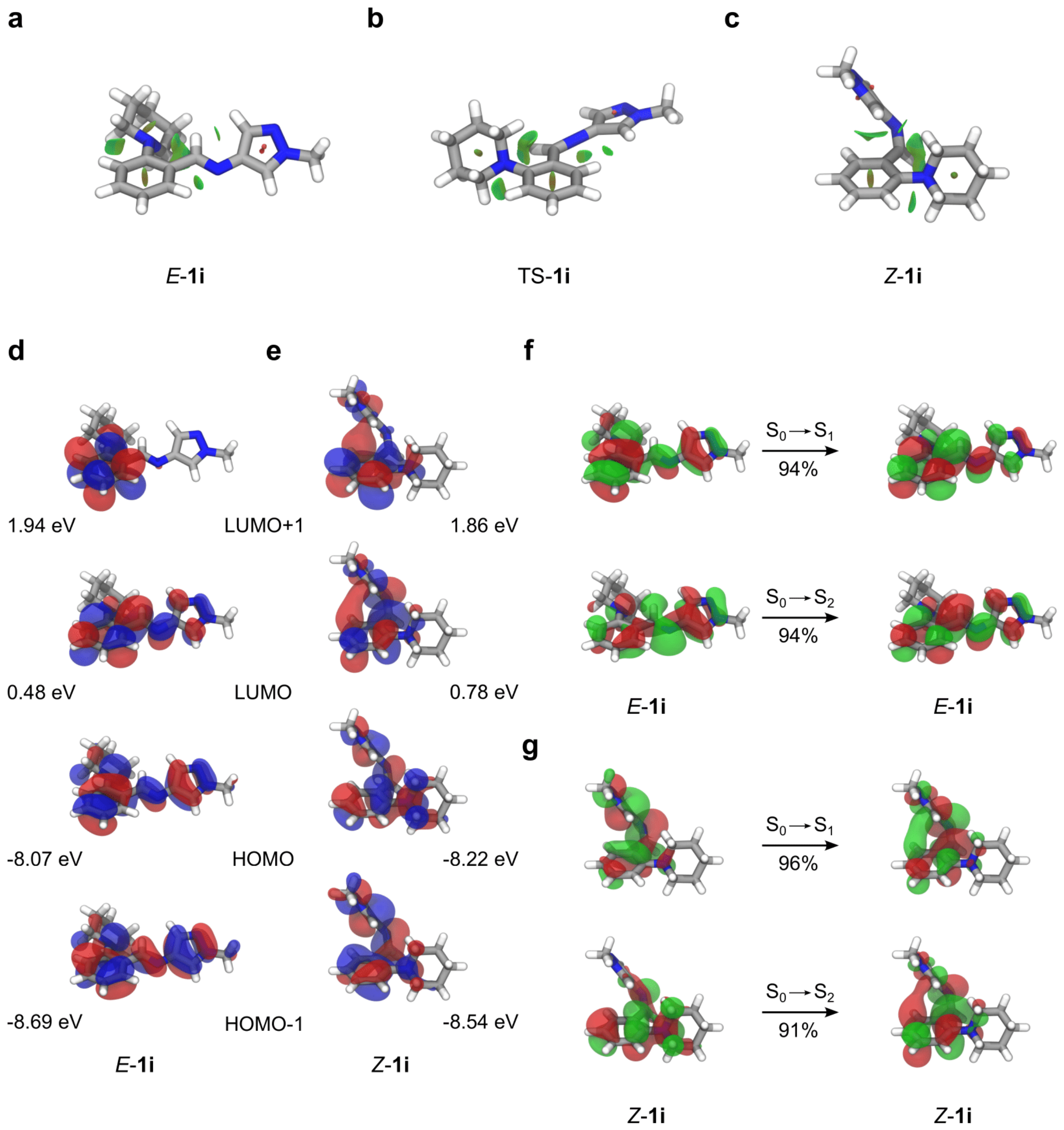

Figure S69. Overview of the theoretically calculated properties of **1i**. The geometry optimized structures, along with calculated non-covalent interaction (NCI) surfaces of (a) *E*-**1i**, (b) TS-**1i**, and (c) *Z*-**1i**. Frontier molecular orbitals (FMOs) of (d) *E*-**1i** and (e) *Z*-**1i** and corresponding energies. NTO pairs ("hole" → "particle") of the S_0→S_1 and S_0→S_1 transitions of (f) *E*-**1i** and (g) *Z*-**1i** with percentage contributions stated.

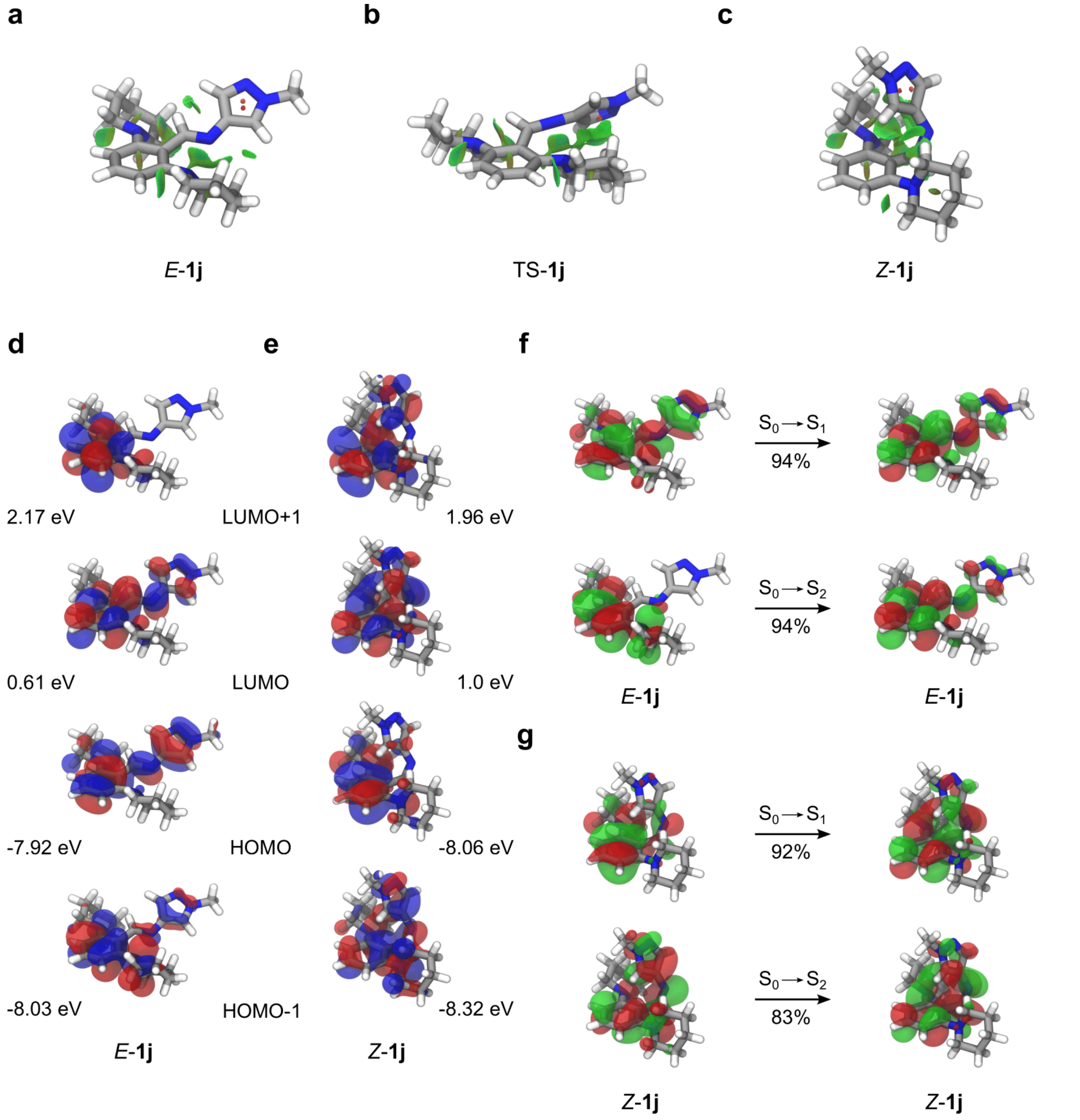

Figure S70. Overview of the theoretically calculated properties of **1j**. The geometry optimized structures, along with calculated non-covalent interaction (NCI) surfaces of (a) *E*-**1j**, (b) TS-**1j**, and (c) *Z*-**1j**. Frontier molecular orbitals (FMOs) of (d) *E*-**1j** and (e) *Z*-**1j** and corresponding energies. NTO pairs ("hole" → "particle") of the $S_0 \rightarrow S_1$ and $S_0 \rightarrow S_1$ transitions of (f) *E*-**1j** and (g) *Z*-**1j** with percentage contributions stated.

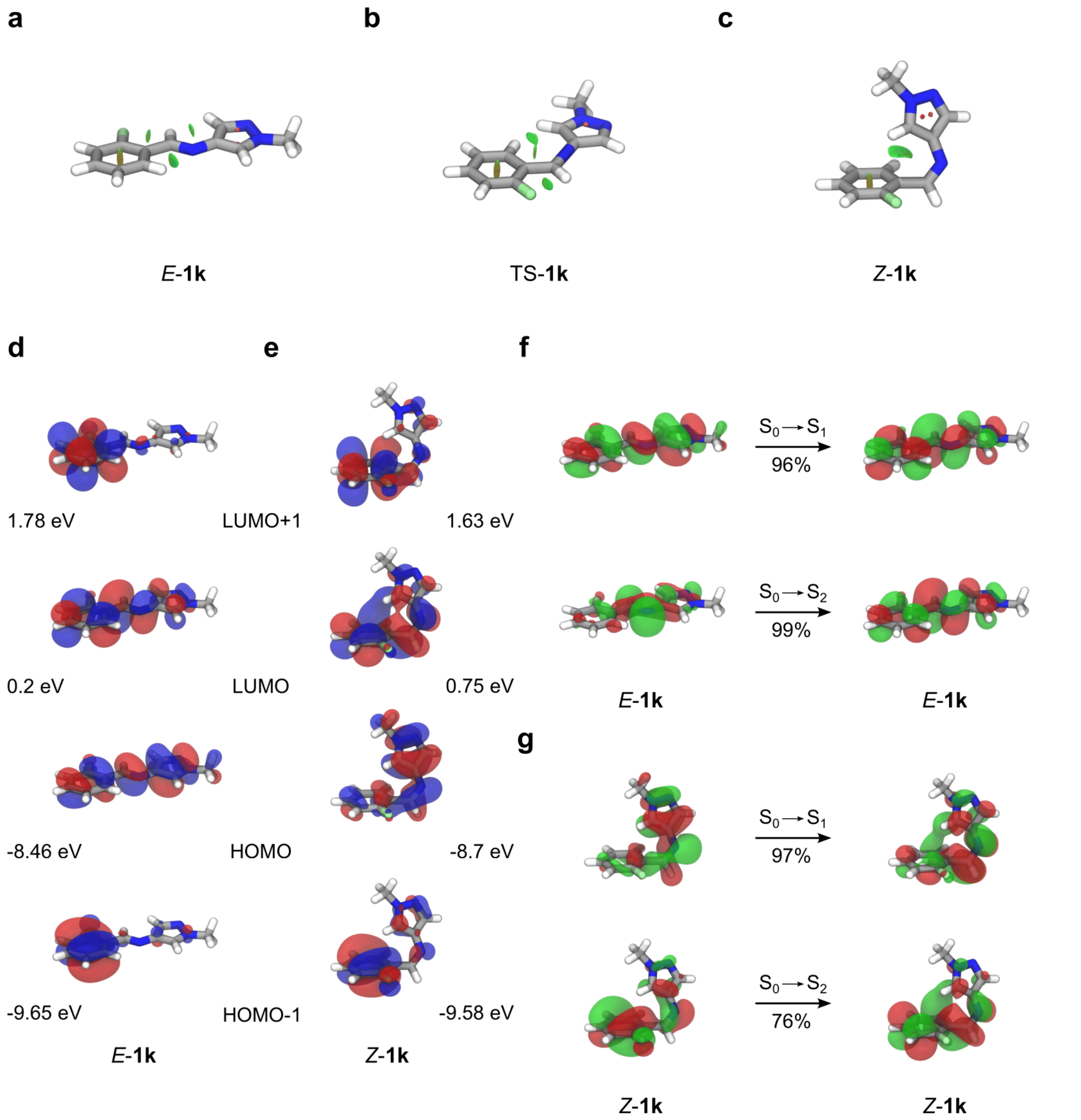

Figure S71. Overview of the theoretically calculated properties of **1k**. The geometry optimized structures, along with calculated non-covalent interaction (NCI) surfaces of (a) *E*-**1k**, (b) TS-**1k**, and (c) *Z*-**1k**. Frontier molecular orbitals (FMOs) of (d) *E*-**1k** and (e) *Z*-**1k** and corresponding energies. NTO pairs ("hole" → "particle") of the $S_0 \rightarrow S_1$ and $S_0 \rightarrow S_1$ transitions of (f) *E*-**1k** and (g) *Z*-**1k** with percentage contributions stated.

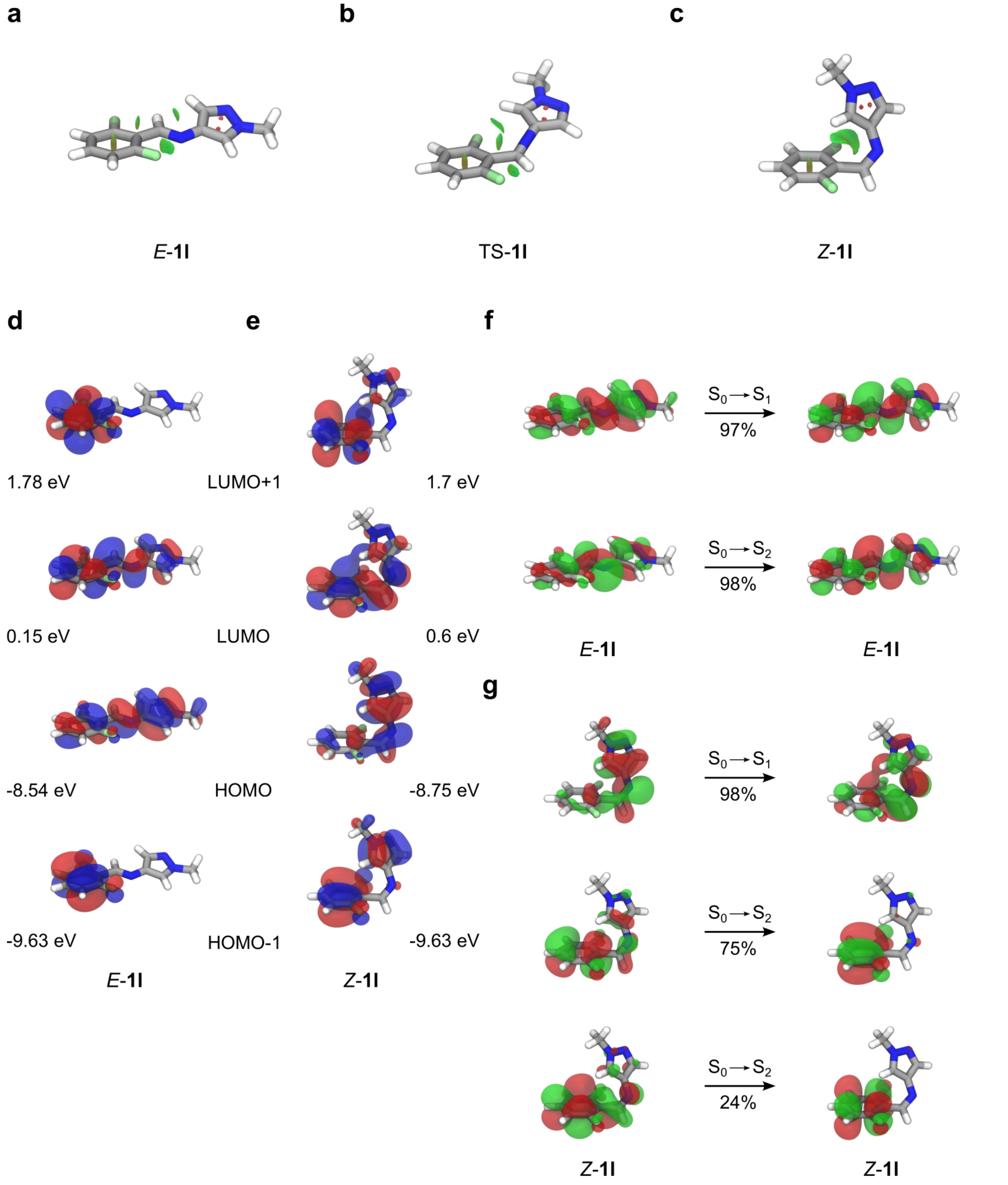

Figure S72. Overview of the theoretically calculated properties of **1l**. The geometry optimized structures, along with calculated non-covalent interaction (NCI) surfaces of (a) *E*-**1l**, (b) TS-**1l**, and (c) *Z*-**1l**. Frontier molecular orbitals (FMOs) of (d) *E*-**1l** and (e) *Z*-**1l** and corresponding energies. NTO pairs ("hole" → "particle") of the $S_0 \rightarrow S_1$ and $S_0 \rightarrow S_1$ transitions of (f) *E*-**1l** and (g) *Z*-**1l** with percentage contributions stated.

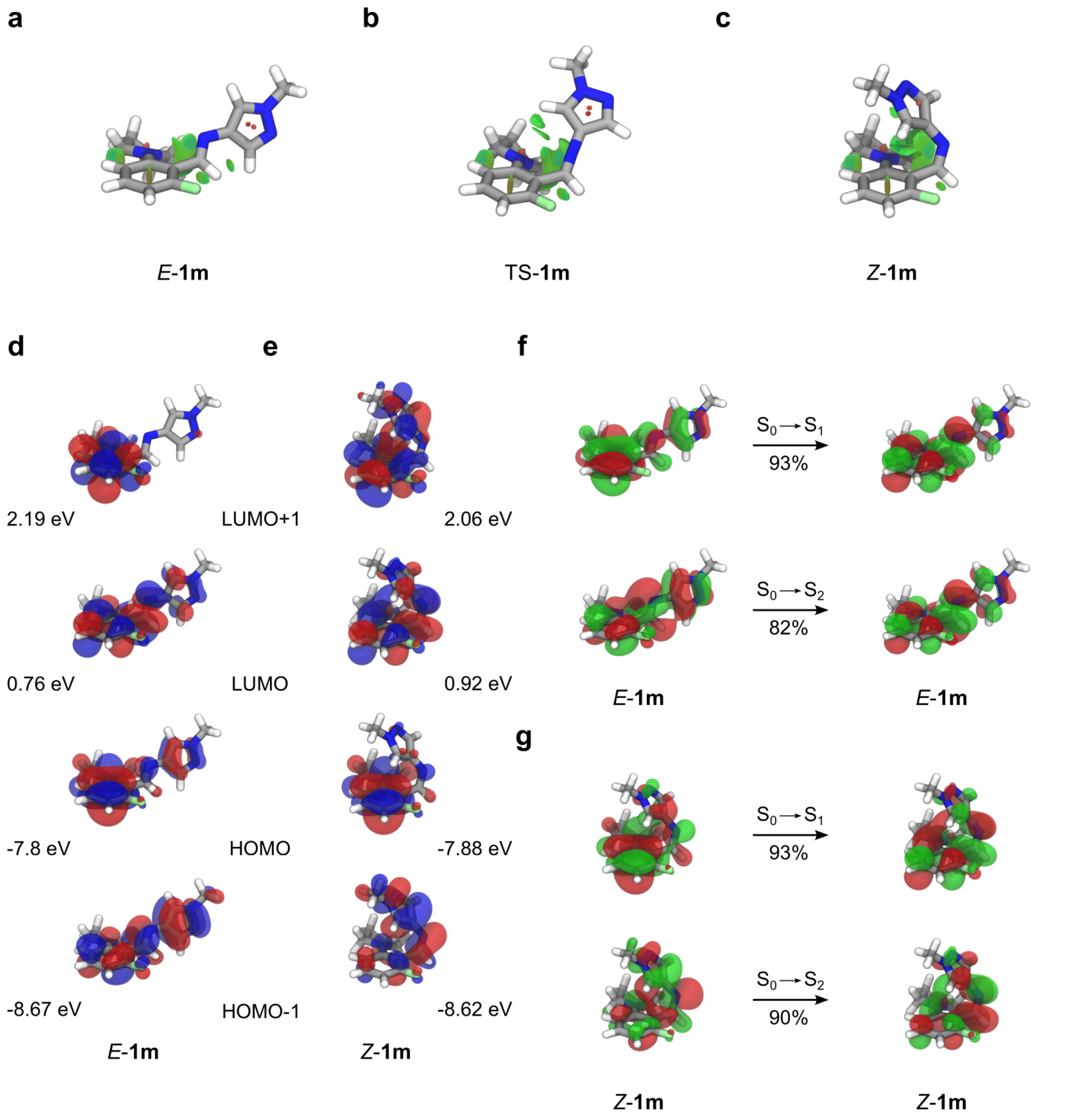

Figure S73. Overview of the theoretically calculated properties of **1m**. The geometry optimized structures, along with calculated non-covalent interaction (NCI) surfaces of (a) *E*-**1m**, (b) TS-**1m**, and (c) *Z*-**1m**. Frontier molecular orbitals (FMOs) of (d) *E*-**1m** and (e) *Z*-**1m** and corresponding energies. NTO pairs ("hole" → "particle") of the $S_0 \rightarrow S_1$ and $S_0 \rightarrow S_1$ transitions of (f) *E*-**1m** and (g) *Z*-**1m** with percentage contributions stated.

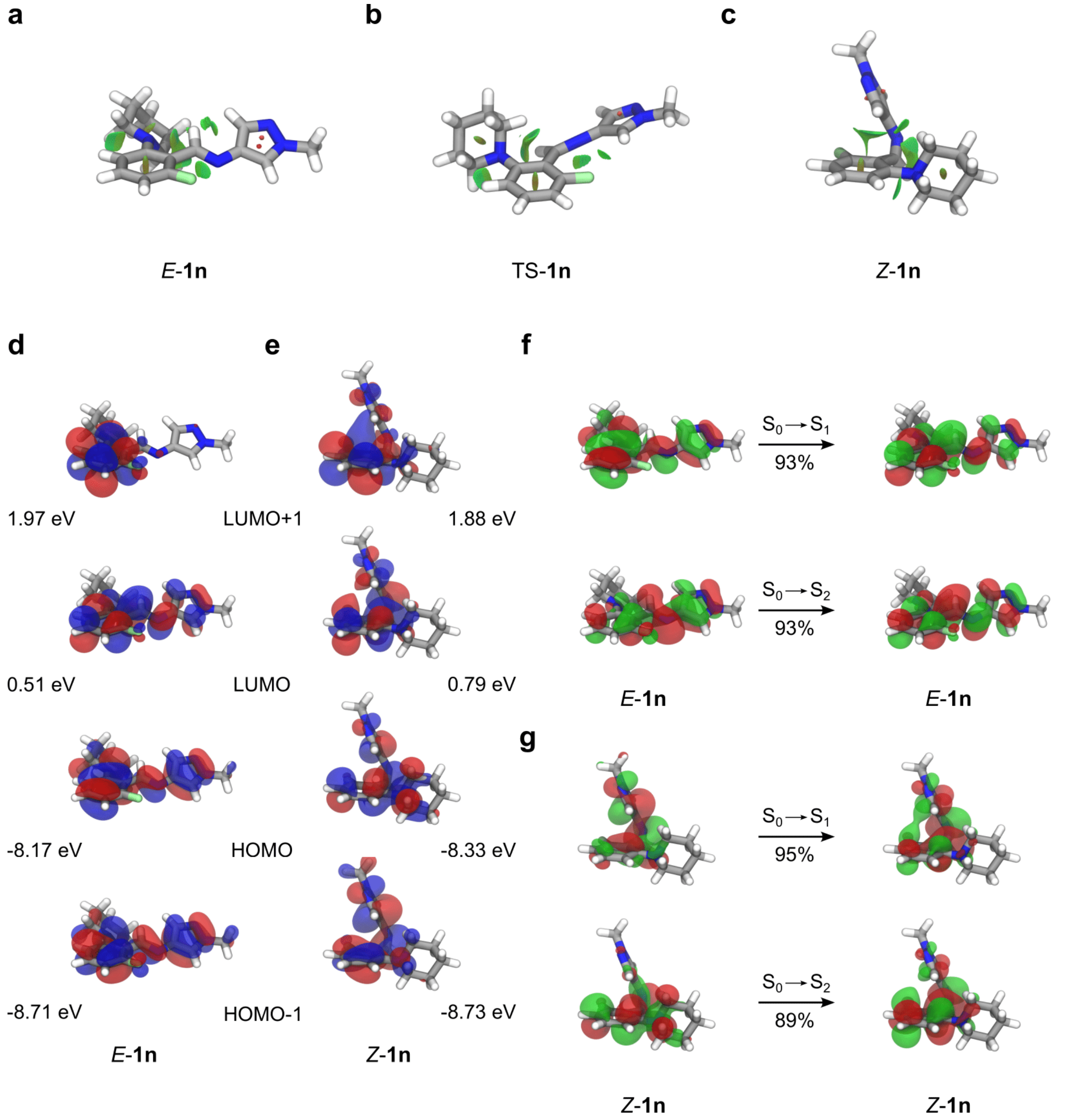

Figure S74. Overview of the theoretically calculated properties of **1n**. The geometry optimized structures, along with calculated non-covalent interaction (NCI) surfaces of (a) *E*-**1n**, (b) TS-**1n**, and (c) *Z*-**1n**. Frontier molecular orbitals (FMOs) of (d) *E*-**1n** and (e) *Z*-**1n** and corresponding energies. NTO pairs ("hole" → "particle") of the $S_0 \rightarrow S_1$ and $S_0 \rightarrow S_1$ transitions of (f) *E*-**1n** and (g) *Z*-**1n** with percentage contributions stated.

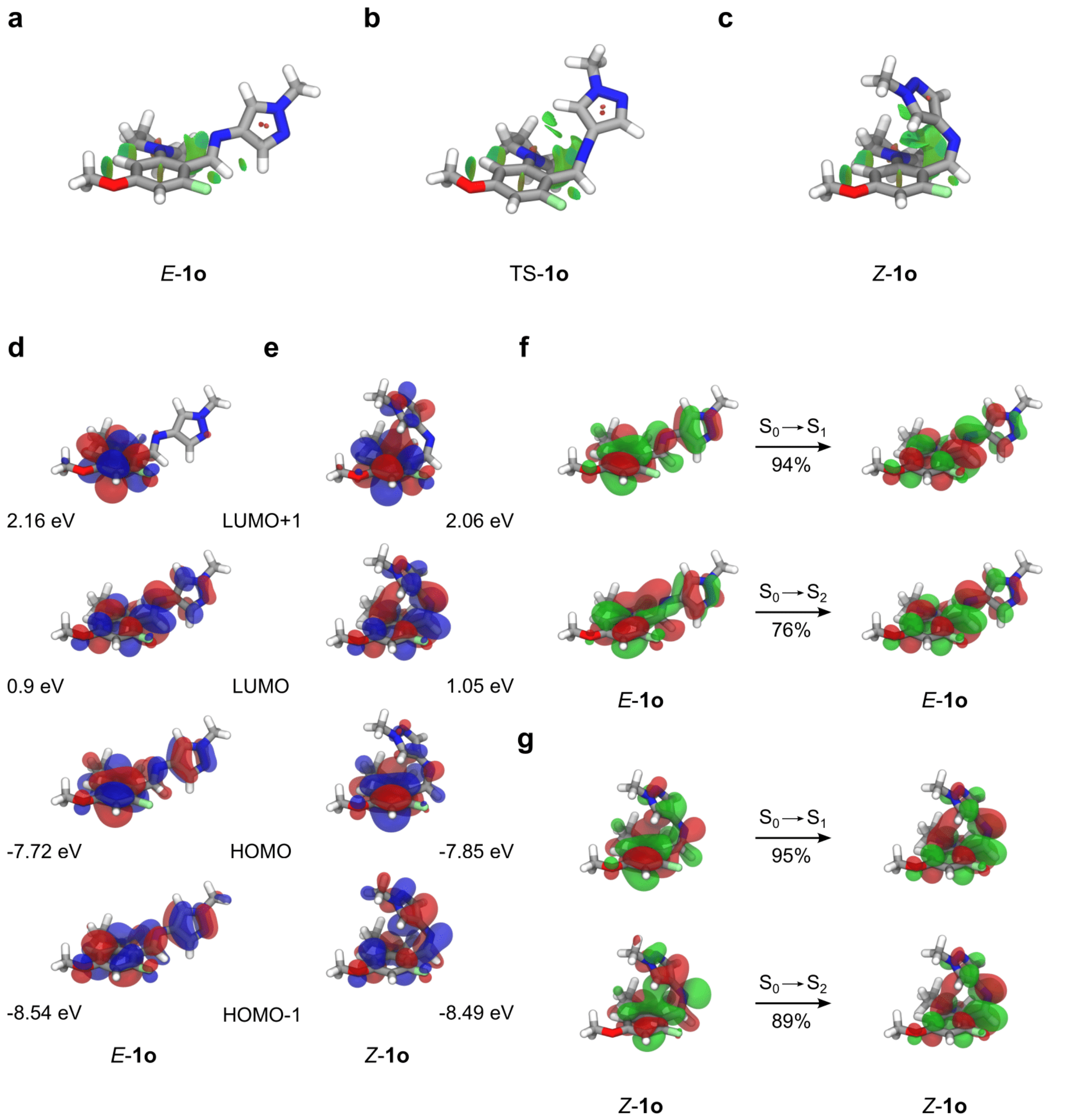

Figure S75. Overview of the theoretically calculated properties of **1o**. The geometry optimized structures, along with calculated non-covalent interaction (NCI) surfaces of (a) *E*-**1o**, (b) TS-**1o**, and (c) *Z*-**1o**. Frontier molecular orbitals (FMOs) of (d) *E*-**1o** and (e) *Z*-**1o** and corresponding energies. NTO pairs ("hole" → "particle") of the $S_0 \rightarrow S_1$ and $S_0 \rightarrow S_1$ transitions of (f) *E*-**1o** and (g) *Z*-**1o** with percentage contributions stated.

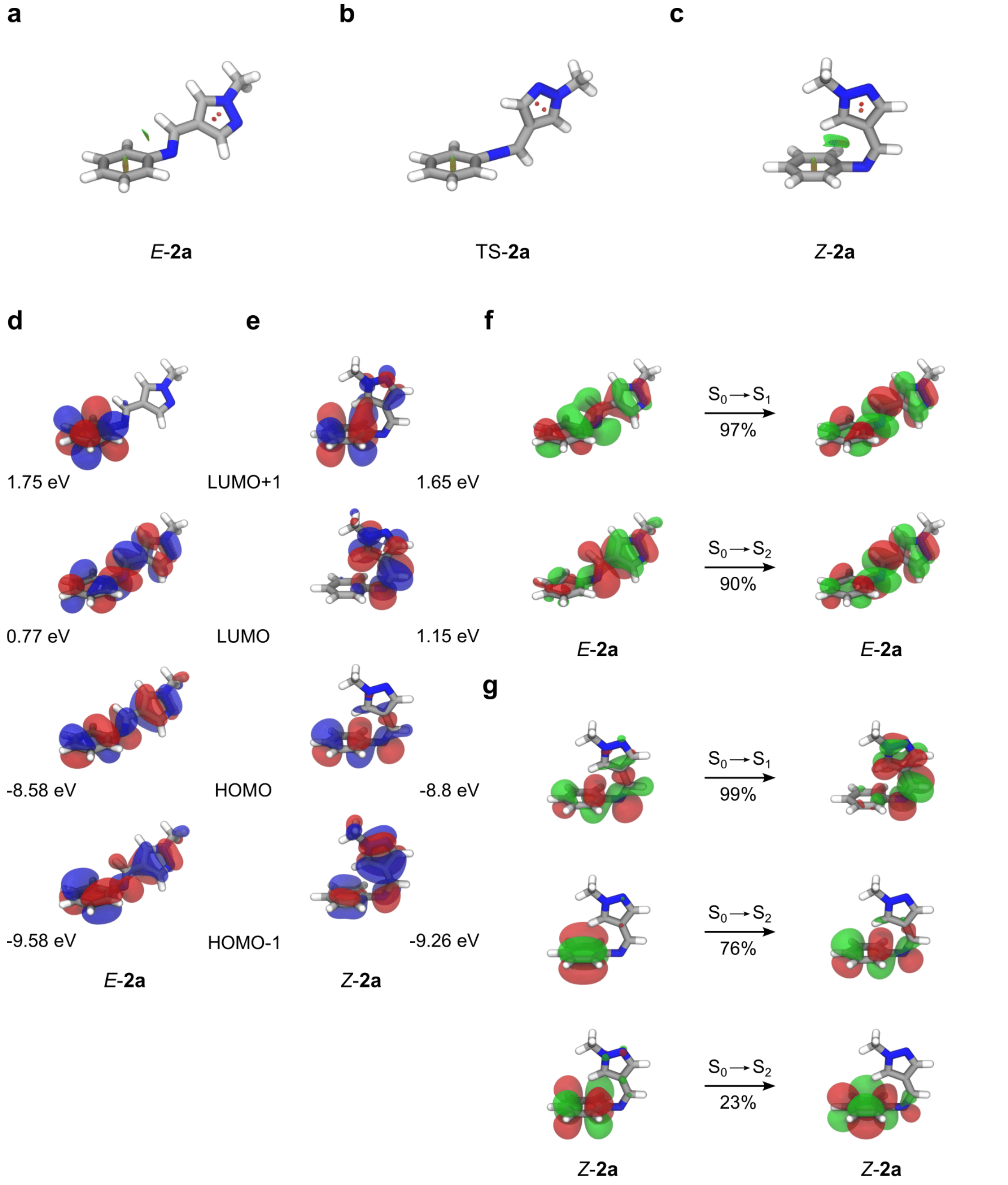

Figure S76. Overview of the theoretically calculated properties of **2a**. The geometry optimized structures, along with calculated non-covalent interaction (NCI) surfaces of (a) *E*-**2a**, (b) TS-**2a**, and (c) *Z*-**2a**. Frontier molecular orbitals (FMOs) of (d) *E*-**2a** and (e) *Z*-**2a** and corresponding energies. NTO pairs ("hole" → "particle") of the S_0→S_1 and S_0→S_1 transitions of (f) *E*-**2a** and (g) *Z*-**2a** with percentage contributions stated.

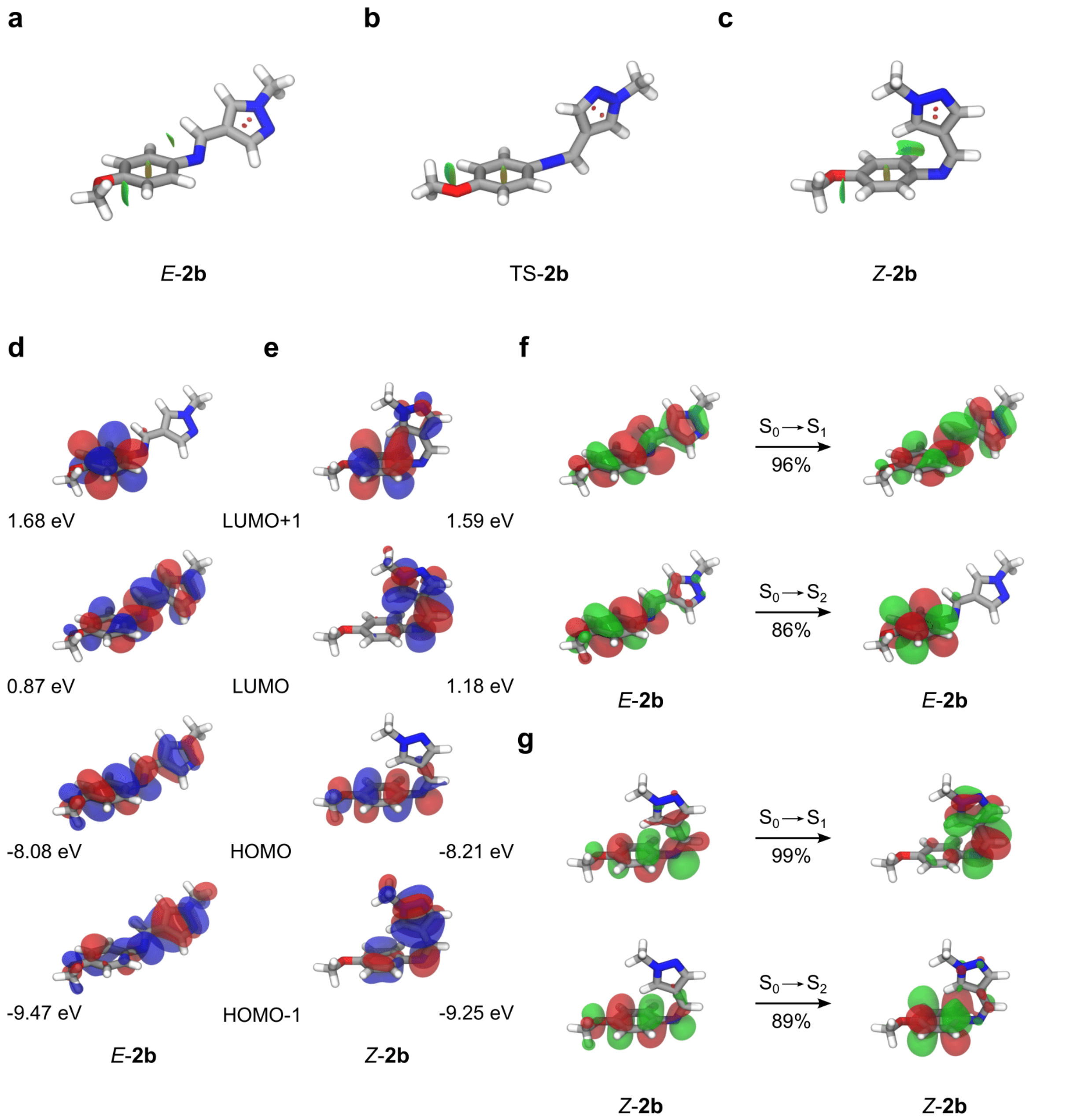

Figure S77. Overview of the theoretically calculated properties of **2b**. The geometry optimized structures, along with calculated non-covalent interaction (NCI) surfaces of (a) *E*-**2b**, (b) TS-**2b**, and (c) *Z*-**2b**. Frontier molecular orbitals (FMOs) of (d) *E*-**2b** and (e) *Z*-**2b** and corresponding energies. NTO pairs ("hole" → "particle") of the $S_0 \rightarrow S_1$ and $S_0 \rightarrow S_1$ transitions of (f) *E*-**2b** and (g) *Z*-**2b** with percentage contributions stated.

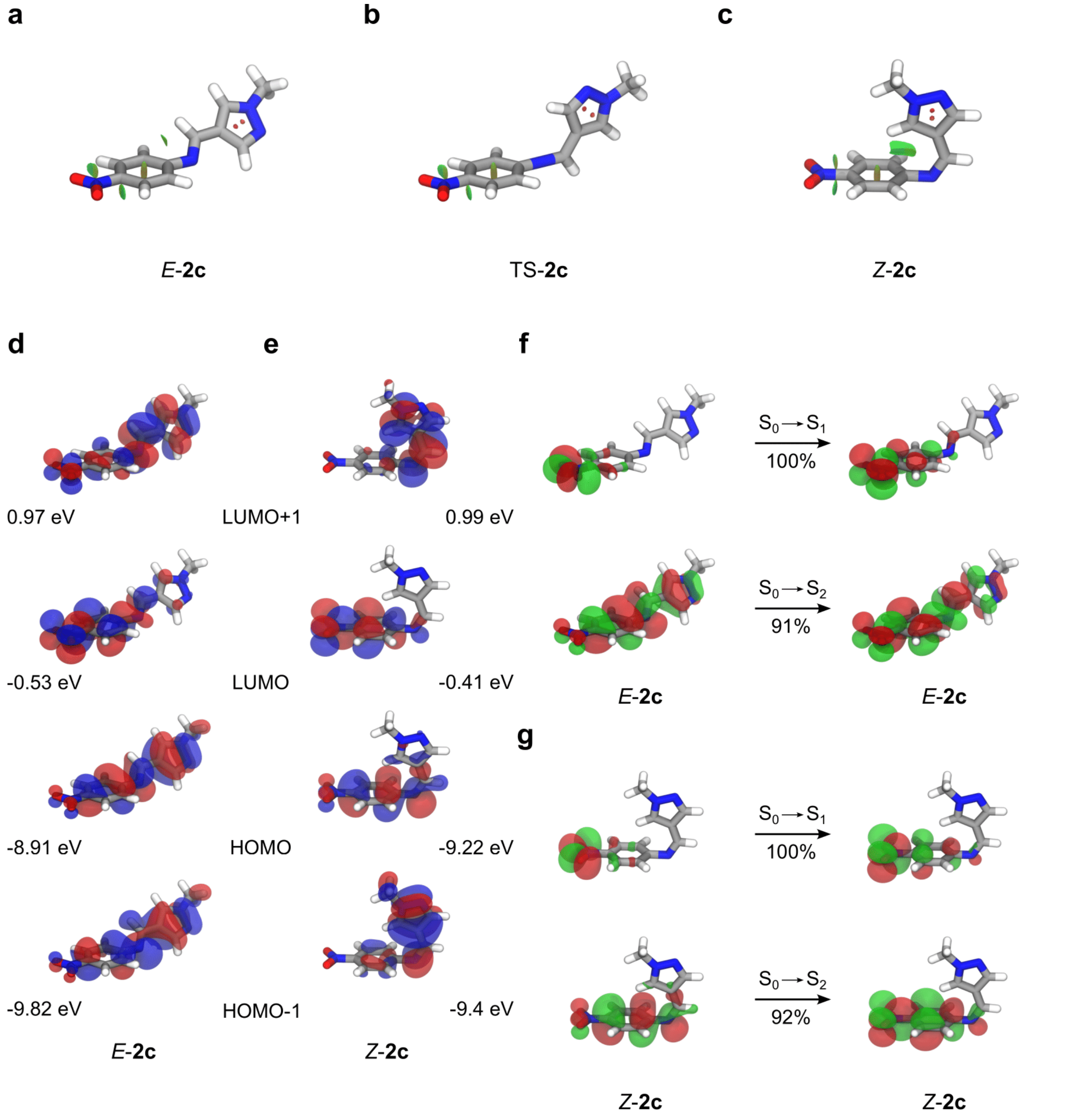

Figure S78. Overview of the theoretically calculated properties of **2c**. The geometry optimized structures, along with calculated non-covalent interaction (NCI) surfaces of (a) *E*-**2c**, (b) TS-**2c**, and (c) *Z*-**2c**. Frontier molecular orbitals (FMOs) of (d) *E*-**2c** and (e) *Z*-**2c** and corresponding energies. NTO pairs ("hole" → "particle") of the $S_0 \rightarrow S_1$ and $S_0 \rightarrow S_1$ transitions of (f) *E*-**2c** and (g) *Z*-**2c** with percentage contributions stated.

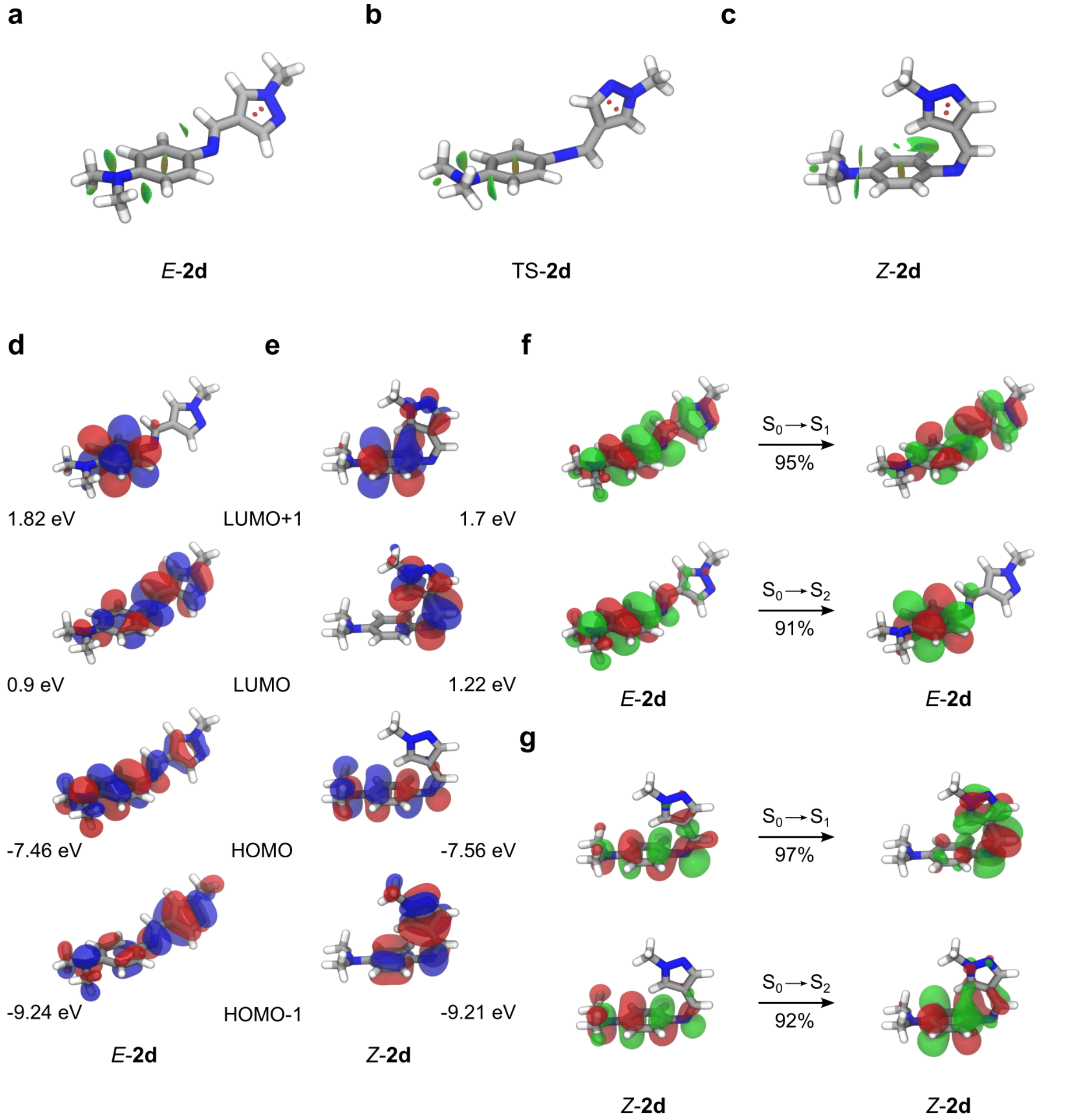

Figure S79. Overview of the theoretically calculated properties of **2d**. The geometry optimized structures, along with calculated non-covalent interaction (NCI) surfaces of (a) *E*-**2d**, (b) TS-**2d**, and (c) *Z*-**2d**. Frontier molecular orbitals (FMOs) of (d) *E*-**2d** and (e) *Z*-**2d** and corresponding energies. NTO pairs ("hole" → "particle") of the $S_0 \rightarrow S_1$ and $S_0 \rightarrow S_1$ transitions of (f) *E*-**2d** and (g) *Z*-**2d** with percentage contributions stated.

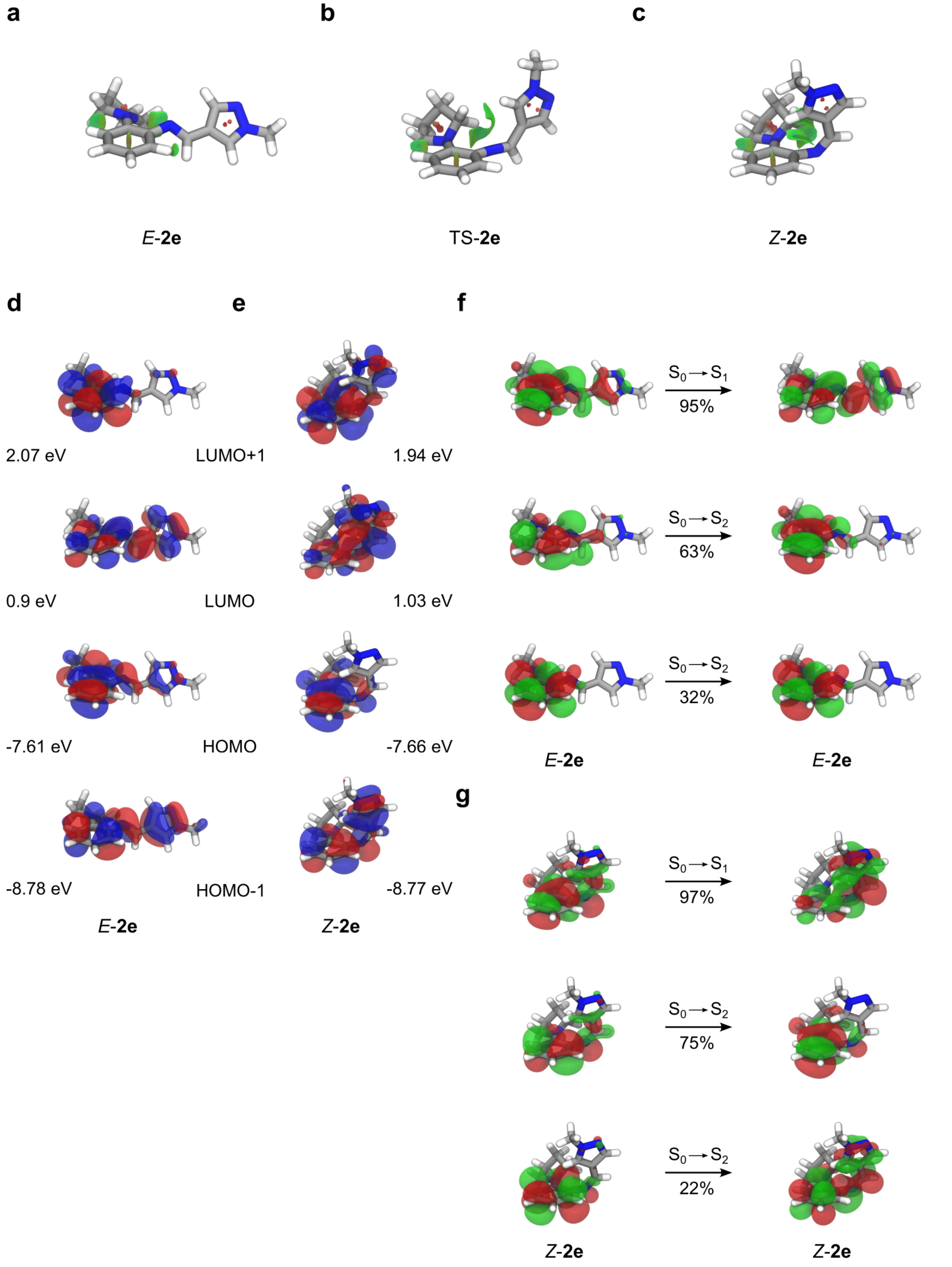

Figure S80. Overview of the theoretically calculated properties of **2e**. The geometry optimized structures, along with calculated non-covalent interaction (NCI) surfaces of (a) *E*-**2e**, (b) TS-**2e**, and (c) *Z*-**2e**. Frontier molecular orbitals (FMOs) of (d) *E*-**2e** and (e) *Z*-**2e** and corresponding energies. NTO pairs ("hole" → "particle") of the $S_0 \rightarrow S_1$ and $S_0 \rightarrow S_1$ transitions of (f) *E*-**2e** and (g) *Z*-**2e** with percentage contributions stated.

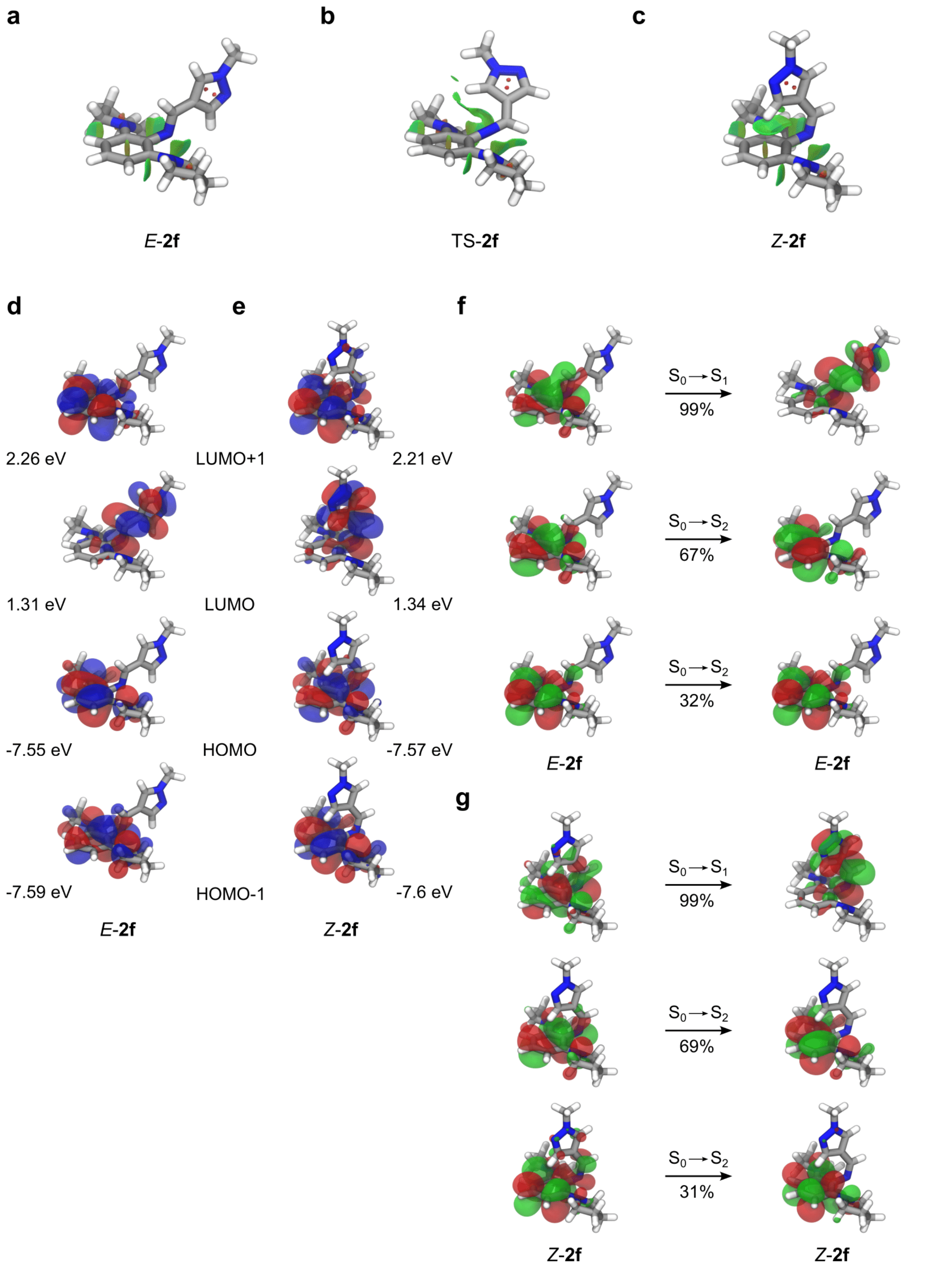

Figure S81. Overview of the theoretically calculated properties of **2f**. The geometry optimized structures, along with calculated non-covalent interaction (NCI) surfaces of (a) *E*-**2f**, (b) TS-**2f**, and (c) *Z*-**2f**. Frontier molecular orbitals (FMOs) of (d) *E*-**2f** and (e) *Z*-**2f** and corresponding energies. NTO pairs ("hole" → "particle") of the $S_0 \rightarrow S_1$ and $S_0 \rightarrow S_1$ transitions of (f) *E*-**2f** and (g) *Z*-**2f** with percentage contributions stated.

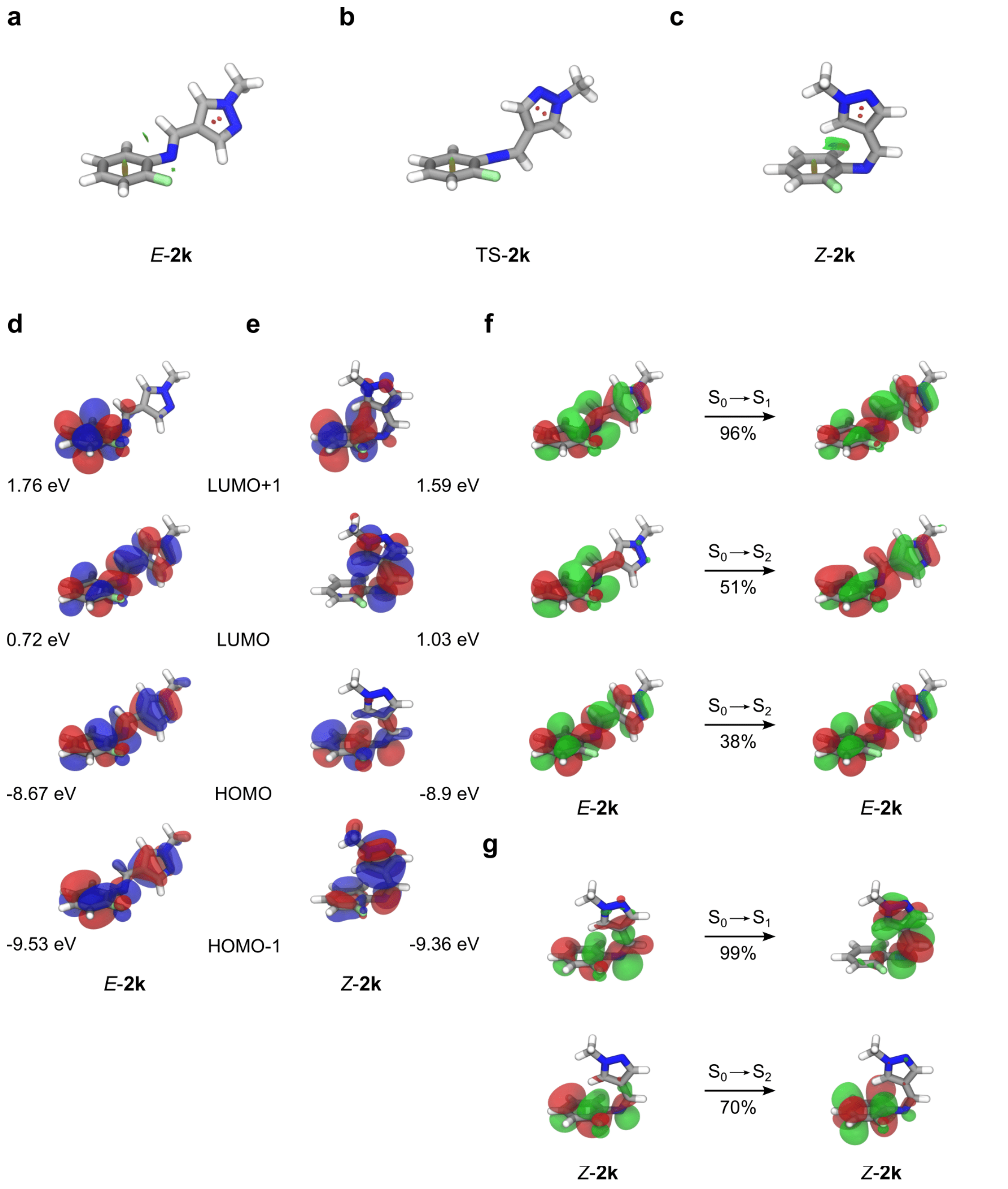

Figure S82. Overview of the theoretically calculated properties of **2k**. The geometry optimized structures, along with calculated non-covalent interaction (NCI) surfaces of (a) *E*-**2k**, (b) TS-**2k**, and (c) *Z*-**2k**. Frontier molecular orbitals (FMOs) of (d) *E*-**2k** and (e) *Z*-**2k** and corresponding energies. NTO pairs ("hole" → "particle") of the $S_0 \rightarrow S_1$ and $S_0 \rightarrow S_1$ transitions of (f) *E*-**2k** and (g) *Z*-**2k** with percentage contributions stated.

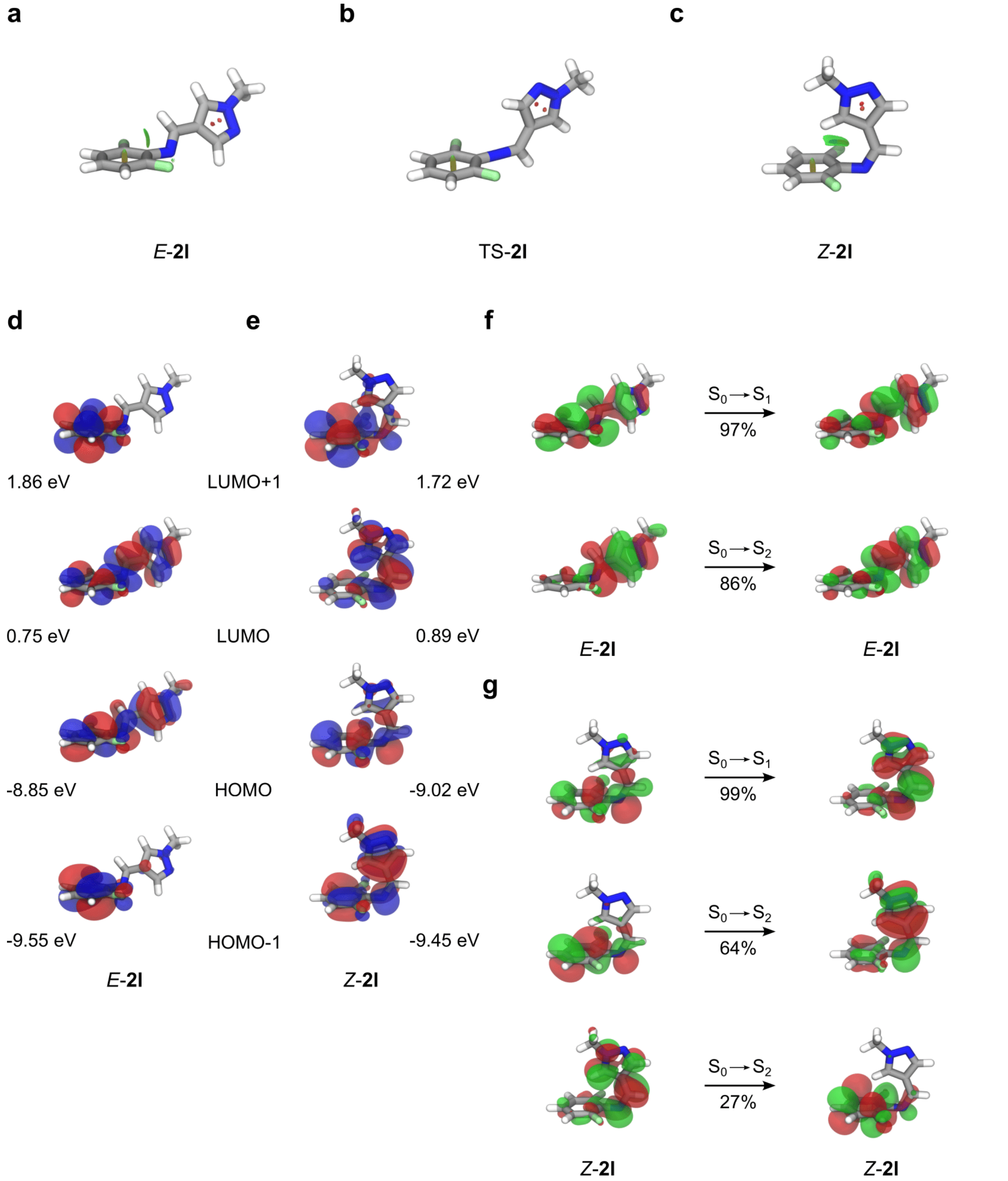

Figure S83. Overview of the theoretically calculated properties of **2l**. The geometry optimized structures, along with calculated non-covalent interaction (NCI) surfaces of (a) *E*-**2l**, (b) TS-**2l**, and (c) *Z*-**2l**. Frontier molecular orbitals (FMOs) of (d) *E*-**2l** and (e) *Z*-**2l** and corresponding energies. NTO pairs ("hole" → "particle") of the $S_0 \rightarrow S_1$ and $S_0 \rightarrow S_1$ transitions of (f) *E*-**2l** and (g) *Z*-**2l** with percentage contributions stated.

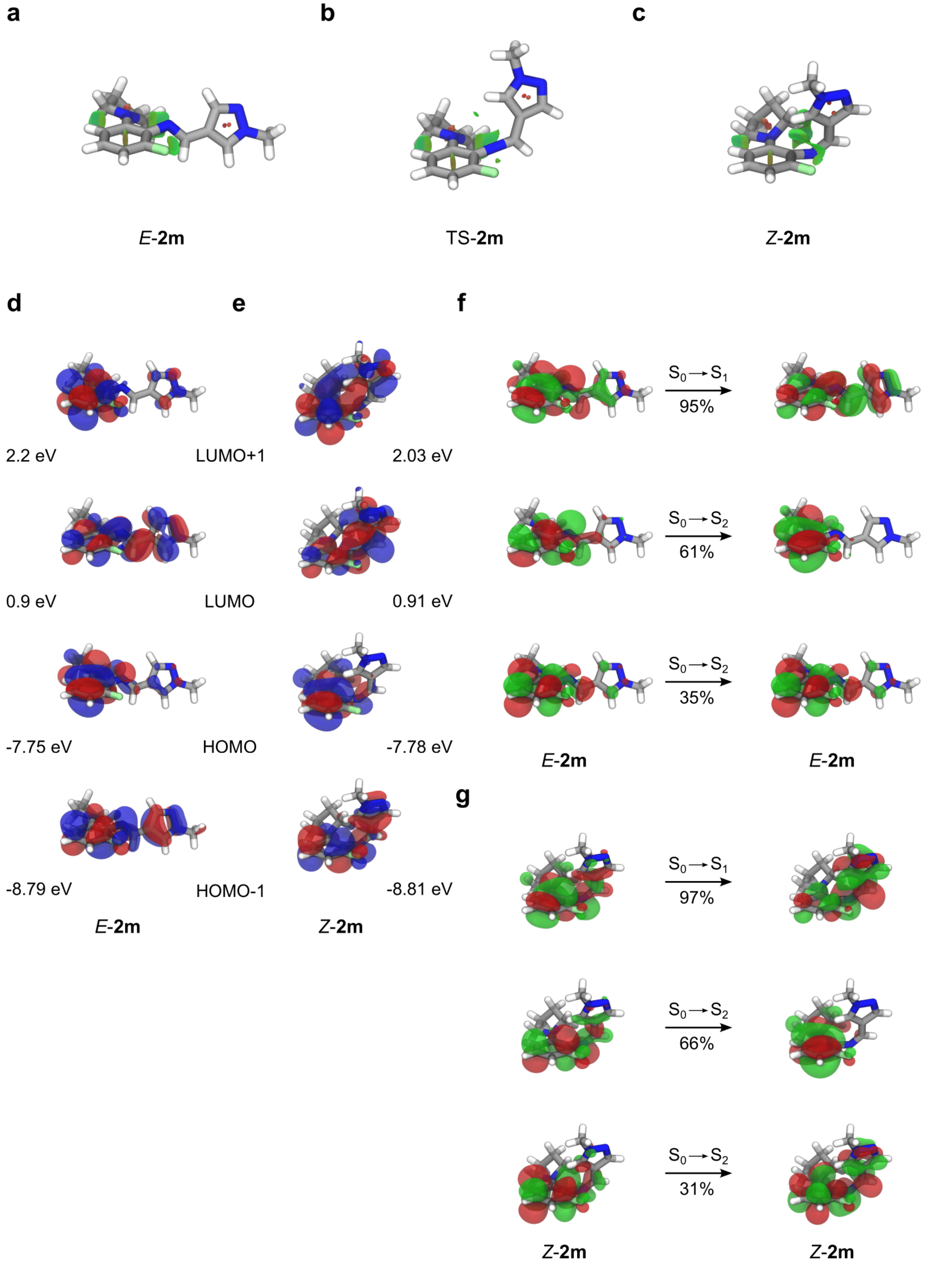

Figure S84. Overview of the theoretically calculated properties of **2m**. The geometry optimized structures, along with calculated non-covalent interaction (NCI) surfaces of (a) *E*-**2m**, (b) TS-**2m**, and (c) *Z*-**2m**. Frontier molecular orbitals (FMOs) of (d) *E*-**2m** and (e) *Z*-**2m** and corresponding energies. NTO pairs ("hole" → "particle") of the S_0→S_1 and S_0→S_1 transitions of (f) *E*-**2m** and (g) *Z*-**2m** with percentage contributions stated.

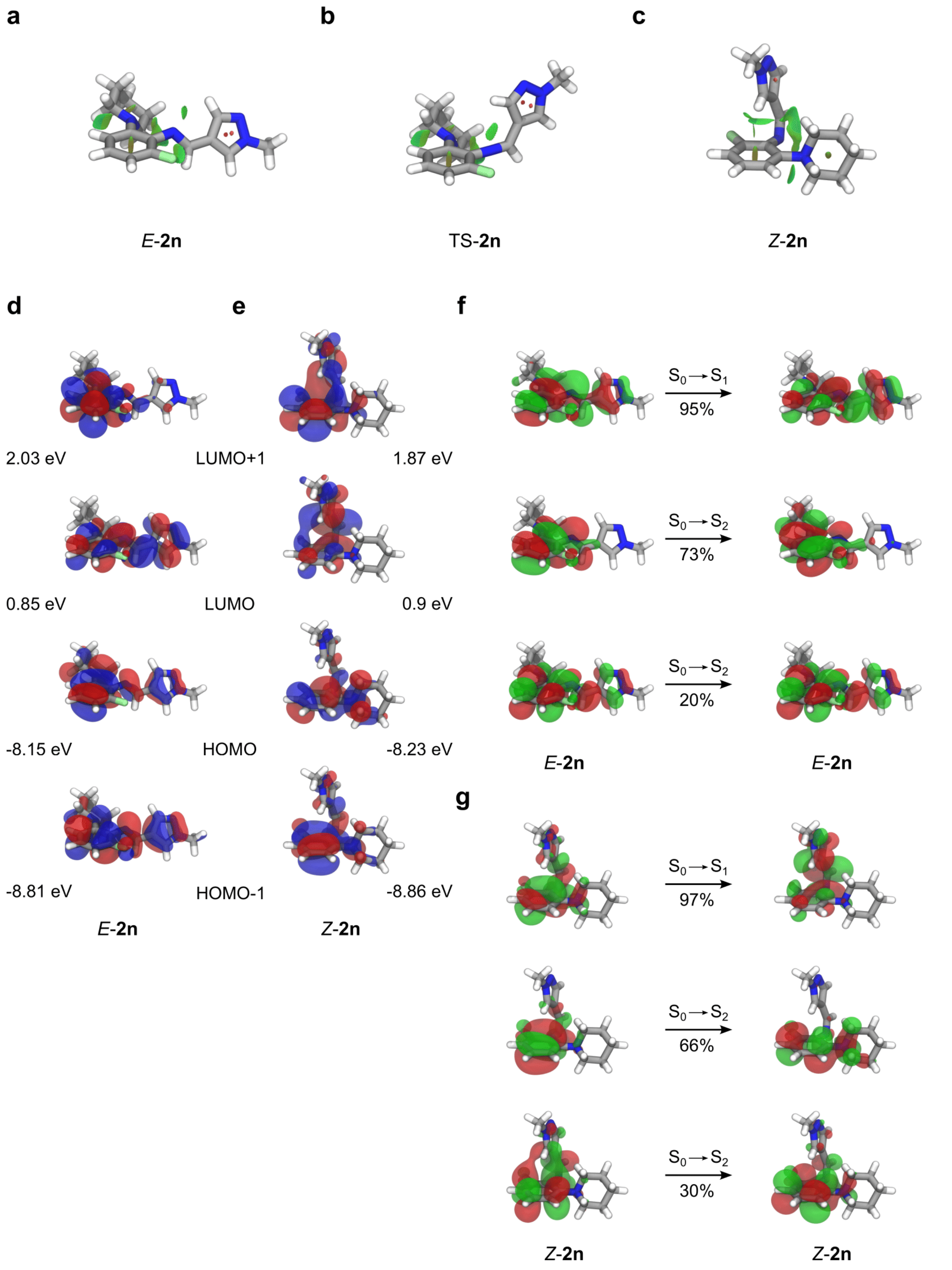

Figure S85. Overview of the theoretically calculated properties of **2n**. The geometry optimized structures, along with calculated non-covalent interaction (NCI) surfaces of (a) *E*-**2n**, (b) TS-**2n**, and (c) *Z*-**2n**. Frontier molecular orbitals (FMOs) of (d) *E*-**2n** and (e) *Z*-**2n** and corresponding energies. NTO pairs ("hole" → "particle") of the $S_0 \rightarrow S_1$ and $S_0 \rightarrow S_1$ transitions of (f) *E*-**2n** and (g) *Z*-**2n** with percentage contributions stated.

6 References

1 J. Wu, L. Kreimendahl, S. Tao, O. Anhalt and J. L. Greenfield, *Chem. Sci.*, 2024, **15**, 3872–3878.

2 K. Stranius and K. Börjesson, *Sci. Rep.*, 2017, **7**, 41145.

3 G. M. Sheldrick, *Acta Crystallogr. Sect. Found. Adv.*, 2015, **71**, 3–8.

4 V. Gold, Ed., *The IUPAC Compendium of Chemical Terminology: The Gold Book*, International Union of Pure and Applied Chemistry (IUPAC), Research Triangle Park, NC, 4th edn., 2019.

5 H. Eyring, *J. Chem. Phys.*, 1935, **3**, 107–115.

6 A. H. Heindl and H. A. Wegner, *Chem. Eur. J.*, 2020, **26**, 13730–13737.

7 E. Fischer, *J. Phys. Chem.*, 1967, **71**, 3704–3706.

8 C. G. Hatchard, C. A. Parker and E. J. Bowen, *Proc. R. Soc. Lond. Ser. Math. Phys. Sci.*, 1956, **235**, 518–536.

9 F. Neese, *WIREs Comput. Mol. Sci.*, 2012, **2**, 73–78.

10 F. Neese, *WIREs Comput. Mol. Sci.*, 2018, **8**, e1327.

11 F. Neese, F. Wennmohs, U. Becker and C. Riplinger, *J. Chem. Phys.*, 2020, **152**, 224108.

12 P. Pracht, F. Bohle and S. Grimme, *Phys. Chem. Chem. Phys.*, 2020, **22**, 7169–7192.

13 S. Grimme, *J. Chem. Theory Comput.*, 2019, **15**, 2847–2862.

14 S. Grimme, F. Bohle, A. Hansen, P. Pracht, S. Spicher and M. Stahn, *J. Phys. Chem. A*, 2021, **125**, 4039–4054.

15 E. R. Johnson, S. Keinan, P. Mori-Sánchez, J. Contreras-García, A. J. Cohen and W. Yang, *J. Am. Chem. Soc.*, 2010, **132**, 6498–6506.

16 J. Contreras-García, E. R. Johnson, S. Keinan, R. Chaudret, J.-P. Piquemal, D. N. Beratan and W. Yang, *J. Chem. Theory Comput.*, 2011, **7**, 625–632.

17 R. A. Boto, F. Peccati, R. Laplaza, C. Quan, A. Carbone, J.-P. Piquemal, Y. Maday and J. Contreras-García, *J. Chem. Theory Comput.*, 2020, **16**, 4150–4158.

18 W. Humphrey, A. Dalke and K. Schulten, *J. Mol. Graph.*, 1996, **14**, 33–38.

19 C. Bannwarth, E. Caldeweyher, S. Ehlert, A. Hansen, P. Pracht, J. Seibert, S. Spicher and S. Grimme, *WIREs Comput. Mol. Sci.*, 2021, **11**, e1493.

20 S. Grimme, C. Bannwarth and P. Shushkov, *J. Chem. Theory Comput.*, 2017, **13**, 1989–2009.

21 P. Pracht, E. Caldeweyher, S. Ehlert and S. Grimme, *ChemRxiv*, 2019, **29**, 8326202.

22 C. Bannwarth, S. Ehlert and S. Grimme, *J. Chem. Theory Comput.*, 2019, **15**, 1652–1671.

23 S. Grimme, A. Hansen, S. Ehlert and J.-M. Mewes, *J. Chem. Phys.*, 2021, **154**, 064103.

24 A. Najibi and L. Goerigk, *J. Comput. Chem.*, 2020, **41**, 2562–2572.

25 E. Caldeweyher, C. Bannwarth and S. Grimme, *J. Chem. Phys.*, 2017, **147**, 034112.

26 E. Caldeweyher, S. Ehlert, A. Hansen, H. Neugebauer, S. Spicher, C. Bannwarth and S. Grimme, *J. Chem. Phys.*, 2019, **150**, 154122.

27 E. Caldeweyher, J.-M. Mewes, S. Ehlert and S. Grimme, *Phys. Chem. Chem. Phys.*, 2020, **22**, 8499–8512.

28 F. Weigend and R. Ahlrichs, *Phys. Chem. Chem. Phys.*, 2005, **7**, 3297.

29 F. Weigend, *Phys. Chem. Chem. Phys.*, 2006, **8**, 1057.

30 V. Barone and M. Cossi, *J. Phys. Chem. A*, 1998, **102**, 1995–2001.
